# Bifunctional Catalysis of a Crossed Aldol Condensation by Diamines: Impact of Tether Composition and Length

**DOI:** 10.1021/acs.joc.6c00659

**Published:** 2026-06-05

**Authors:** Philip P. Lampkin, R. Charles Roberts, Bianca Czeslawski, Samuel H. Gellman

**Affiliations:** Department of Chemistry, University of Wisconsin−Madison, 1101 University Avenue, Madison, Wisconsin 53706, United States

## Abstract

The crossed aldol condensation between hydrocinnamaldehyde and 2,6-dimethoxybenzaldehyde was used to explore bifunctional catalysis by a set of diamines. The catalytic mechanism appears to involve nucleophilic activation of hydrocinnamaldehyde, as the enamine, and electrophilic activation of 2,6-dimethoxybenzaldehyde, as the iminium. Our goal was to learn how catalytic efficacy is influenced by variations in the molecular scaffold that displays the two primary amine groups. One set of diamines contained oligomethylene linkers of 4 to 16 carbons, and another set contained oligoether linkers (−O–CH_2_–CH_2_– repeat unit) of 5 to 20 atoms. These flexible diamines were compared with a previously described peptide-based catalyst in which a helical conformation induces spatial proximity of the two amine groups. The most effective catalysis among the oligomethylene-linked diamines was observed for tethers of 12 to 16 carbon atoms. Among the oligoether-linked diamines, the most effective catalysis was observed for tethers of 8 to 14 atoms. The helix-forming α/β-peptide, with 15 atoms between the two amine groups, was superior to oligomethylene-linked diamines with tethers of comparable lengths, but the α/β-peptide diamine was matched in catalytic prowess by oligoether diamines. The superiority of oligoether tethers relative to oligomethylene tethers is attributed to conformational differences between the tethers. Collectively, the data support the conclusion that relatively long, flexible tethers can enable a pair of reactive groups to function cooperatively, at least when each reactive group forms a covalent intermediate.

## Graphical abstract

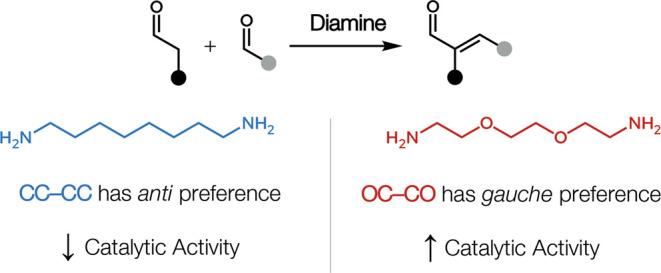

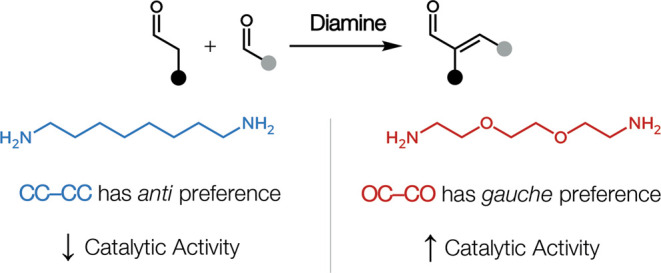

## Introduction

Bifunctional catalysts facilitate chemical transformations through the coordinated action of two reactive units arranged on a molecular scaffold.^1^ Considerable effort has been devoted to the development of bifunctional catalysts. Most synthetic examples are based on compact scaffolds that rigidly connect reactive groups.^2–10^ The properties of bifunctional catalysts with flexible scaffolds have received less attention.^11–16^

We recently described flexible dihydrazides that act as bifunctional catalysts for homoaldol condensations (Figure 1A).^17^ These catalysts, in which two cyclic hydrazide units are connected by an oligomethylene tether, appear to drive reaction through simultaneous electrophilic and nucleophilic activation of aldehydes. The dihydrazide with the highest catalytic activity had a 10-methylene tether. We hypothesized that intramolecular carbon–carbon (C–C) bond formation was the rate-determining step for the dihydrazide-catalyzed aldol condensation. This hypothesis is consistent with prior studies of aminocatalyzed aldol reactions, which identified C–C bond formation as rate determining.^18–20^ For the dihydrazide with a 10-methylene tether, C–C bond formation would lead to transient formation of a 19-membered macrocycle.

**Figure 1:**
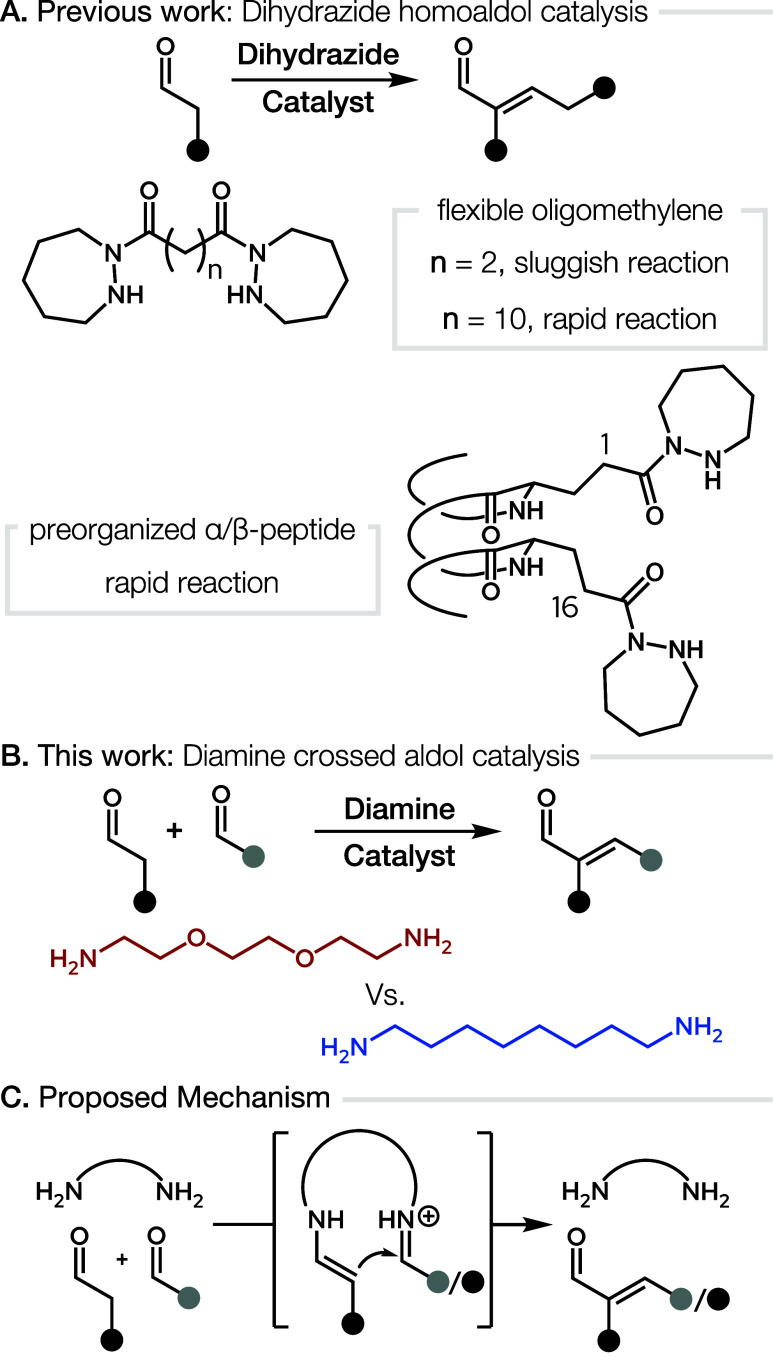
(A) Catalysis of homoaldol reactions by dihydrazides with oligomethylene and α/β-peptide scaffolds. (B) Bifunctional catalysis of crossed aldol condensations using diamines linked by oligomethylene and oligoether tethers. (C) Proposed mechanism for crossed aldol condensations catalyzed by diamines.

Results with flexible dihydrazide catalysts provided a basis for evaluating the effects of linker preorganization.^21^ This effort employed peptides containing both α- and β-amino acid residues with a repeating αββ pattern in the backbone. The β residues were locally preorganized with a five-membered ring constraint. This α/β-peptide family strongly favors a helical conformation with approximately three residues per turn.^22^ Placing hydrazide units within side chains of successive α residues (*i*,*i*+3 sequence spacing) was therefore expected to result in alignment of the reactive diad along one side of the helix. This prediction was validated with a series of crystal structures.^21^ The most effective α/β-peptide catalyst was modestly more active than the best flexible dihydrazide, as judged by relative initial rates.

In the work described here, we asked whether the trends observed for flexible vs preorganized dihydrazide catalysts of homoaldol condensations would carry over to diamine catalysts of a crossed aldol condensation (Figure 1B,C). We previously observed that a primary amine catalytic diad was effective for crossed aldol condensations featuring an aryl aldehyde as the electrophilic component.^23^ Diverse examples were demonstrated, including reactions leading to macrocycle formation.

The present studies focused on reaction of hydrocinnamaldehyde with 2,6-dimethoxybenzaldehyde. We focused on a single substrate pair in order to compare α,ω-diamino alkanes of varying length with *n*-butylamine to probe for bifunctional catalysis. Reaction outcomes from this flexible diamine series were compared with the performance of a helical α/β-peptide that displays a primary amine diad; this α/β-peptide was identified as the most effective catalyst of crossed aldol reactions in our previous study.^23^ Consistent with the previous homoaldol study involving dihydrazides,^21^ the α/β-peptide was modestly more effective than α,ω-diamino alkanes with similar numbers of bonds between the primary amine groups.

We used the crossed aldol reaction to extend the analysis of flexible linker composition to include oligoethers with an −O–CH_2_–CH_2_– repeat unit. α,ω-Diamines in the oligoether series were superior to analogues in the oligomethylene series. The catalytic enhancement likely arises from conformational differences between oligomethylene and oligoether segments. The best oligoether diamines were comparable in activity to the α/β-peptide with a primary amine diad. These findings highlight the promise of oligoether units for future exploration of bifunctional catalysis.

## Results

### Reactivity Assessment via Yield at a Fixed Time

An initial survey of catalytic activities was conducted by comparing crossed aldol product yields under a defined set of conditions (Figure 2). Because our goal was to elucidate correlations between diamine structure and catalytic activity, rather than to develop a new synthetic method, all experiments involved a single pair of substrates, hydrocinnamaldehyde (**A**_**N**_) as the nucleophile and 2,6-dimethoxybenzaldehyde (**A**_**E**_) as the electrophile, at constant initial concentrations of 5 mM and 6.25 mM, respectively. Other reaction conditions were inspired by the work of Erkkilä and Pihko,^18^ including use of a mixture of 96:4 isopropanol:water as solvent and inclusion of 10 mM triethylamine and 50 mM propionic acid (2 and 10 equiv., respectively, relative to hydrocinnamaldehyde). The relatively high concentration of the acid additive was found to maximize catalytic activity (Figure S7). Addition of 2 equiv of triethylamine provided enhanced catalytic activity relative to reactions conducted with 0 or 4 equiv of triethylamine (Figure S8). Each reaction was conducted at 37 °C for 24 h. The acid and base additives presumably facilitate the many proton-transfer steps required for the aldol condensation mechanism.^24^

**Figure 2:**
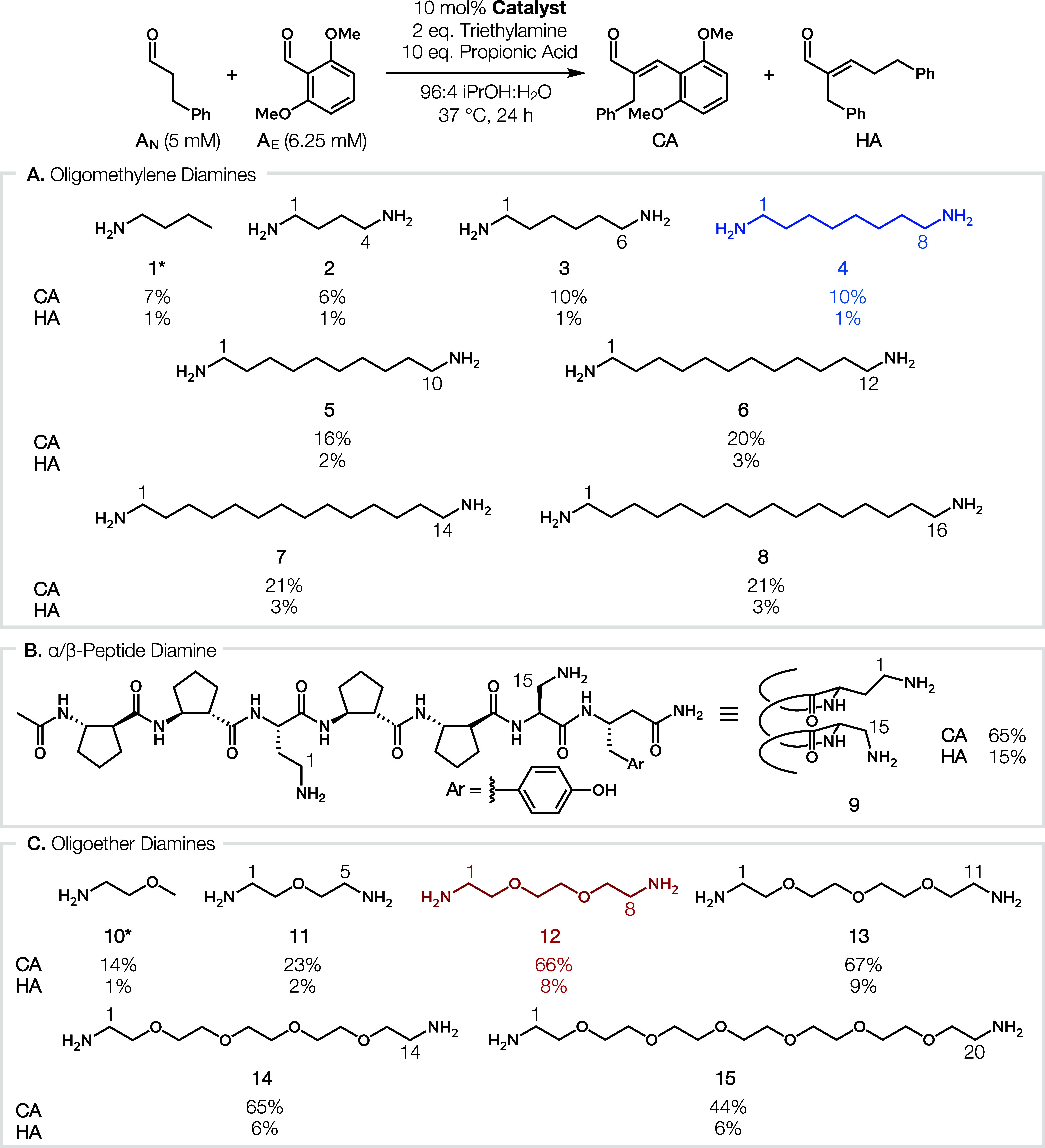
Crossed aldol (**CA**) and homoaldol (**HA**) product yields determined by UPLC using an internal standard after 24 h of reaction with (A) oligomethylene, (B) α/β-peptide or (C) oligoether diamines **1** to **15**. (*) = 20 mol % catalyst used.

A series of α,ω-diamino alkanes (**2–8**) was evaluated for catalysis of the crossed aldol condensation, based on comparison with *n*-butylamine (**1**) (Figure 2A). Each diamine was introduced at 0.5 mM (10 mol % relative to hydrocinnamaldehyde), and *n*-butylamine was introduced at 1 mM (20 mol % relative to hydrocinnamaldehyde); thus, the total concentration of primary amino groups was constant across these reactions. The major product of each reaction was the result of crossed aldol condensation (**CA**). A small amount of the homoaldol condensation product (**HA**) was isolated as well. No β-hydroxy aldehydes were observed, which presumably reflects a Mannich-type mechanism.^25,26^ No other products were detected (Figure S5). In each case, the aldol condensation product was generated predominantly as the *E* alkene isomer, but trace amounts of the *Z* isomer were detected. Yield determinations reflect the sum of the *E* and *Z* isomers for **CA** and for **HA**, as shown in Figure 2A.

α,ω-Diamine **2**, with the shortest linker (four methylenes), was no more effective at promoting aldol condensation than monoamine **1**. This observation is consistent with previous findings in the dihydrazide series for homoaldol condensation catalysis.^17^ Small increases in **CA** yield were observed as the linker was lengthened to six or eight methylenes (**3** or **4**). Further lengthening provided more significant increases in **CA** yield, which were maximal (∼20%) for linkers of 12, 14, or 16 methylenes (**6–8**). These findings are consistent with previous trends among dihydrazides in that catalysis becomes more effective as the linker grows. However, a clear maximum was observed in the dihydrazide series, while no maximum was evident among the α,ω-diamines. It is possible that yield would decline with α,ω-diamino alkanes longer than **8**.

To evaluate the effect of linker preorganization, we examined α/β-peptide **9**, which bears a primary amine diad (Figure 2B). The *i*,*i*+3 sequence relationship of the two amine-bearing side chains causes alignment in the helical conformation favored by this α/β-peptide backbone. This α/β-peptide was previously shown to catalyze crossed aldol condensations between alkyl and aryl aldehydes.^23^ α/β-Peptide **9** was more effective at promoting crossed aldol condensation than the best of the α,ω-diamino alkanes, with the peptide providing 65% **CA** yield vs 21% for **7** or **8**. There are 14 atoms between the amino groups in **7**, 16 intervening atoms in **8** and 15 intervening atoms in α/β-peptide **9**. The improved **CA** yield with catalyst **9** suggests that preorganization of the reactive diad provided by the helical peptide backbone leads to a modest increase in catalytic efficacy, which is consistent with results from comparable reactions involving dihydrazide catalysts of homoaldol condensation.^17^ The α/β-peptide catalyst appeared to be somewhat less selective for crossed aldol vs homoaldol condensation relative to α,ω-diamine **7** or **8** (**CA**:**HA** ∼ 4 for the α/β-peptide vs 7 for the flexible diamines).

We explored a second set of flexible diamines in which the linkers contained −O–CH_2_–CH_2_– units rather than exclusively methylene (−CH_2_−) units (Figure 2C). Monoamine **10** was selected as a basis for comparison with oligoether diamines **11–15**, which feature 5, 8, 11, 14, or 20 atoms between the amine groups. Ether-containing monoamine **10** was more effective at catalyzing the crossed aldol condensation relative to homologue **1**, as judged by yield of **CA** (14% for **10** vs 7% for **1**). Each of the five oligoether diamines was more effective than monoamine **10** under conditions that held the total concentration of primary amine groups constant. Oligoether diamines **12–14**, with linkers of 8–14 atoms, were most effective as crossed aldol condensation catalysts, with **CA** yields of ∼66% in each case. Catalytic efficacy declined with further linker extension to 20 atoms in oligoether diamine **15**, but even in this case the diamine provided a superior **CA** yield (44%) relative to monoamine **10** (at double the concentration). Extending the oligoether segment to 23 atoms led to a modest decline in **CA** yield, with relatively little effect upon further extension to 29 or 35 atoms (Table S3). It is notable that flexible oligoether diamine **14**, with 14 atoms between the two primary amine groups, was comparable to preorganized α/β-peptide catalyst **9**, with 15 atoms between the primary amine groups, in terms of **CA** yield.

### Reactivity Assessment via Relative Initial Rates

To gain further insight on the relationship between linker identity and catalytic activity, we measured initial rates of **(** ***E*** **)-CA** formation for selected diamines, using the conditions introduced above, which included 0.5 mM diamine. For comparison, we measured initial rates for 1 mM monoamine **1** or **10**. All other initial rates were normalized to that of monoamine **1**, and the resulting relative initial rate values (ν_REL_) provided the basis for our comparisons (Figure 3).

**Figure 3:**
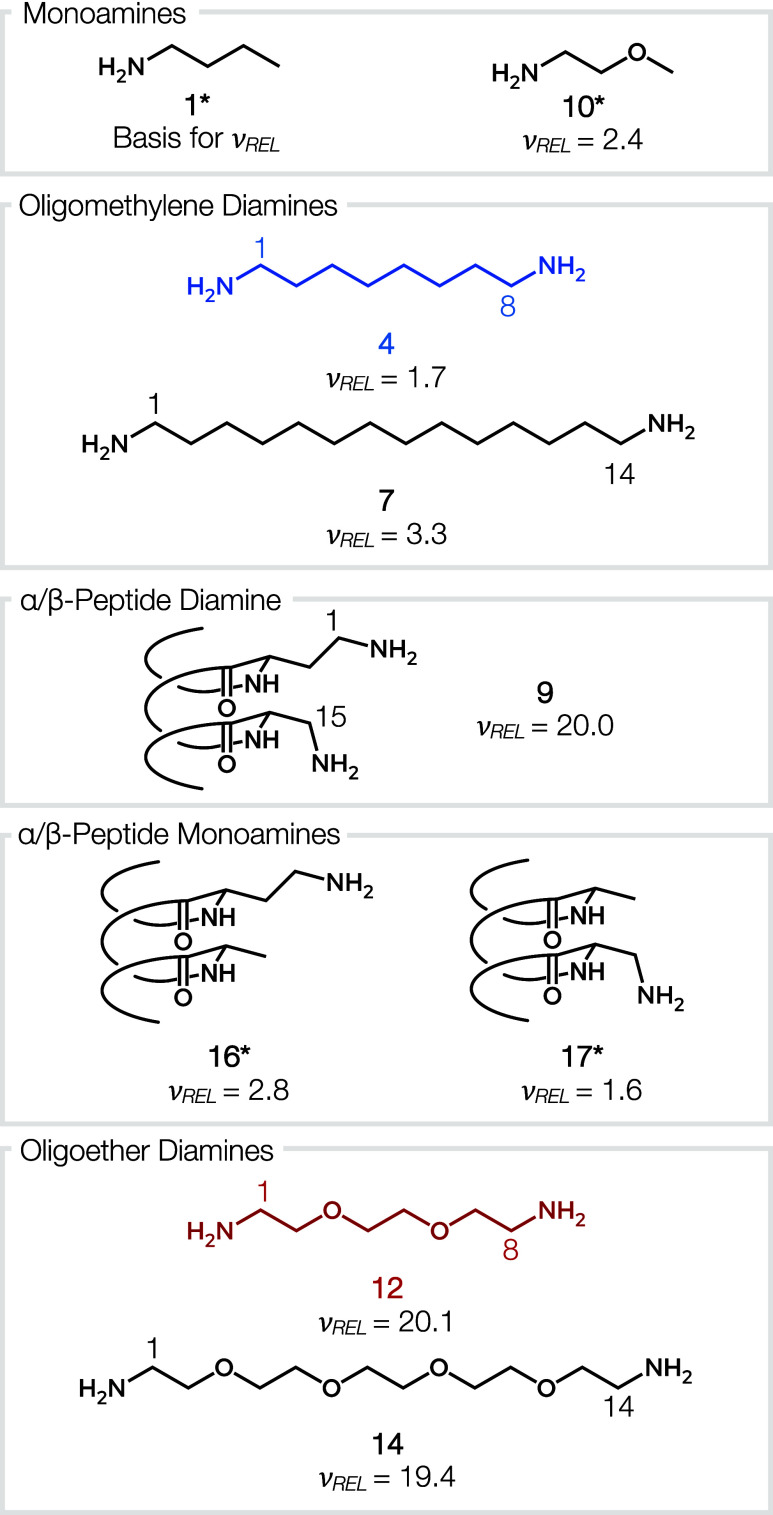
Relative initial rates of **CA** formation (ν_REL_) for monoamine and diamine catalysts measured under the standard reaction conditions. Initial rates of **CA** formation were quantified by UPLC using an internal standard. (*) = 20 mol % catalyst used.

The trends among ν_REL_ data paralleled those among yield data (Figure 2). Thus, α,ω-diamine **4**, with 8 methylenes between amine groups, was slightly more active than monoamine **1**, while α,ω-diamine **7**, with a 14-methylene linker, was more reactive than the shorter α,ω-diamine. α/β-Peptide **9**, with a preorganized 15-atom linker between the amine groups, was significantly more active (ν_REL_ = 20) than either of the α,ω-diamino alkanes. To gain additional perspective on the contribution of preorganization, we analyzed two α/β-peptide monoamines, **16** and **17**, that resulted from replacing one or the other of the amine-bearing α-amino acid residues in **9** with alanine. At double the concentration of **9**, each α/β-peptide monoamine was slightly more active than *n*-butylamine (**1**): ν_REL_ was 2.8 for α/β-peptide **16**, bearing only a 2,4-diaminobutanoic acid residue, and ν_REL_ was 1.6 for α/β-peptide **17**, bearing only a 2,3-diaminopropanoic acid residue. It is unclear why these monoamine α/β-peptides are slightly more active catalysts than *n*-butylamine. If these ν_REL_ values are used to normalize the value for the α/β-peptide diamine, then the benefit of linker preorganization in terms of catalytic activity is between 7-fold and 12-fold.

Comparison of monoamine activities via ν_REL_ revealed that ether-containing monoamine **10** was modestly more active than *n*-butylamine (**1**), which is consistent with the conclusion derived from yield data. Oligoether diamines **12** and **14**, with 8 or 14 atoms between the amine groups, displayed considerably higher activity (∼20-fold) relative to *n*-butylamine, but the benefit of linking the two amine groups with an oligoether unit was less pronounced (∼8-fold) relative to ether-containing monoamine **10**. The similarity in ν_REL_ values for different spacer lengths paralleled the similarity in **CA** yield for oligoether diamines **12** and **14**. In addition, the ν_REL_ comparisons mirrored the yield comparisons in showing that conformationally preorganized α/β-peptide **9** and flexible oligoether **14**, with similar numbers of atoms in the linking segments, are indistinguishable in their ability to catalyze the crossed aldol condensation.

Oligoether diamine catalysis might be enhanced by cation chelation, which could preorganize the oligoether backbone to position the amine groups near one another (Figure S9). We tested this hypothesis by evaluating the effect of LiClO_4_, NaClO_4_ or KClO_4_ on ν_REL_ for formation of **CA** with either **14** or **15** as catalyst. None of the salt additives exerted a significant effect on ν_REL_ for either diamine (Tables S1 and S2), which seems to invalidate the chelation hypothesis.

### Exploration of Catalyst Order

We hypothesized that the diamines explored here function as bifunctional catalysts, with simultaneous activation of **A**_**N**_ via enamine formation and **A**_**E**_ via iminium formation (Figure 1C). Erkkilä and Pihko previously proposed dual activation in pyrrolidine-catalyzed crossed aldol reactions involving formaldehyde as the electrophile.^18^ By extension, we wondered whether dual activation of **A**_**N**_ and **A**_**E**_ might occur with a monoamine such as **1** (“sibling catalytic species”).^27^ To address these questions, we undertook Variable Time Normalization Analysis (VTNA)^28,29^ based on **CA** formation in reactions catalyzed by monoamine **1** or oligoether diamine **12**.

Our VTNA studies required measurement of **CA** formation over time for a series of reactions in which the concentration of monoamine **1** or diamine **12** was varied while other conditions described above were held constant (Figure 4). For monoamine **1**, the concentration was varied between 0.05 mM and 4.25 mM; data from measurements with 13 concentrations were used, and reactions were monitored for up to 11 h. Because of the low activity of this catalyst, it was not practical to allow these reactions to approach completion. For diamine **12**, concentration was varied between 0.0625 and 0.625 mM; data from measurements with 6 concentrations were used, and reactions were monitored for up to 7 h.

**Figure 4:**
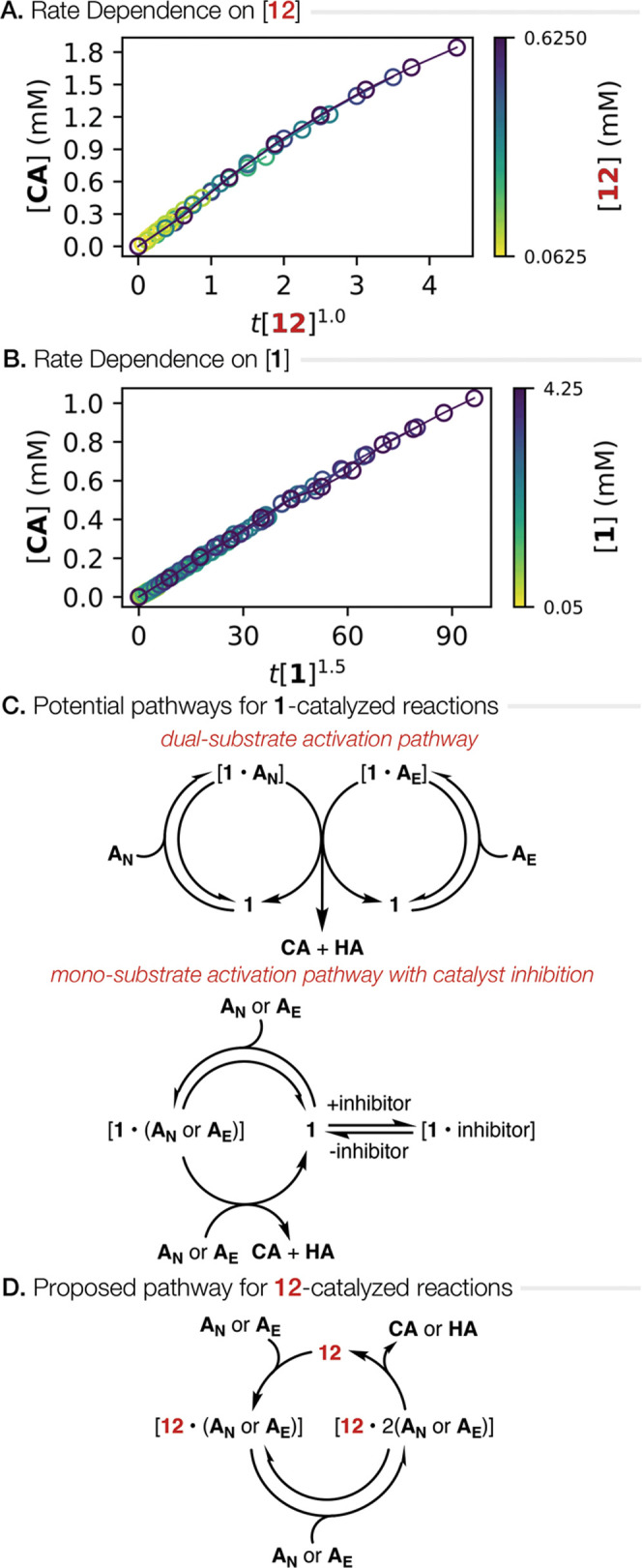
Approximately (A) 1.0-order kinetic dependence on diamine **12** and (B) 1.5-order kinetic dependence on monomamine **1** derived by VTNA. (C) Potential mechanistic pathways for reactions catalyzed by monoamine **1**: dual-substrate activation involving sibling catalytic species and monosubstrate activation with catalyst inhibition. (D) Proposed bifunctional pathway for reactions catalyzed by diamine **12**.

Our implementation of VTNA involved overlay of **CA** concentration profiles obtained with different catalyst concentrations plotted against the normalized time function *t*[catalyst]^γ^, where *t* is time, and the exponent γ is order in catalyst at the rate-determining step. This expression for the normalized time function requires that the catalyst is not deactivated during the reaction process, i.e., that the catalyst concentration remains constant.^30^ Evidence against deactivation of an amine catalyst under the crossed aldol conditions is presented in the Supporting Information (Figures S27–S32). The value of γ that causes the **CA** concentration profiles to overlay suggests the catalyst order. Burés has articulated the benefits of this visual analysis strategy, which makes use of all product concentration measurements across the set of varying conditions for a given catalyst.^28–30^

For oligoether diamine **12**, profile overlay was achieved for γ = 1.0 (Figure 4A). This outcome suggests first order catalysis by the diamine. Collectively, the VTNA result along with the evident superiority of diamine **12** in terms of **CA** yield and ν_REL_ relative to monoamine **1** or **10** are consistent with the hypothesis that diamine **12** functions as a bifunctional catalyst. We propose that **12** activates both the nucleophile (**A**_**N**_) and the electrophile (**A**_**E**_) for rate-determining formation of the C–C bond in the crossed aldol condensation (Figure 4D). By extrapolation, we propose that other diamines discussed here, including oligomethylene diamine **4** and α/β-peptide diamine **9**, also function as bifunctional catalysts. It is possible that an intermolecular pathway contributes to the overall reaction with these diamines, but the VTNA results with diamine **12** suggest that this pathway is unlikely to account for a significant proportion of product formation.

Evidence supporting the bifunctional catalysis hypothesis were obtained from trapping experiments involving diamine **4**. This reaction was set up under conditions summarized in Figure 2, but with a larger amount of diamine **4** (80 mol %). After 1 h, 5 equiv. NaBH_4_ was added to the reaction mixture to reduce enamine or iminium species to the corresponding amines. This trapping reaction mixture was analyzed by ultraperformance liquid chromatography–mass spectrometry (UPLC-MS). Masses consistent with the reduced forms of intermediates in the proposed bifunctional catalysis pathway, and other intermediates, were detected (Figures S20–S26).

By extrapolation from the in-depth analysis of diamines **4** and **12**, we hypothesize that oligomethylene-linked diamines **3–8** and oligoether-linked diamines **11–15**, as well as the longer diamines shown in Table S3, all function as bifunctional catalysts of the crossed aldol condensation; each of these diamines produces a higher product yield at 24 h relative to the corresponding monoamine (**1** or **10**; Figure 2, and Table S3). We further hypothesize that formation of the C–C bond is the rate-determining step for each diamine catalyst. This hypothesis suggests that the rate-determining step for each diamine involves transient closure of a large ring, containing from 10 atoms, for diamine **11**, to 38 atoms (Table S3). The ability of even quite long flexible diamines to surpass the corresponding monoamine is consistent with trends in a variety of macrocyclization reactions.^31,32^

For monoamine **1**, profile overlay was achieved for γ = 1.5 (Figure 4B). This result could suggest dual substrate activation via enamine and iminium as sibling catalytic species (Figure 4C). Alternatively, this outcome could imply first-order catalytic activation accompanied by partial catalyst inhibition. We favor the dual activation hypothesis because initial rate data are inconsistent with irreversible inactivation of **1**, and we could find no evidence that **CA**, **HA**, **A**_**N**_ or **A**_**E**_ inhibited the reaction (Figures S33–S38). The finding that catalysis by monoamine **1** is not first-order supports our hypothesis that diamine **12** and, by extension, the other diamines we have examined act as bifunctional catalysts.

### Computational Analysis: Oligoether vs Oligomethylene Conformations

Comparison of diamine series **2–8** and **10–15** revealed that catalysis of the crossed aldol condensation is more effective with the oligoether linkers than with the oligomethylene linkers. We hypothesize that this difference arises from conformational differences between the two types of linker. In the oligomethylene linkers, each CH_2_CH_2_—CH_2_CH_2_ torsional unit will prefer an anti conformation (torsion angle ∼ 180°).^33^ There are two types of torsional unit in the oligoether linkers, CH_2_O—CH_2_CH_2_ and OCH_2_—CH_2_O. CH_2_O—CH_2_CH_2_ prefers an *anti* conformation, but OCH_2_—CH_2_O prefers *gauche* conformations (torsion angle ∼ 60°).^33,34^ Thus, the most stable conformation of an oligomethylene linker should place the two amine groups too far from one another to support a bifunctional catalytic mechanism.^35–38^ In contrast, an oligoether linker should have multiple low-energy conformations that allow the two terminal nitrogen atoms to approach one another, as required for a bifunctional catalytic mechanism in which a covalently linked enamine and iminium react to form the new C–C bond.

We computationally evaluated the conformational landscapes of derivatives of diamines **4** and **12** with acetaldehyde-derived enamine and iminium groups at the termini (designated **4**′ and **12**′). Ensembles of ∼1200 conformers representative of the conformational landscape for each species were generated using RDKit and optimized at the M06–2X/def2-TZVP/SMD = iPrOH level. Conformers with negative vibrational modes were excluded from the ensembles. Computational methods and geometric parameters for all conformers are provided in the Supporting Information.

The lowest free energy conformer identified for **4**′ had a fully extended eight-methylene tether with the maximum possible number (seven) of *anti* dihedral angles (Figure 5A). In this conformer, the catalyst-bound enamine and iminium groups are ∼14 Å from one another. Conformers of **4**′ with fewer *anti* dihedral angles were higher in energy. In contrast, the lowest free energy conformer identified for **12**′ had only two *anti* dihedral angles (Figure 5B). This conformer brought the terminal catalyst-bound enamine and iminium groups to within ∼4 Å of one another. The analysis suggests that there are many other conformations of **12**′ with multiple *gauche* torsions that bring the ends near one another, as required for bifunctional catalysis. Conformers of **12**′ with fully extended linkers (seven *anti* dihedral angles) were much higher in energy (>3 kcal/mol) relative to the conformation shown in Figure 5B. Calculated Boltzmann distributions depicting the populations of conformers of **4**′ and **12**′ as a function of C1–C13 distance or number of tether dihedrals in the *anti* configuration are supplied in the Supporting Information (Figure S73).

**Figure 5:**
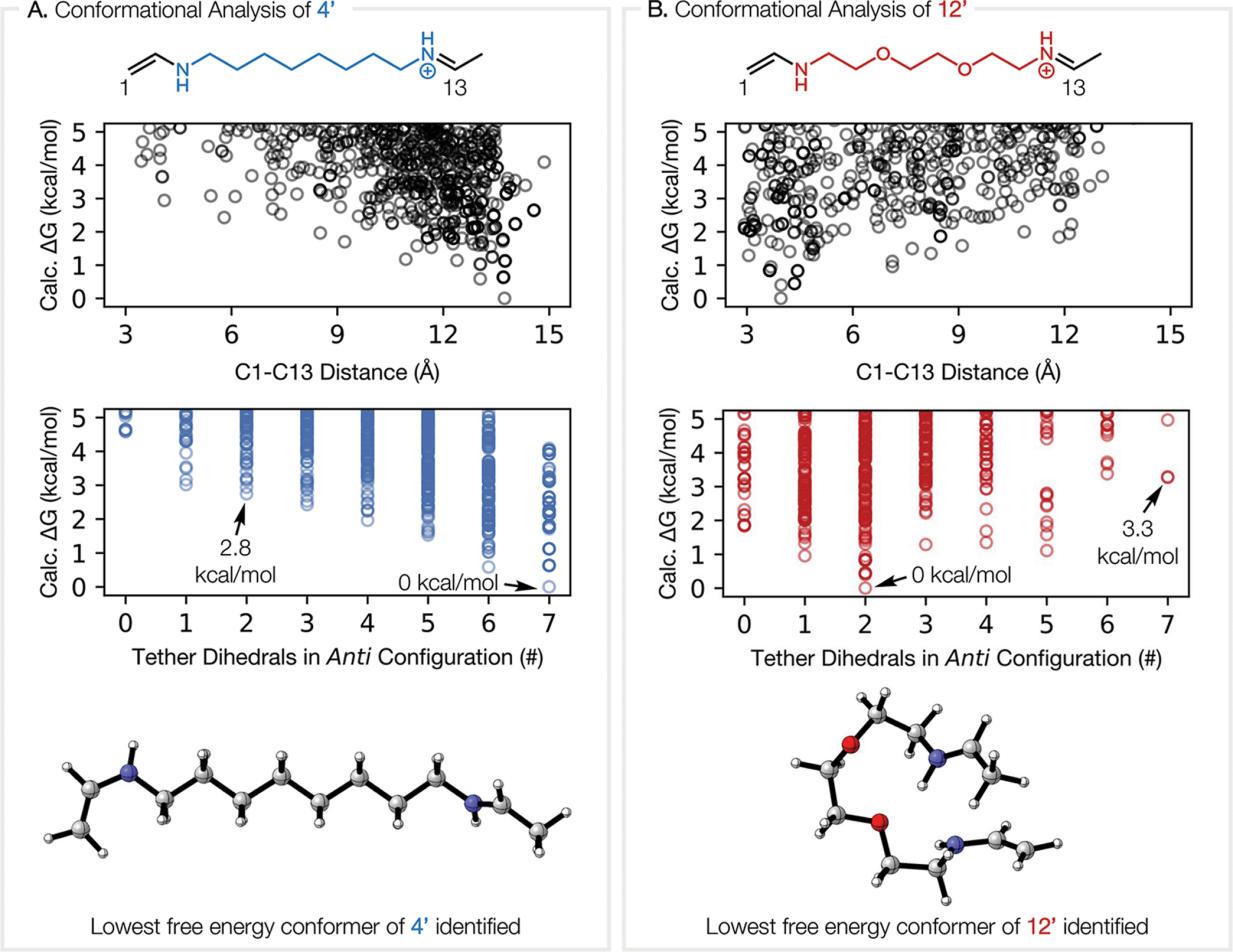
Computational analysis of conformer relative free energies as a function of C1–C13 distance and the number of anti dihedral angles in the tether for diamines (A) **4**′ and (B) **12**′. The lowest free energy conformer identified for each species is shown. Calculations were carried out at the M06–2X/def2-TZVP/SMD = iPrOH level of theory.

The data presented in Figure 5 suggest that oligoether diamine **12** exhibits a small energetic preference to bring terminal iminium and enamine groups into proximity as required for C–C bond formation. In contrast, oligomethylene-linked diamine **4** disfavors proximity of the terminal reactive groups. This conformational difference may explain why diamines with oligoether linkers (**10–15**) are more effective catalysts than analogues with oligomethylene linkers. More broadly, our findings suggest that oligoether linkers may be useful for generating simple flexible bifunctional catalysts that exhibit a propensity to bring together reactive units.

## Discussion

The studies described here were motivated by our recent finding that dihydrazides with oligomethylene linkers served as highly active catalysts of homoaldol condensations, providing higher yields at a given time point and substantially faster reactions relative to a monohydrazide.^17^ The most effective dihydrazides had relatively long linkers, with 10 methylenes proving to be optimal. In contrast, the shortest linker provided no benefit. Replacing the flexible oligomethylene linkers with a preorganized α/β-peptide scaffold led to a dihydrazide with modestly improved catalytic efficacy relative to the best flexible dihydrazide.^21^ We wondered whether these trends would be reproduced in a different catalytic system.

The results presented above involve a crossed aldol condensation and catalysts that contain two primary amine groups as the reactive diad. The crossed aldol reactions were conducted in isopropanol containing 4 vol % water, with amine and acid additives, while the earlier homoaldol reactions were conducted in dry acetonitrile with only an acid additive (amine additives suppressed homoaldol reactions in that system). Overall, the dihydrazide-catalyzed homoaldol reactions were considerably faster than the crossed aldol reactions described here. The absolute differences between a monofunctional catalyst and the best bifunctional catalysts were larger in the hydrazide-based system than in the primary amine-based system. However, despite these experimental differences, key trends in the two systems were comparable.

For the crossed aldol condensations discussed here, a short oligomethylene linker (**2**) did not provide any benefit relative to the monoamine reference catalyst (**1**). In contrast, linkers containing as many as 16 methylene units led to significant improvements in crossed aldol yield (Figure 2). A further yield improvement was achieved by replacing the flexible oligomethylene linker with a preorganized α/β-peptide linker. These trends were evident also in terms of ν_REL_, a parameter that depends on the specific conditions employed, as does yield. The diamine with a 14-methylene linker displayed a ∼3-fold improvement in ν_REL_, with *n*-butylamine (**1**) as the reference. α/β-Peptide diamine (**9**), on the other hand, displayed a ∼20-fold improvement in ν_REL_, as judged against **1**. However, α/β-peptide monoamines **16** and **17** were modestly more active than **1**; compared to the monoamine peptides, α/β-peptide diamine **9** displayed a 7- to 12-fold improvement in ν_REL_. This catalytic benefit of the preorganized α/β-peptide linker is comparable to the benefit observed in the dihydrazide series,^21^ where the best α/β-peptide displayed a ∼3.5-fold improvement in ν_REL_ when compared with a flexible dihydrazide containing a linker of similar length, but the α/β-peptide monohydrazides were ∼2-fold *less* active than a simple monohydrazide as judged by ν_REL_.

The earlier studies of hydrazide-based homoaldol catalysis relied on measurements of initial rate as a function of catalyst concentration to assess the order in catalyst.^17,21^ The results suggested that the homoaldol condensations were first-order in dihydrazide, which is consistent with a bifunctional catalysis mechanism. Here, we used the VTNA approach^29,30^ to evaluate reaction order for diamine catalysts. The results were consistent with first-order dependence on diamine concentration, supporting the conclusion that the diamines act as bifunctional catalysts. Consistent with our previous study of oligomethylene-linked dihydrazides,^17^ reductive intermediate trapping experiments yielded species with masses matching those of the reduced forms of intermediates within the proposed bifunctional reaction pathway.

We used the diamine system to evaluate oligoether linkers, which had no precedent in the dihydrazide studies. Judged by yield and/or ν_REL_ under fixed conditions, oligoether diamines with linkers of 8, 11, or 14 atoms were superior to oligomethylene diamines as crossed-aldol catalysts. This trend likely arises from conformational differences between segments composed exclusively of methylene units vs segments containing oxygen atoms interspersed among methylene units. Among the oligomethylene linkers, each torsional unit (CH_2_CH_2_—CH_2_CH_2_) prefers an *anti* conformation. In contrast, among the oligoether linkers, OCH_2_—CH_2_O torsional units prefer a *gauche* conformation. Our computational study suggests that many low-energy conformations of an oligoether linker bring the amine groups into proximity, while the lowest energy conformations of an oligomethylene linker hold those amines apart.

## Conclusions

The results presented here suggest that simple, flexible linkers that are relatively long (8–16 atoms in systems we have examined) can enable coordinated action of two reactive groups. A helix-forming α/β-peptide scaffold that is preorganized to align the reactive diad offers modest benefit relative to an oligomethylene linker, but an oligoether linker can match the folded peptide in terms of catalytic activity.

The benefits of oligoether linkers for bifunctional catalysis are attributed to the *gauche* preference of OCH_2_–CH_2_O torsion units, which enable a segment containing such units to fold back on itself with little or no torsional strain.^31,32^ The decreased steric profile of an oxygen atom relative to a methylene unit^39^ may also contribute to the advantage evident for oligoether linkers. Although we have observed the oligoether benefit in only one catalytic system, we note the related observation that radical-mediated macrolactone-forming reactions were superior for oligoether-containing substrates relative to oligomethylene-containing substrates.^40^ A number of studies have identified pairs of reactive units that facilitate valuable organic transformations.^41–45^ Linkage of such pairs with oligoethers could offer a facile approach for exploring the potential for bifunctional catalysis.

## Methods

### General Procedure: Crossed Aldol Condensation

To a 2 mL screw-top vial was added 60 μL of a stock solution containing hydrocinnamaldehyde (0.003 mmol, 1 equiv), 2,6-dimethoxybenzaldehyde (0.00375 mmol, 1.25 equiv), triethylamine (0.006 mmol, 2 equiv), propionic acid (0.03 mmol, 10 equiv) and diphenylacetonitrile (0.003 mmol, 1 equiv., internal standard) in 96:4 isopropanol:water (concentration of hydrocinnamaldehyde = 50 mM). The reaction solution was then diluted with 490 μL of 96:4 isopropanol:water (final concentration of hydrocinnamaldehyde was 5 mM), the vial was capped and set inside a 37 °C oven for 10 min. The vial was then charged with 50 μL of a stock solution of diamine (0.0003 mmol, 0.1 equiv) or monoamine (0.0006 mmol, 0.2 equiv) in 96:4 isopropanol:water. The vial was capped, shaken briefly and then left to sit, without stirring, for 24 h in a 37 °C oven.

### General Procedure: Determination of Product Yields and Initial Rates of CA Formation

**CA** and **HA** yields at 24 h were determined by UPLC analysis of aliquots of the reaction solution. Aliquots (0.2 μL injection volume) were analyzed using a Waters Acquity H-Class UPLC equipped with a Waters Acquity Premier BEH Phenyl (1.7 μm 2.1 × 150 mm^2^) column using a 3 min isocratic method with a mobile phase generated by mixing one volume of H_2_O + 0.1% trifluoroacetic acid with two volumes of acetonitrile + 0.1% trifluoroacetic acid. The flow rate was 0.3 mL per minute. The eluent was monitored based on absorption at 214 nm. Calibration curves (external standards) were used to calculate product yields based on integrated areas of peaks corresponding **CA**, **HA** and the internal standard (diphenylacetonitrile). Initial rates of **CA** formation were determined by analyzing reaction solution aliquots every hour for the first 5 h of reaction. Reaction profiles used in VTNA were obtained using the same method by analyzing aliquots taken at multiple reaction time points.

### Conformer Ensemble Generation and Optimization

Initial conformer geometries were generated using RDKit.^46^ Quantum mechanical calculations on conformer ensembles for **4**′ and **12**′ were conducted using Gaussian 16.^47^ Optimizations and vibrational frequency calculations were carried out at the B3LYP/6–31G­(d,p) and M06–2X/def2-TZVP levels using the SMD solvent model for isopropanol.^48–53^ Conformers exhibiting negative vibrational modes were excluded from the ensembles for **4**′ and **12**′. A tether dihedral was defined as being in the *anti* configuration when the angle measured for the dihedral was 180° ± 30°.^54^ Reported C1-to-C13 distances are through space.

## Supplementary Material



## Data Availability

The data underlying this study are available in the published article, in its Supporting Information, and openly available in GitHub at 10.5281/zenodo.20029283.

## Notes

Experimental information, computational methods, kinetics data, NMR spectra, and characterization data (PDF)
