# Supplementary material for: Bifunctional Catalysis of a Crossed Aldol Condensation by Diamines: Impact of Tether Composition and Length
Source: J Org Chem. 2026 Jun 5;91(24):8312–21. doi: 10.1021/acs.joc.6c00659 (PMC13288624; doi:10.1021/acs.joc.6c00659)
Supplement: Supplementary file 1 [file jo6c00659_si_001.pdf]

Bifunctional Catalysis of a Crossed Aldol Condensation by Diamines: Impact of Tether Composition and Length

Philip P. Lampkin, R. Charles Roberts, Bianca Czeslawski, and Samuel H. Gellman*

Department of Chemistry, University of Wisconsin–Madison,
1101 University Avenue, Madison, WI 53706, United States

Email: gellman@chem.wisc.edu

Supporting information

Table of Contents

1. **General Information** **S3**
2. **Synthetic Information** **S4**
    - **2.1** - Synthesis of 2-benzyl-3-(2,6-dimethoxyphenyl)acrylaldehyde (**CA**)
    - **2.2** - Synthesis of Foldamers **9**, **16** and **17**
3. **Experimental Information** **S6**
    - **3.1** - General Experimental Information
    - **3.2** - Aldol Condensation Reaction Procedure
    - **3.3** - UPLC Calibration Curves for **CA** and **HA**
    - **3.4** - Representative Crossed Aldol Reaction Chromatogram
    - **3.5** - Determination of Initial Rates of **CA** Formation
    - **3.6** - Other Experiments
        - **3.6.1** - Effect of acid loading on initial rate of **CA** formation catalyzed by **1** or **12**
        - **3.6.2** – Effect of base loading on initial rate of **CA** formation catalyzed by **12**
        - **3.6.3** - Effect of perchlorate salts on aldol reactions catalyzed by oligoether diamines
        - **3.6.4** - Reactivity of oligoether diamines with very long tethers
        - **3.6.5** - Determination of **CA** isomer configuration by NOESY NMR
        - **3.6.6** - Dependence of initial rate of **CA** formation on **1** and **12** loading
        - **3.6.7** - Relative rates of **CA** formation normalized to monoamine **10**
        - **3.6.8** - In-situ trapping of reaction intermediates
4. **Variable Time Normalized Analysis** **S32**
    - **4.1** - Determination of reaction order for **1** and **12**
    - **4.2** - Examination of potential inhibition of **1** by **CA**, **HA**, $\mathbf{A_N}$ or $\mathbf{A_E}$
    - **4.3** - Potential mechanisms for reactions catalyzed by **1**
5. **Initial Rates and Yields** **S45**
    - **5.1** - Yields Summary for 24 h Reactions
    - **5.2** - Initial and Relative Rates Summary
    - **5.3** - Initial Rates for **1**, **4**, **7**, **9**, **10**, **12**, **14**, **15**, **16** and **17**
6. **Computational Information** **S82**
    - **6.1** - General Computational Information
    - **6.2** - Conformer Ensemble Generation and Optimization Procedure
    - **6.3** - Boltzmann distributions of conformers **4’** and **12’** – M06-2X/def2-TZVP
    - **6.4** - DFT-derived Geometric Parameters for **4’** – B3LYP/6-31G(d,p)
    - **6.5** - DFT-derived Geometric Parameters for **4’** – M06-2X/def2-TZVP
    - **6.6** - DFT-derived Geometric Parameters for **12’** – B3LYP/6-31G(d,p)
    - **6.7** - DFT-derived Geometric Parameters for **12’** – M06-2X/def2-TZVP
7. **NMR, MS and UPLC Data** **S148**
8. **References** **S153**
9. **Instrumentation Funding Acknowledgements** **S155**

1. General Information

All substrates, diamine catalysts and amino acid materials were purchased from Ambeed, Chem-Impex International or Sigma-Aldrich and used as received. Solvents used were HPLC-grade. Reactions were carried out on the benchtop without any effort to exclude air or moisture. Materials were stored on the benchtop unless otherwise stated. High-performance liquid chromatography (HPLC) was carried out using an Agilent 1260 Infinity II preparative LC UV-MSD system equipped with a 1290 Infinity II Preparative Open-Bed Sampler/Collector module and preparative Waters XSelect CSH C18 OBD column (130 Å, 5 µm, 19 mm X 250 mm). Analytical ultra performance liquid chromatography (UPLC) for peptide purity and reaction outcome analysis were carried out using a Waters Acquity H-Class UPLC equipped with an Waters UPLC Phenyl BEH column (300 Å, 1.7 µm, 2.1 mm X 150 mm) or Waters UPLC C18 CSH column (300 Å, 1.7 µm, 2.1 mm X 150 mm), respectively. Kinetics experiments were conducted with the UPLC sample manager module maintained at 37 °C. Peptide synthesis was carried out using a CEM Mars II microwave reactor.

NMR spectra were obtained using a Bruker NEO 500 MHz NMR spectrometer. Chemical shifts (δ) are reported in parts per million (ppm). All ^{1}H and ^{13}C NMR spectra are internally referenced to tetramethylsilane (δ(^{1}H) = 0.00 ppm, δ(^{13}C) = 0.00 ppm). Data for ^{1}H and ^{13}C NMR signal multiplicity are reported as s (singlet), d (doublet), t (triplet), q (quartet) or m (multiplet), and coupling constants (*J*) are reported in Hz. High resolution mass spectra were obtained using a Bruker Impact II (ESI-Q-TOF-MS).

2. Synthetic Information

All simple amines and diamines (catalysts **1**-**20**) were purchased from Ambeed or Sigma Aldrich and used as received. HR-ESI-MS was used to confirm diamine mass prior to use. The homoaldol product (**HA**) was prepared according to a published procedure, and analytical data for **HA** agreed with literature values.[1,2]

2.1 Synthesis of 2-benzyl-3-(2,6-dimethoxyphenyl)acrylaldehyde (CA)

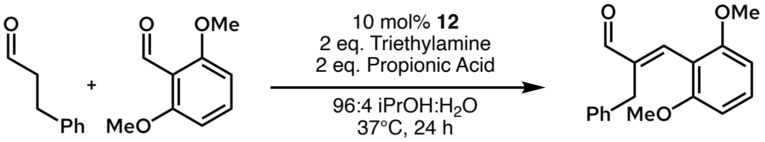

(0.5 M) 1.25 equiv. (*E*)-CA

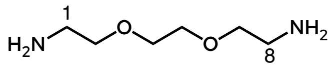

12

To a 25 mL round bottom flask were added 2,6-dimethoxybenzaldehyde (210 mg, 1.27 mmol, 1.25 equiv.), hydrocinnamaldehyde (1.01 mmol, 0.135 mL, 1 equiv.), triethylamine (2.01 mmol, 0.27 mL, 2 equiv.), propionic acid (2.01 mmol, 0.15 mL, 2 equiv.), 2 mL of 96:4 isopropanol:H_2O containing 0.5 M hydrocinnamaldehyde, and a stir bar. The reaction solution was heated to 37°C in an oil bath, after which diamine **12** (0.10 mmol, 0.013 mL, 0.1 equiv.) was added. The flask was capped, and the mixture left to stir at 37°C for 24 hours. The reaction solution was then diluted with 50 mL of ethyl acetate and washed with water (2 x 25 mL) and brine (1 x 25 mL). The organic layer was isolated, dried with sodium sulfate, and concentrated *in vacuo*. The crude product was dissolved in 1 mL of 1:1 H_2O/ACN and passed through a 22-micron PTFE syringe filter into a 2.5 mL HPLC vial. This process was repeated 4 times to rinse the remaining crude material from the flask into HPLC vials. The solutions in each HPLC vial were mixed to equalize the concentration of crude product across all vials. Crude product was purified on an HPLC equipped with a Waters XSelect CSH C18 OBD column (130 Å, 5 µm, 19 mm X 250 mm) using a gradient of 60:40 Water:MeCN (0.1%TFA) to 10:90 Water:MeCN (0.1%TFA) over 14 minutes with a flow rate of 18 mL/min. Fractions containing purified product were combined and concentrated under a stream of N_2 to remove MeCN. The concentrated solution was frozen and lyophilized, yielding a white powder of pure **(*E*)-CA** (16.6 mg, 6.6% yield).

2-Benzyl-3-(2,6-dimethoxyphenyl)acrylaldehyde (CA): ^{1}H NMR (600 MHz, $CDCl_3$): δ = 9.71 (s, 1H), 7.41 (s, 1H), 7.30 (t, 1H), 7.16 (t, 2H), 7.09 (t, 1H), 7.02 (d, 2H), 6.58 (d, 2H), 3.75 (s, 6H), 3.61 (s, 2H) ppm; ^{13}C{^{1}H} NMR (151 MHz, $CDCl_3$): δ = 194.9, 157.4, 144.7, 144.1, 139.3, 130.6, 128.4, 127.8, 125.5, 112.6, 103.5, 55.5, 32.0 ppm; HR-ESI-MS: *m/z:* 283.1324, 305.1142 ([*M*+H]$^+$ calculated for $C_{18}H_{19}O_3^+$: 283.1329, [*M*+Na]$^+$ calculated for $C_{18}H_{18}O_3Na^+$: 305.1149).

Note on determination of CA alkene configuration: The configuration of the alkene in samples of **CA** was confirmed by ^{1}H^{1}H NOESY NMR. For full experimental details, see section 3.6.5.

2.2 Synthesis of Foldamers **9**, **16** and **17**

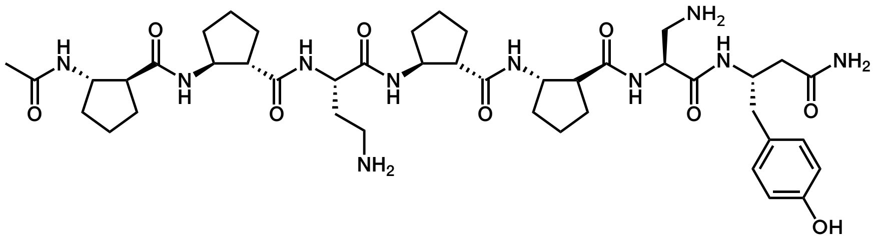

Acetyl-ACPC-ACPC-Dab-ACPC-ACPC-Dap-B^{3}HTyr-C(O)NHMe (9)

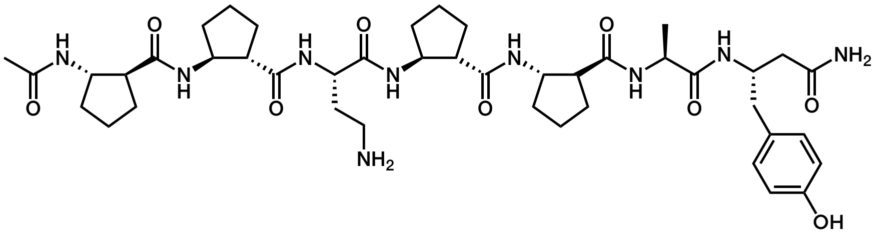

Acetyl-ACPC-ACPC-Dab-ACPC-ACPC-Ala-B^{3}HTyr-C(O)NHMe (16)

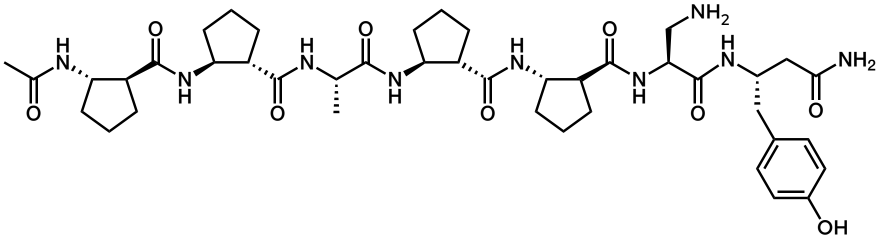

Acetyl-ACPC-ACPC-Ala-ACPC-ACPC-Dap-B^{3}HTyr-C(O)NHMe (17)

Dab-Dap (**9**), Dab-Ala (**16**) and Ala-Dap (**17**) foldamers were synthesized according to a literature procedure,[1,2] yielding a white powder. Analytical data for all peptides agreed with literature values.[1] All peptides were >95% pure by UPLC, and expected masses for peptides were observed by HR-ESI-MS.

Notes on peptide catalyst stock preparation: stock solutions were prepared by dissolving pure peptides in 96:4 HPLC-grade iPrOH:nanopure H_2O and transferring the resulting solution to pre-weighed vials. Peptide solutions were then concentrated to dryness under a gentle stream of N_2, and vials were placed under a high vacuum for 48 h to remove trace H_2O. Vials containing dried, purified peptide were weighed to determine the final isolated mass of the peptide for calculation of peptide stock concentration. Peptides isolated by HPLC are TFA salts. Foldamer catalyst stock solution calculations account for the TFA counterion.

3. Experimental Information

3.1 General Experimental Information

Catalyst, reactant and reagent stocks were prepared using 96:4 iPrOH:H_2O. All stocks were used within 1 week of preparation. Hydrocinnamaldehyde was distilled immediately prior to use. All kinetics and yield determination experiments were conducted at least twice. Diphenylacetonitrile (**DiPhMeCN**) was used as an internal standard in all reactions. Yields reported for **HA** take into account that the maximum theoretical yield of **HA** corresponds to half of the starting amount of hydrocinnamaldehyde (**A_N**).

3.2 Aldol Condensation Reaction Procedure

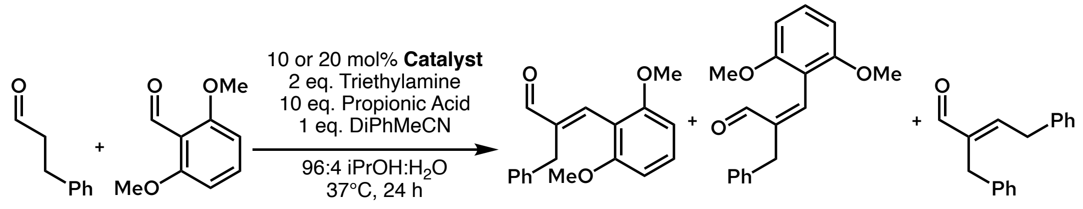

A_N (5 mM) 1.25 equiv. **A_E** ***(E)*-CA** ***(Z)*-CA** **HA**

To a 2 mL glass LC vial was added 60 µL of a stock solution containing hydrocinnamaldehyde (**A_N**, 0.003 mmol added to reaction, 50 mM in stock), 2,6-diemthoxybenzaldehyde (**A_E**, 0.00375 mmol added, 1.25 equiv. relative to **A_N**, 62.5 mM in stock), triethylamine (0.006 mmol added to reaction, 2.0 equiv. relative to **A_N**, 100 mM in stock), propionic acid (0.03 mmol added to reaction, 10 equiv. relative to **A_N**, 500 mM in stock) and **DiPhMeCN** (internal standard; 0.003 mmol added to reaction, 1.25 equiv. relative to **A_N**, 50 mM in stock) in 96:4 iPrOH:H_2O. The reaction solution was diluted with 490 µL of a 96:4 mixture of iPrOH:H_2O containing **A_N** (5 mM final **A_N** concentration), and the vial was sealed with a pierceable cap and set inside a 37°C oven for 10 minutes. To the 37 °C solution was then added 50 µL of a stock solution of diamine (0.0003 mmol added to reaction, 10 mol% relative to **A_N**, 6 mM in stock) or monoamine catalyst (0.0006 mmol added to reaction, 20 mol% relative to **A_N**, 12 mM in stock). The vial was briefly shaken and left to sit, without stirring, for 24 h in the 37°C oven. After 24 h, an aliquot of the reaction mixture (0.2 µL injection volume) was analyzed using a Waters Acquity H-Class UPLC equipped with an analytical Waters Acquity Premier BEH Phenyl 1.7 µm 2.1 x 150 mm column. A 5.5 minute isocratic elution with a 33:67 A:B mobile phase mixture (A = H_2O with 0.1% TFA and B = MeCN with 0.1% TFA) and 0.4 mL/min flow rate was used. UV-Vis Absorbances at 214 nm of products ***(E)*-CA**, ***(Z)*-CA** and **HA** as well as the **DiPhMeCN** internal standard were used with calibration curves to quantify reaction yields.

3.3 UPLC Calibration Curves for **CA** and **HA**

Calibration curves for **CA** and **HA** were constructed using a Waters Acquity H-Class UPLC equipped with an analytical Waters Acquity Premier BEH Phenyl 1.7 µm 2.1 x 150 mm column. Samples containing increasing amounts of the products (relative to **DiPhMeCN**) and a constant amount of internal standard (0.003 mmol, 5 mM **DiPhMeCN**) in 96:4 iPrOH:H_2O were prepared. These samples were analyzed by UPLC (0.2 µL injection volume) using a 5.5 minute isocratic elution with a 33:67 A:B mobile phase mixture (A = H_2O with 0.1% TFA and B = MeCN with 0.1% TFA) and 0.4 mL/min flow rate. The ratios of the area under the UV-Vis absorbance peak for each product and the **DiPhMeCN** internal standard measured on the 214 nm channel were used for yield quantification. The calibration curve for **HA** accounts for the maximum **HA** yield being half that of the starting amount of $\mathbf{A_N}$ (e.g., an input of 1 mmol $\mathbf{A_N}$ can produce a maximum of 0.5 mmol **HA**).

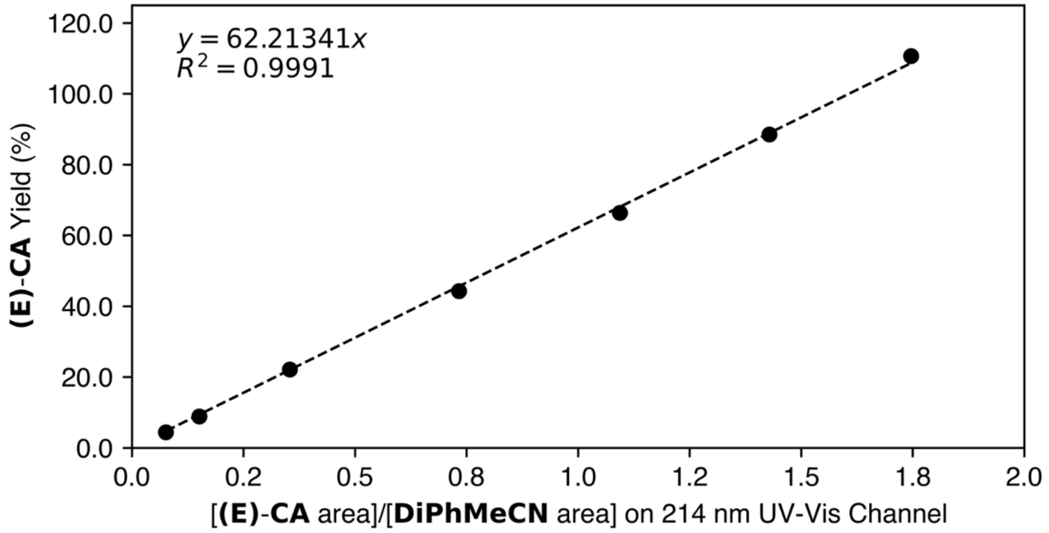

Figure S1. Calibration curve for **(*E*)-CA** using **DiPhMeCN** as an internal standard.

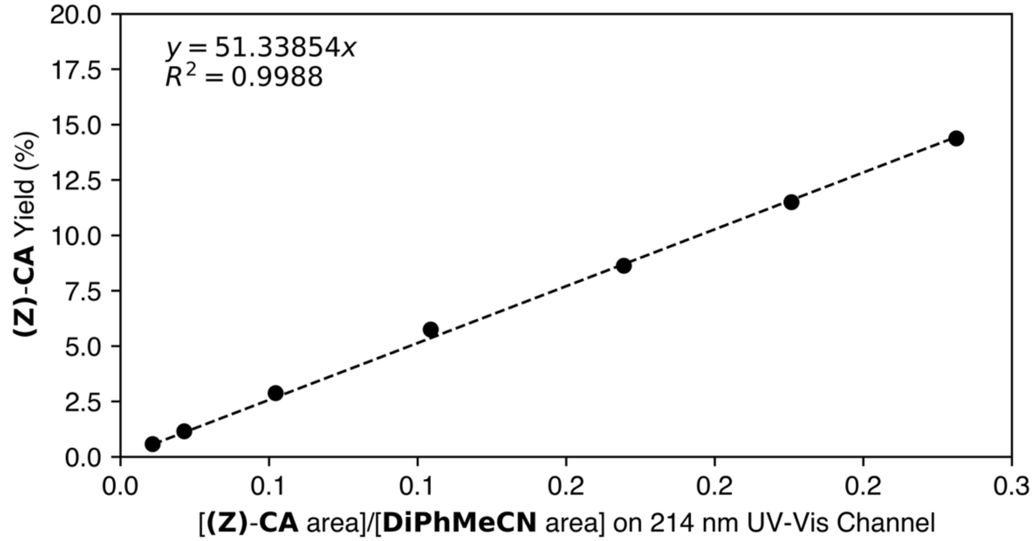

Figure S2. Calibration curve for **(*Z*)-CA** using **DiPhMeCN** as an internal standard.

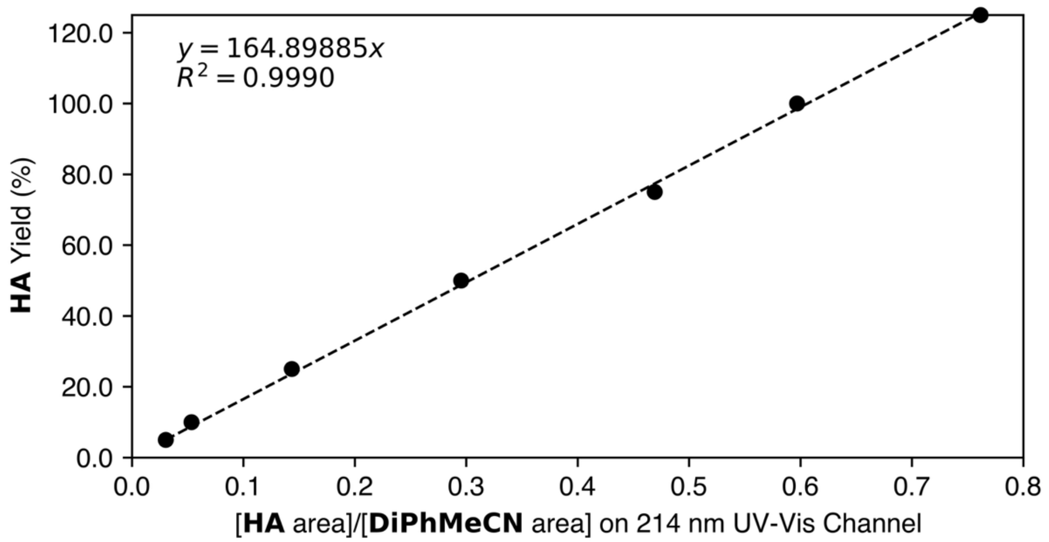

Figure S3. Calibration curve for **HA** using **DiPhMeCN** as an internal standard.

3.4 Representative Crossed Aldol Reaction Chromatogram

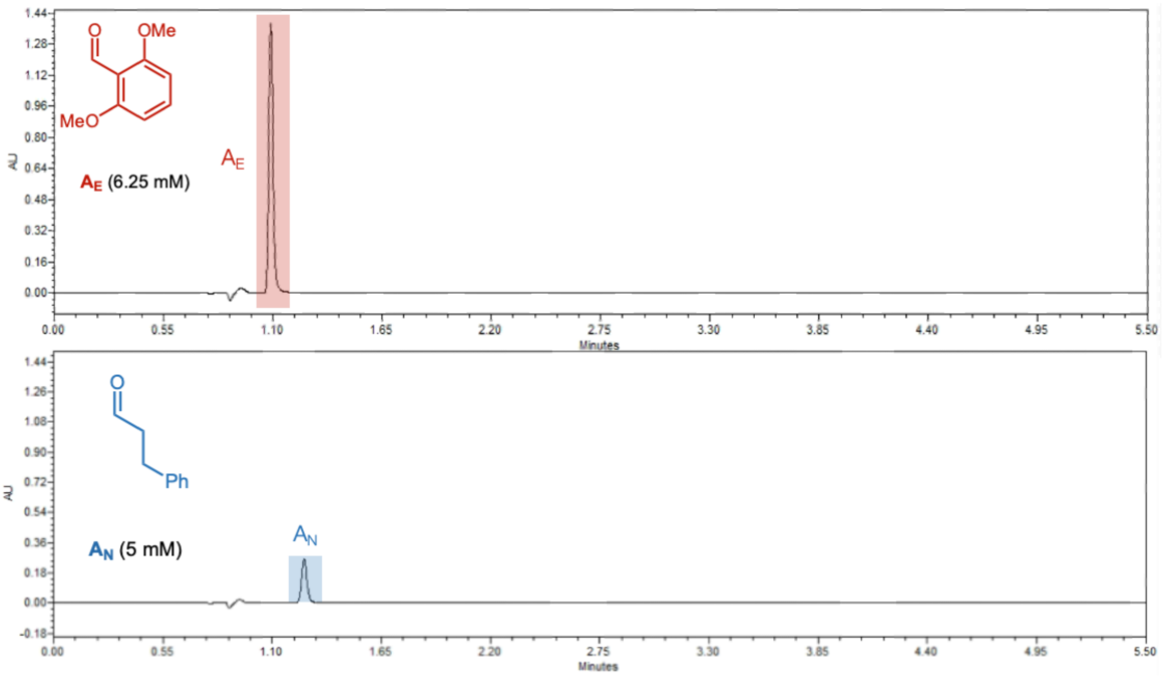

Figure S4. UPLC chromatograms (214 nm channel) of pure A_E and A_N at reaction concentrations.

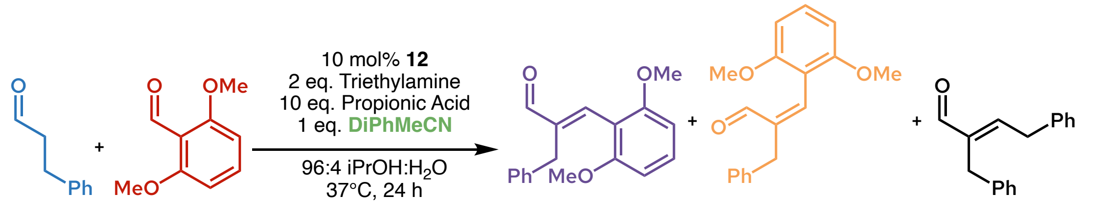

A$_N$ (5 mM) 1.25 equiv. **A**$_E$ **(*E*)-CA** **(*Z*)-CA** **HA**

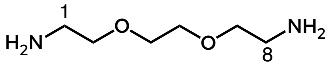

12

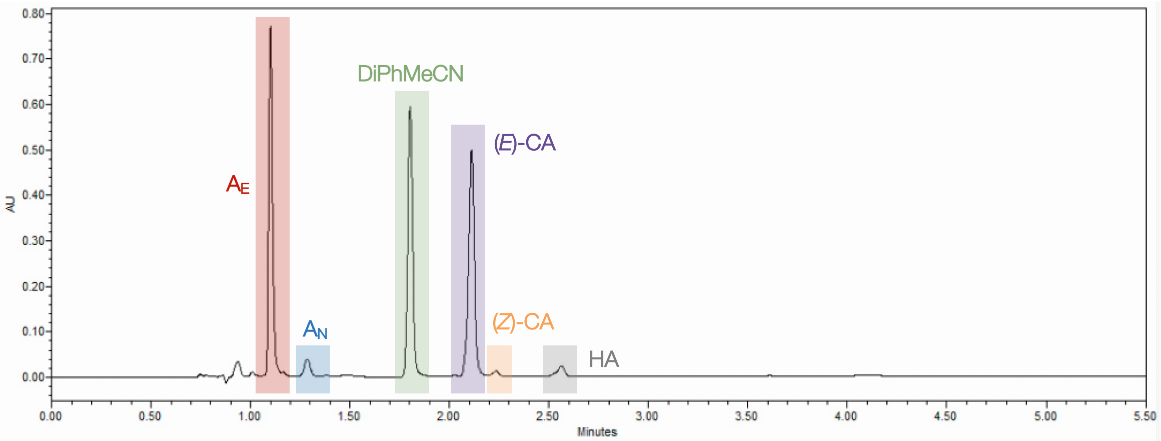

Figure S5. Representative UPLC chromatogram (214 nm channel) of the crossed aldol condensation reaction catalyzed by **12** after 24 h. Unreacted starting materials **A**$_E$**, A**$_N$, products **(*E*)-CA**, **(*Z*)-CA**, **HA**, and the internal standard **DiPhMeCN** are labeled.

3.5 Determination of Initial Rates of **CA** Formation

Initial rates of **CA** formation for catalysts **1**, **4**, **7**, **9**, **16**, **17**, **12**, and **14** were determined using a modification of the aldol condensation reaction procedure detailed in section 3.2 of this document. Aliquots of the reaction mixture were analyzed by UPLC according to the procedure each hour for the first 5 h of the reaction.

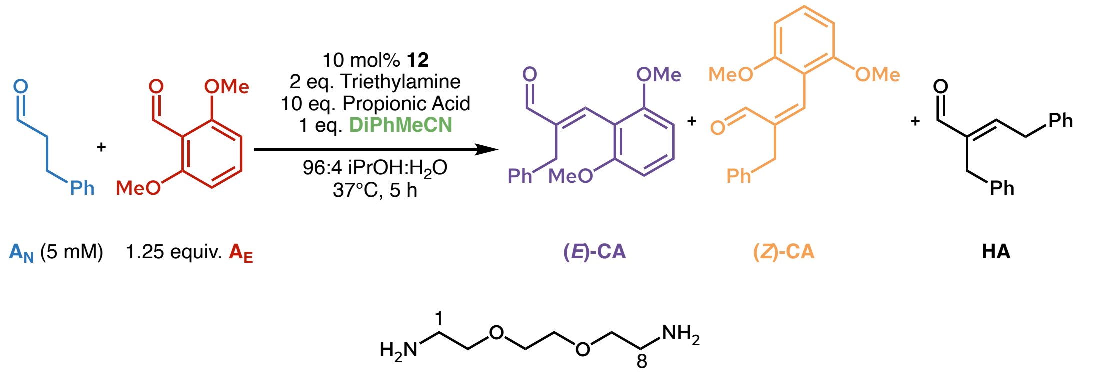

12

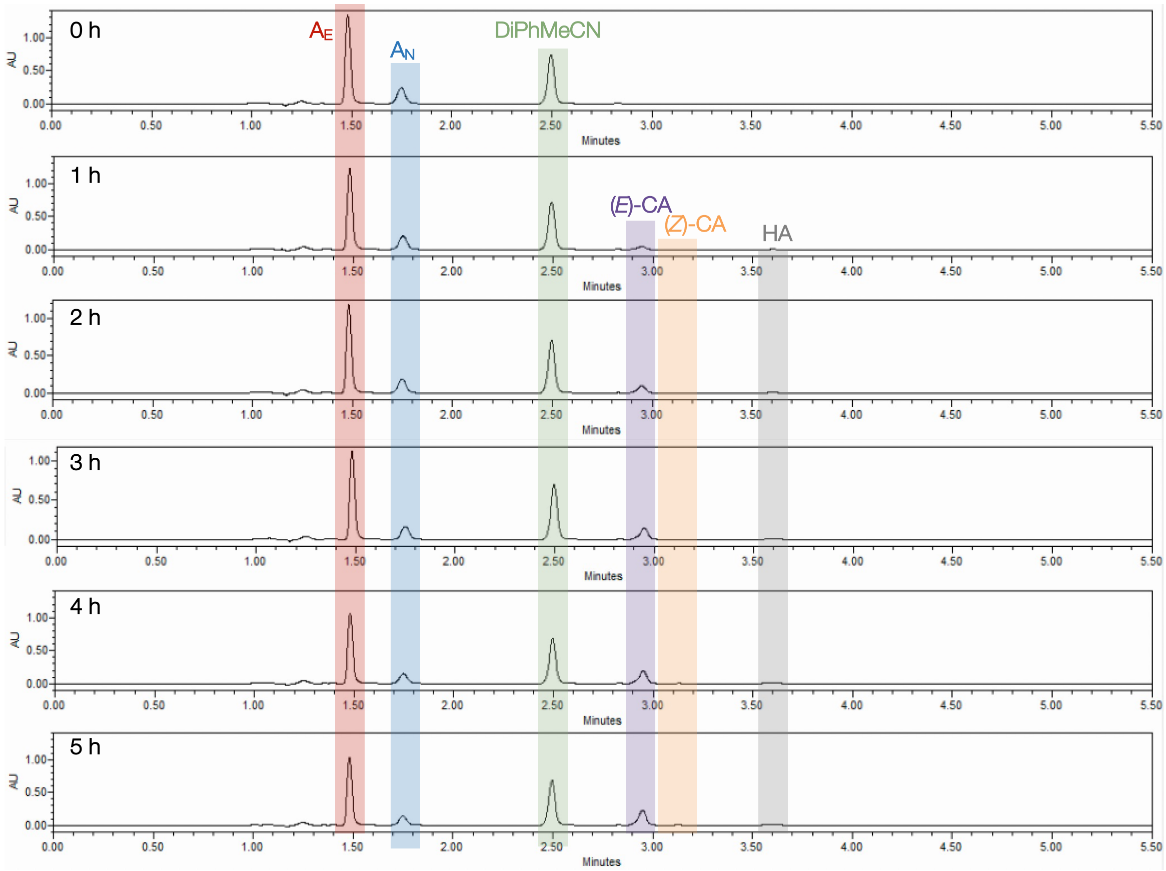

Figure S6. Stacked representative UPLC chromatogram (214 nm channel) of the crossed aldol condensation reaction catalyzed by **12** over 5 h of reaction. Unreacted starting materials **A_E, A_N**, products **(*E*)-CA**, **(*Z*)-CA**, **HA**, and the internal standard **DiPhMeCN** are labeled.

3.6 Other Experiments

3.6.1 Effect of acid loading on initial rate of **CA** formation catalyzed by **1** or **12**

The effect of acid loading on initial rates of **CA** formation (v_{INT}) catalyzed by **1** or **12** was determined by varying the amount of propionic acid in **1**- and **12**-catalyzed reactions. Reactions were carried out according to the procedure detailed in section 3.2 of this document. A large excess of acid (10 eq.) was found to significantly accelerate the **12**-catalyzed reaction but did not significantly affect the **1**-catalyzed reaction (Figure S7).

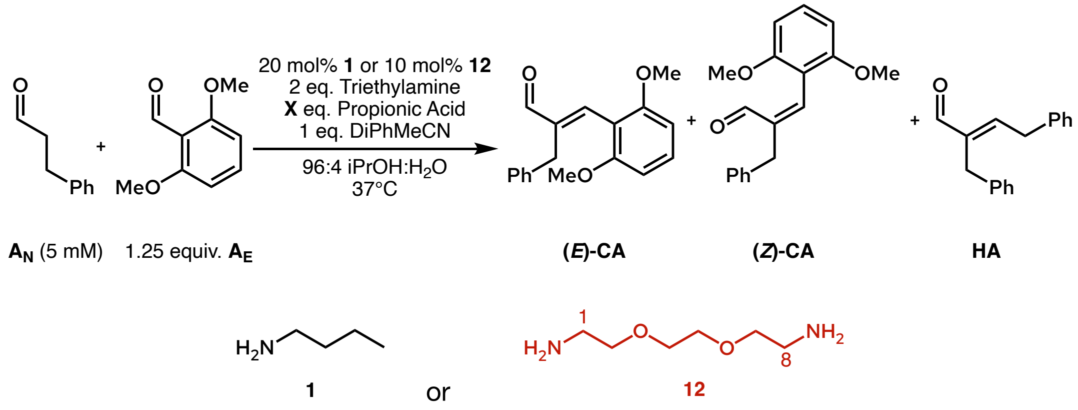

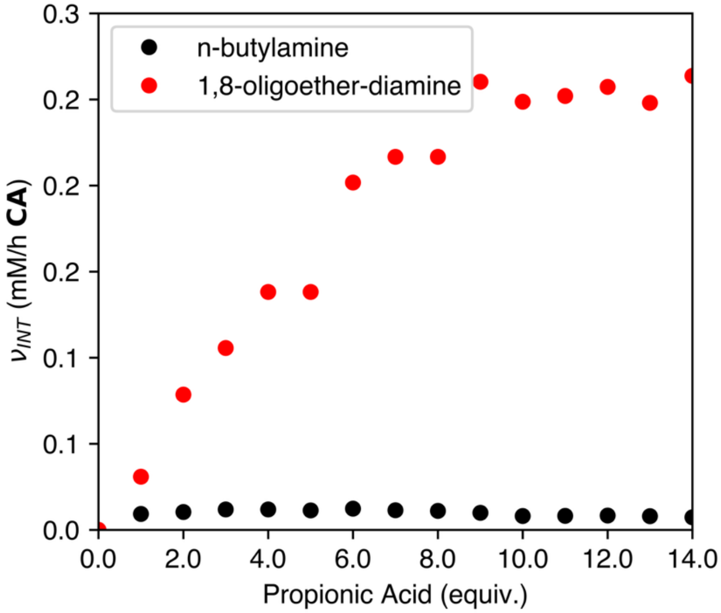

Figure S7. Effect of propionic acid loading on the initial rate of **CA** formation (v_{INT}) catalyzed by *n*-butylamine (**1**) or 1,8-oligoether-diamine (**12**).

3.6.2 Effect of base loading on initial rate of **CA** formation catalyzed by **12**

The effect of base loading on initial rates of **CA** formation (v_{INT}) catalyzed by **12** was determined by varying the amount of triethylamine acid and propionic acid in **12**-catalyzed reactions with 3 different loadings of TEA (0, 2 and 4 equivalents). Reactions were carried out according to the procedure detailed in section 3.2 of this document. Triethylamine loadings of 2 eq. provided the greatest v_{INT} values at high propionic acid loadings (Figure S8).

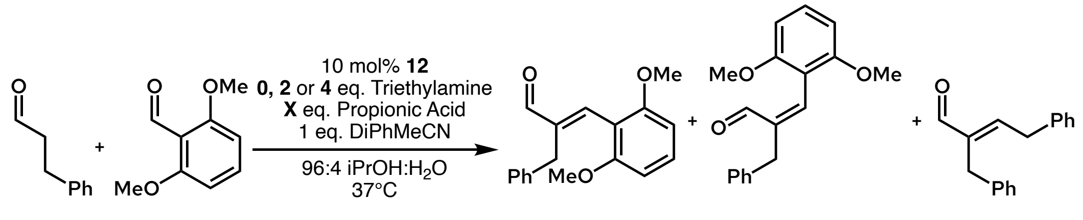

A_N (5 mM) 1.25 equiv. A_E **(*E*)-CA** **(*Z*)-CA** **HA**

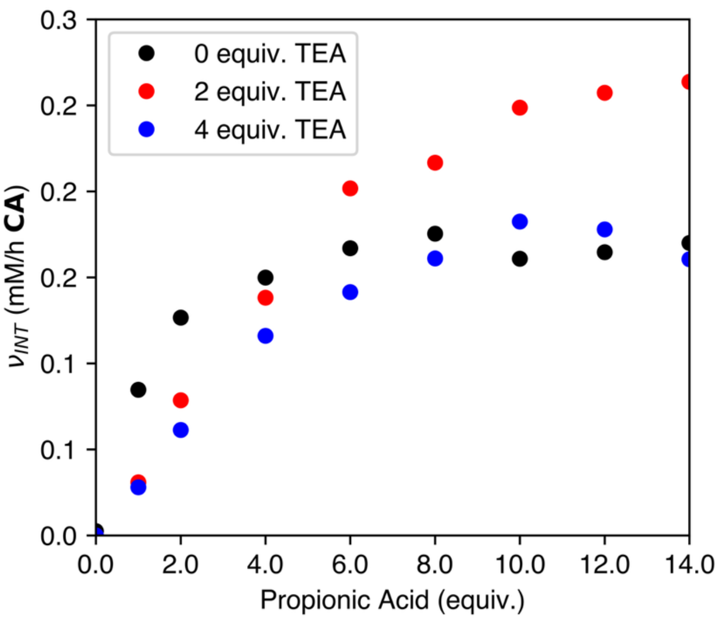

Figure S8. Effect of TEA loading across multiple acid loadings on the initial rate of **CA** formation (v_{INT}) catalyzed by 1,8-oligoether-diamine (**12**).

3.6.3 Effect of perchlorate salts on aldol reactions catalyzed by oligoether diamines

We hypothesized that addition of Li^+, Na^+ or K^+ ions to the reaction mixture might enhance the activity of oligoether diamines by facilitating robust preorganization of the amine reactive groups through coordination of the O-atoms in the oligoether linker with the Li^+, Na^+ or K^+ ions (Figure S9). We examined this hypothesis by adding $XClO_4$ (X = Li^+, Na^+ or K^+) to reactions catalyzed by oligoether diamines 14- or 20-atoms in length. Reactions were carried out according to the procedure detailed in section 3.2 of this document. No significant changes in initial rates of **CA** formation (v_{INT}) were observed for reactions with $XClO_4$ relative to reactions without $XClO_4$ (Table S1 and Table S2). These observations suggest that ion chelation does not preorganize the oligoether diamines.

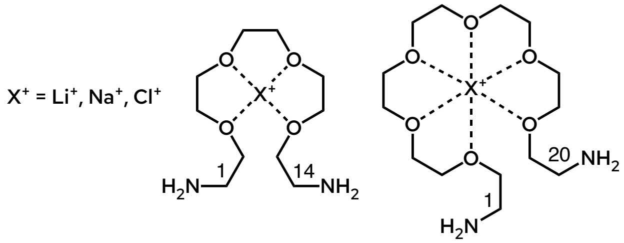

Figure S9. Potential chelation-induced preorganization of oligoether diamines.

Table S1. Initial (v_{INT}) and relative (v_{REL}) rates of **CA** formation for catalyst **14** in the presence of 0, 0.1 or 1.0 equivalents of various perchlorate salts

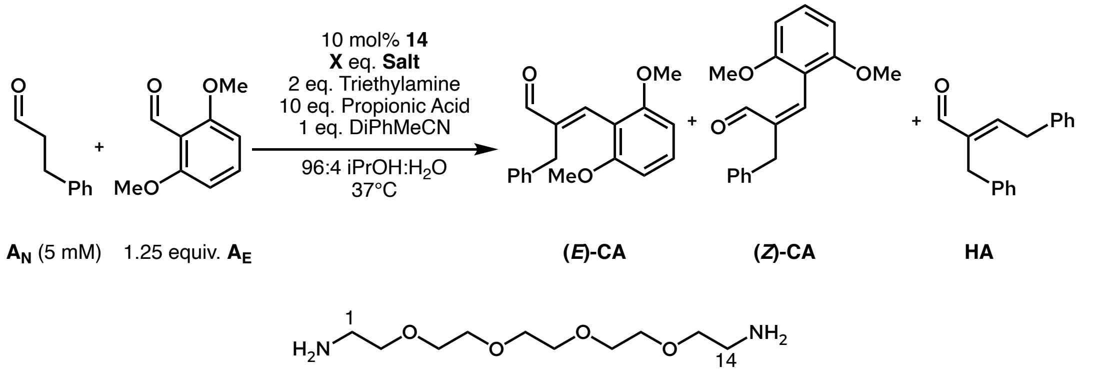

#	Catalyst	Salt	X (Equiv.)	v_{INT} (mM/h CA)	v_{REL}
**14**	1,14-oligoether-diamine	None	0	0.24527	1.0
"	"	$LiClO_4$	0.1	0.21703	0.9
"	"	$LiClO_4$	1.0	0.22367	0.9
"	"	$NaClO_4$	0.1	0.22580	0.9
"	"	$NaClO_4$	1.0	0.23274	0.9
"	"	$KClO_4$	0.1	0.24229	1.0
"	"	$KClO_4$	1.0	0.25469	1.0

Table S2. Initial (v_{INT}) and relative (v_{REL}) rates of **CA** formation for catalyst **15** in the presence of 0, 0.1 or 1.0 equivalents of various perchlorate salts

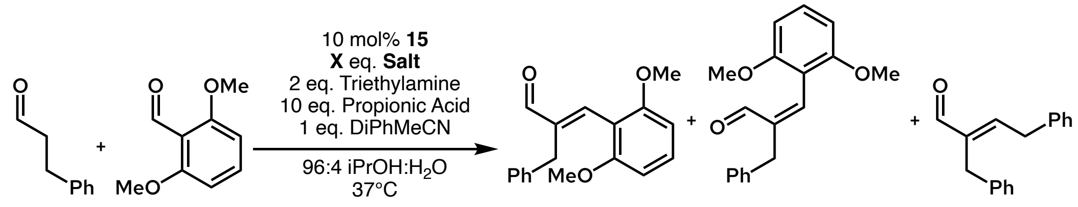

$\mathbf{A_N}$ (5 mM) 1.25 equiv. $\mathbf{A_E}$ **(*E*)-CA** **(*Z*)-CA** **HA**

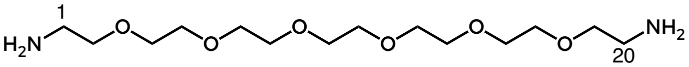

15

#	Catalyst	Salt	X (Equiv.)	v_{INT} (mM/h CA)	v_{REL}
**15**	1,20-oligoether-diamine	None	0	0.11949	1.0
"	"	$LiClO_4$	0.1	0.12625	1.1
"	"	$LiClO_4$	1.0	0.09388	0.8
"	"	$NaClO_4$	0.1	0.11531	1.0
"	"	$NaClO_4$	1.0	0.09094	0.8
"	"	$KClO_4$	0.1	0.11959	1.0
"	"	$KClO_4$	1.0	0.12391	1.0

3.6.4 Reactivity of oligoether diamines with very long tethers

Oligoether diamines with tethers containing 23, 29 or 35 atoms provided slightly lower yields after 24 h relative to the 1,20-oligoether-diamine (**15**) catalyst (Table S3). Reactions were carried out according to the procedure detailed in section 3.2 of this document. The enhanced activity displayed by these very long diamines relative to *n*-butylamine (**1**; 7% yield of **CA**) suggests that even highly extended tethers can provide a benefit to reactivity.

Table S3. 24 h **(*E*)-CA**, **(*Z*)-CA** and **HA** yields for catalysts **18** to **20**

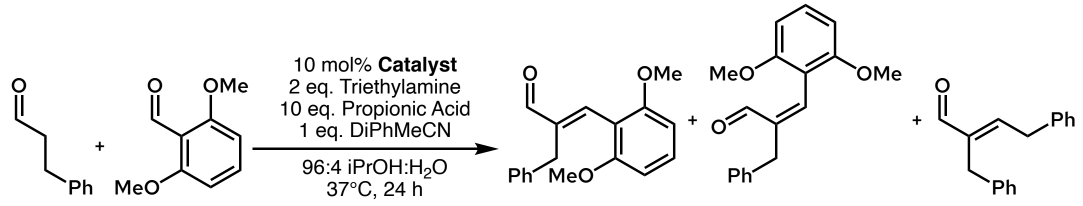

$\mathbf{A_N}$ (5 mM) 1.25 equiv. $\mathbf{A_E}$ **(*E*)-CA** **(*Z*)-CA** **HA**

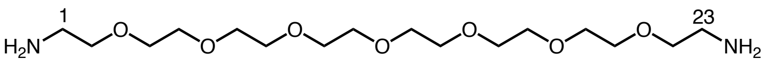

18

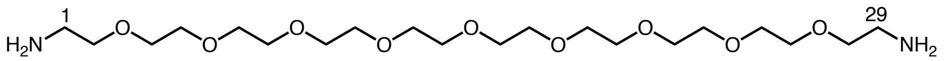

19

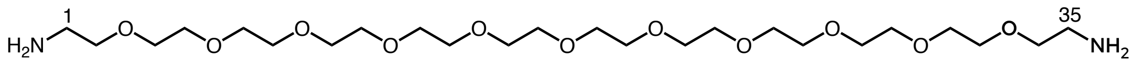

20

#	Catalyst	(*E*)-CA Yield (%)	(*Z*)-CA Yield (%)	HA Yield (%)
**18**	1,23-oligoether-diamine	36	6	3
**19**	1,29-oligoether-diamine	33	5	3
**20**	1,35-oligoether-diamine	33	4	3

3.6.5 Determination of **CA** isomer configuration by NOESY NMR

The configuration of **(*E*)-** and **(*Z*)-CA** were established by 1D $^{1}H^{1}H$ NOESY NMR. A through-space interaction between the aldehyde ^{1}H and alkene ^{1}H of **(*E*)-CA** was observed. No interaction was observed for **(*Z*)-CA**. The configuration of **HA** was determined in a previous publication using the same method.[1]

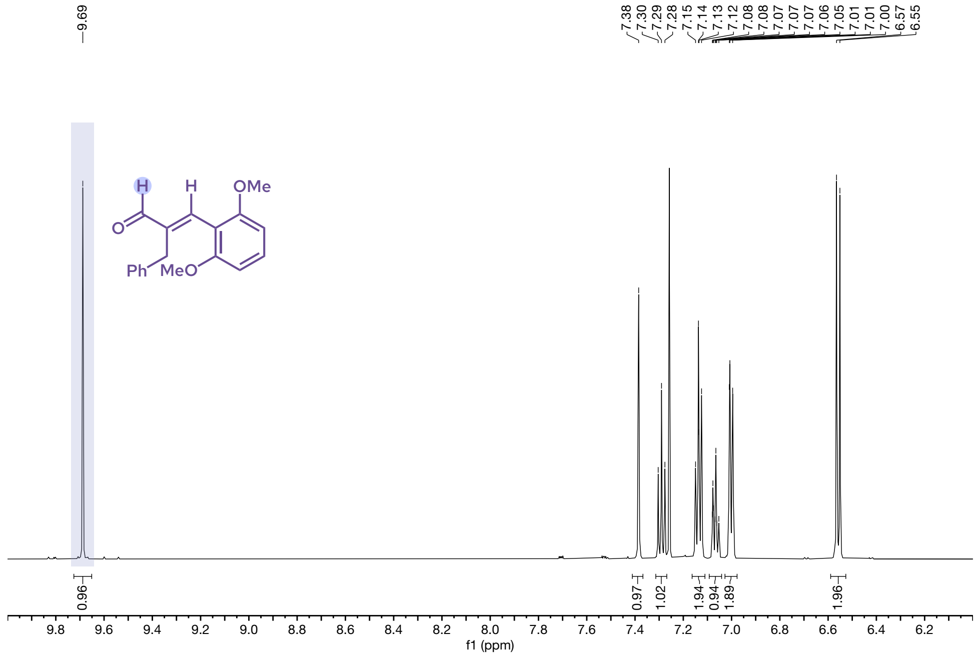

Figure S10. ^{1}H NMR of pure **(*E*)-CA**.

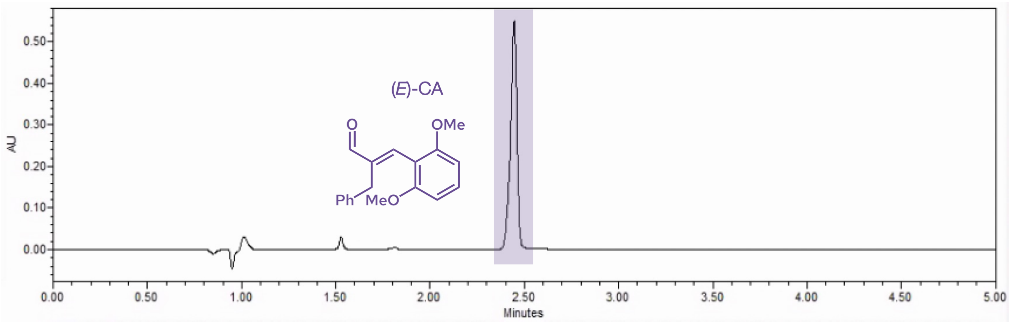

Figure S11. UPLC chromatogram (214 nm channel) of pure **(*E*)-CA**.

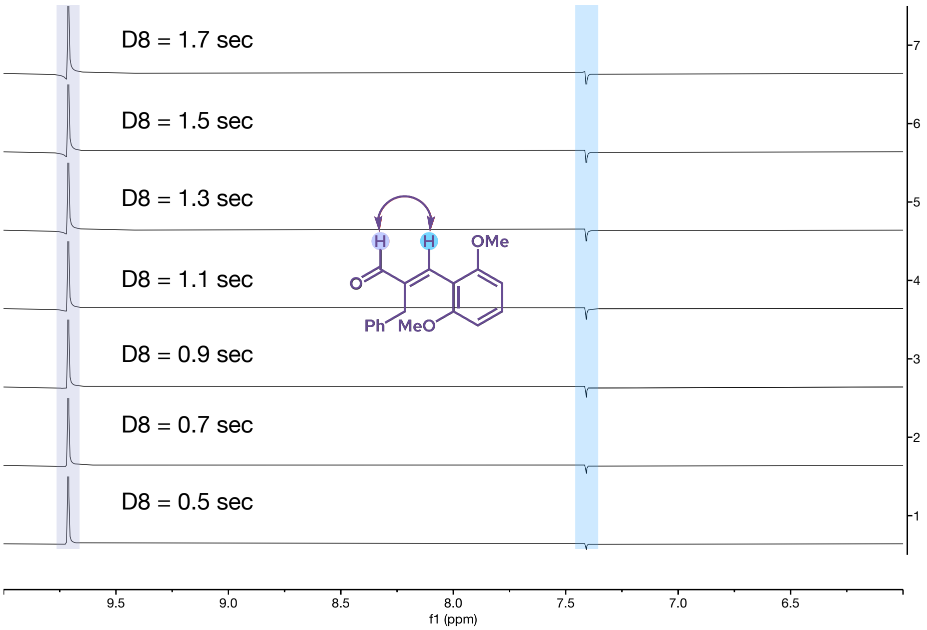

Figure S12. 1D $^{1}H^{1}H$ NOESY NMR of pure **(*E*)-CA**. The resonance corresponding to the aldehyde ^{1}H (9.62 ppm, purple) was excited. An interaction with the alkene ^{1}H (7.41 ppm, blue) was observed.

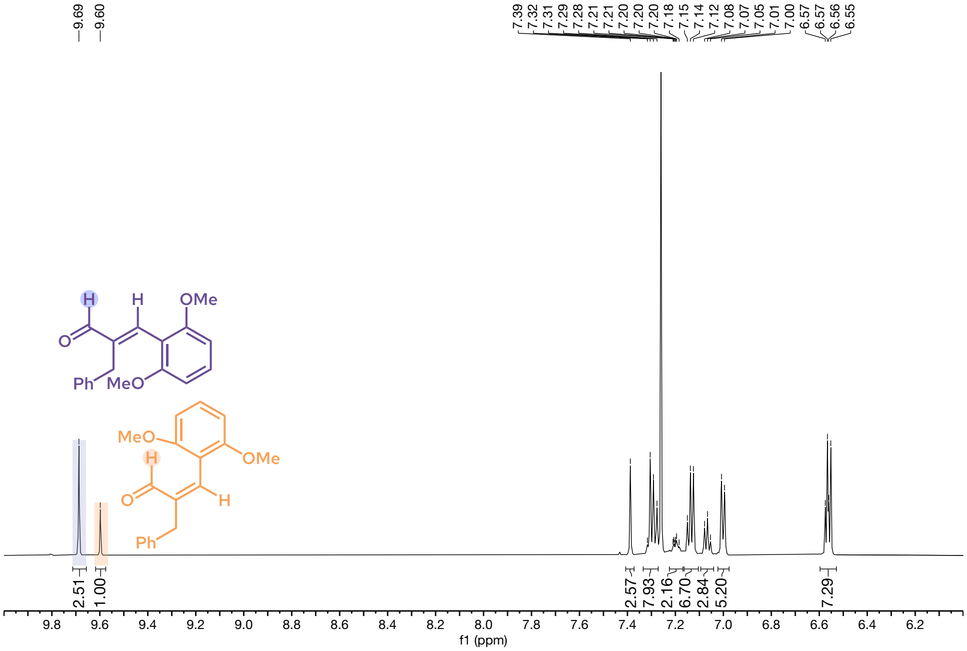

Figure S13. ^{1}H NMR of ~2.5:1 mixture of **(*E*)-CA**:**(Z)-CA.**

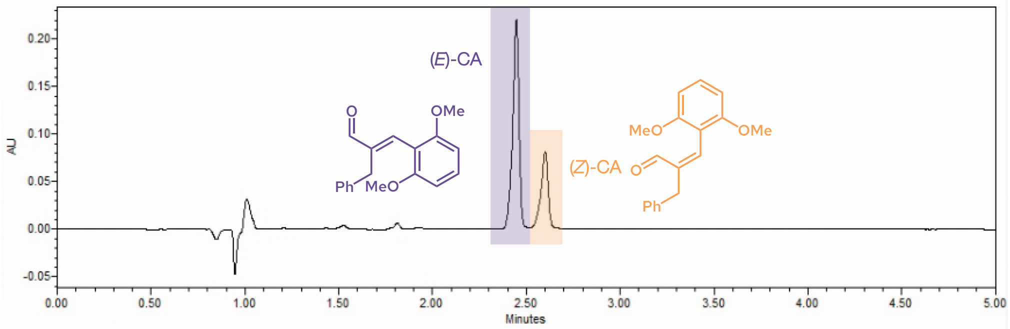

Figure S14. UPLC chromatogram (214 nm channel) of a ~2.5:1 mixture of ***(E)*-CA**:**(Z)-CA.**

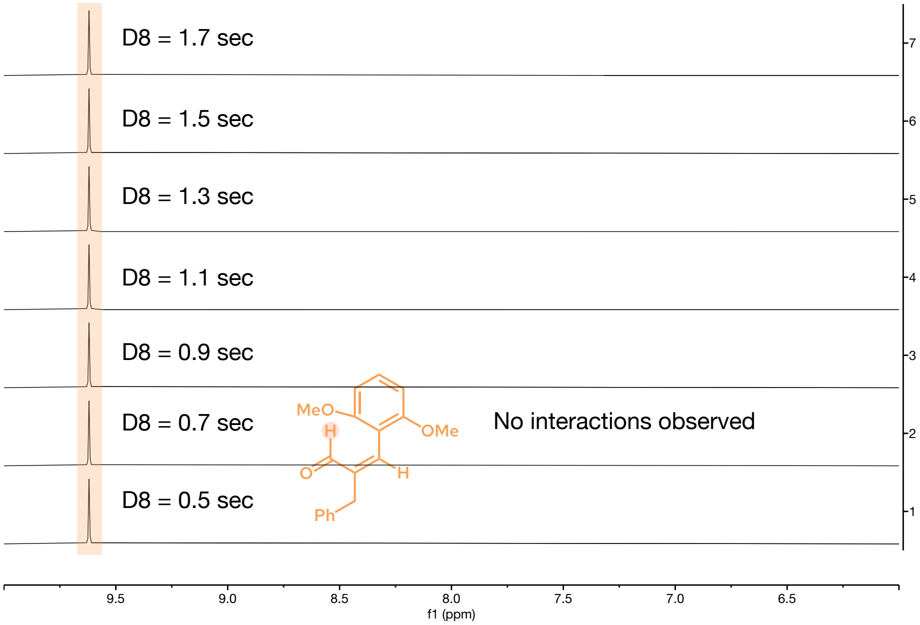

Figure S15. 1D ^{1}H^{1}H NOESY NMR of **(*Z*)-CA** in the mixture. The resonance corresponding to the aldehyde ^{1}H (9.71 ppm, orange) was excited. No interactions were observed.

3.6.6 Dependence of initial rate of **CA** formation on **1** and **12** loading

Initial rates of **CA** formation (v_{INT}) were determined at several loadings of *n*-butylamine (**1**) and 1,8-oligoether-diamine (**12**) using the procedure detailed in section 3.5 to determine the dependence of initial rate on each catalyst. First (R^2 = 0.914, Figure S16) and second order (R^2 = 0.954, Figure S17) fits of the kinetics data for **1** were poorer than an ~1.5-order fit (R^2 = 0.997, Figure S18). Thus, an approximately 1.5-order rate dependence was observed for monoamine **1** using initial rate analysis to determine the reaction order of **1**.

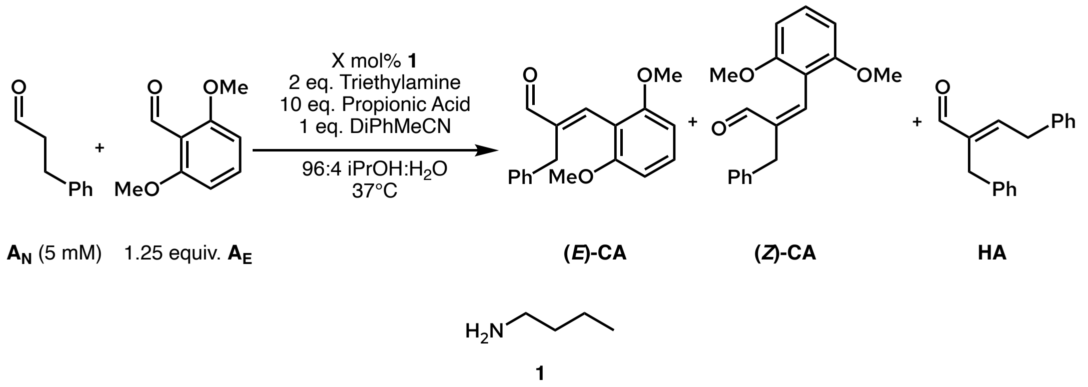

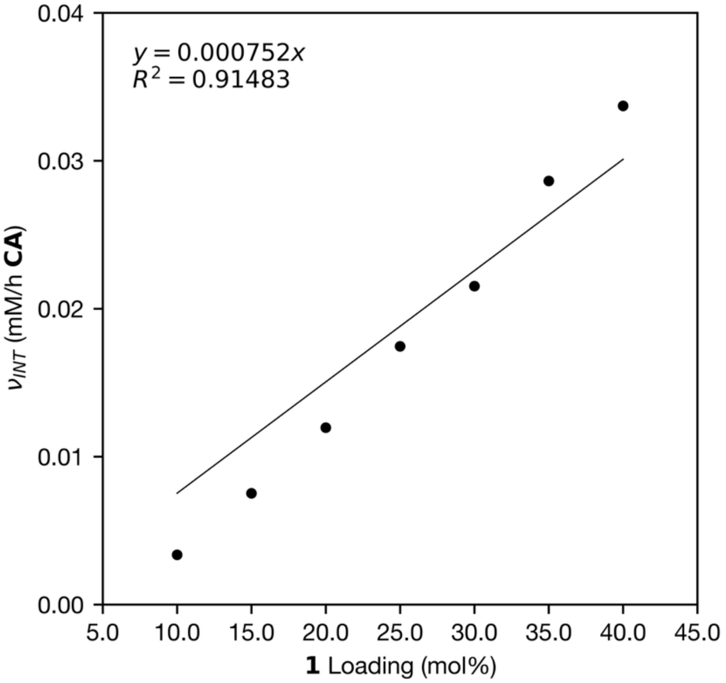

Figure S16. First order fit for dependence of initial rate of **CA** formation (v_{INT}) on loading of monoamine **1**.

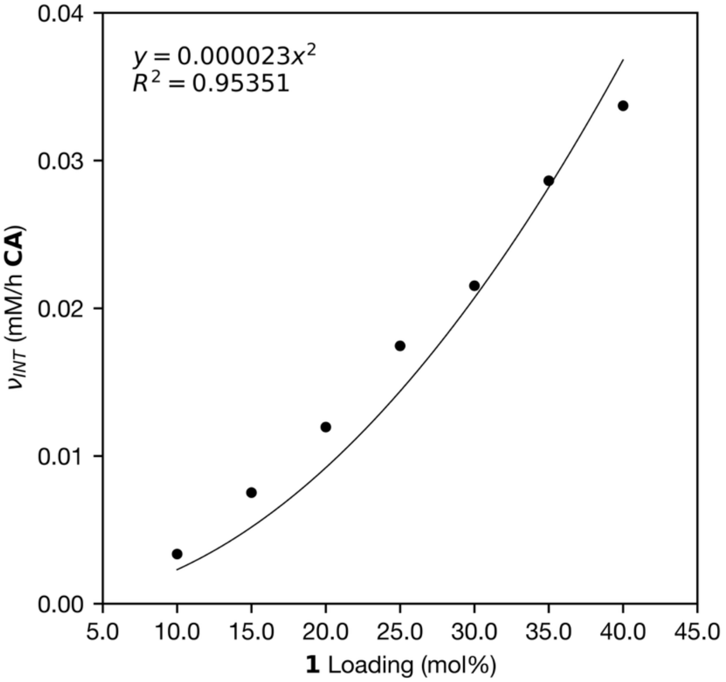

Figure S17. Second order fit for dependence of initial rate of **CA** formation (v_{INT}) on loading of monoamine **1**.

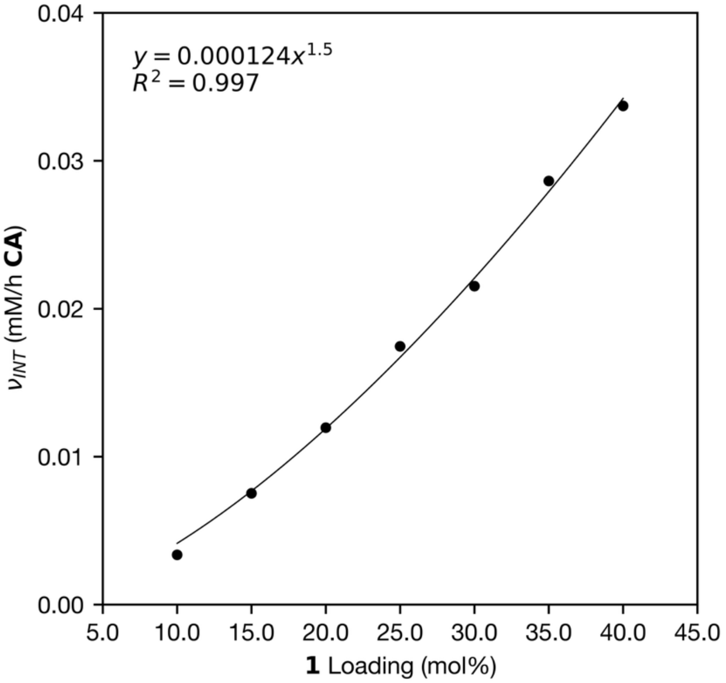

Figure S18. Approximately 1.5-order fit for dependence of initial rate of **CA** formation (v_{INT}) on loading of monoamine **1**.

Oligoether-linked diamine **12** displayed an approximately 1.0-order rate dependence of initial rate on catalyst loading (Figure S19).

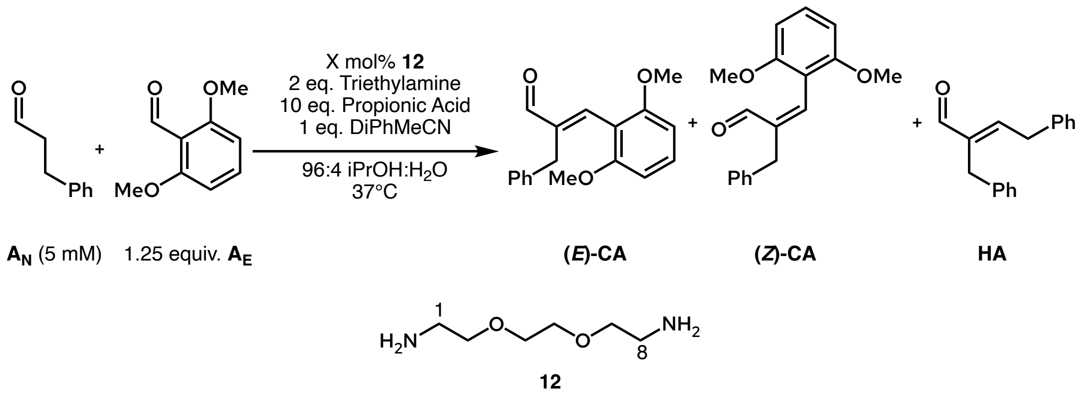

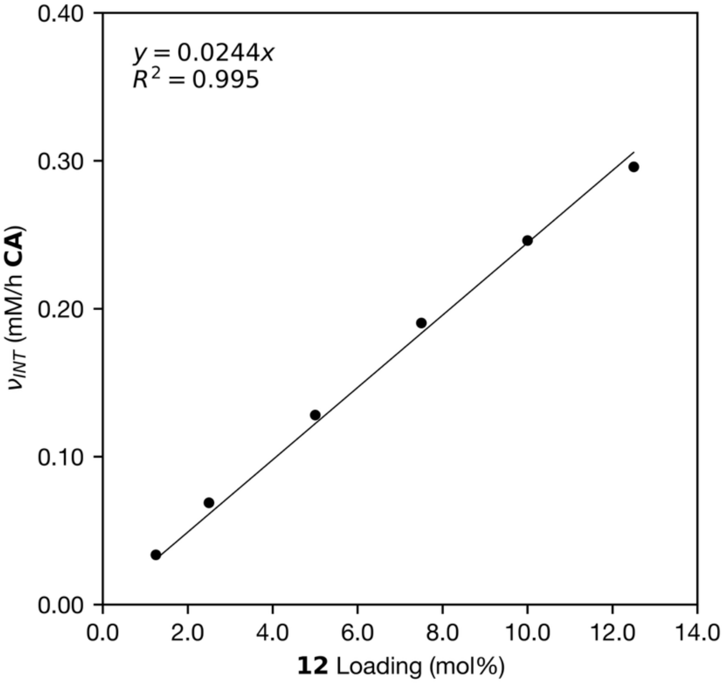

Figure S19. Dependence of initial rate of **CA** formation (v_{INT}) on loading of diamine **12**.

3.6.7 Relative rates of **CA** formation normalized to monoamine **10**

Initial and relative rates of **CA** formation were determined using the procedure detailed in section 3.5. The relative rates reported in Table S4 use monoamine **10** as the basis for calculation of v_{REL}.

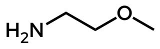

10

Table S4. Initial **CA** formation rates (v_{INT}) and relative **CA** formation rates (v_{REL}), for **1**, **4**, **7**, **9**, **10**, **12**, **14**, **15**, **16**, and **17** relative to monoamine **10**.

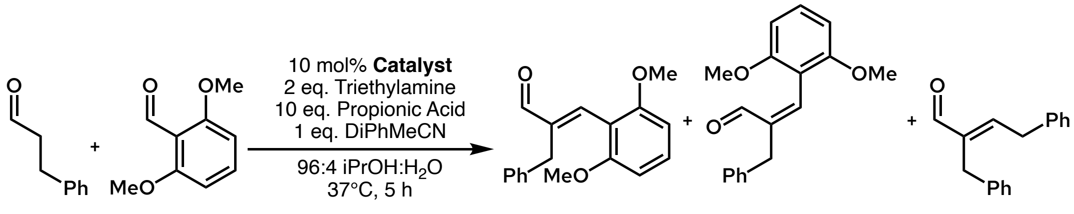

#	Catalyst	v_{INT} (mM/h CA)	v_{REL}
**1***	*n*-butylamine	0.01266	0.4
**4**	1,8-oligomethylene-diamine	0.02161	0.7
**7**	1,14-oligomethylene-diamine	0.04226	1.4
**9**	Acetyl-ACPC-ACPC-Dab-ACPC-ACPC-Dap-β^{3}HTyr-C(O)NH_2	0.2534	8.3
**10***	2-methoxyethylamine	0.0304	1.0
**12**	1,8-oligoether-diamine	0.25434	8.4
**14**	1,14-oligoether-diamine	0.24527	8.1
**15**	1,20-oligoether-diamine	0.11949	3.9
**16***	Acetyl-ACPC-ACPC-Dab-ACPC-ACPC-Ala-β^{3}HTyr-C(O)NH_2	0.03555	1.2
**17***	Acetyl-ACPC-ACPC-Ala-ACPC-ACPC-Dap-β^{3}HTyr-C(O)NH_2	0.01964	0.6

(*) = 20 mol% catalyst used.

3.6.8 In-situ trapping of reaction intermediates

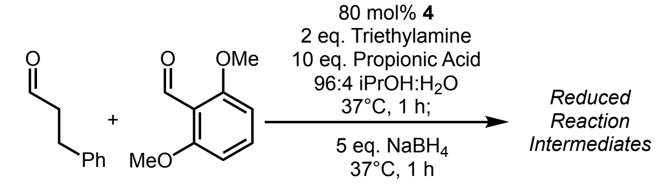

A_N (5 mM) **A_E** (6.25 mM)

Reductive trapping of crossed aldol reaction intermediates was accomplished using a modified literature procedure.[2]

To a 100 mL round bottom flask equipped with a stir bar were added **A_E** (38.7 mg, 0.23 mmol) and catalyst **4** (21.5 mg, 0.15 mmol). The flask was capped with a septum and charged with 37.3 mL of a 96:4 iPrOH:H_2O solution, 24.5 µL of **A_N** (0.19 mmol), 50 µL of triethylamine (0.37 mmol) and 140 µL of propionic acid (1.86 mmol) via micro syringe. The resulting reaction mixture was then heated to 37 °C in an oil bath and allowed to stir for 1 h, after which $NaBH_4$ (35.3 mg, 0.93 mmol) was added in 3 portions. The second and third portions were added after gas evolution from the previous addition had ceased. The reaction solution was then stirred for an additional hour at 37°C, after which the mixture was concentrated under vacuum. The resulting concentrated solution was redissolved in 10 mL of 1:1 MeCN:H_2O and 1 mL of the solution was filtered through a 0.22 µm PTFE filter into a 0.5 dram vial. A 100 µL portion of the filtered sample was further diluted with 900 µL of 1:1 MeCN:H_2O and passed through a fresh 0.22 µm PTFE filter into an LCMS sample vial. A 0.1 µL sample was examined with a UPLC-MS instrument equipped with a 100 mm BEH-C18 column using a gradient of 90% A (H_2O + 0.1% formic acid), 10% B (MeCN + 0.1% formic acid) ramped to 10% A, 90% B over 8 minutes followed by holding at 100% B for 2 minutes and re-equilibration back to starting conditions for 2 minutes. Single ion recording (SIR) mass spectrometry was used to monitor for the elution of diamines that correspond to reduction products derived from enamine/iminium species that are expected to form transiently under the reaction conditions (Figure S20). Major peaks for each SIR chromatogram were integrated to extract mass spectra of each species.

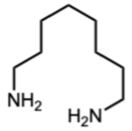

4

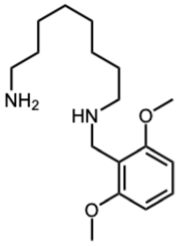

4A

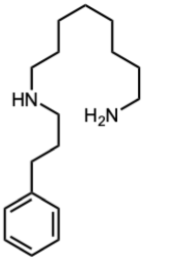

4B

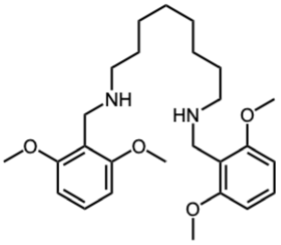

4C

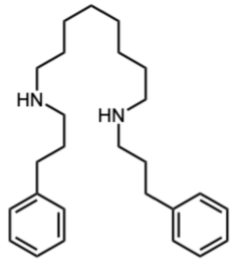

4D

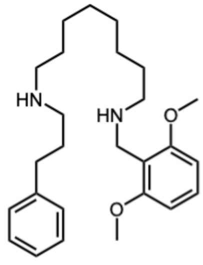

4E

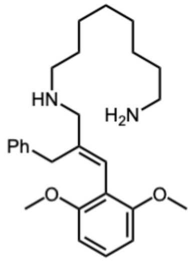

4F

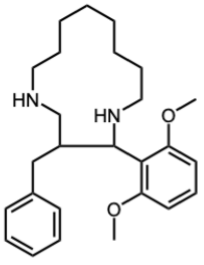

4G

Figure S20. Putative structures of reduced intermediates

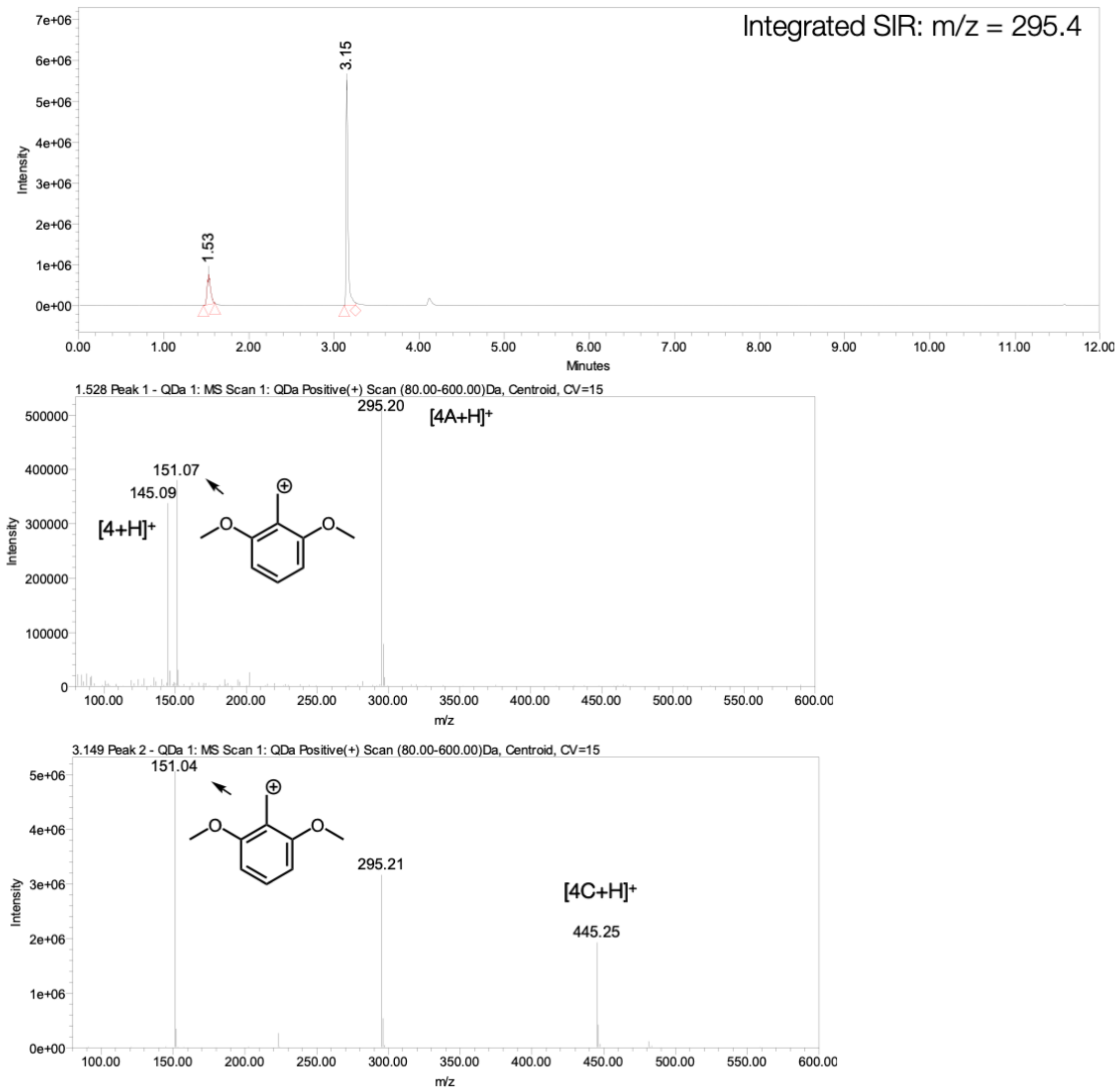

Figure S21. Single ion recording (SIR) of ions with *m/z* value of 295.4 ± 0.5. targeting the molecular ion of [**4A**+H]$^+$. Extracted mass spectra at retention times corresponding to each major peak display molecular ion peaks along with the most abundant fragments. Peak 1 (1.53 min) displays *m/z* consistent with [**4A**+H]$^+$, while peak 2 (3.15 min) displays *m/z* consistent with [**4C**+H]$^+$.

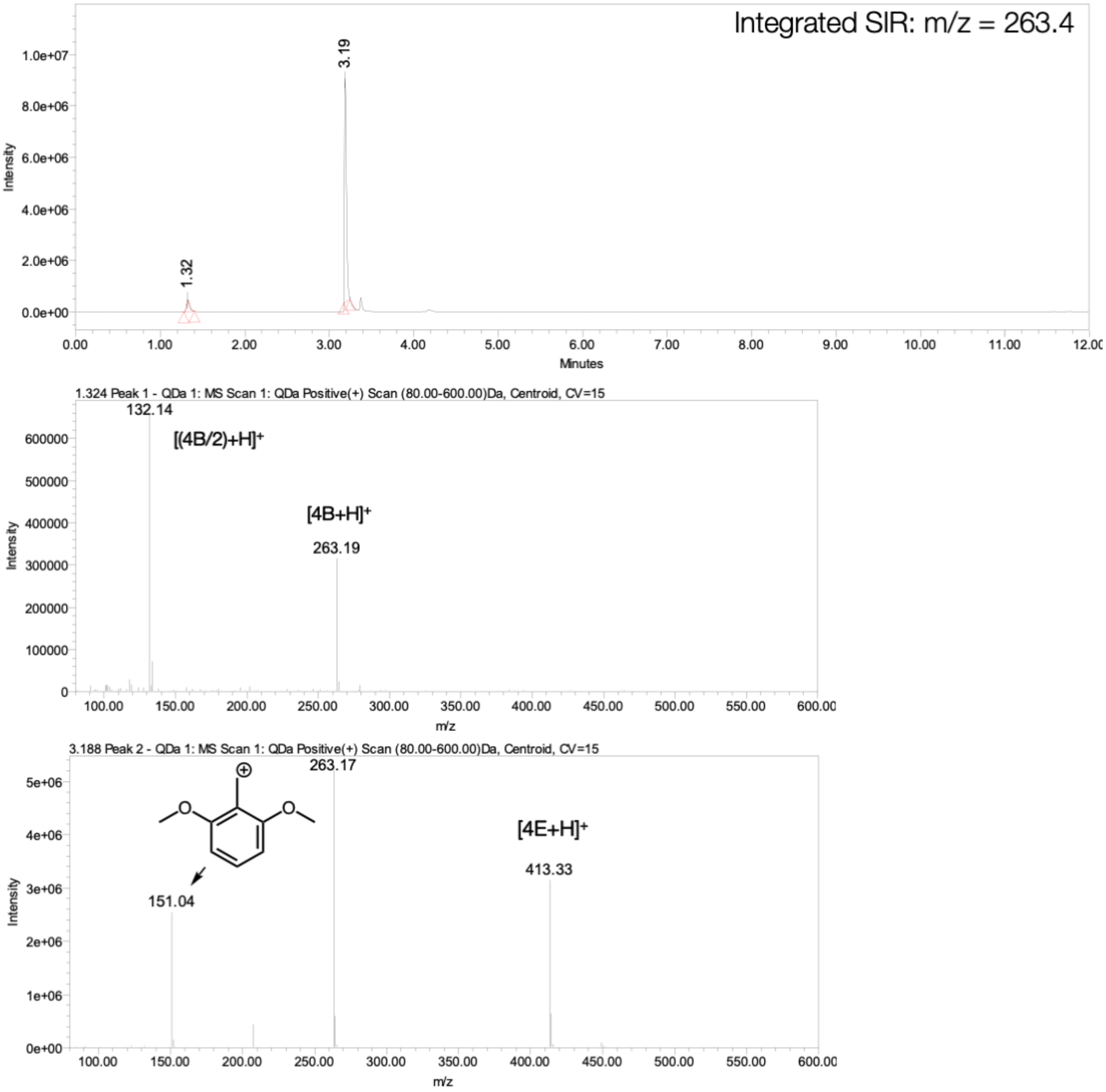

Figure S22. Single ion recording (SIR) of ions with *m/z* value of 263.4 ± 0.5, targeting the molecular ion of [**4B**+H]$^+$. Extracted mass spectra at retention times corresponding to each major peak display molecular ion peaks along with the most abundant fragments. Peak 1 (1.32 min) displays *m/z* consistent with [**4B**+H]$^+$, while peak 2 (3.19 min) displays *m/z* consistent with [**4E**+H]$^+$.

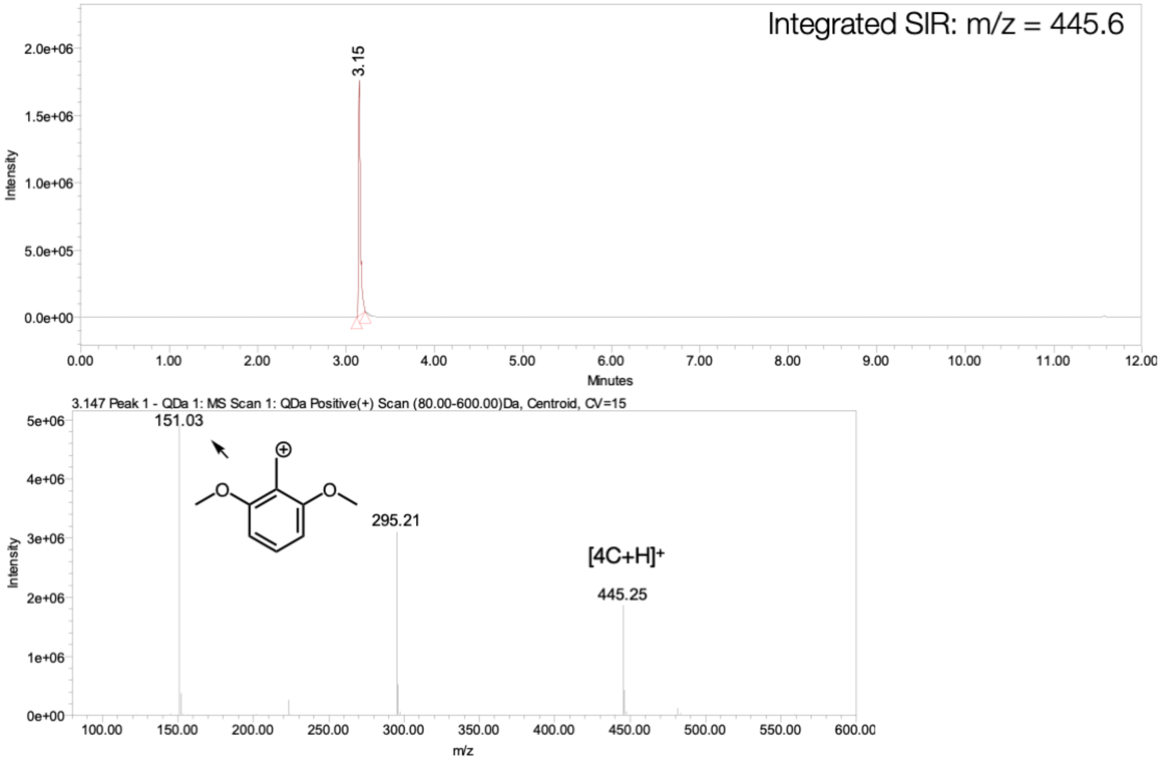

Figure S23. Single ion recording (SIR) of ions with *m/z* value of 445.6 ± 0.5, targeting the molecular ion of [**4C**+H]$^+$. The extracted mass spectrum of the lone peak displays the expected molecular ion peak along with the most abundant fragments.

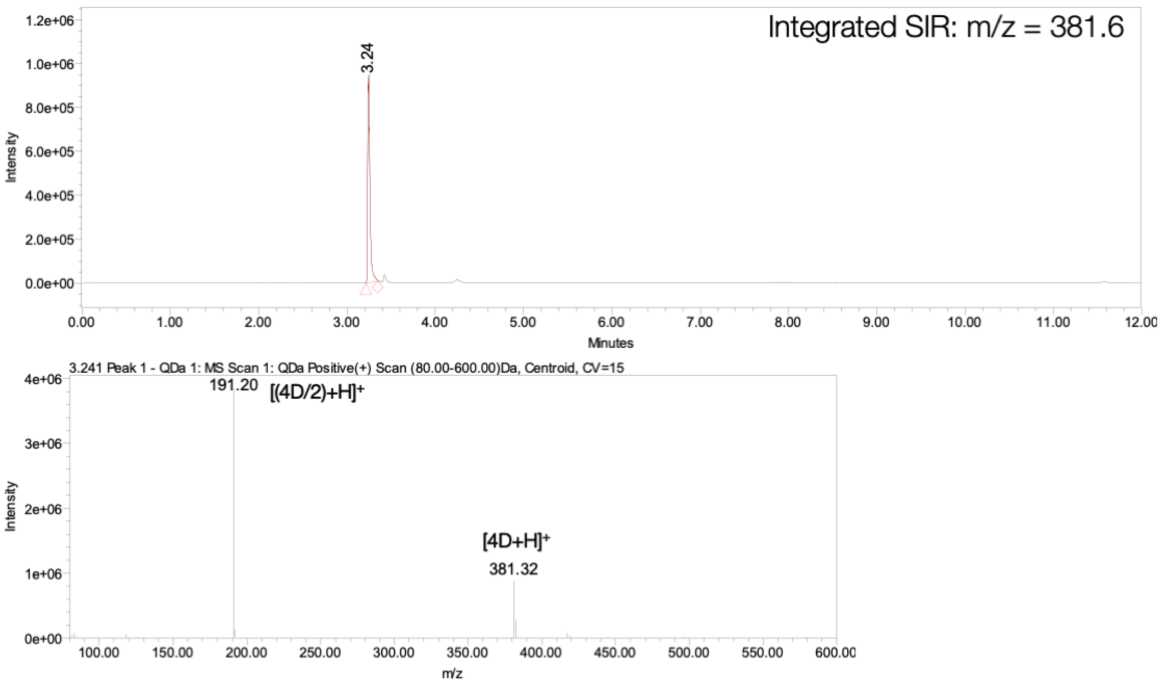

Figure S24. Single ion recording (SIR) of ions with *m/z* value of 381.6 ± 0.5, targeting the molecular ion of [**4D**+H]$^+$. The extracted mass spectrum of the major peak displays the expected molecular ion peak.

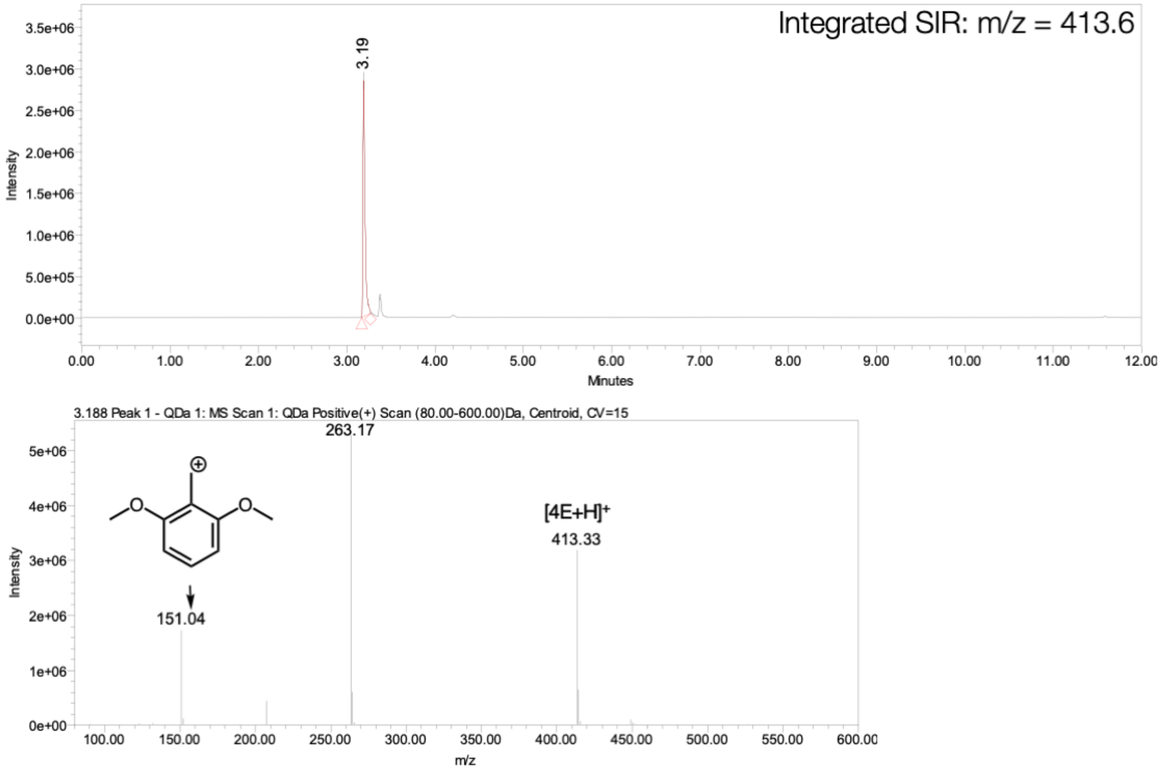

Figure S25. Single ion recording (SIR) of ions with *m/z* value of 413.6 ± 0.5, targeting the molecular ion of [**4E**+H]$^+$. The extracted mass spectrum of the major peak displays the expected molecular ion peak along with the most abundant fragments.

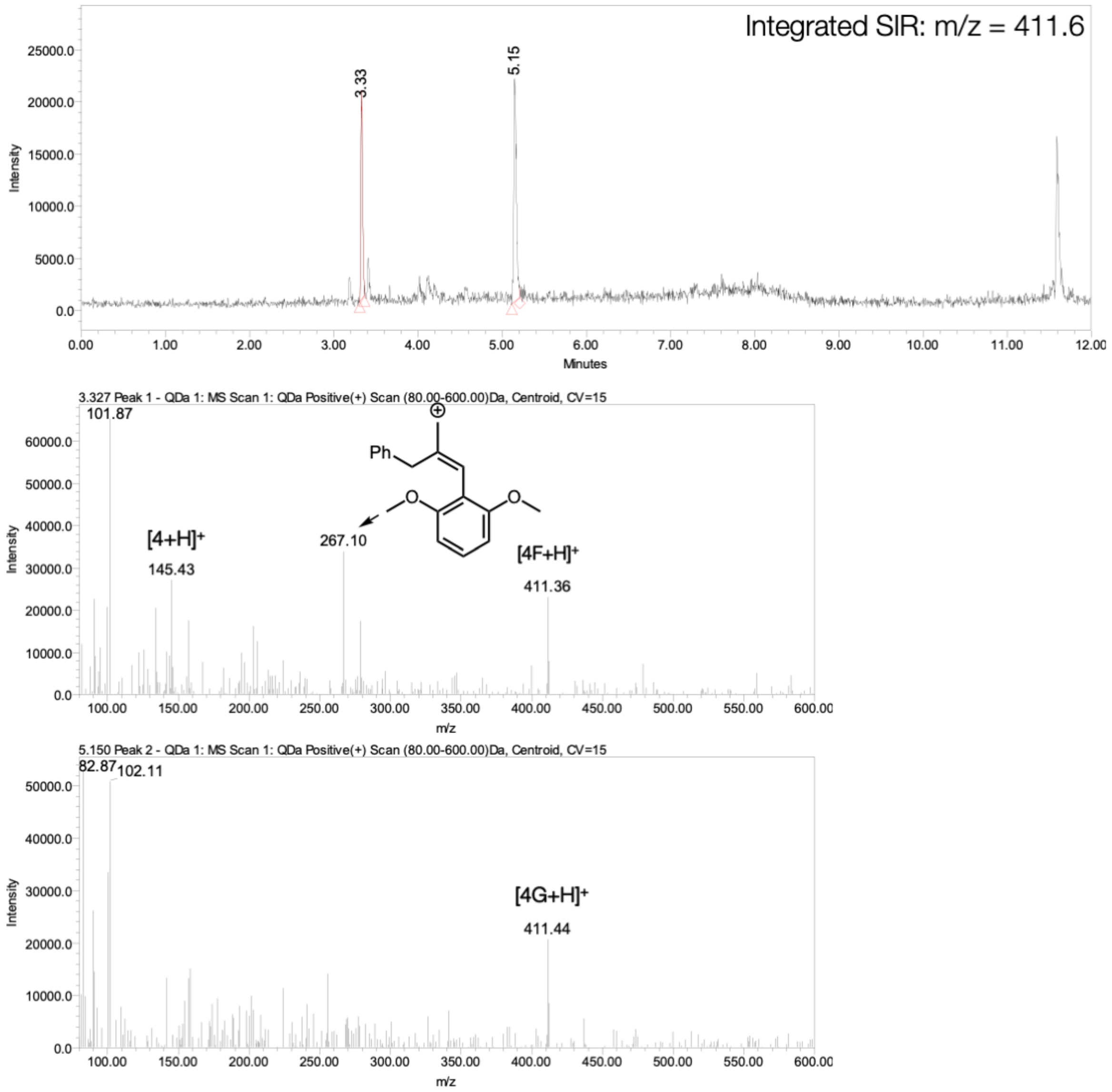

Figure S26. Single ion recording (SIR) of ions with *m/z* value of 411.6 ± 0.5, targeting the molecular ions of [**4F**+H]$^+$ and [**4G**+H]$^+$, which are isomers. Extracted mass spectra at retention times corresponding to each major peak display the expected molecular ion peaks, along with the most abundant fragments. The most abundant ions from peak 1 (3.33 min) match those expected from [**4F**+H]$^+$. The second peak (5.15 min) does not have apparent fragments of high abundance relative to surrounding noise and is tentatively assigned to [**4G**+H]$^+$.

4. Visual Time Normalized Analysis

4.1 Determination of reaction order for **1** and **12**

Reaction profiles depicting the formation of **CA** as a function of time for crossed aldol reactions catalyzed by different amounts of *n*-butylamine (**1**) or 1,8-oligoether-diamine (**12**) were generated using the procedure detailed in section 3.5 (Figures S27 and S29). The reaction profiles obtained for each catalyst were subjected to variable time normalized analysis (VTNA) to determine the approximate reaction orders of **1** (order = 1.5) and **12** (order = 1.0) on **CA** formation. Thirteen different catalyst loadings for **1** (0.05–4.25 mM) and six different catalyst loadings for **12** (0.0625-0.6250 mM) were used. Superimposition of the reaction profiles obtained for each catalyst against the normalized time function t[catalyst]$^{\gamma}$ allows for visual determination of the approximate reaction order for each catalyst. The γ exponent is varied until all reaction profiles for a catalyst overlap. The value of γ where all profiles overlap is the approximate order of the catalyst in the reaction.[4]

For monoamine **1**, γ = 1.5 resulted in the best overlap of all reaction profiles (Figure S31). For diamine **12**, γ = 1.0 resulted in the best overlap of all reaction profiles (Figure S32). Other γ values for each catalyst led to poorer overlap. These results are consistent with dual substrate activation by **1** and bifunctional substrate activation by **12**

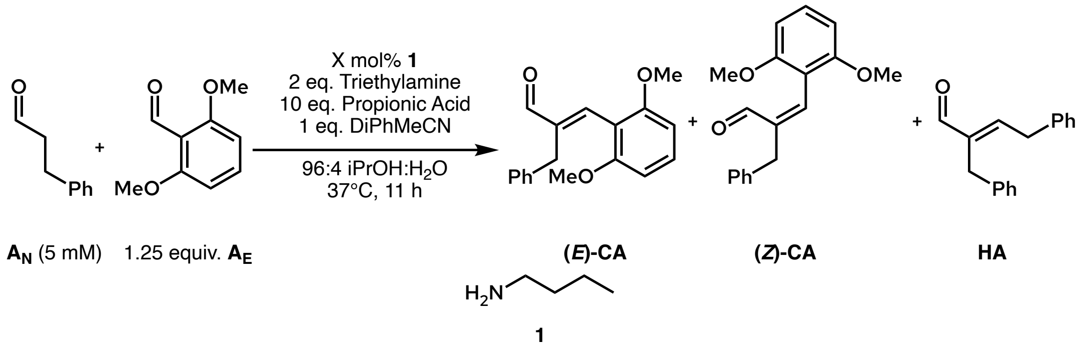

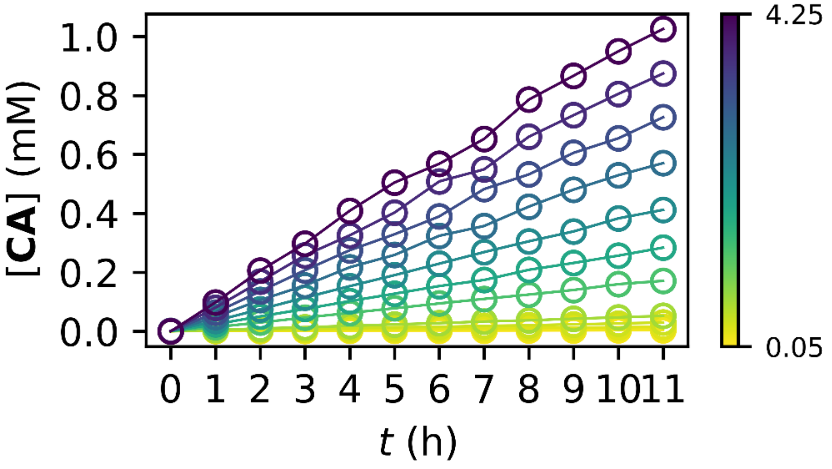

Figure S27. Reaction profiles depicting **CA** concentration as a function of time obtained under the standard reaction conditions using 0.05-4.25 mM **1** as a catalyst (13 different loadings).

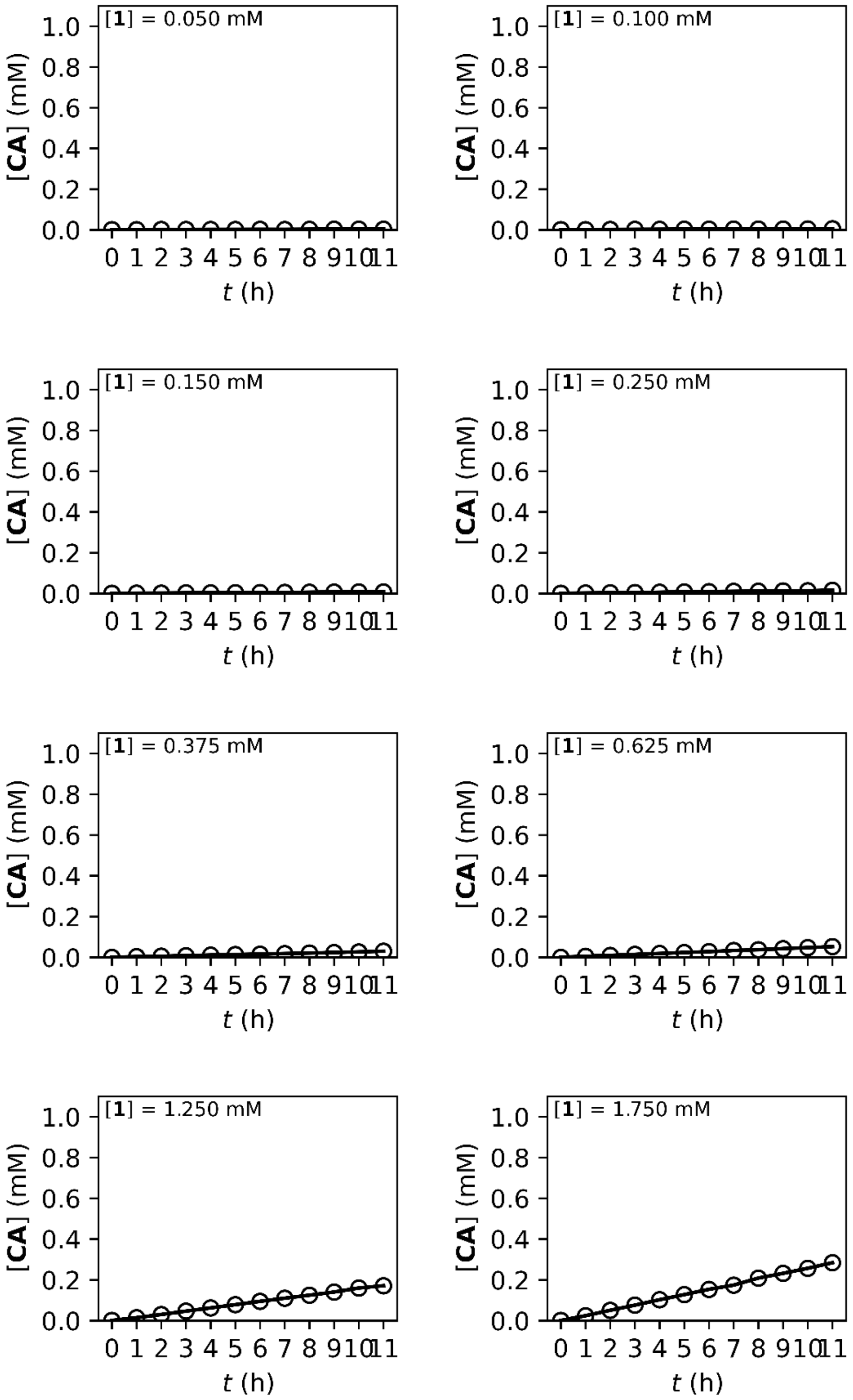
[**1**] = 0.050 mM
[**1**] = 0.100 mM
[**1**] = 0.150 mM
[**1**] = 0.250 mM
[**1**] = 0.375 mM
[**1**] = 0.625 mM
[**1**] = 1.250 mM
[**1**] = 1.750 mM
[**CA**] (mM)
t (h)

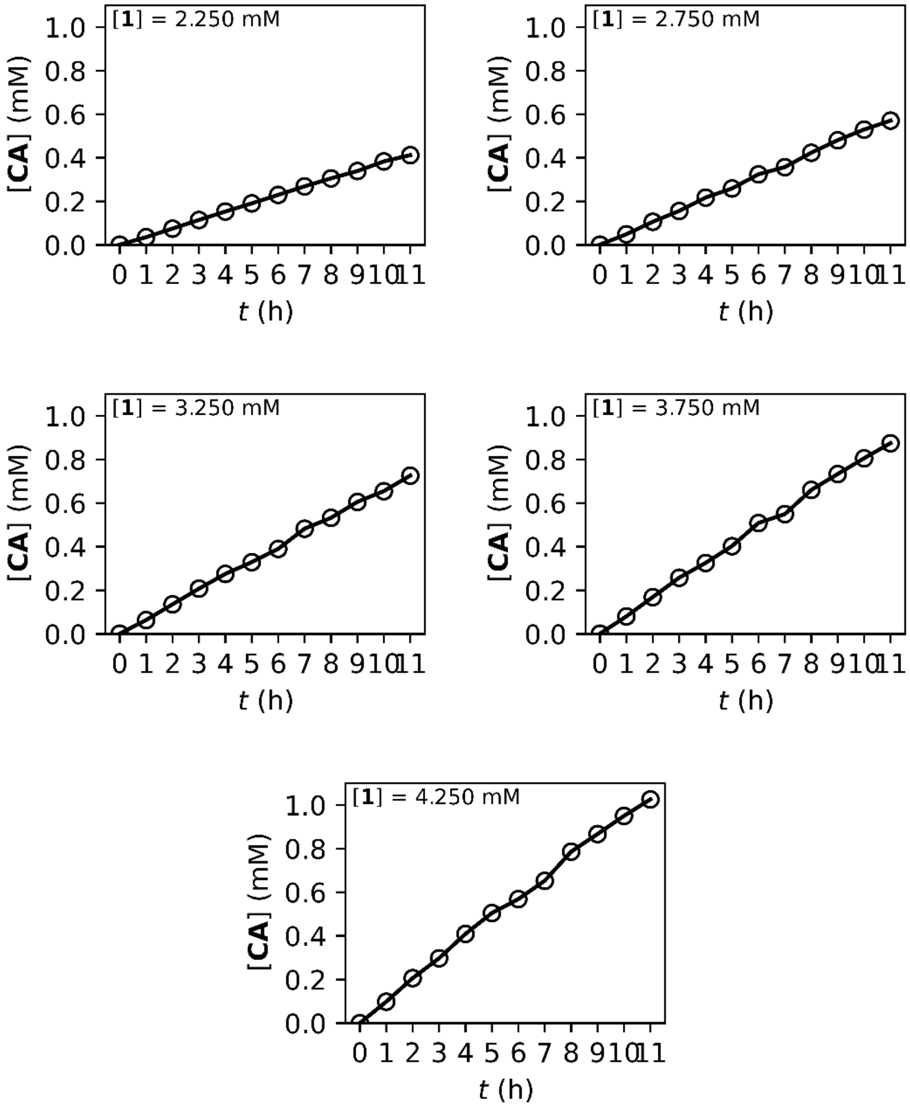

Figure S28. Individual reaction profiles depicting **CA** concentration as a function of time obtained under the standard reaction conditions using 0.05-4.25 mM **1** as a catalyst (13 different catalyst loadings).

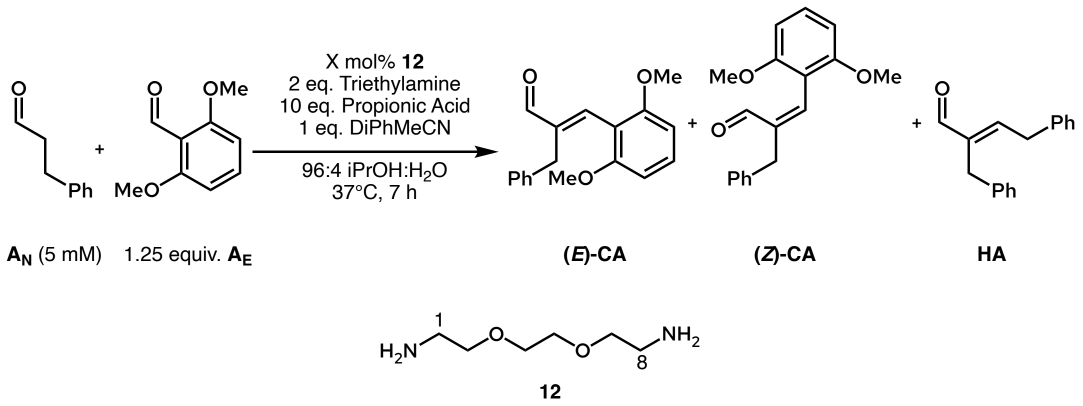

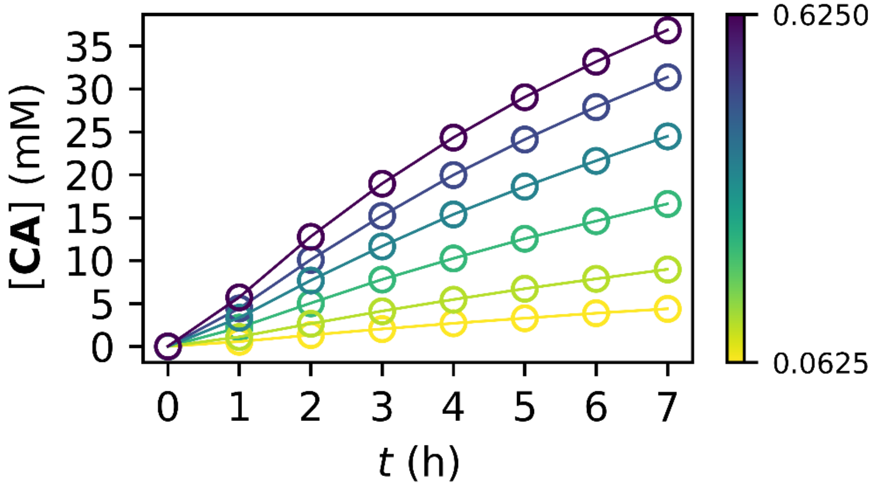

Figure S29. Reaction profiles depicting **CA** concentration as a function of time obtained under the standard reaction conditions using 0.0625-0.6250 mM **12** as a catalyst (6 different loadings).

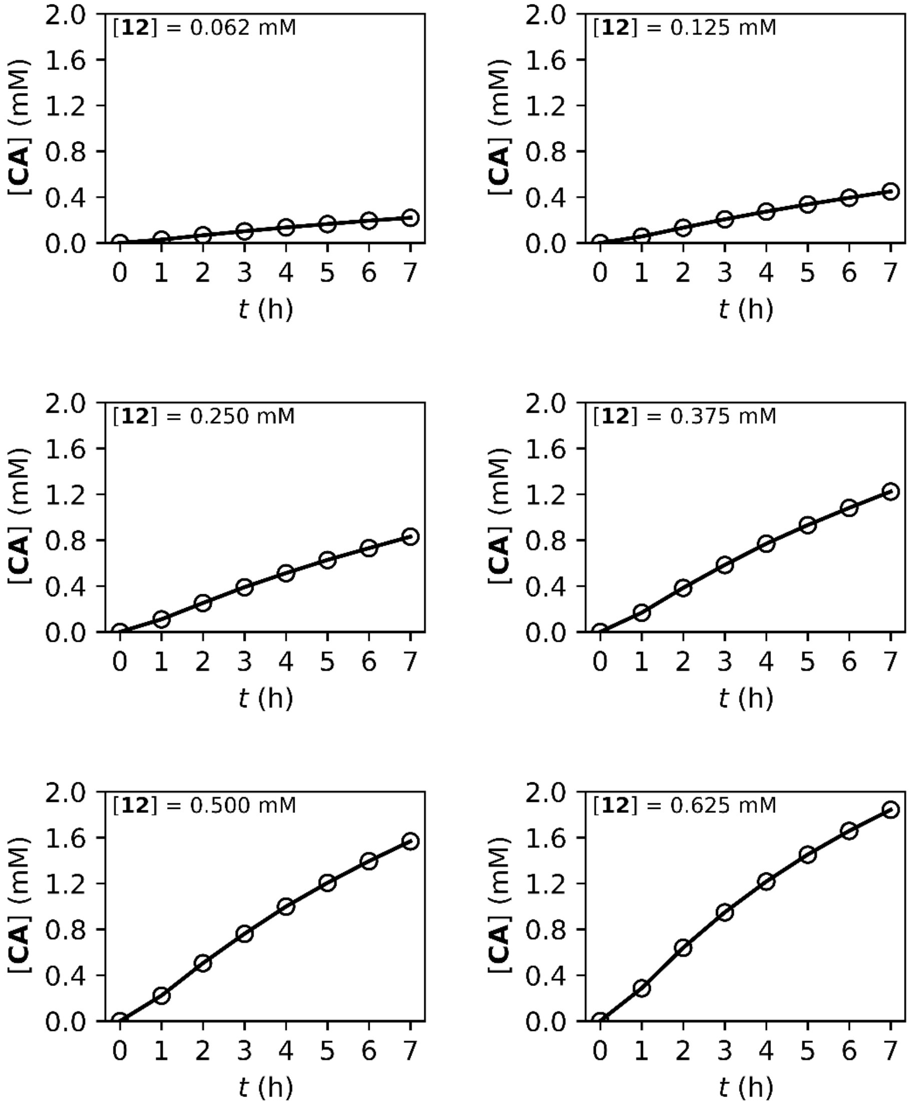

Figure S30. Individual reaction profiles depicting **CA** concentration as a function of time obtained under the standard reaction conditions using 0.0625-0.6250 mM **12** as a catalyst (6 different loadings).

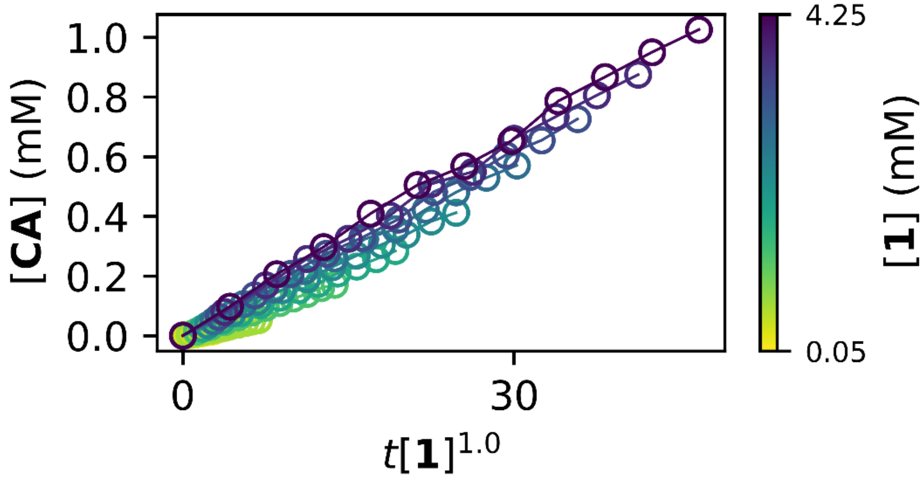

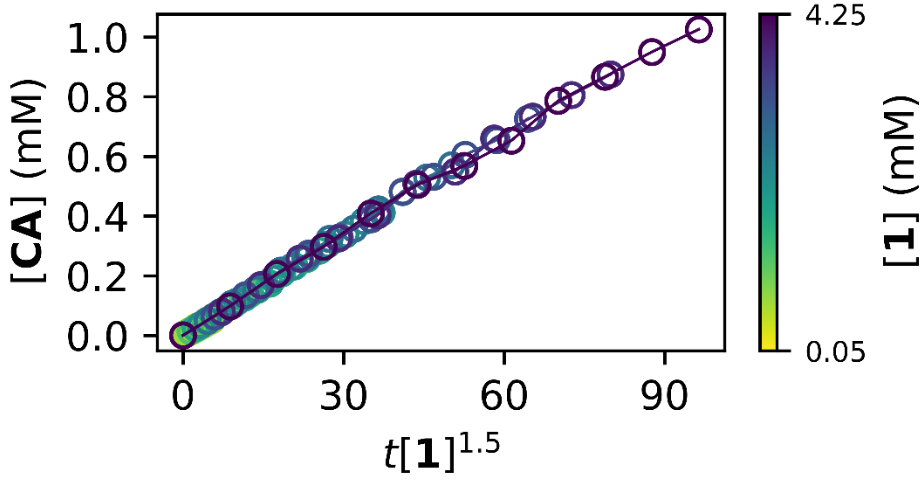

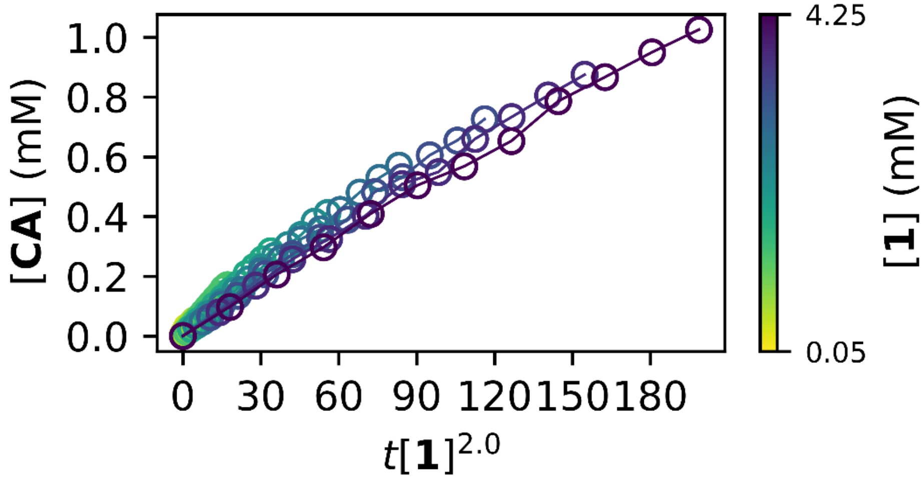

Figure S31. VTNA of monoamine **1** reaction profiles with different γ values. A γ value of 1.5 gives the greatest overlap of reaction profiles indicating an approximate reaction order of 1.5 for monoamine **1**. Other values for the γ exponent give poorer overlap.

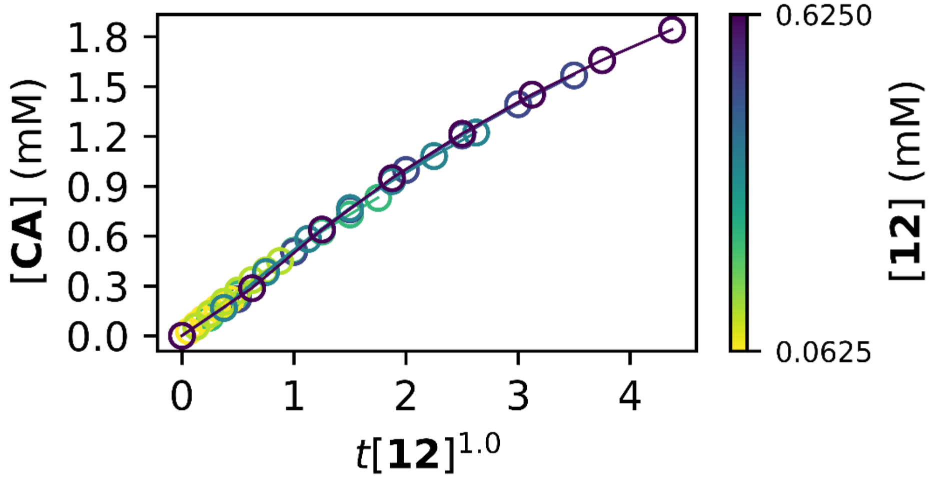
[**CA**] (mM)
1.8
1.5
1.2
0.9
0.6
0.3
0.0
0
1
2
3
4
t[**12**]$^{1.0}$
0.6250
0.0625
[**12**] (mM)

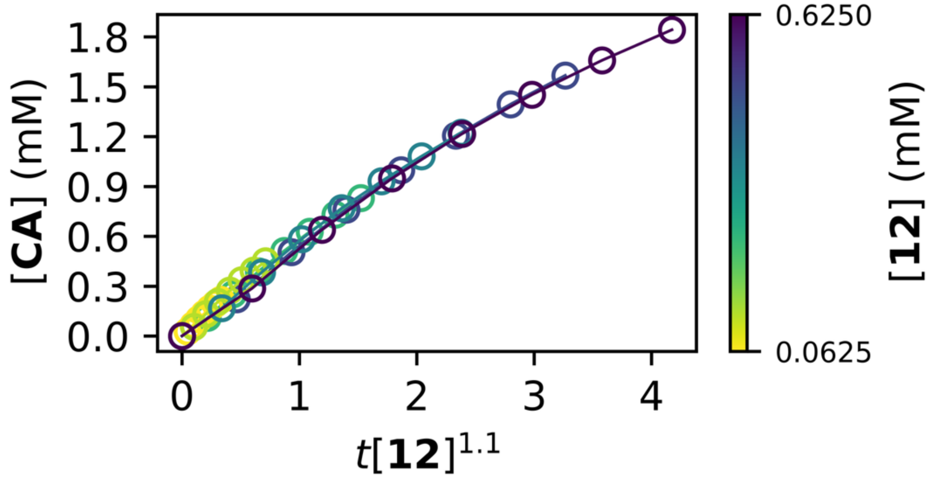
[**CA**] (mM)
1.8
1.5
1.2
0.9
0.6
0.3
0.0
0
1
2
3
4
t[**12**]$^{1.1}$
0.6250
0.0625
[**12**] (mM)

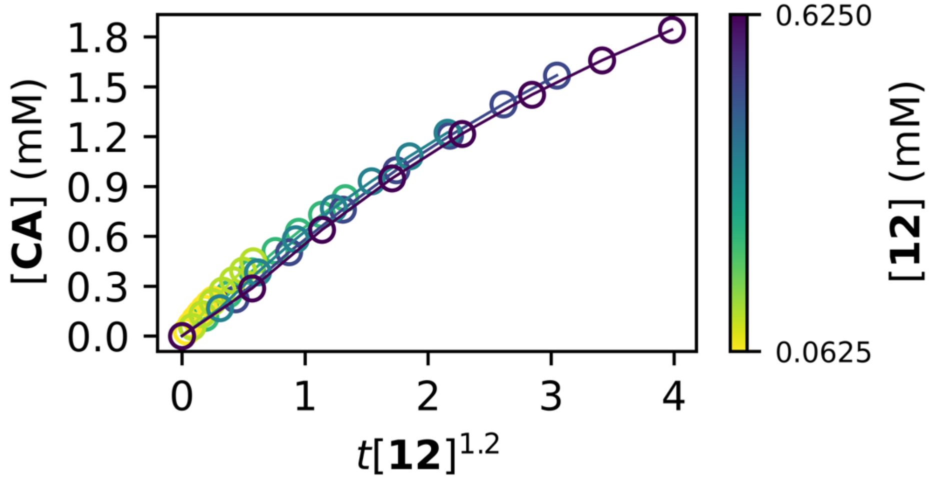
[**CA**] (mM)
1.8
1.5
1.2
0.9
0.6
0.3
0.0
0
1
2
3
4
t[**12**]$^{1.2}$
0.6250
0.0625
[**12**] (mM)

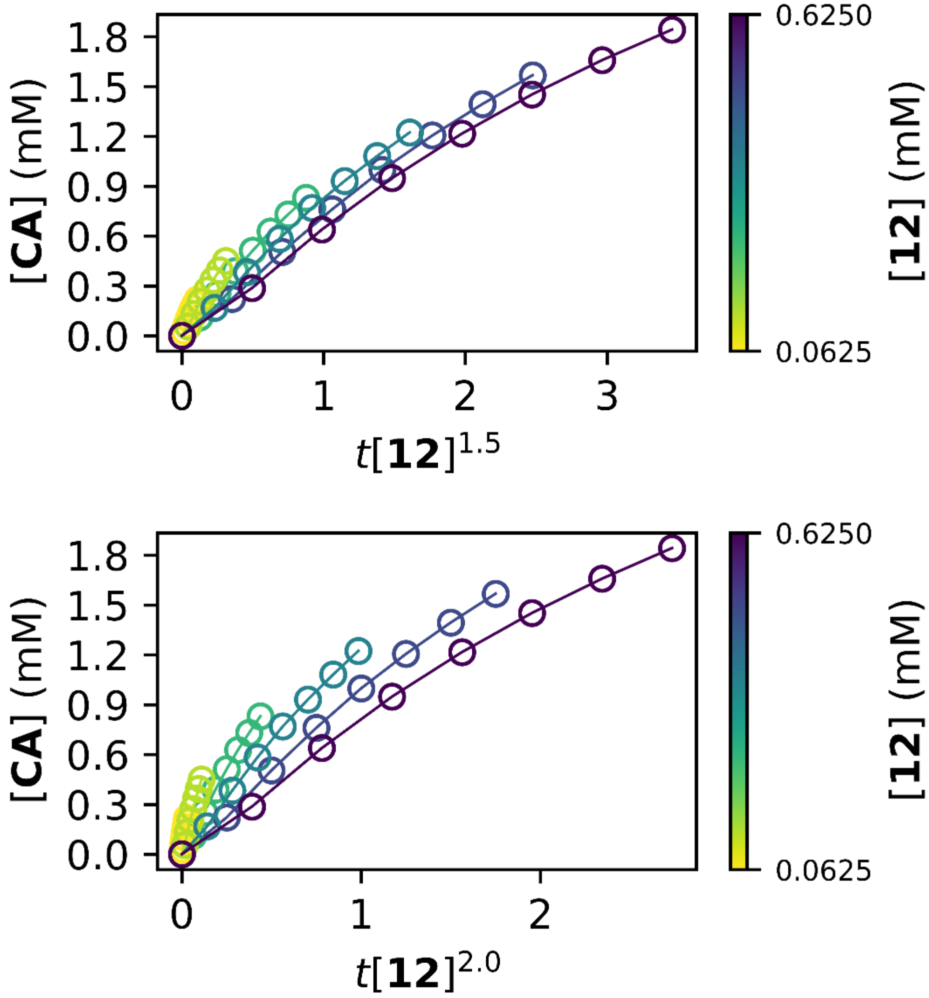

Figure S32. VTNA of diamine **12** reaction profiles with different γ values. A γ value of 1.0 gives the greatest overlap of reaction profiles indicating an approximate reaction order of 1.0 for monoamine **12**. Other values for the γ exponent give poorer overlap.

4.2 Examination of potential inhibition of **1** by **CA**, **HA**, $\mathbf{A_N}$ or $\mathbf{A_E}$

An order of approximately 1.5 was observed for monoamine **1** and is consistent with either a sibling catalytic species mechanism wherein 2 equivalents of **1** act synergistically to form products, or a mechanism wherein 1 equivalent of **1** generates products but is irreversibly inhibited. A sibling catalytic mechanism was proposed by Pihko and coworkers to be operative for a pyrrolidine-catalyzed crossed aldol condensation based on observation of kinetic orders of greater than one for pyrrolidine.[3] Orders greater than one may indicate that multiple catalysts are involved in the rate determining step of a reaction, but can result from irreversible catalyst inhibition pathways occurring in a reaction catalyzed by a single catalyst.[4]

We fit rate laws derived by Burés et al. for the 'sibling catalytic species' (our proposed mechanism) and for a mechanism involving irreversible inhibition to initial rates of **CA** formation (v_{INT}) measured over a wide range of loadings of monoamine **1** (Figure S33).[4] We observed a divergence from the experimental initial rates at low catalyst loadings for the inhibition mechanism. Therefore, we suggest that the total, irreversible inhibition pathway is not consistent with the v_{INT} values observed for **1**.

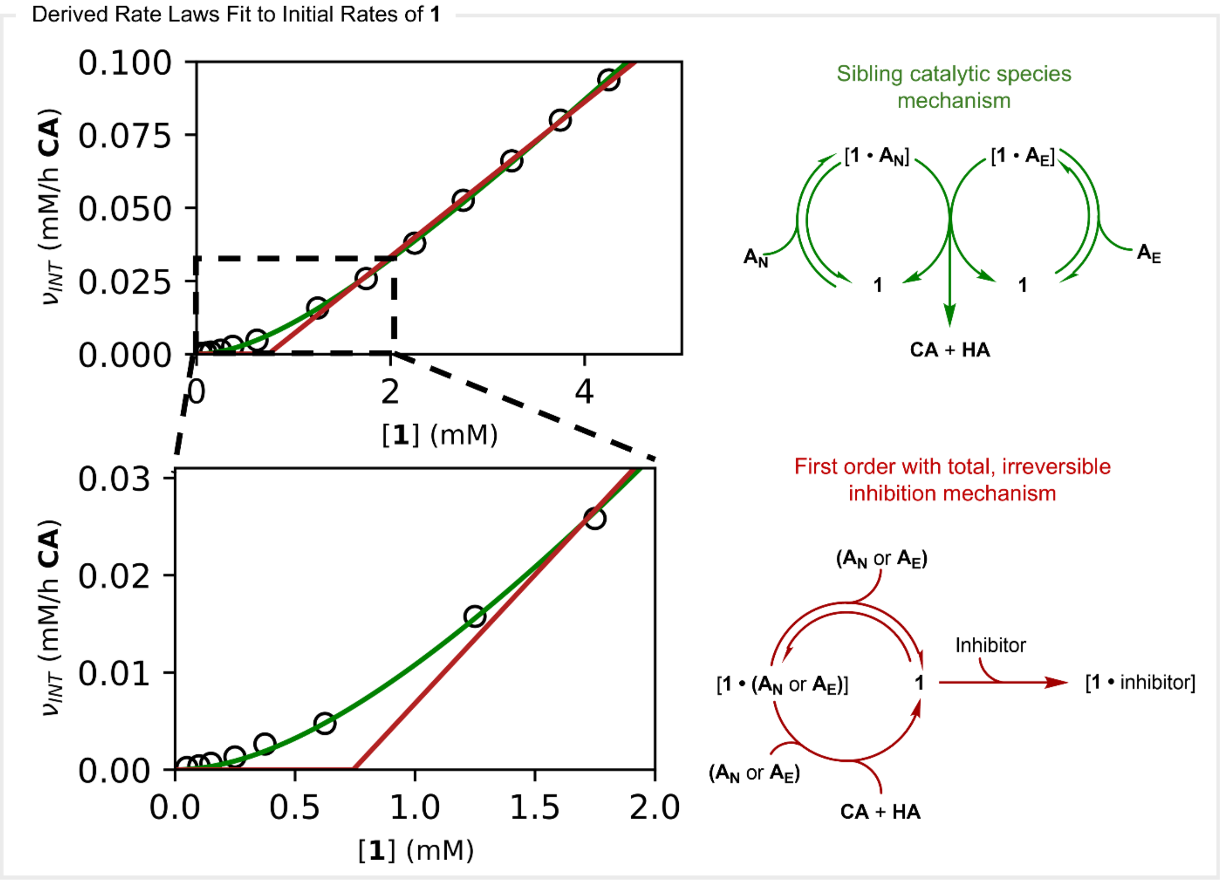

Figure S33. Derived rate laws for the proposed sibling catalytic species mechanism (green) and total irreversible inhibition mechanism (red) fitted using the least mean squares method to experimentally-determined initial rates (black circles) over many loading concentrations of monoamine **1**. Inset highlights that the rate law for the sibling catalytic species mechanism exhibits a superior fit relative to the rate law for the irreversible inhibition mechanism .

4.3 Potential mechanisms for reactions catalyzed by 1

Other reaction mechanisms involving generation of product by 1 equivalent of monoamine **1** and partial or reversible inhibition of **1** can result in plots of v_{INT} as a function of **1** loading indistinguishable from those resulting from our proposed sibling catalytic species mechanism (Figure S34).

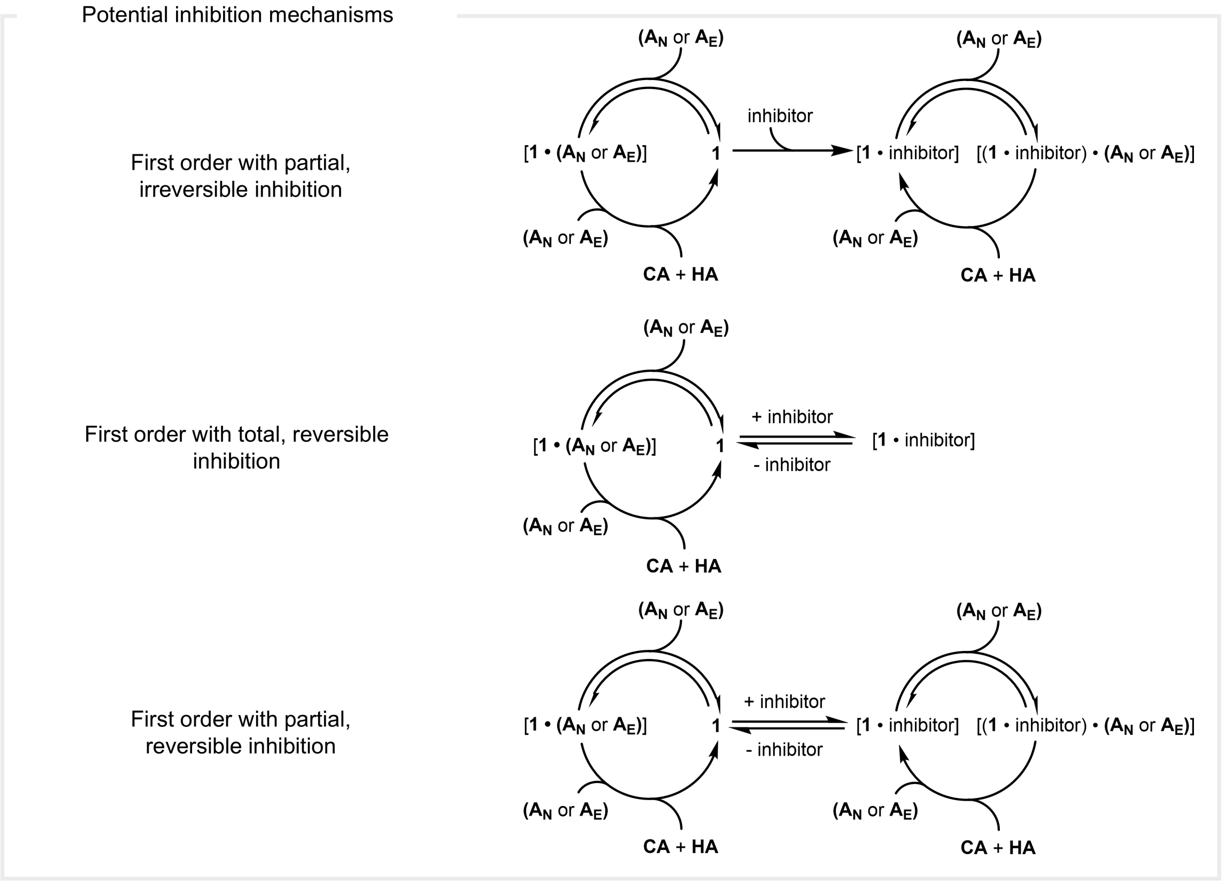

Figure S34. Three potential inhibition pathways that would result in similar kinetics behaviors as observed.

The proposed and partial inhibition mechanisms might be distinguished from one another by measuring v_{INT} at multiple concentrations of **1** and $\mathbf{A_N}$ and $\mathbf{A_E}$.[4] We observed that neither substrate acts as an inhibitor, with increased concentration of either substrate resulting in an increase in v_{INT} at multiple loadings of monoamine **1** (Figure S35).

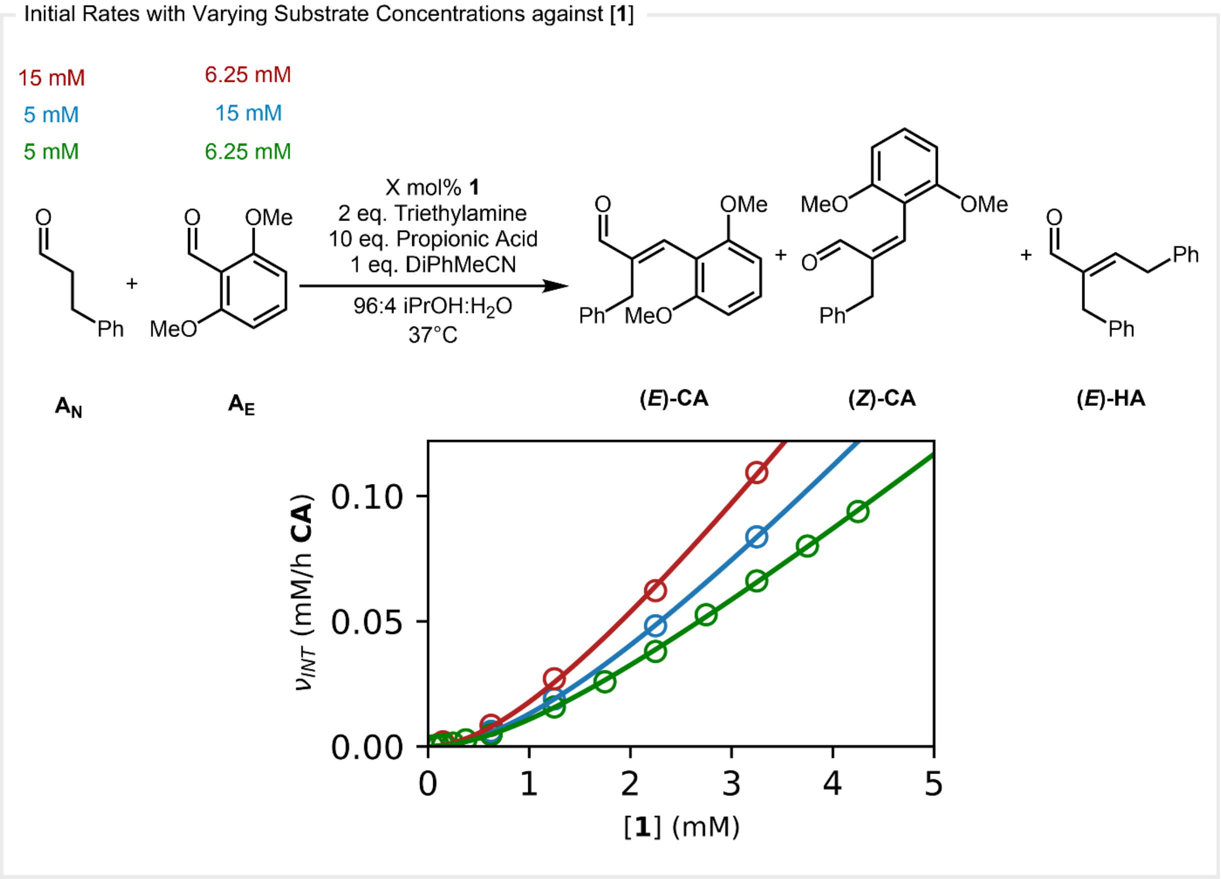

Figure S35. Initial rates using three different combinations of starting $\mathbf{A_N}$ and $\mathbf{A_E}$ concentrations. Each set is fit with the derived sibling catalytic species rate law.

For reactions thought to progress via sibling catalytic species mechanisms, it is common for at least one substrate to act as an inhibitor when the equilibrium between the substrate being bound to the catalyst and the free catalyst favors the substrate being bound.[4] For such systems, the highest reaction rate should be observed under reaction conditions wherein the concentrations of each substrate bound to the catalysts are equal (i.e. $[\mathbf{1} \cdot A_N] = [\mathbf{1} \cdot A_E]$) . If this mechanistic scenario is operative, then increasing the concentration of at least one of the two substrates should slow the reaction rate.[4]

If the equilibrium between each catalyst in the bound and unbound state favors the unbound state in a sibling catalytic species system, then increasing the concentration of either substrate can lead to an increase in reaction rate.

We superimposed derived rate law functions (green lines) for mechanistic scenarios where the unbound state is favored in the sibling catalytic species mechanism over our measured v_{INT} values obtained for monoamine **1** (Figure S36). Increases in the concentrations of each substrate corresponded to increases in initial rates, indicating that neither substrate acts as an inhibitor (Figure S36 B and C). As neither substrate acts as an inhibitor, we suggest that our experimental data are consistent with a sibling catalytic species mechanism for monoamine **1** and a preference for unbound catalysts.

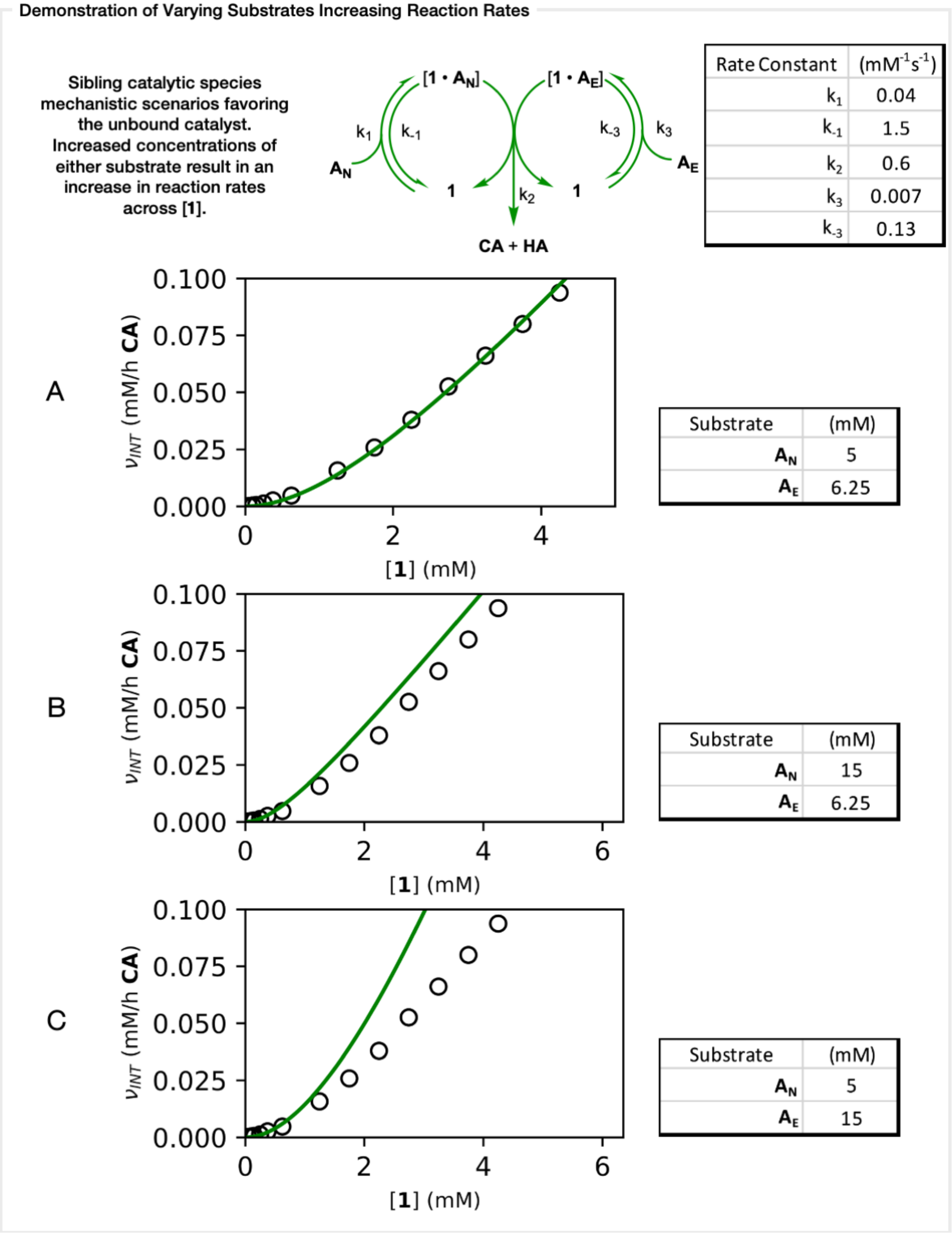

Figure S36. Initial rates plotted against concentrations of **1** with a representative sibling catalytic species derived rate law with set variables. Increases in [$\mathbf{A_N}$]$_{initial}$ and [$\mathbf{A_E}$]$_{initial}$ both correspond with increases in initial rates across catalyst concentrations.

We examined whether product inhibited the reaction by measuring v_{INT} values for crossed aldol condensation reactions catalyzed by **1** in the presence of different amounts of each product, **CA** or **HA** added at the start of reactions (Figure S37). Addition of **CA** or **HA** to the reaction mixture did not slow the initial rates of **CA** formation relative to reactions conducted without added product.

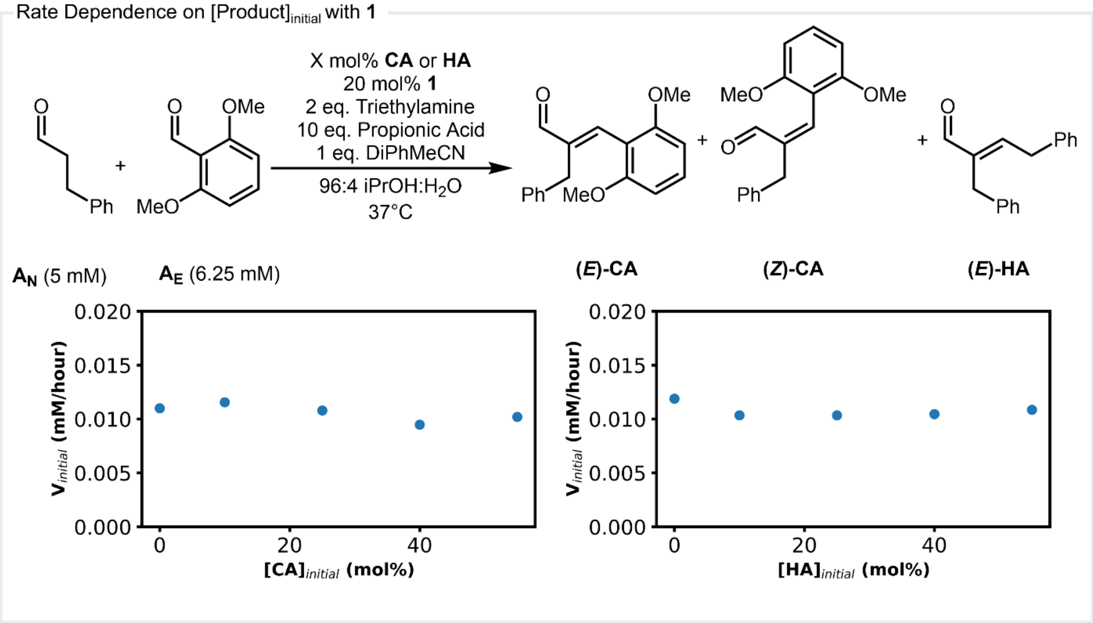

Figure S37. Initial rates of **CA** formation (v_{INT}) using 20 mol% **1** with added **CA** or **HA** products.

The same experiment was conducted using **12** (Figure S38). Again, addition of **CA** or **HA** to the reaction mixture did not slow the initial rates of **CA** formation.

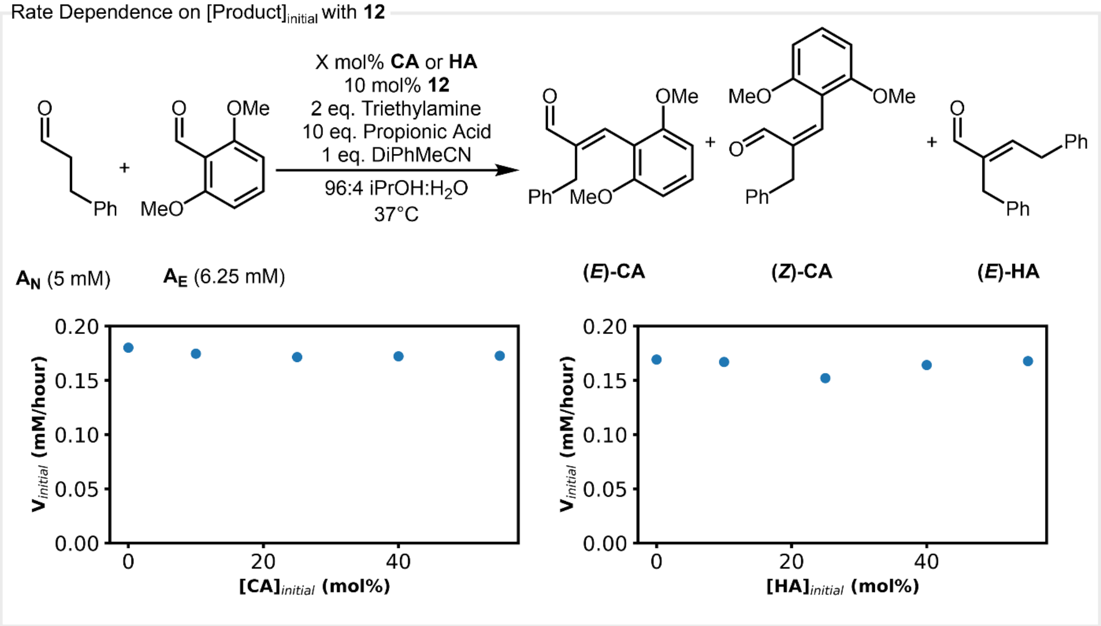

Figure S38. Initial rates of **CA** formation (v_{INT}) using 10 mol% **12** with added **CA** or **HA** products.

5. Initial Rates and Yields

5.1 Yield Summary for 24 h Reactions

Table S5. 24 h **(*E*)-CA**, **(*Z*)-CA** and **HA** yields for catalysts **1-21**

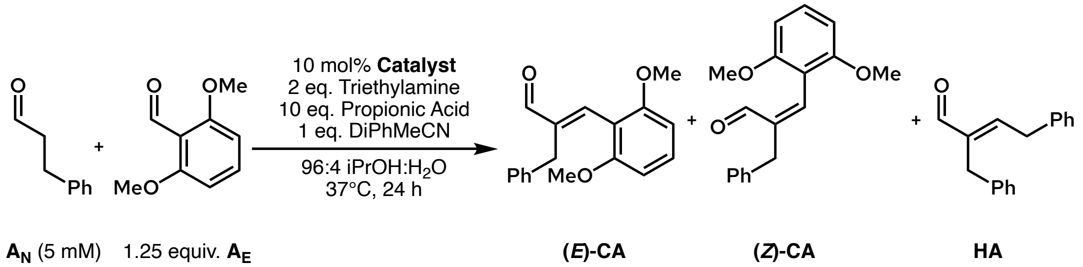

#	Catalyst	(*E*)-CA Yield (%)	(*Z*)-CA Yield (%)	HA Yield (%)
**1***	*n*-butylamine	7	0	1
**2**	1,4-oligomethylene-diamine	5	0	1
**3**	1,6-oligomethylene-diamine	10	0	1
**4**	1,8-oligomethylene-diamine	9	0	1
**5**	1,10-oligomethylene-diamine	15	1	2
**6**	1,12-oligomethylene-diamine	19	1	3
**7**	1,14-oligomethylene-diamine	21	1	3
**8**	1,16-oligomethylene-diamine	20	1	3
**9**	Acetyl-ACPC-ACPC-Dab-ACPC-ACPC-Dap-β^{3}HTyr-C(O)NH_2	54	10	15
**10***	2-methoxyethylamine	14	0	1
**11**	1,5-oligoether-diamine	22	0	2
**12**	1,8-oligoether-diamine	64	2	8
**13**	1,11-oligoether-diamine	58	9	9
**14**	1,14-oligoether-diamine	53	12	6
**15**	1,20-oligoether-diamine	38	6	6
**16***	Acetyl-ACPC-ACPC-Dab-ACPC-ACPC-Ala-β^{3}HTyr-C(O)NH_2	9	0	2
**17***	Acetyl-ACPC-ACPC-Ala-ACPC-ACPC-Dap-β^{3}HTyr-C(O)NH_2	16	0	1
**18**	1,23-oligoether-diamine	36	6	3
**19**	1,29-oligoether-diamine	33	5	3
**20**	1,35-oligoether-diamine	33	4	3
**21***	2-(2-methoxyethoxy)ethanamine	15	0	2

(*) = 20 mol% catalyst used.

5.2 Initial and Relative Rates Summary

Table S6. initial **CA** formation rate (v_{INT}), relative **CA** formation rate (v_{REL}) and 5 h **CA** yield for reactions catalyzed by **1**, **4**, **7**, **9**, **10**, **12**, **14**, **15**, **16**, and **17** relative to monoamine **1**.

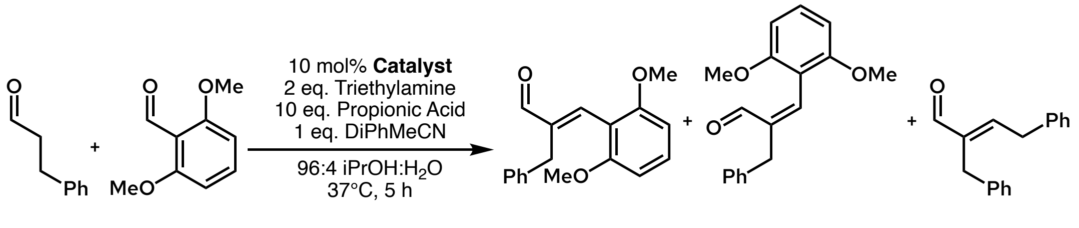

A$_N$ (5 mM) 1.25 equiv. **A$_E$** **(*E*)-CA** **(*Z*)-CA** **HA**

#	Catalyst	v_{INT} (mM/h CA)	v_{REL}	5 h CA yield (%)
**1***	*n*-butylamine	0.01266	1.0	1.3
**4**	1,8-oligomethylene-diamine	0.02161	1.7	1.9
**7**	1,14-oligomethylene-diamine	0.04226	3.3	4.2
**9**	Acetyl-ACPC-ACPC-Dab-ACPC-ACPC-Dap-β^{3}HTyr-C(O)NH$_2$	0.2534	20.0	24.8
**10***	2-methoxyethylamine	0.0304	2.4	3.0
**12**	1,8-oligoether-diamine	0.25434	20.1	24.9
**14**	1,14-oligoether-diamine	0.24527	19.4	23.9
**15**	1,20-oligoether-diamine	0.11949	9.4	11.5
**16***	Acetyl-ACPC-ACPC-Dab-ACPC-ACPC-Ala-β^{3}HTyr-C(O)NH$_2$	0.03555	2.8	2.0
**17***	Acetyl-ACPC-ACPC-Ala-ACPC-ACPC-Dap-β^{3}HTyr-C(O)NH$_2$	0.01964	1.6	3.5

(*) = 20 mol% catalyst used.

Table S7. Initial rates of **CA** formation (v_{INT}) for reactions with 10 to 40 mol% **1**

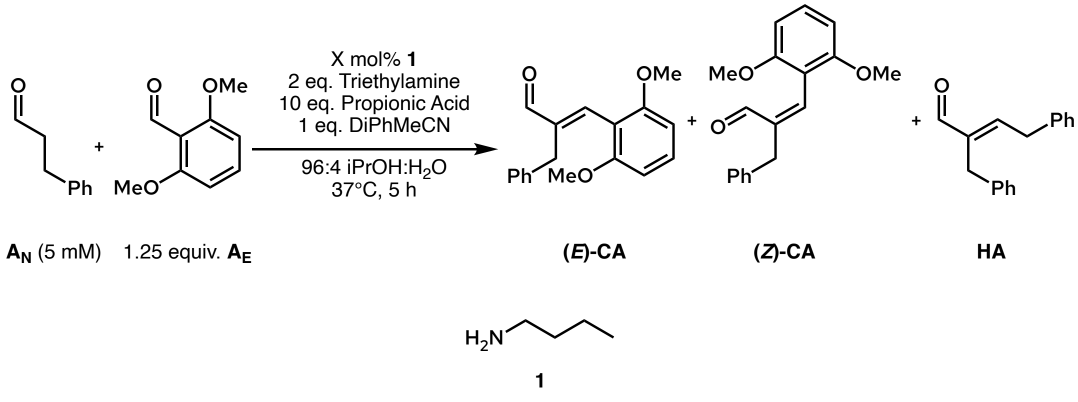

**1 loading (mol%)**	v_{INT} **(mM/h CA)**
10	0.00336
15	0.00752
20	0.01266
25	0.01745
30	0.02152
35	0.02863
40	0.03371

Table S8. Initial rates of **CA** formation (v_{INT}) for reactions with 10 to 40 mol% **12**

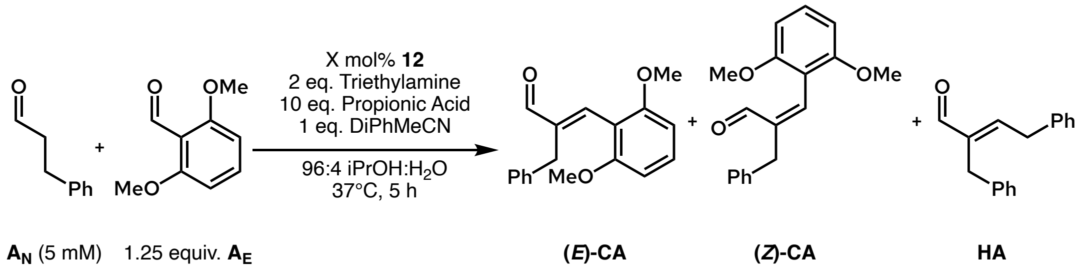

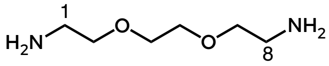

12

**12 loading (mol%)**	v_{INT} **(mM/h CA)**
1.25	0.03367
2.5	0.06878
5.0	0.12814
7.5	0.19036
10.0	0.25434
12.5	0.29586

5.3 Initial Rates for **1**, **4**, **7**, **9**, **10**, **12**, **14**, **15**, **16** and **17**

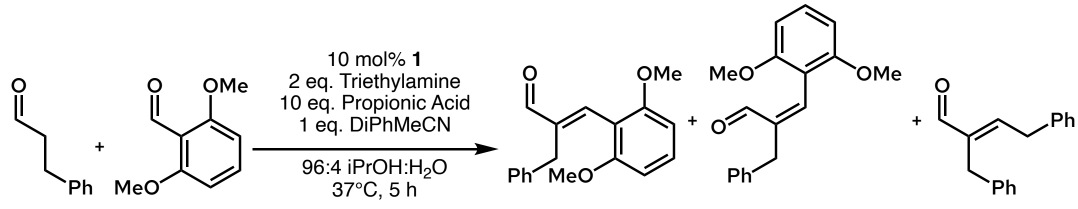

$\mathbf{A_N}$ (5 mM) 1.25 equiv. $\mathbf{A_E}$ **(*E*)-CA** **(*Z*)-CA** **HA**

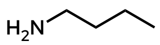

1

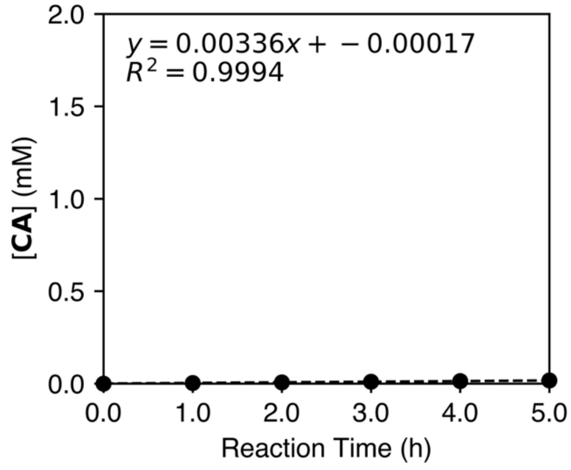

Figure S39. Determination of initial rate of **CA** formation for the aldol reaction catalyzed by 10 mol% **1**.

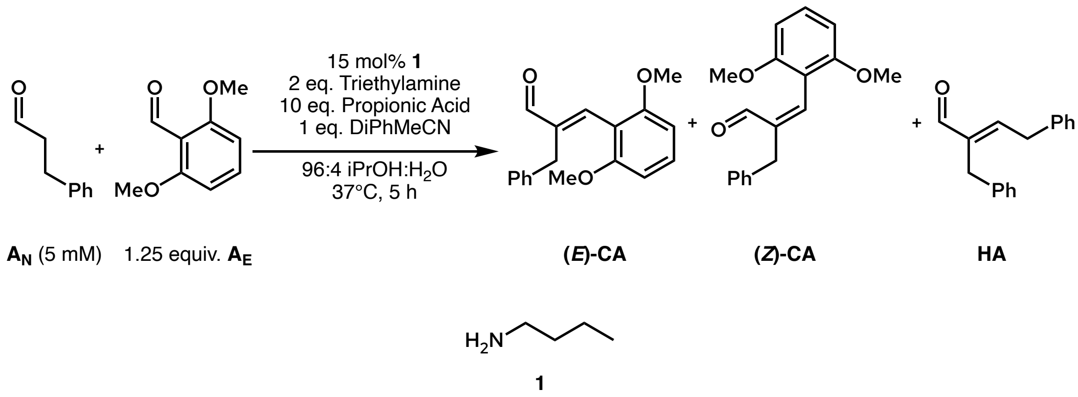

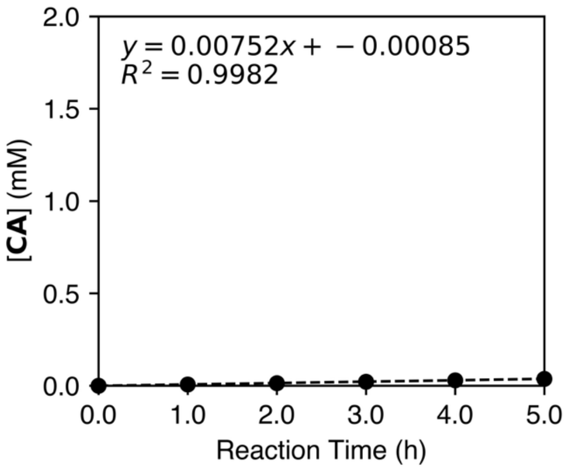

Figure S40. Determination of initial rate of **CA** formation for the aldol reaction catalyzed by 15 mol% **1**.

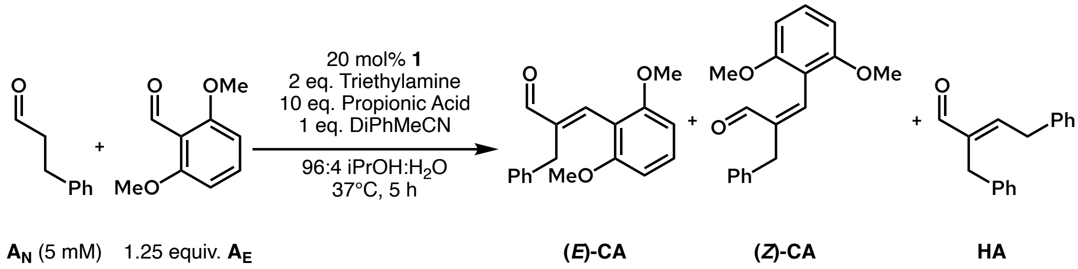

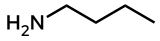

1

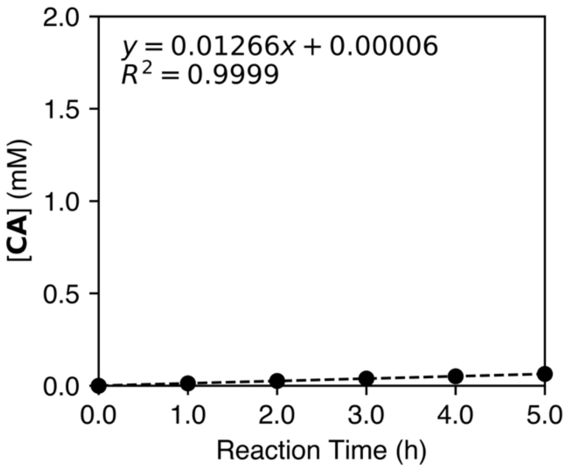

Figure S41. Determination of initial rate of **CA** formation for the aldol reaction catalyzed by 20 mol% **1**.

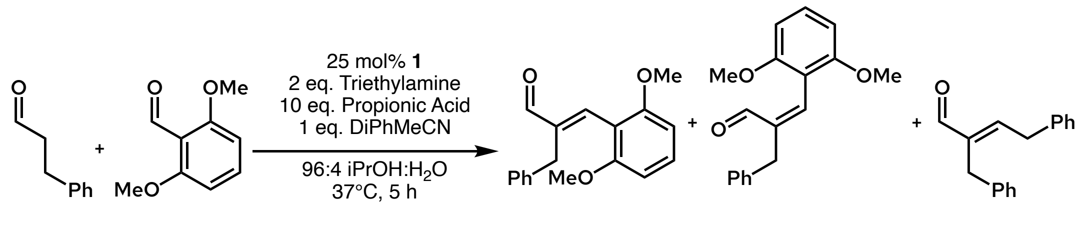

A_N (5 mM) 1.25 equiv. A_E (*E*)-CA (*Z*)-CA HA

1

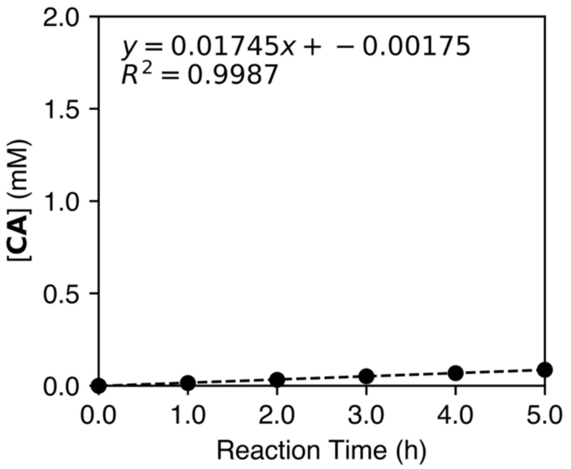

Figure S42. Determination of initial rate of **CA** formation for the aldol reaction catalyzed by 25 mol% **1**.

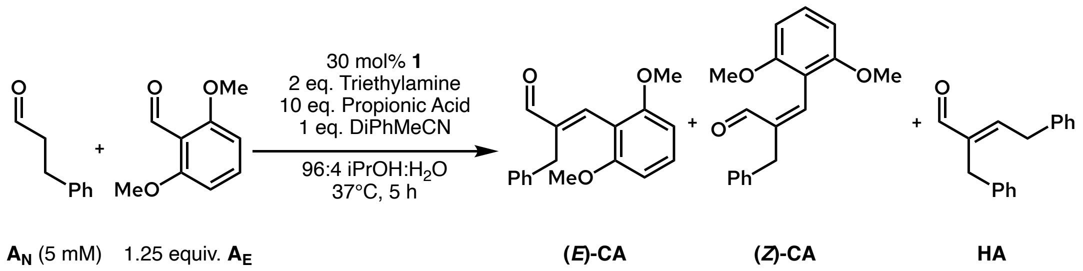

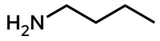

1

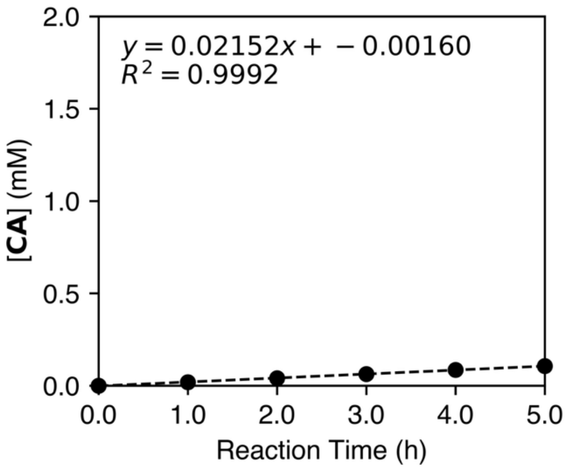

Figure S43. Determination of initial rate of **CA** formation for the aldol reaction catalyzed by 30 mol% **1**.

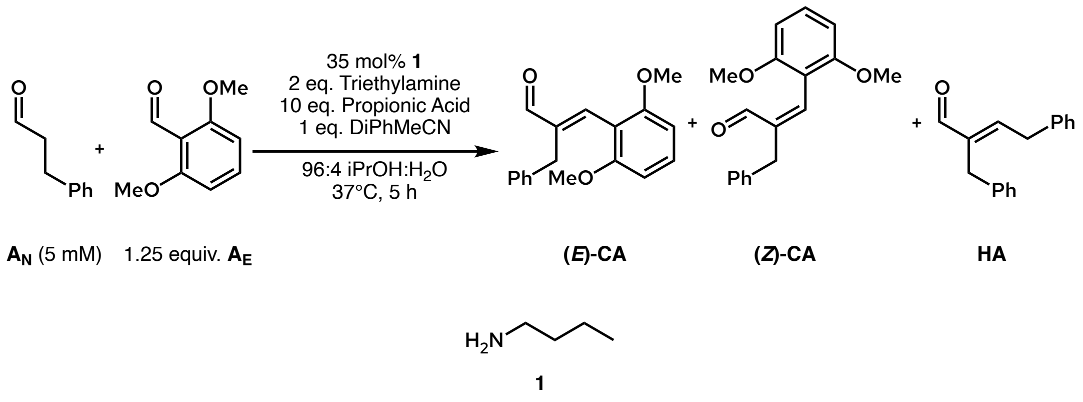

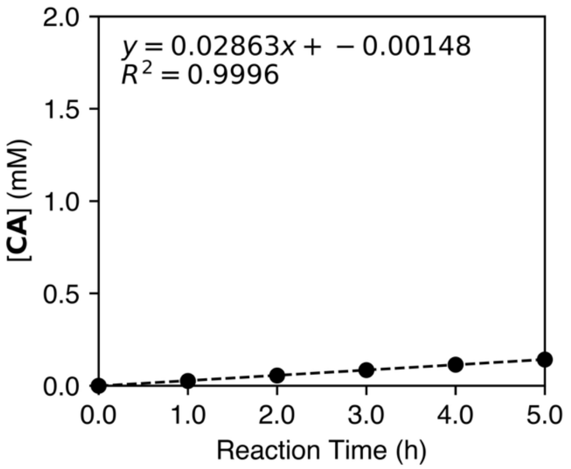

Figure S44. Determination of initial rate of **CA** formation for the aldol reaction catalyzed by 35 mol% **1**.

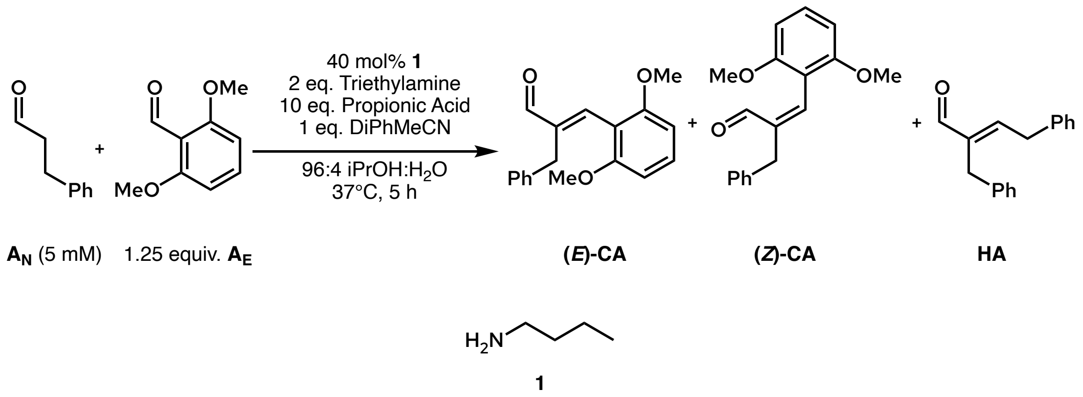

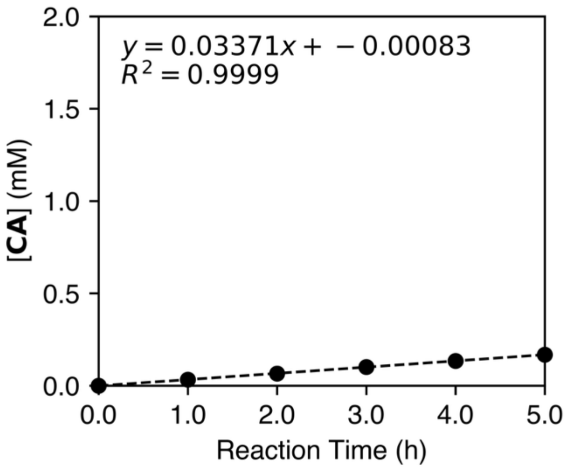

Figure S45. Determination of initial rate of **CA** formation for the aldol reaction catalyzed by 40 mol% **1**.

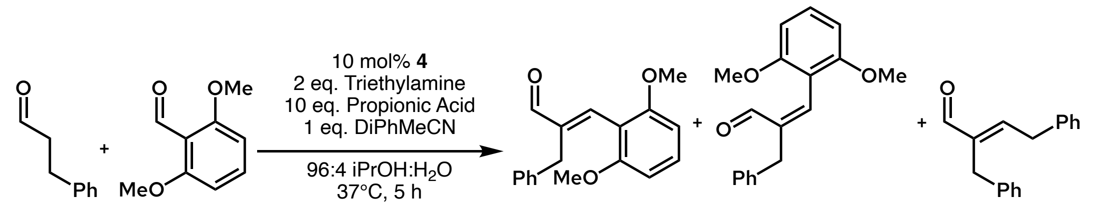

A_N (5 mM) 1.25 equiv. A_E **(*E*)-CA** **(*Z*)-CA** **HA**

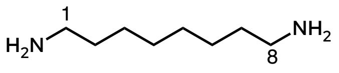

4

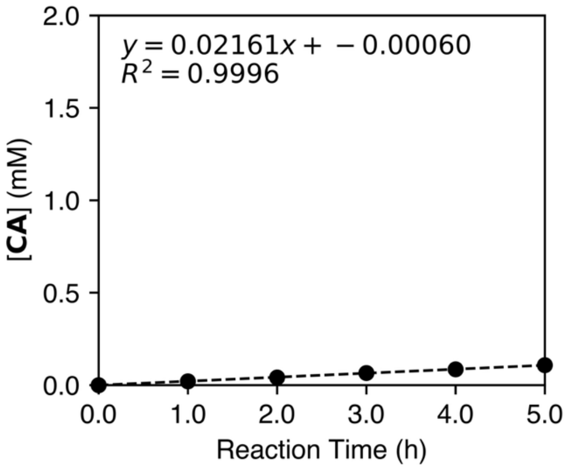

Figure S46. Determination of initial rate of **CA** formation for the aldol reaction catalyzed by 10 mol% **4**.

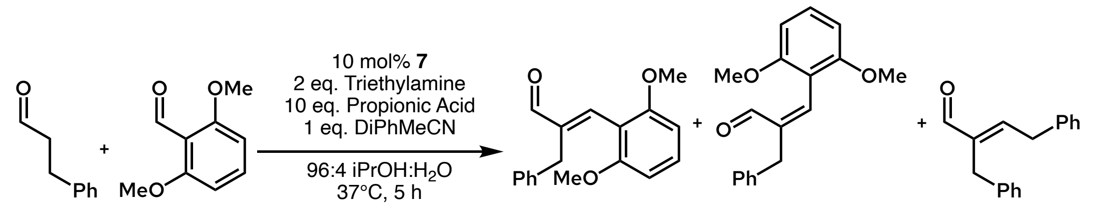

A_N (5 mM) 1.25 equiv. A_E (*E*)-CA (*Z*)-CA HA

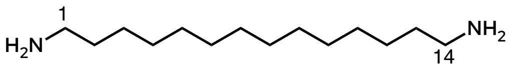

7

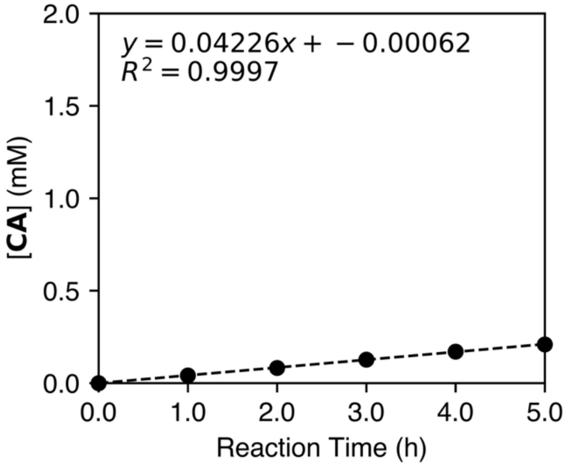

Figure S47. Determination of initial rate of **CA** formation for the aldol reaction catalyzed by 10 mol% **7**.

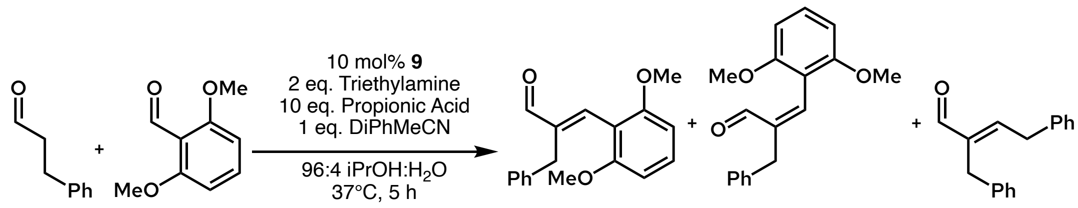

A_N (5 mM) 1.25 equiv. A_E (*E*)-CA (*Z*)-CA HA

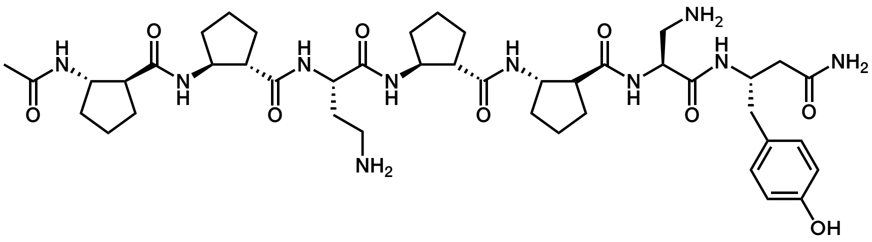

9

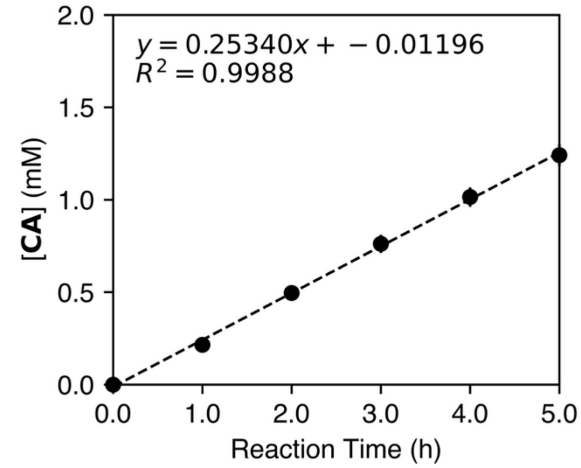

Figure S48. Determination of initial rate of **CA** formation for the aldol reaction catalyzed by 10 mol% **9**.

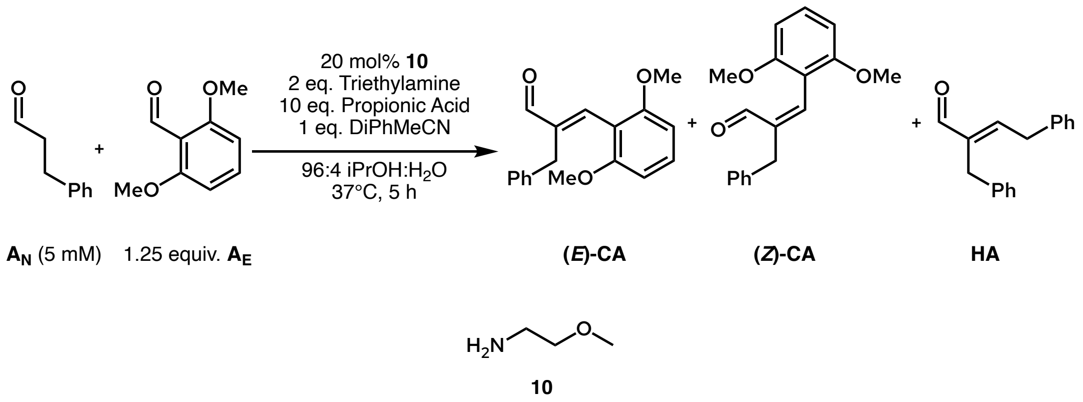

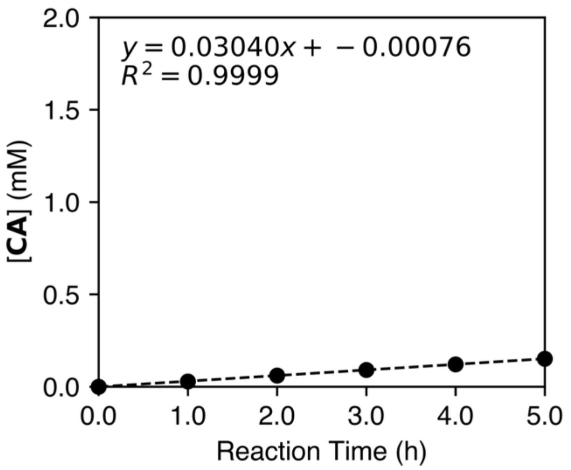

Figure S49. Determination of initial rate of **CA** formation for the aldol reaction catalyzed by 20 mol% **10**.

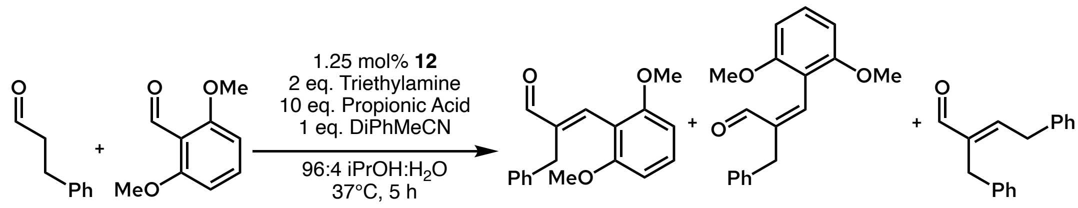

A_N (5 mM) 1.25 equiv. A_E (*E*)-**CA** (*Z*)-**CA** **HA**

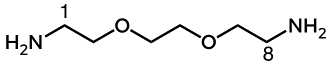

12

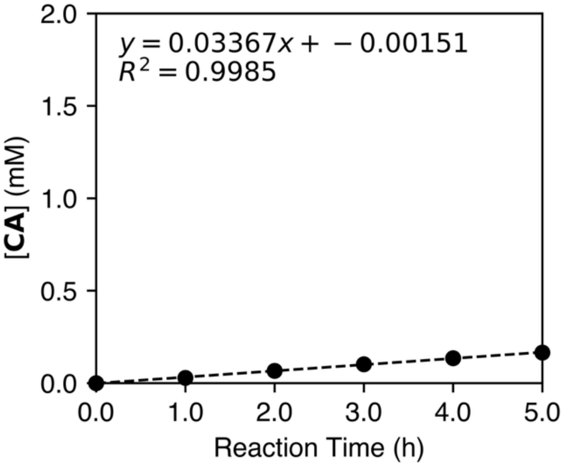

Figure S50. Determination of initial rate of **CA** formation for the aldol reaction catalyzed by 1.25 mol% **12**.

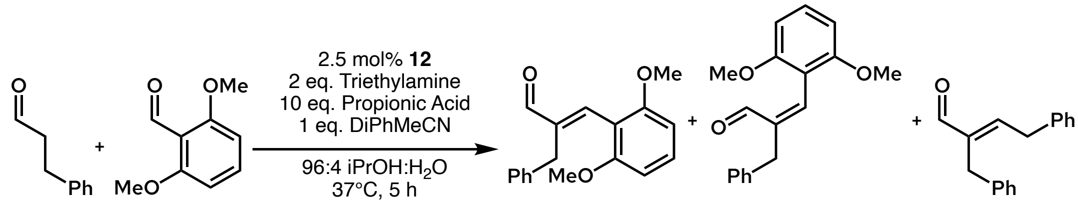

A_N (5 mM) 1.25 equiv. A_E (*E*)-CA (*Z*)-CA HA

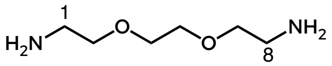

12

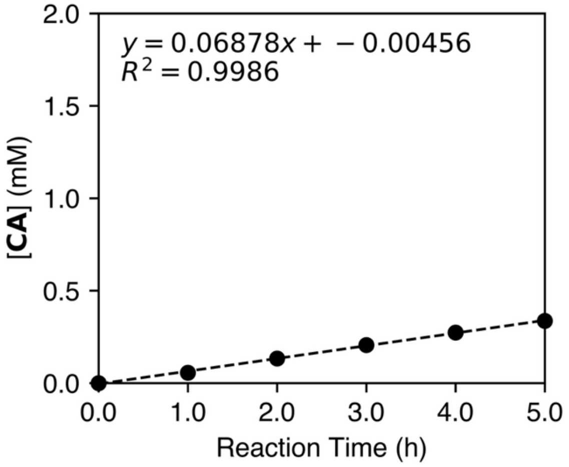

Figure S51. Determination of initial rate of **CA** formation for the aldol reaction catalyzed by 2.5 mol% **12**.

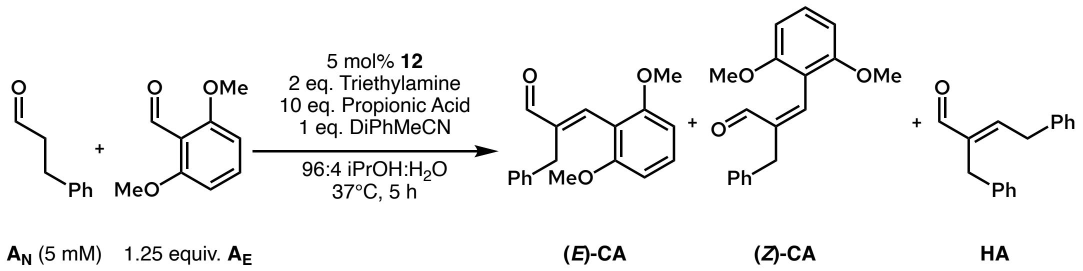

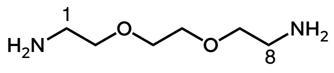

12

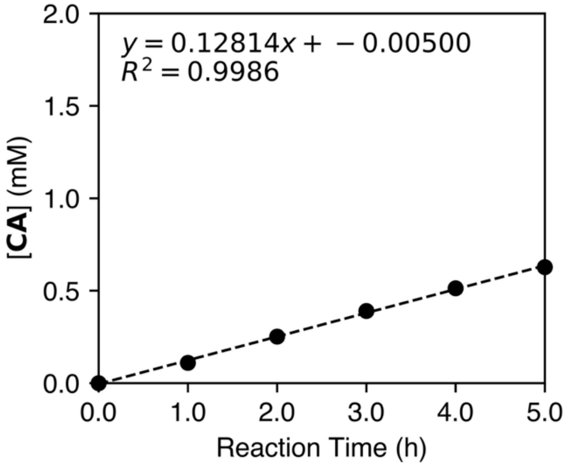

Figure S52. Determination of initial rate of **CA** formation for the aldol reaction catalyzed by 5 mol% **12**.

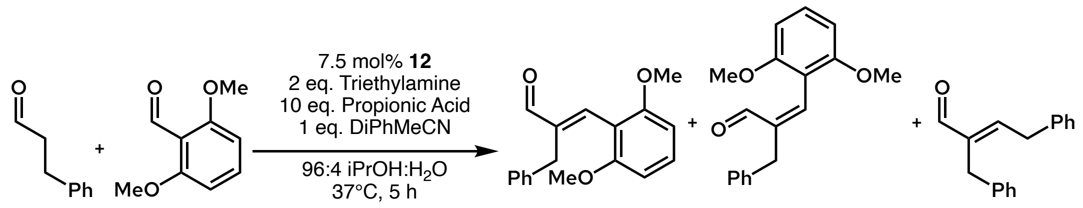

A_N (5 mM) 1.25 equiv. **A_E** **(*E*)-CA** **(*Z*)-CA** **HA**

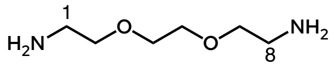

12

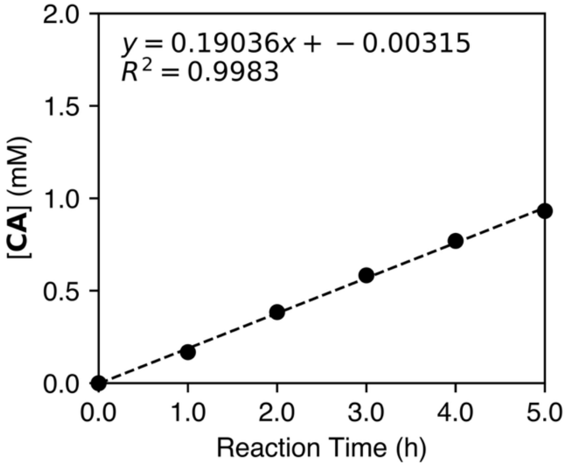

Figure S53. Determination of initial rate of **CA** formation for the aldol reaction catalyzed by 7.5 mol% **12**.

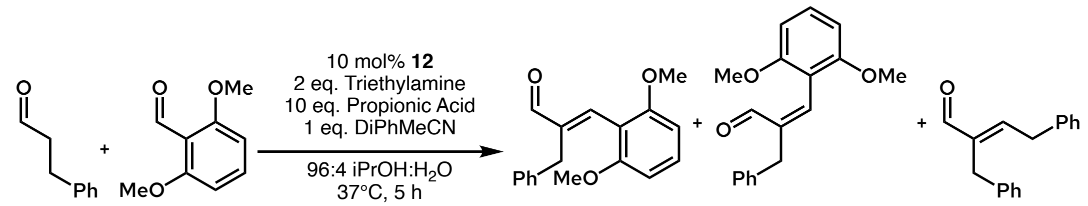

A_N (5 mM) 1.25 equiv. A_E (*E*)-CA (*Z*)-CA HA

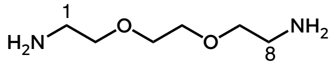

12

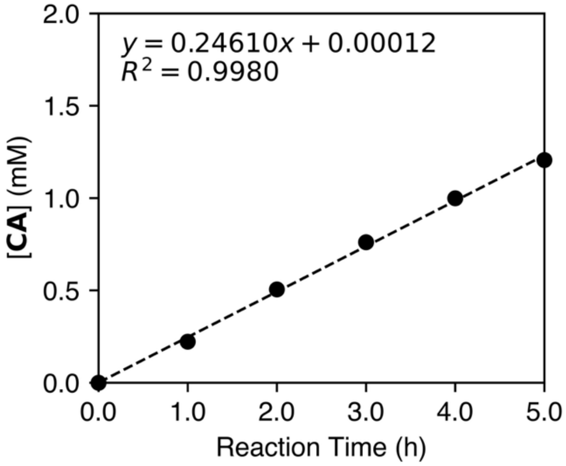

Figure S54. Determination of initial rate of **CA** formation for the aldol reaction catalyzed by 10 mol% **12**.

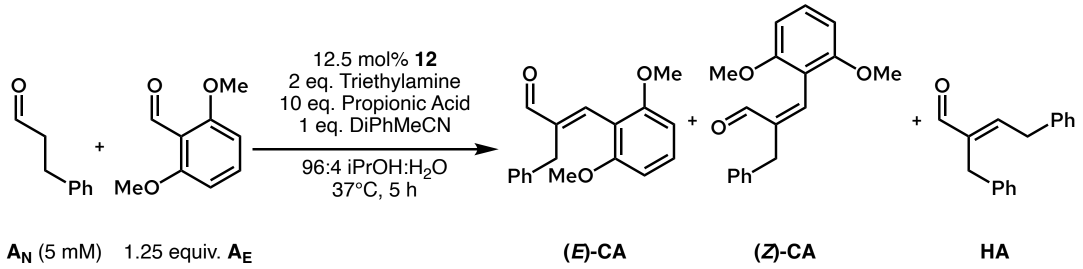

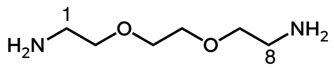

12

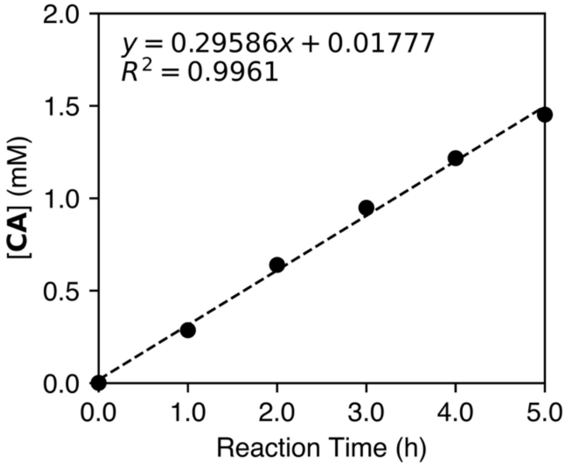

Figure S55. Determination of initial rate of **CA** formation for the aldol reaction catalyzed by 12.5 mol% **12**.

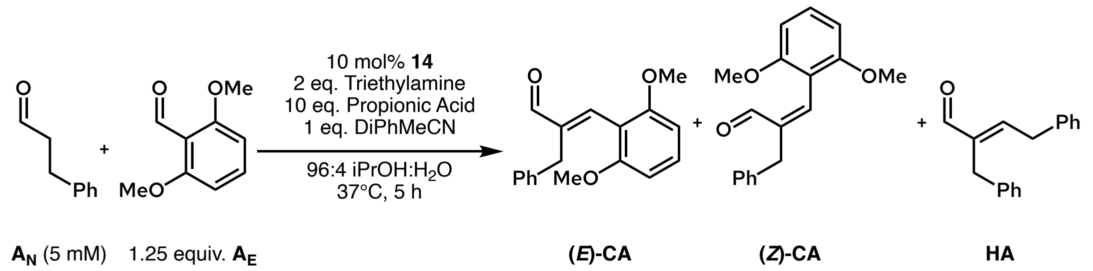

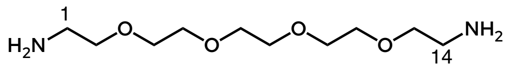

14

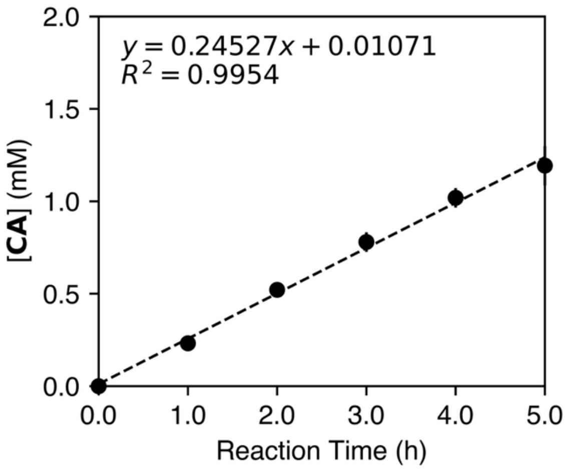

Figure S56. Determination of initial rate of **CA** formation for the aldol reaction catalyzed by 10 mol% **14**.

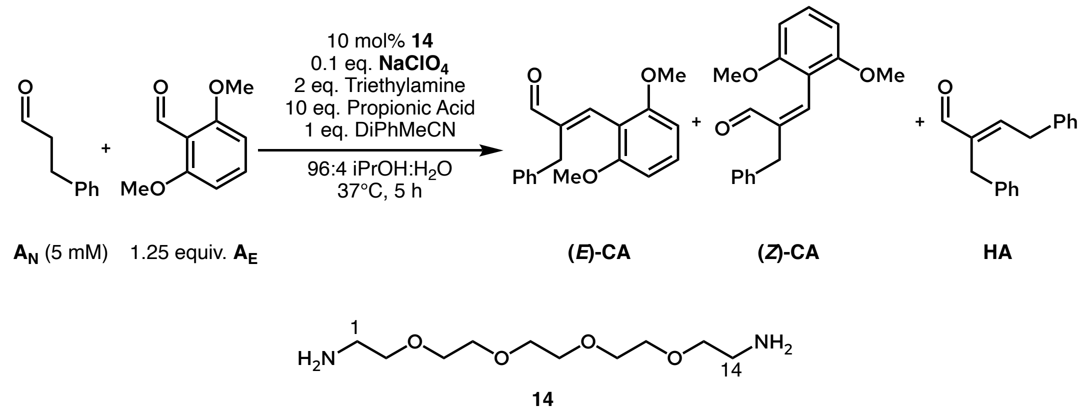

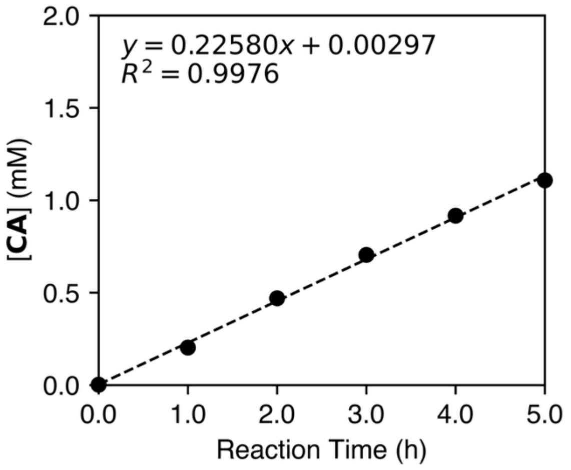

Figure S57. Determination of initial rate of **CA** formation for the aldol reaction catalyzed by 10 mol% **14** in the presence of 0.1 eq. $NaClO_4$.

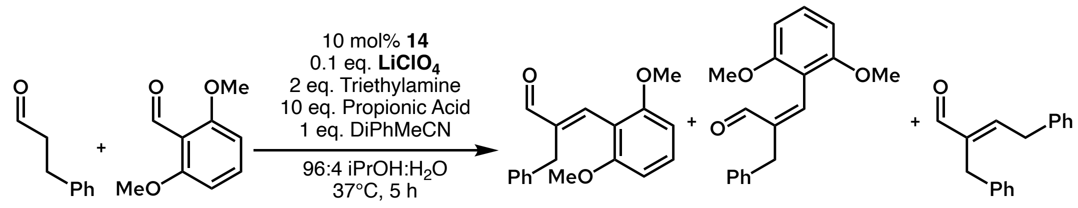

A_N (5 mM) 1.25 equiv. A_E **(*E*)-CA** **(*Z*)-CA** **HA**

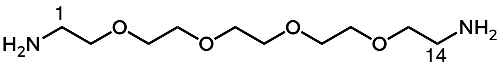

14

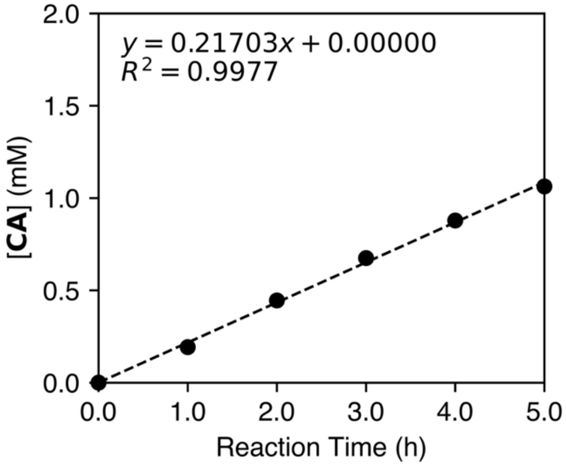

Figure S58. Determination of initial rate of **CA** formation for the aldol reaction catalyzed by 10 mol% **14** in the presence of 0.1 eq. $LiClO_4$.

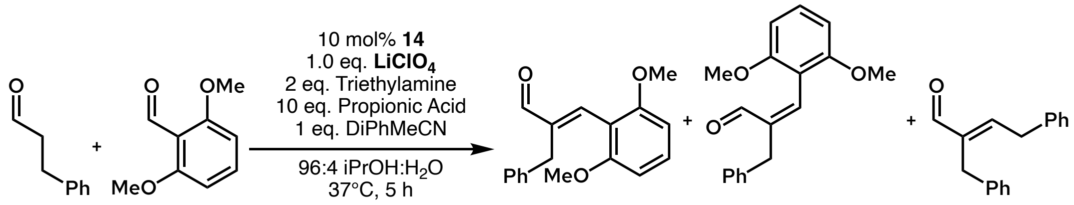

$\mathbf{A_N}$ (5 mM) 1.25 equiv. $\mathbf{A_E}$ **(*E*)-CA** **(*Z*)-CA** **HA**

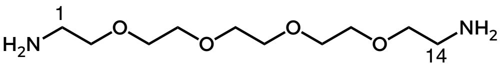

14

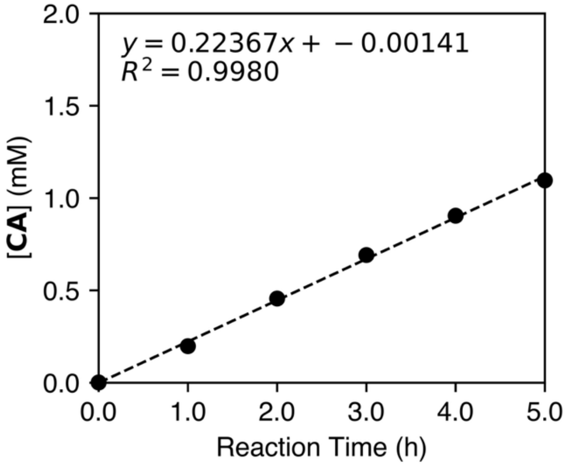

Figure S59. Determination of initial rate of **CA** formation for the aldol reaction catalyzed by 10 mol% **14** in the presence of 1.0 eq. $LiClO_4$.

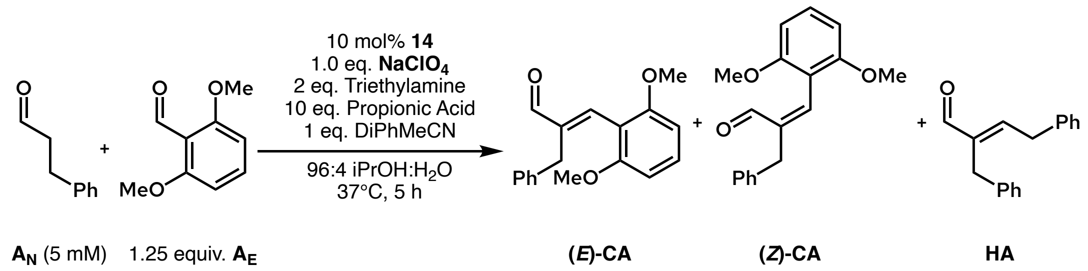

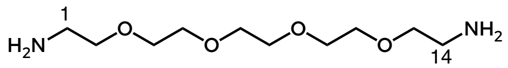

14

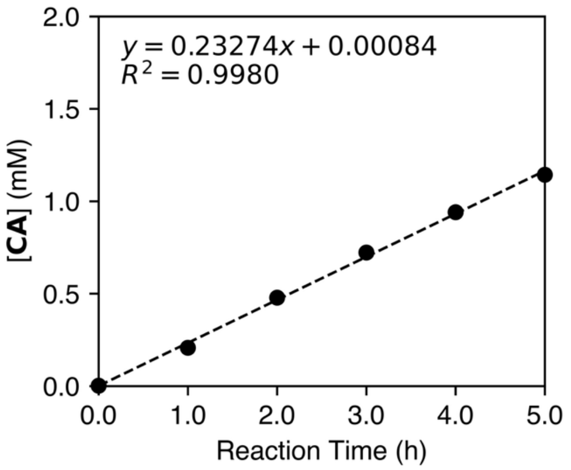

Figure S60. Determination of initial rate of **CA** formation for the aldol reaction catalyzed by 10 mol% **14** in the presence of 1.0 eq. $NaClO_4$.

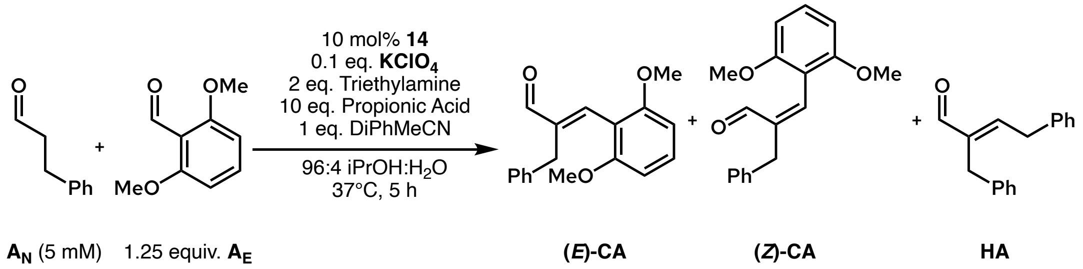

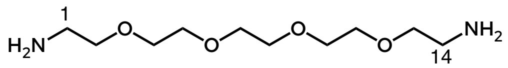

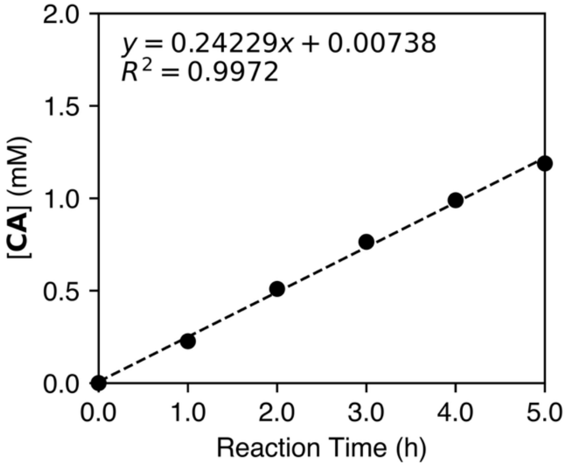

Figure S61. Determination of initial rate of **CA** formation for the aldol reaction catalyzed by 10 mol% **14** in the presence of 0.1 eq. $KClO_4$.

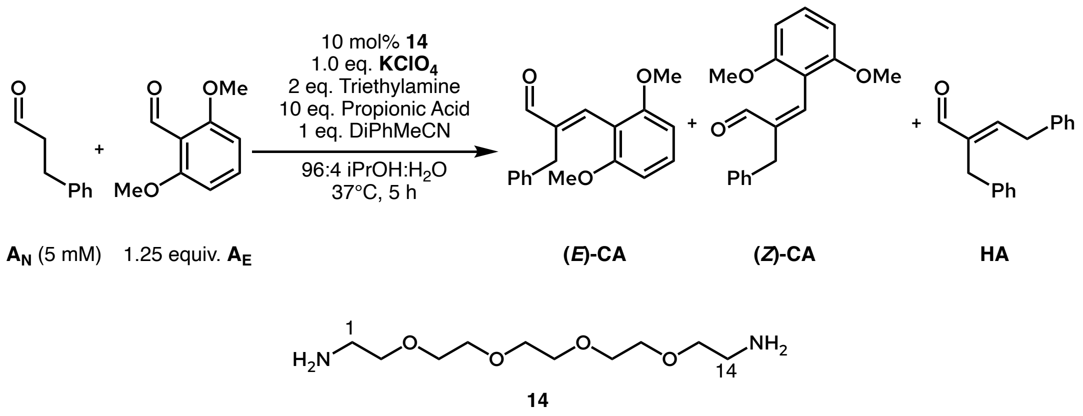

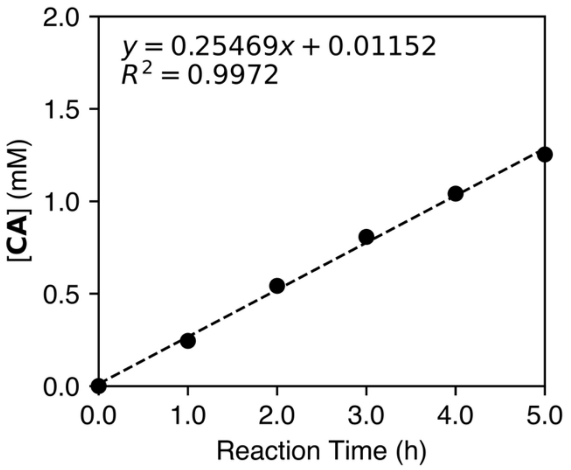

Figure S62. Determination of initial rate of **CA** formation for the aldol reaction catalyzed by 10 mol% **14** in the presence of 1.0 eq. $KClO_4$.

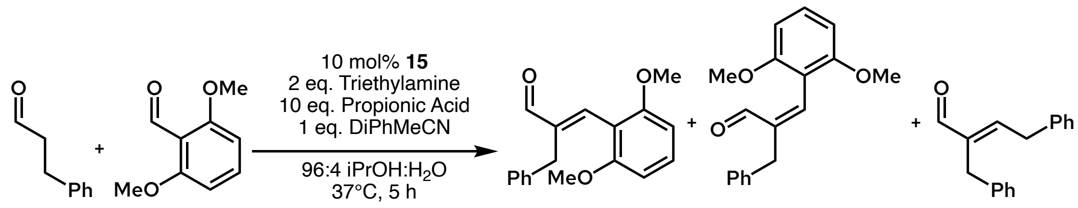

A_N (5 mM) 1.25 equiv. A_E (*E*)-**CA** (*Z*)-**CA** **HA**

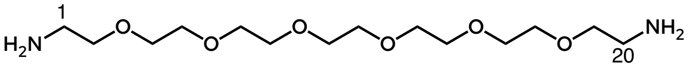

15

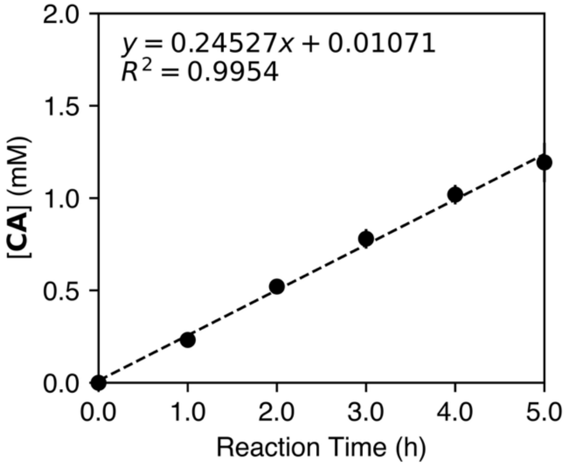

Figure S63. Determination of initial rate of **CA** formation for the aldol reaction catalyzed by 10 mol% **15**.

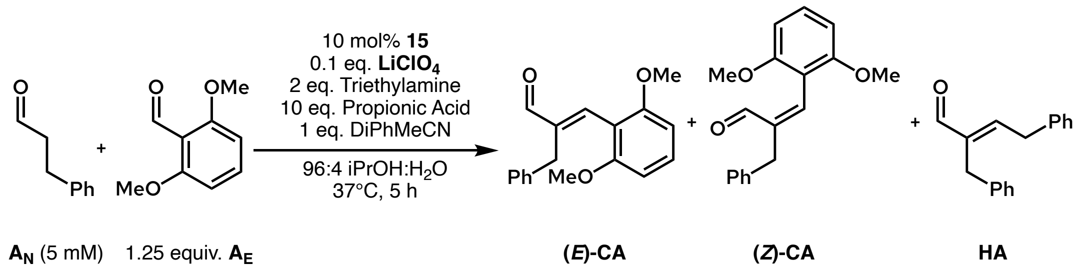

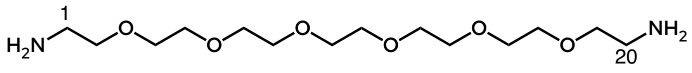

15

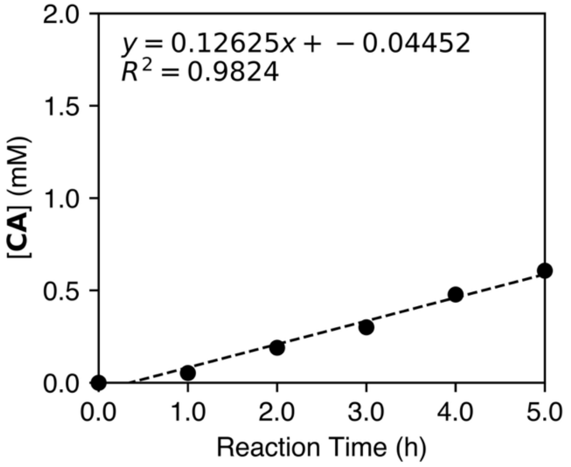

Figure S64. Determination of initial rate of **CA** formation for the aldol reaction catalyzed by 10 mol% **15** in the presence of 0.1 eq. $LiClO_4$.

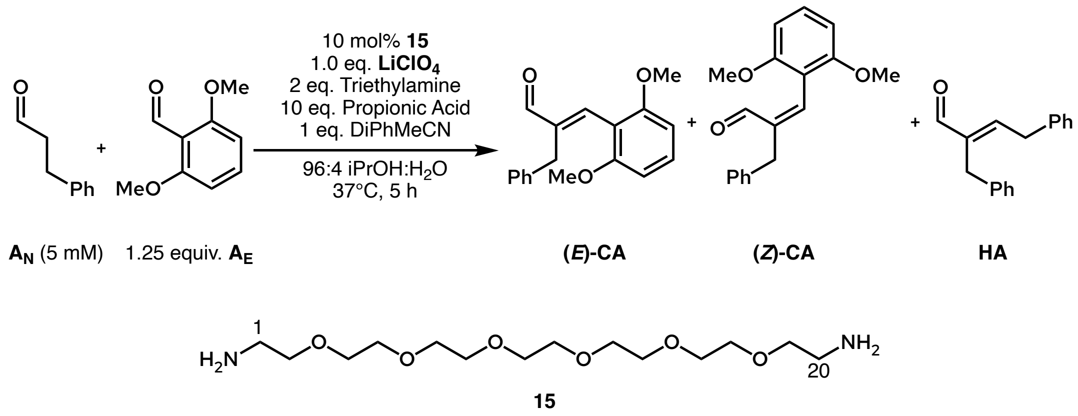

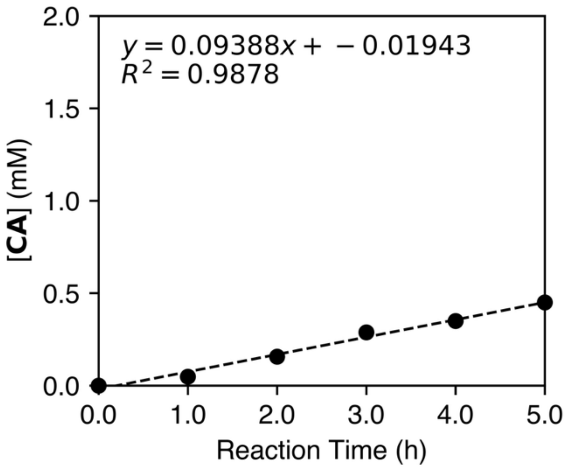

Figure S65. Determination of initial rate of **CA** formation for the aldol reaction catalyzed by 10 mol% **15** in the presence of 1.0 eq. $LiClO_4$.

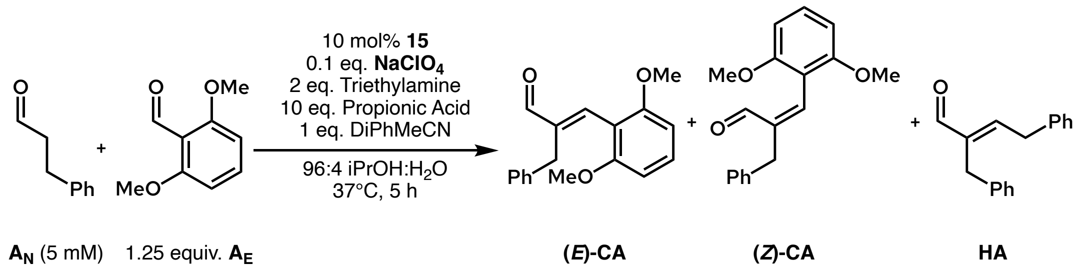

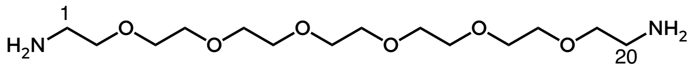

15

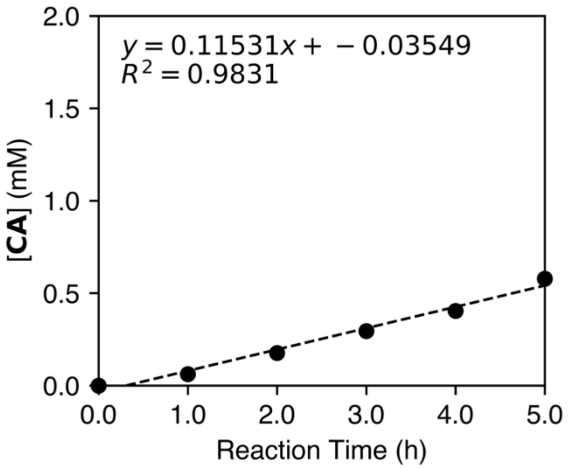

Figure S66. Determination of initial rate of **CA** formation for the aldol reaction catalyzed by 10 mol% **15** in the presence of 0.1 eq. $NaClO_4$.

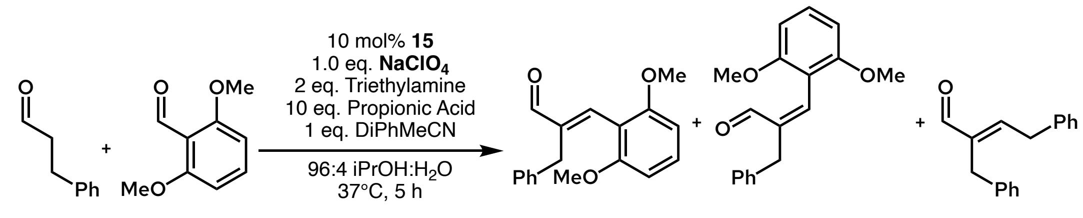

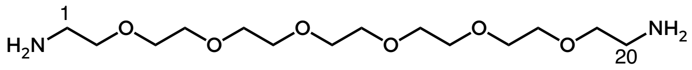

15

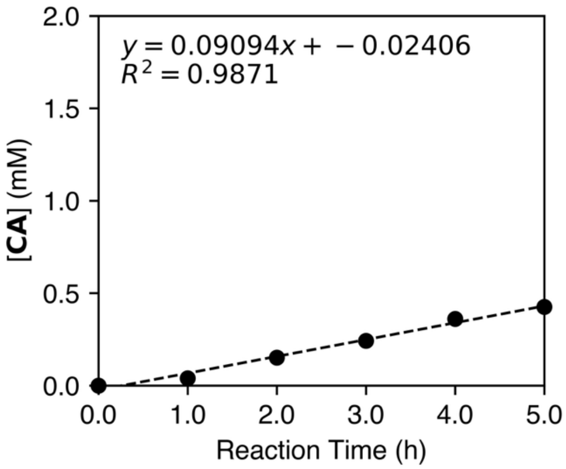

Figure S67. Determination of initial rate of **CA** formation for the aldol reaction catalyzed by 10 mol% **15** in the presence of 1.0 eq. $NaClO_4$.

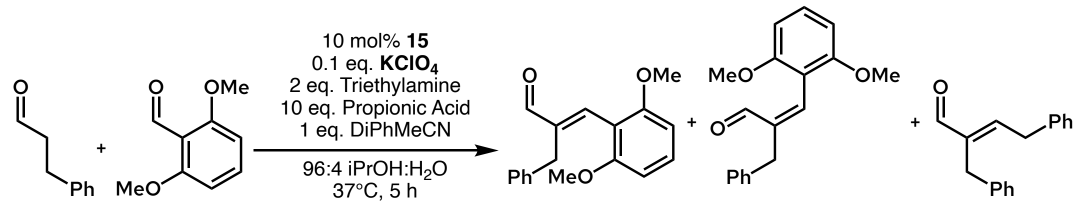

A_N (5 mM) 1.25 equiv. A_E (*E*)-CA (*Z*)-CA HA

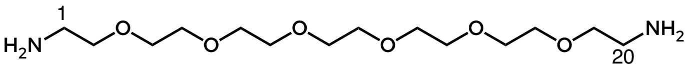

15

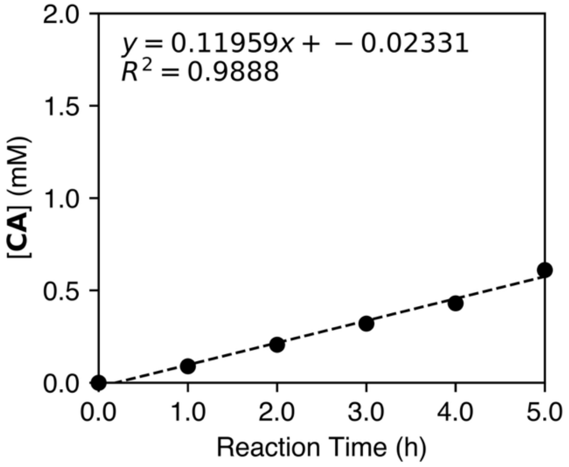

Figure S68. Determination of initial rate of **CA** formation for the aldol reaction catalyzed by 10 mol% **15** in the presence of 0.1 eq. $KClO_4$.

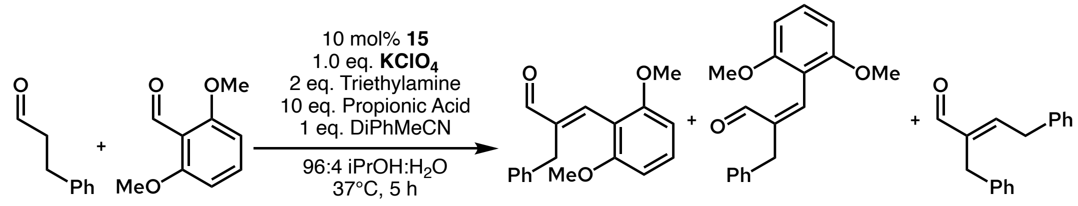

A_N (5 mM) 1.25 equiv. A_E (*E*)-CA (*Z*)-CA HA

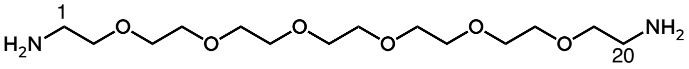

15

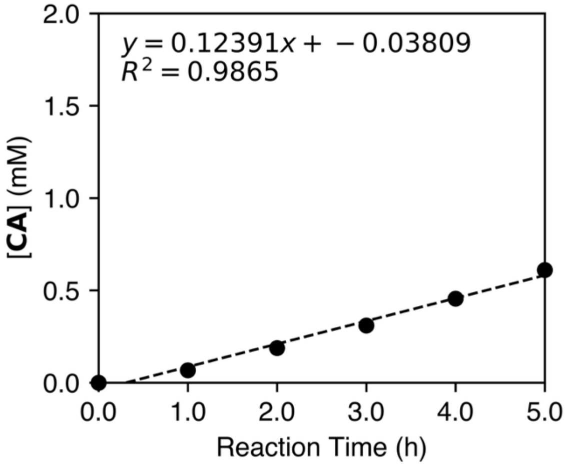

Figure S69. Determination of initial rate of **CA** formation for the aldol reaction catalyzed by 10 mol% **15** in the presence of 1.0 eq. $KClO_4$.

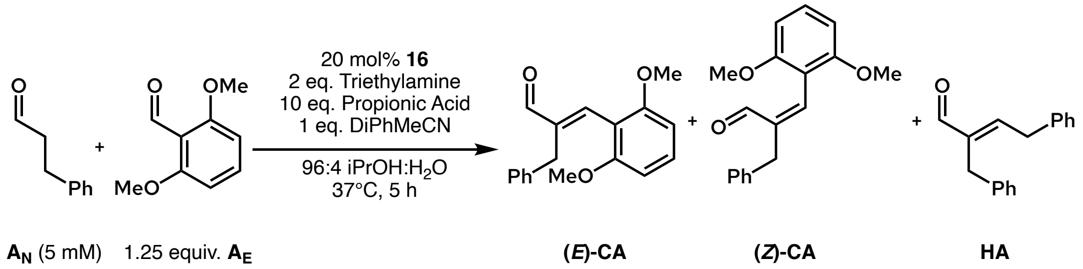

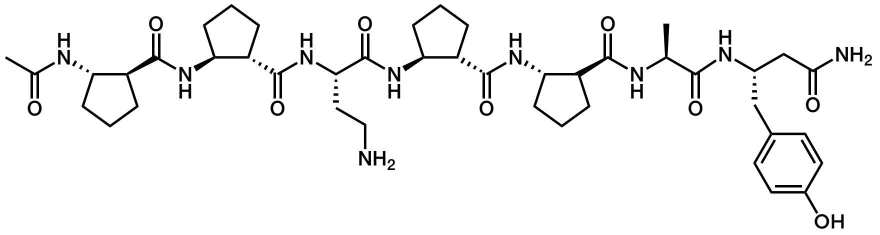

16

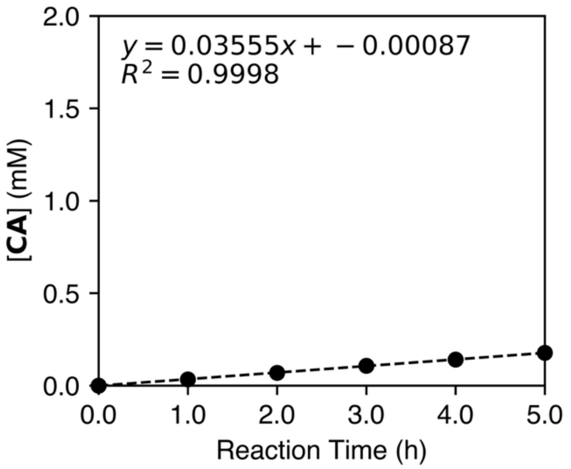

Figure S70. Determination of initial rate of **CA** formation for the aldol reaction catalyzed by 20 mol% **16**.

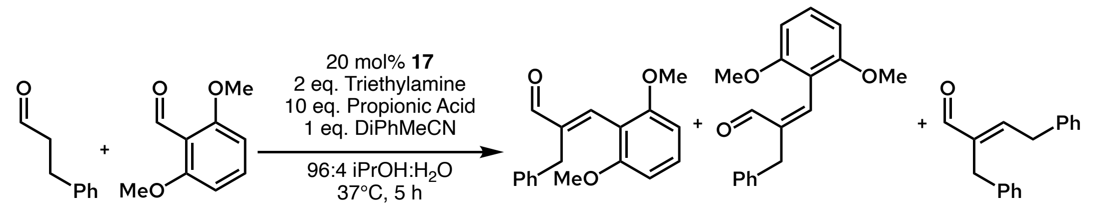

AN (5 mM) 1.25 equiv. **A**E (*E*)-CA (*Z*)-CA HA

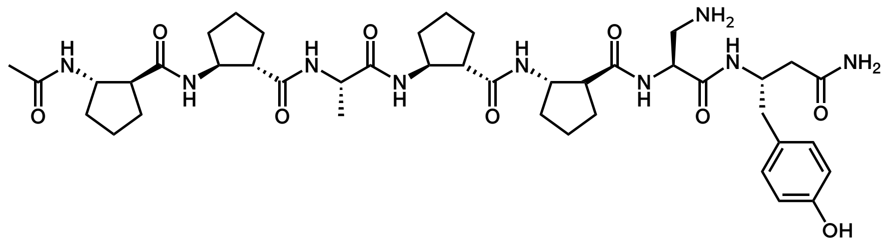

17

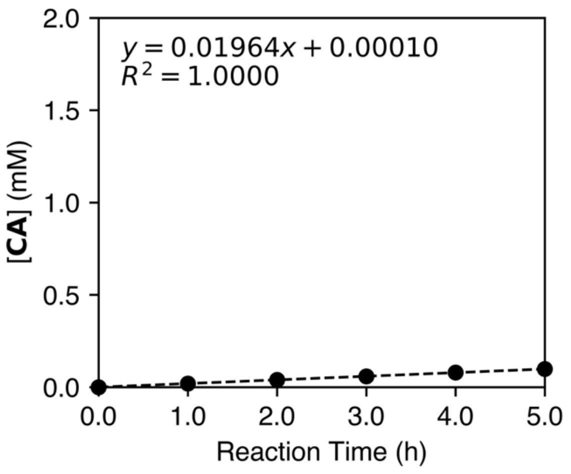

Figure S71. Determination of initial rate of **CA** formation for the aldol reaction catalyzed by 20 mol% **17**.

6. Computational Information

6.1 General Conformational Information

Initial geometries for all conformers were generated using RDKit.[5] DFT computations were conducted using Gaussian 16.[6] Optimizations and vibrational frequency calculations were carried out at the B3LYP/6-31G(d,p)/(SMD=iPrOH) and M06-2X/def2-TZVP/(SMD=iPrOH) levels.[7-11] Conformers with negative vibrational modes were excluded from the datasets. Conformers were visualized using CYLview20.[12] Jupyter notebooks used for conformer generation are available on GitHub. XYZ coordinates, energetics data and geometric parameters for all optimized conformers of **4'** and **12'** at both levels of theory are provided in a separate spreadsheet in the GitHub repository for this work. Tether dihedrals were defined as being in the *anti* configuration when the angle measured is ±180° ± 30°.[13] Tether dihedrals were defined as being in the *gauche* configuration when the angle is ±120° ± 30°.[13] Reported C1 to C13 distances are through space.

6.2 Conformer Ensemble Generation and Optimization Procedure

RDKit was used to generate 300,000 conformers (150,000 per iminium configurational isomer) of diamines **4'** and **12'** with acetaldehyde-derived iminium and enamine moieties on the scaffold. The distance between C1 and C13, distance between C4 and C11, the number of *anti* dihedral angles in the tether, the number of *gauche* dihedral angles in the tether, the dihedral angle between atoms C1-C2-N3-C4 and the dihedral angle between atoms C10-C11-N12-C13 were determined for each conformer. Using these parameters, ensembles of ~1100 representative conformers (~550 species per iminium configurational isomer) for **4'** and **12'** were selected for further optimization. The geometries of all conformers in each ensemble were optimized at the B3LYP/6-31G(d,p)/(SMD=iPrOH) and M06-2X/def2-TZVP/(SMD=iPrOH) levels. Vibrational frequency calculations were conducted on each optimized conformer geometry to confirm the conformers had no negative vibrational modes. Conformers with negative vibrational modes were removed from the ensembles. Geometric parameters reported in sections 6.3 to 6.6 of this document were determined using the optimized conformer ensembles for diamines **4'** and **12'**.

At both levels of theory, conformers of **4'** with fully extended tethers (all dihedrals in the *anti* configuration) were favored. For **12'** evaluated at the B3LYP/6-31G(d,p)/(SMD=iPrOH) level, conformers with 1 to 5 tether dihedrals in the *anti* configuration were found to have similar energies. When assessed at the M06-2X/def-2TZVP/(SMD=iPrOH) level, **12'** exhibited an energetic preference for conformers with 2 tether dihedrals in the *anti* configuration. Conformer energies determined at the M06-2X/def2-TZVP level are more accurate than those determined at the lower B3LYP/6-31G(d,p) level.[14]

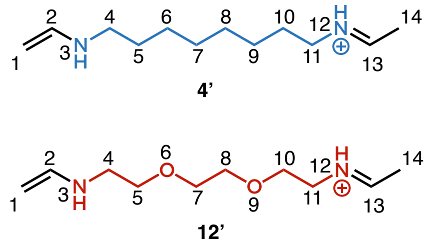

Figure S72. Atom numbering scheme for **4'** and **12'**.

6.3 Boltzmann distributions of conformers of **4’** and **12’** – M06-2X/def2-TZVP

Boltzmann distributions were plotted for conformers of **4’** and **12’** optimized at the M06-2X/def2-TZVP level of theory with relative free energies less than 5 kcal/mol. The total number of conformers included in the Boltzmann distribution for **4’** was 630. The total number of conformers included in the Boltzmann distribution of **12’** was 425. C1-C13 distances were directly plotted against individual conformer population % or binned into 0.5 Å groups from 2 - 15 Å.

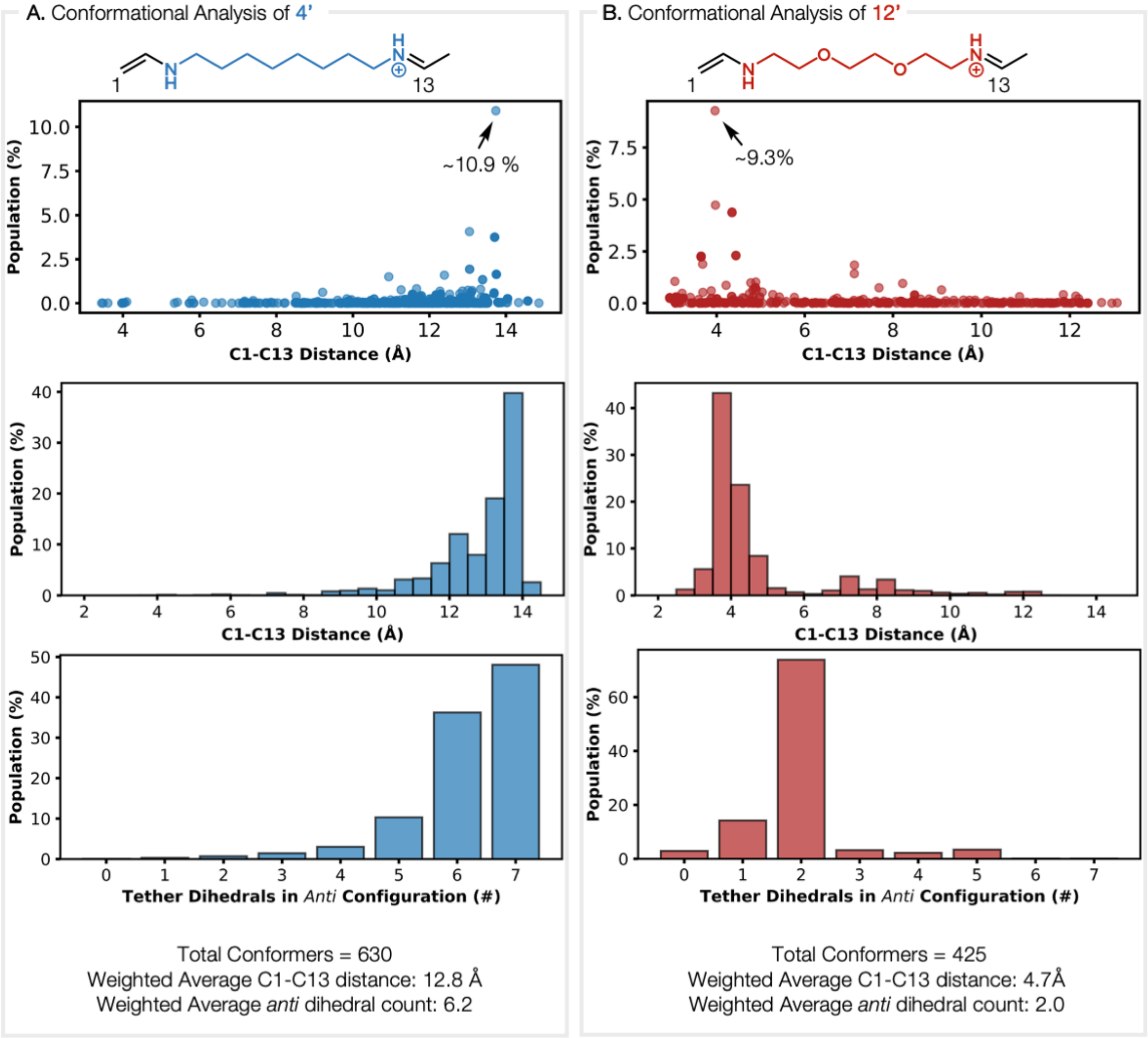

Figure S73. Populations of conformers of (A) **4’** and (B) **12’** as a function of C1-C13 distance (directly plotted or binned into 0.5 Å groupings) or number of tether dihedrals in the *Anti* configuration.

6.4 DFT-derived Geometric Parameters for **6'** – B3LYP/6-31G(d,p)

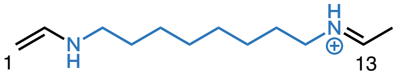

4'

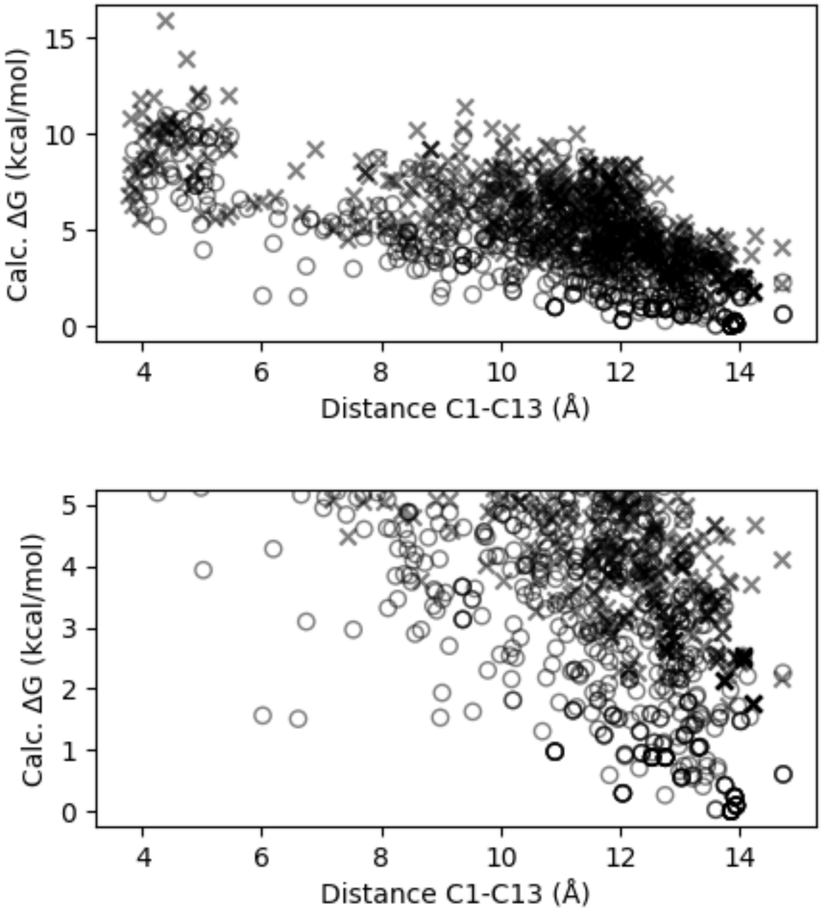

Figure S74. C1-C13 distances vs. calculated ΔG for conformers of **4'** optimized at the B3LYP/6-31G(d,p)/(SMD=iPrOH) level.

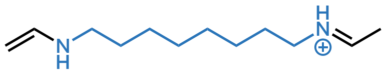

4’

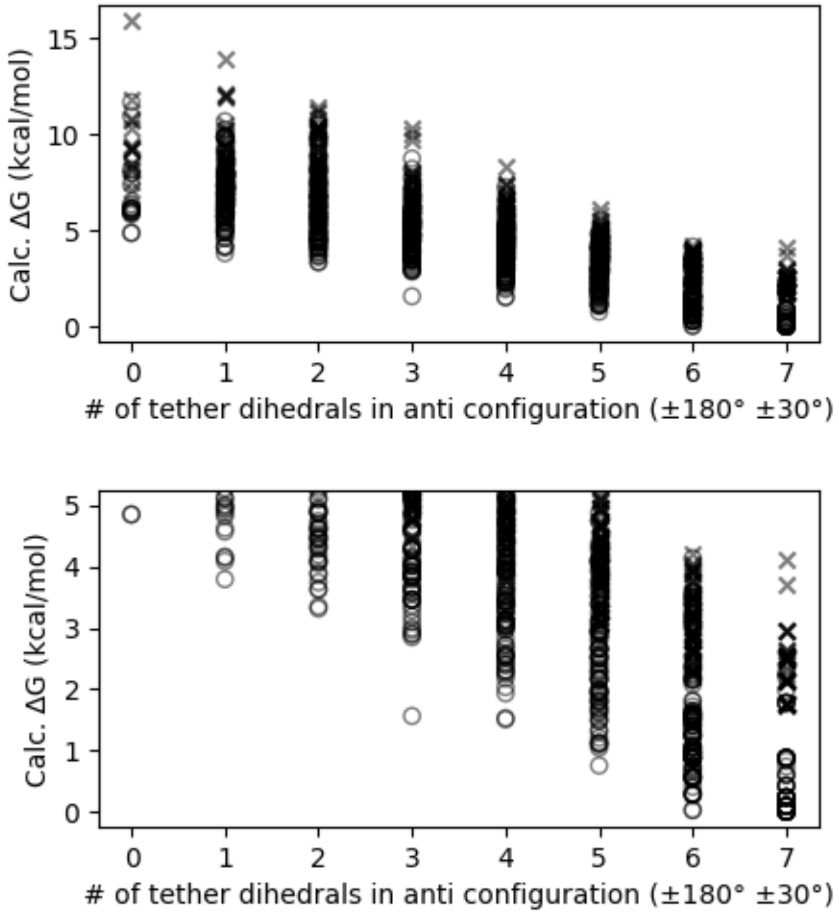

Figure S75. Number of tether dihedrals in *Anti* configuration vs. calculated ΔG for conformers of **4’** optimized at the B3LYP/6-31G(d,p)/(SMD=iPrOH) level.

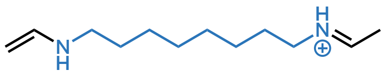

4’

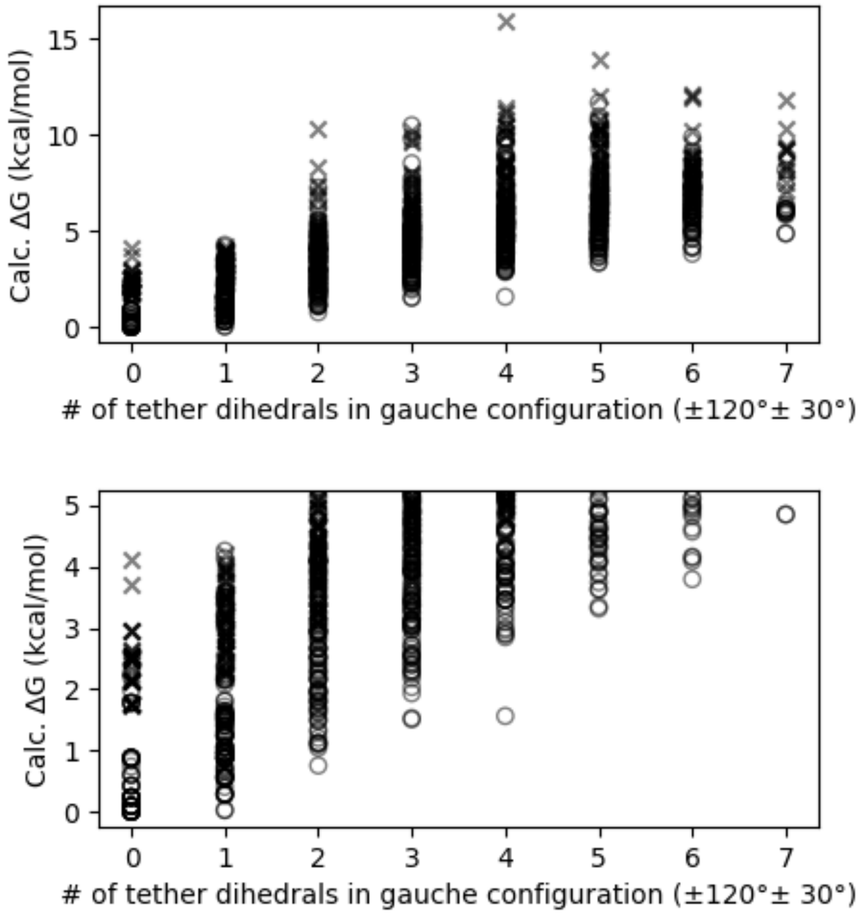

Figure S76. Number of tether dihedrals in *gauche* configuration vs. calculated ΔG for conformers of **4’** optimized at the B3LYP/6-31G(d,p)/(SMD=iPrOH) level. O markers = conformers with *E* iminiums. X markers = conformers with *Z* iminiums.

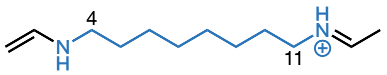

4’

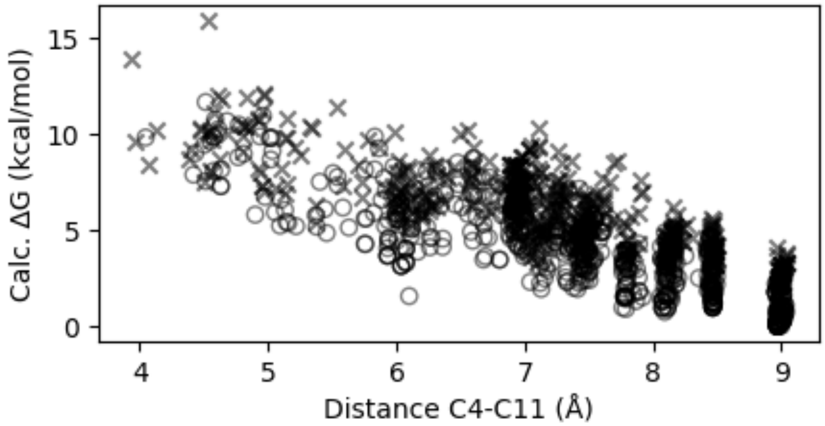

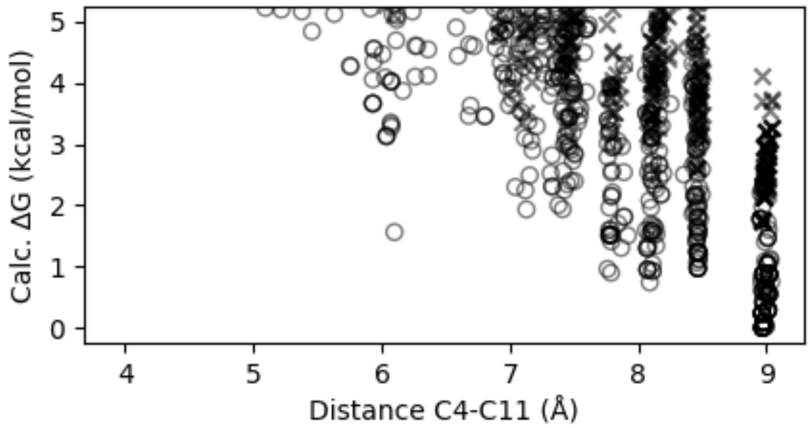

Figure S77. C4-C11 distances vs. calculated ΔG for conformers of **4’** optimized at the B3LYP/6-31G(d,p)/(SMD=iPrOH) level. O markers = conformers with *E* iminiums. X markers = conformers with *Z* iminiums.

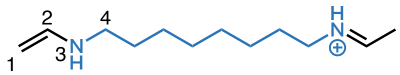

4’

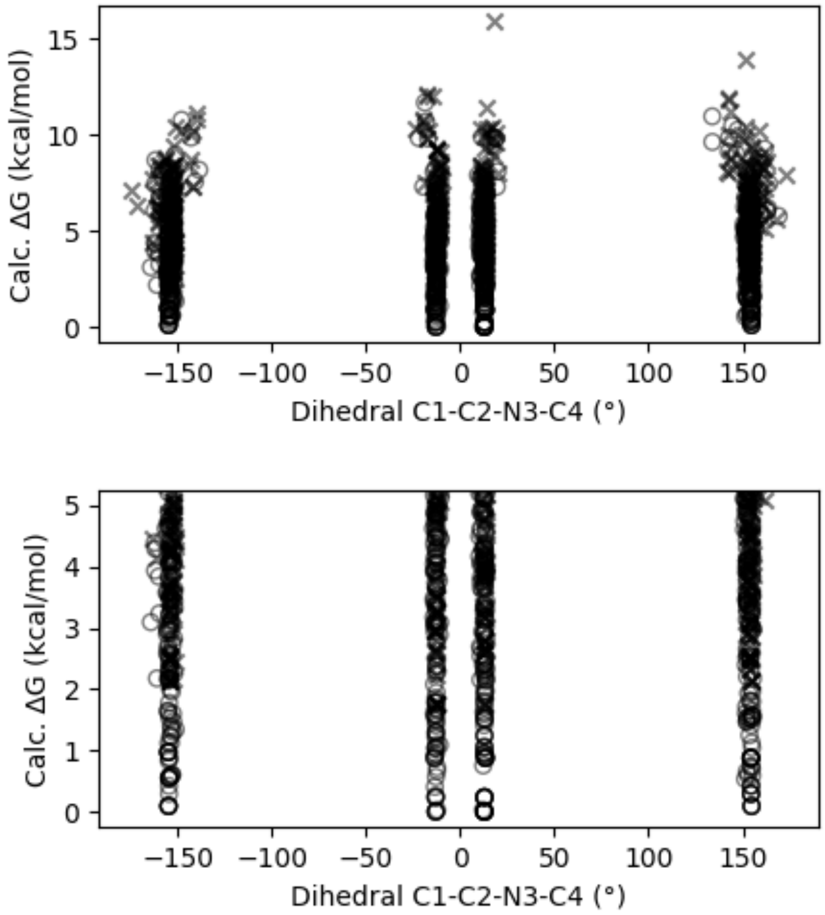

Figure S78. C1-C2-N3-C4 dihedral angles vs. calculated ΔG for conformers of **4’** optimized at the B3LYP/6-31G(d,p)/(SMD=iPrOH) level. O markers = conformers with *E* iminiums. X markers = conformers with *Z* iminiums.

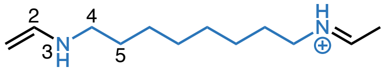

4’

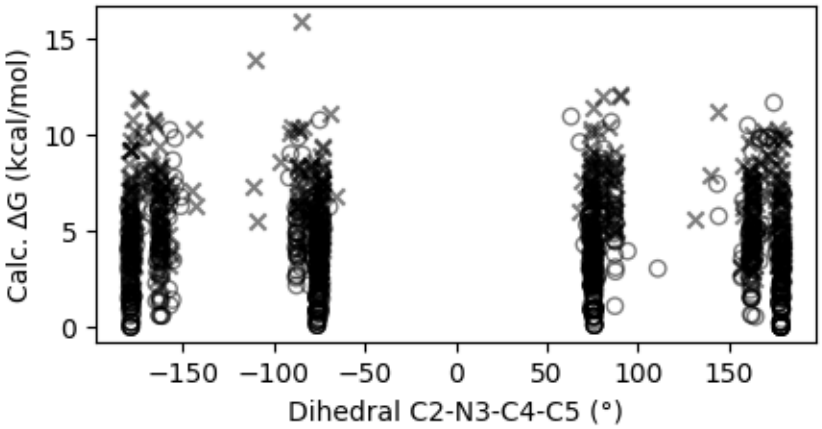

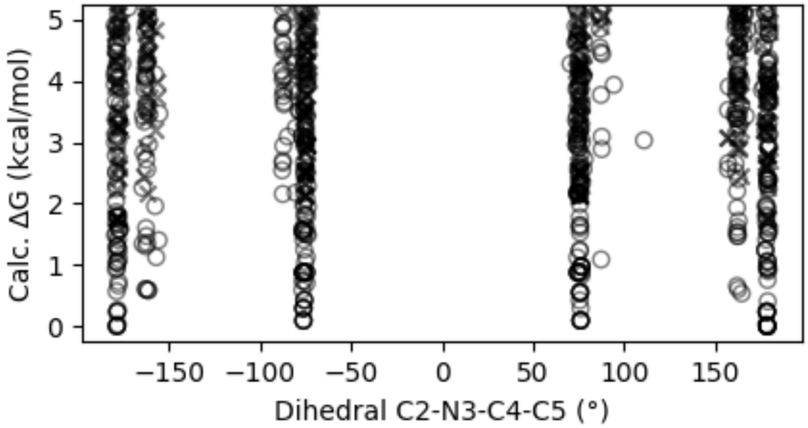

Figure S79. C2-N3-C4-C5 dihedral angles vs. calculated ΔG for conformers of **4’** optimized at the B3LYP/6-31G(d,p)/(SMD=iPrOH) level. O markers = conformers with *E* iminiums. X markers = conformers with *Z* iminiums.

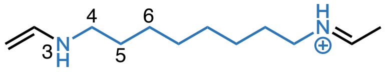

4’

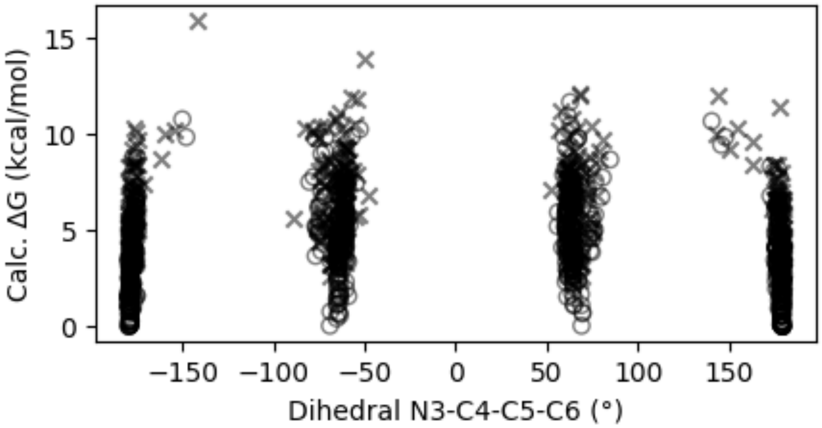

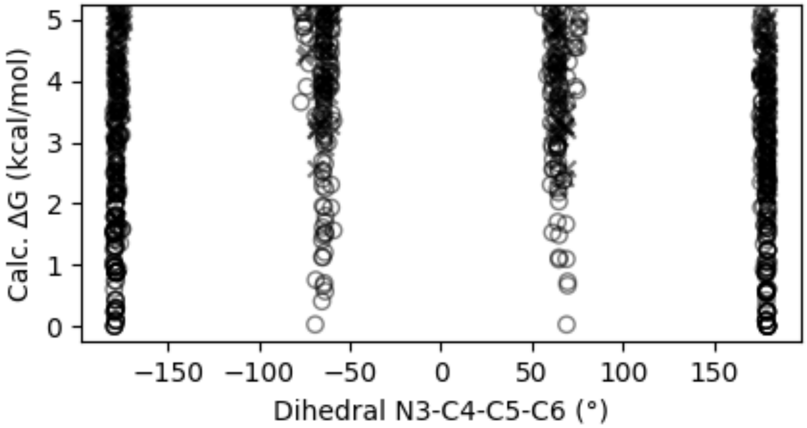

Figure S80. N3-C4-C5-C6 dihedral angles vs. calculated ΔG for conformers of **4’** optimized at the B3LYP/6-31G(d,p)/(SMD=iPrOH) level. O markers = conformers with *E* iminiums. X markers = conformers with *Z* iminiums.

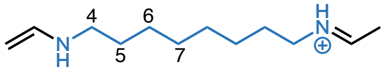

4’

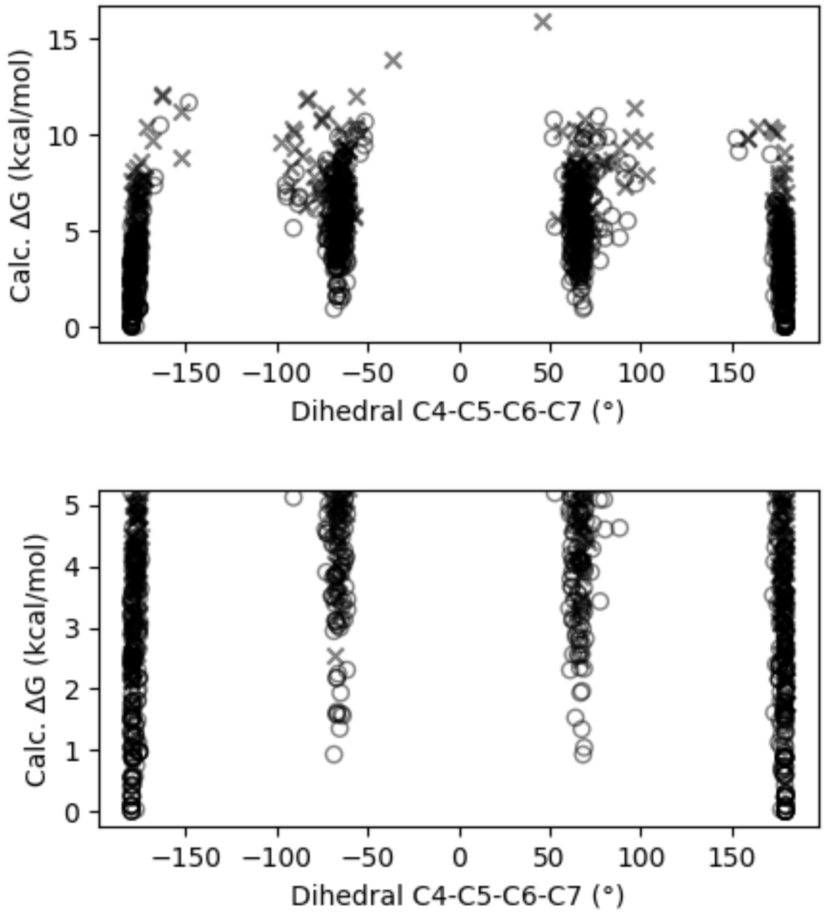

Figure S81. C4-C5-C6-C7 dihedral angles vs. calculated ΔG for conformers of **4’** optimized at the B3LYP/6-31G(d,p)/(SMD=iPrOH) level. O markers = conformers with *E* iminiums. X markers = conformers with *Z* iminiums.

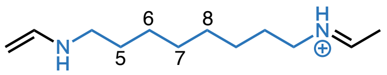

4’

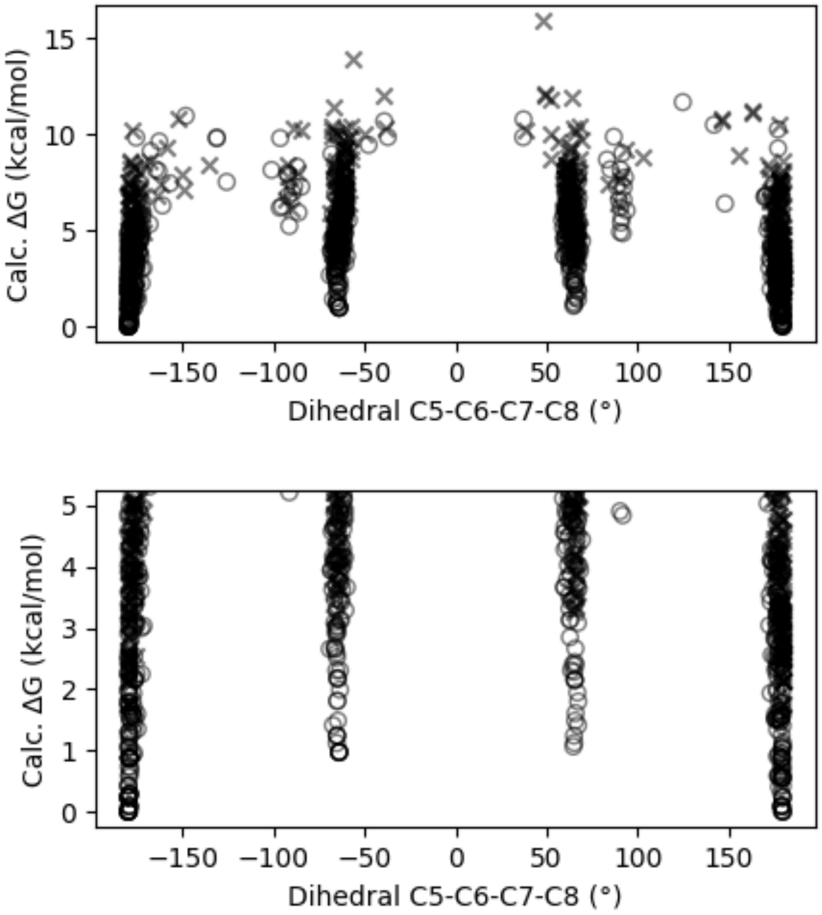

Figure S82. C5-C6-C7-C8 dihedral angles vs. calculated ΔG for conformers of **4’** optimized at the B3LYP/6-31G(d,p)/(SMD=iPrOH) level. O markers = conformers with *E* iminiums. X markers = conformers with *Z* iminiums.

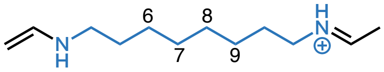

4’

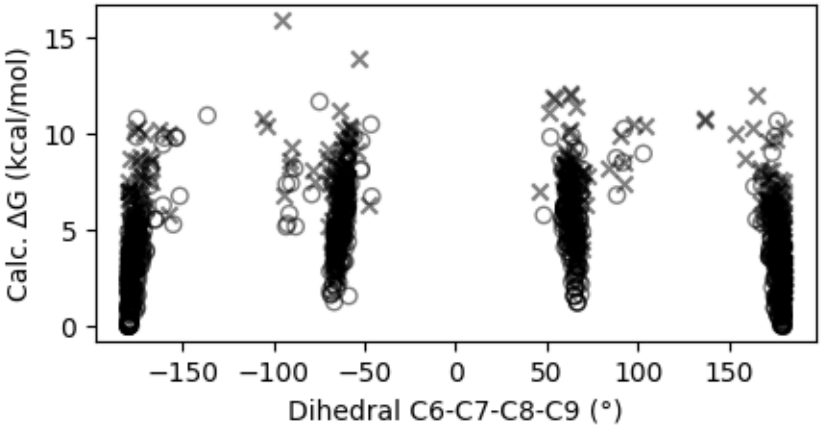

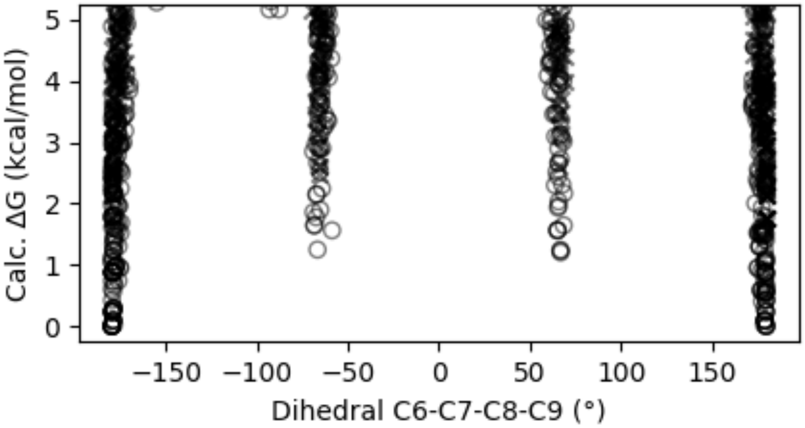

Figure S83. C6-C7-C8-C9 dihedral angles vs. calculated ΔG for conformers of **4’** optimized at the B3LYP/6-31G(d,p)/(SMD=iPrOH) level. O markers = conformers with *E* iminiums. X markers = conformers with *Z* iminiums.

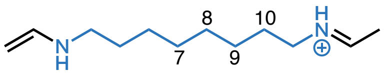

4’

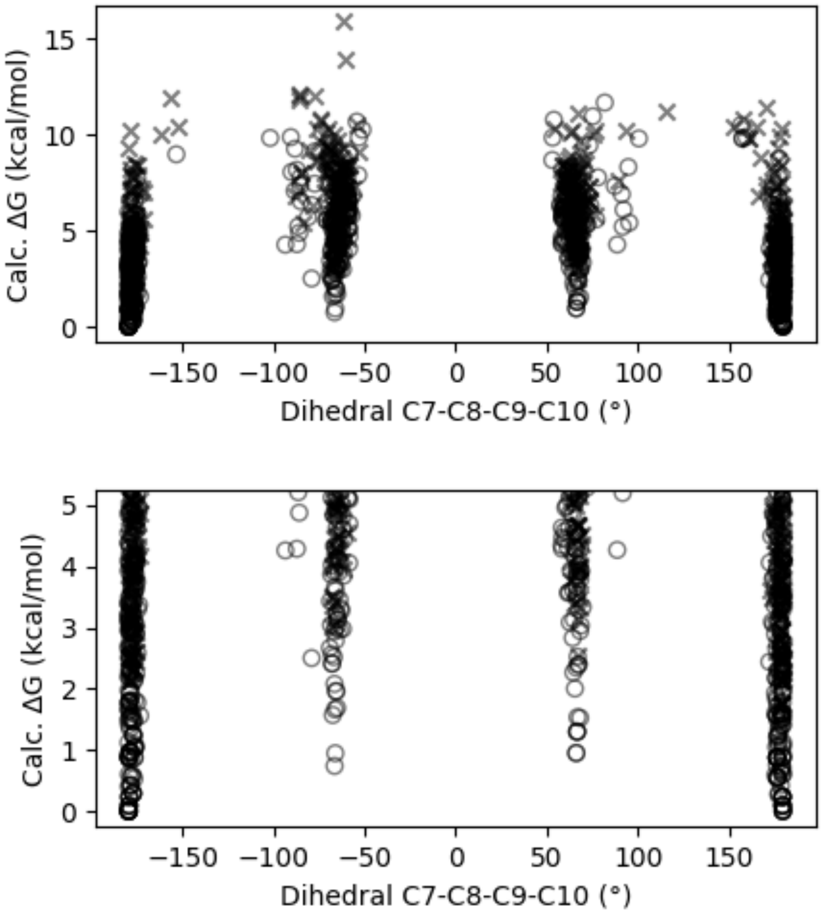

Figure S84. C7-C8-C9-C10 dihedral angles vs. calculated ΔG for conformers of **4’** optimized at the B3LYP/6-31G(d,p)/(SMD=iPrOH) level. O markers = conformers with *E* iminiums. X markers = conformers with *Z* iminiums.

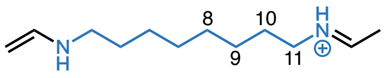

4’

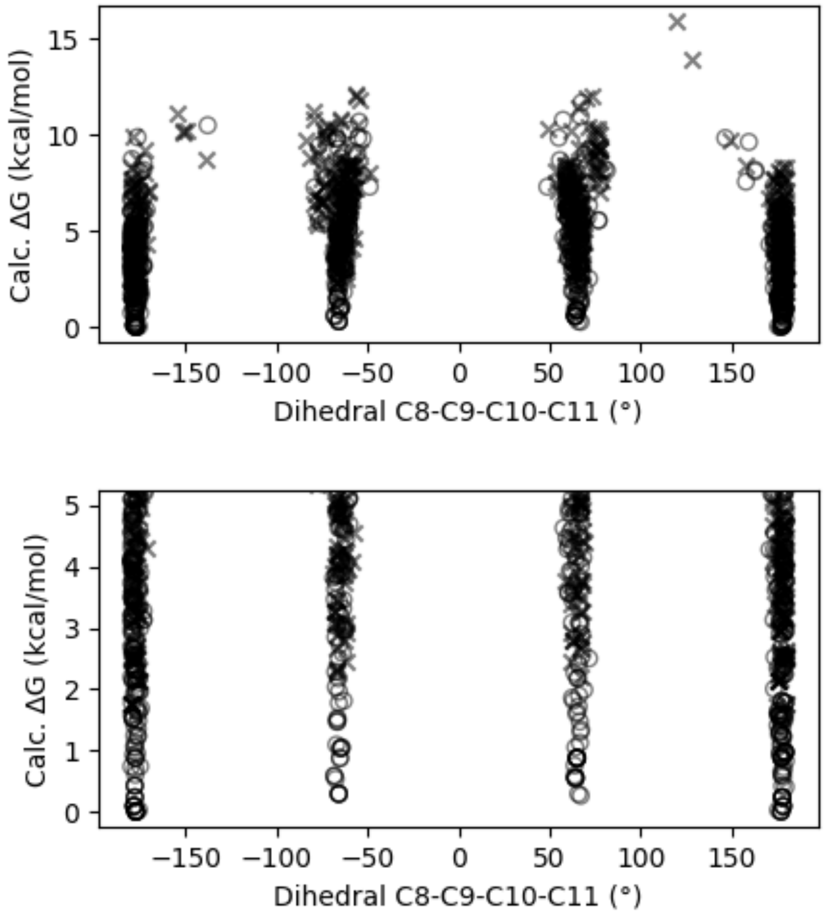

Figure S85. C8-C9-C10-C11 dihedral angles vs. calculated ΔG for conformers of **4’** optimized at the B3LYP/6-31G(d,p)/(SMD=iPrOH) level. O markers = conformers with *E* iminiums. X markers = conformers with *Z* iminiums.

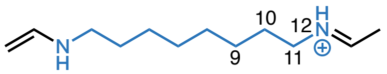

4’

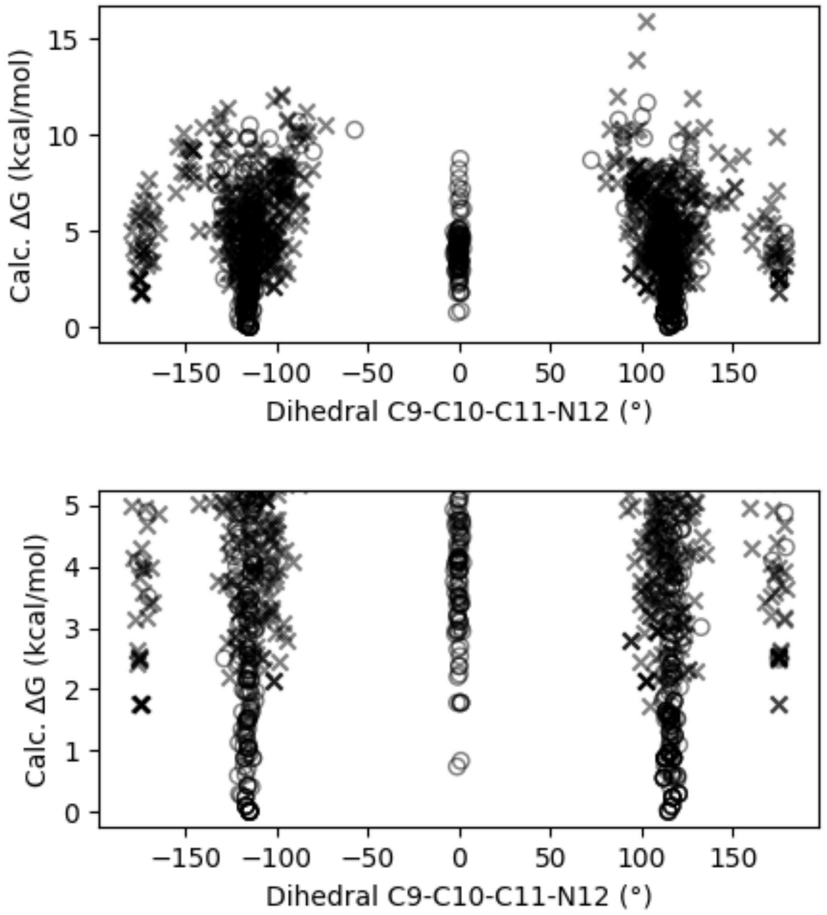

Figure S86. C9-C10-C11-N12 dihedral angles vs. calculated ΔG for conformers of **4’** optimized at the B3LYP/6-31G(d,p)/(SMD=iPrOH) level. O markers = conformers with *E* iminiums. X markers = conformers with *Z* iminiums.

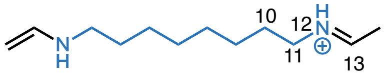

4’

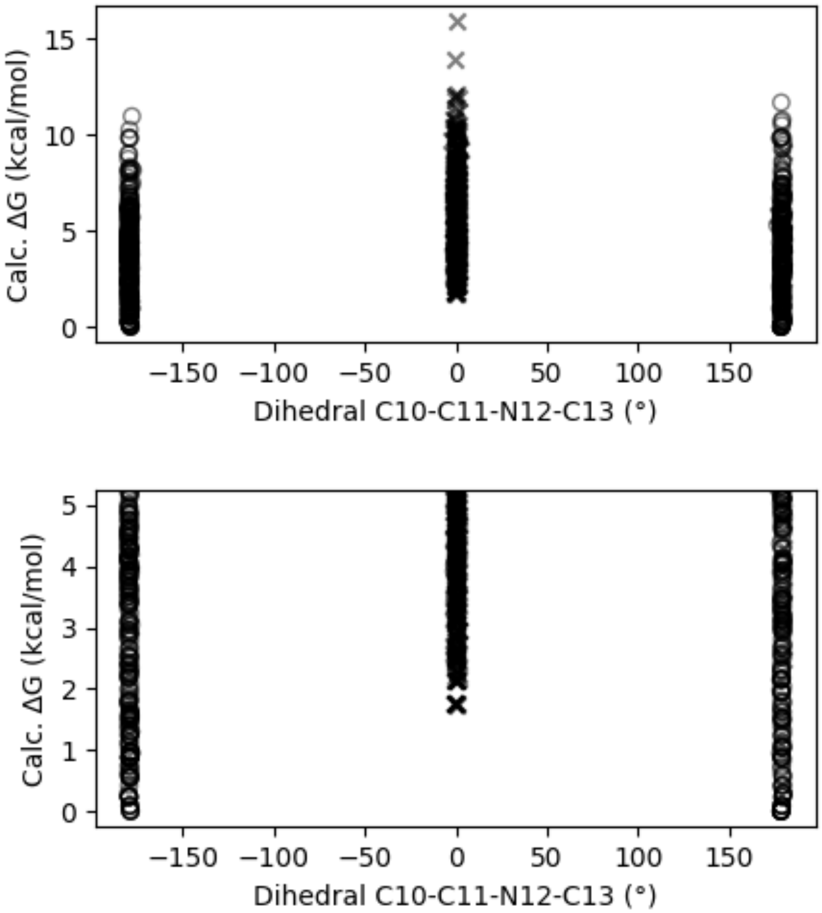

Figure S87. C10-C11-N12-C13 dihedral angles vs. calculated ΔG for conformers of **4’** optimized at the B3LYP/6-31G(d,p)/(SMD=iPrOH) level. O markers = conformers with *E* iminiums. X markers = conformers with *Z* iminiums.

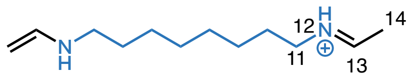

4'

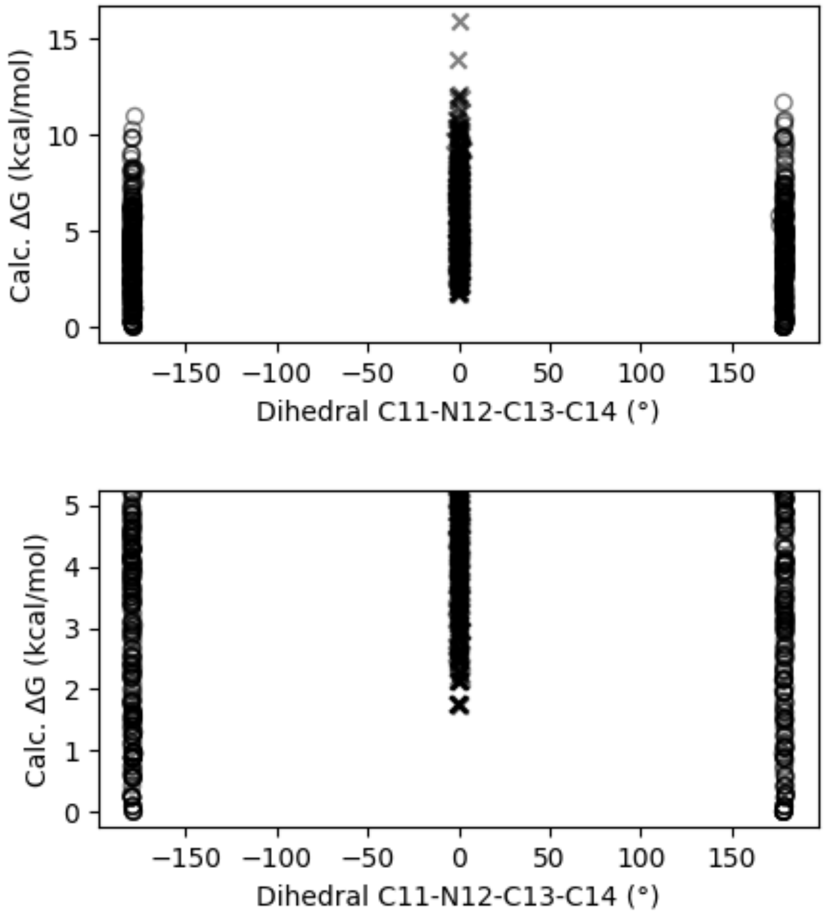

Figure S88. C11-N12-C13-C14 dihedral angles vs. calculated ΔG for conformers of **4'** optimized at the B3LYP/6-31G(d,p)/(SMD=iPrOH) level. O markers = conformers with *E* iminiums. X markers = conformers with *Z* iminiums.

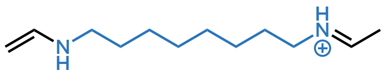

4’

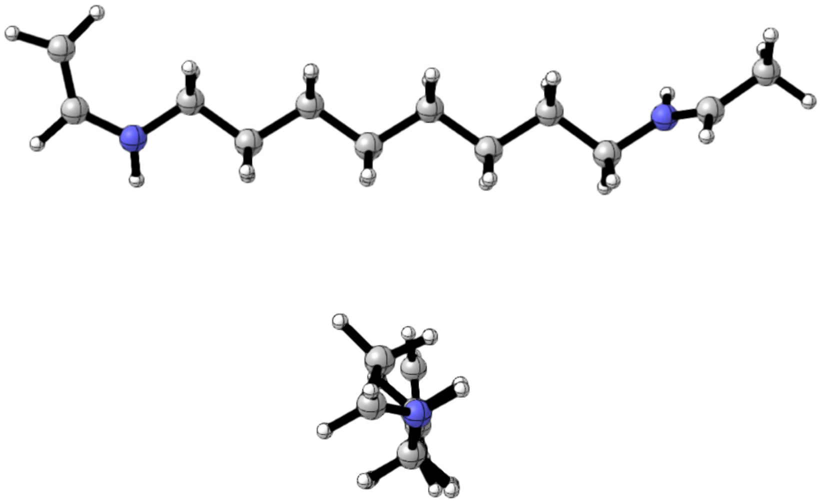

Figure S89. Front and side view of the lowest free energy conformer of **4’** at the B3LYP/6-31G(d,p)/(SMD=iPrOH) level.

6.5 DFT-derived Geometric Parameters for **6’** – M06-2X/def2-TZVP

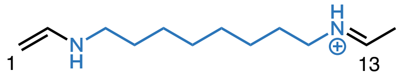

4’

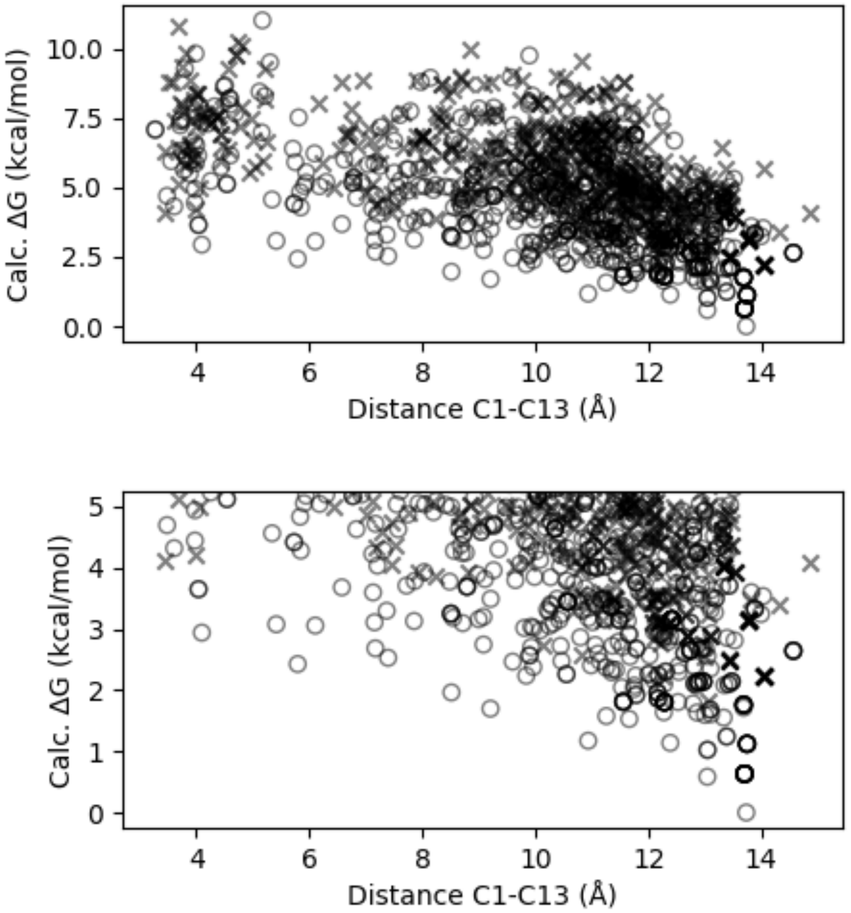

Figure S90. C1-C13 distances vs. calculated ΔG for conformers of **4’** optimized at the M06-2X/def2-TZVP/(SMD=iPrOH) level.

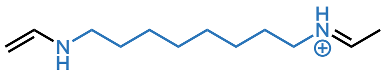

4’

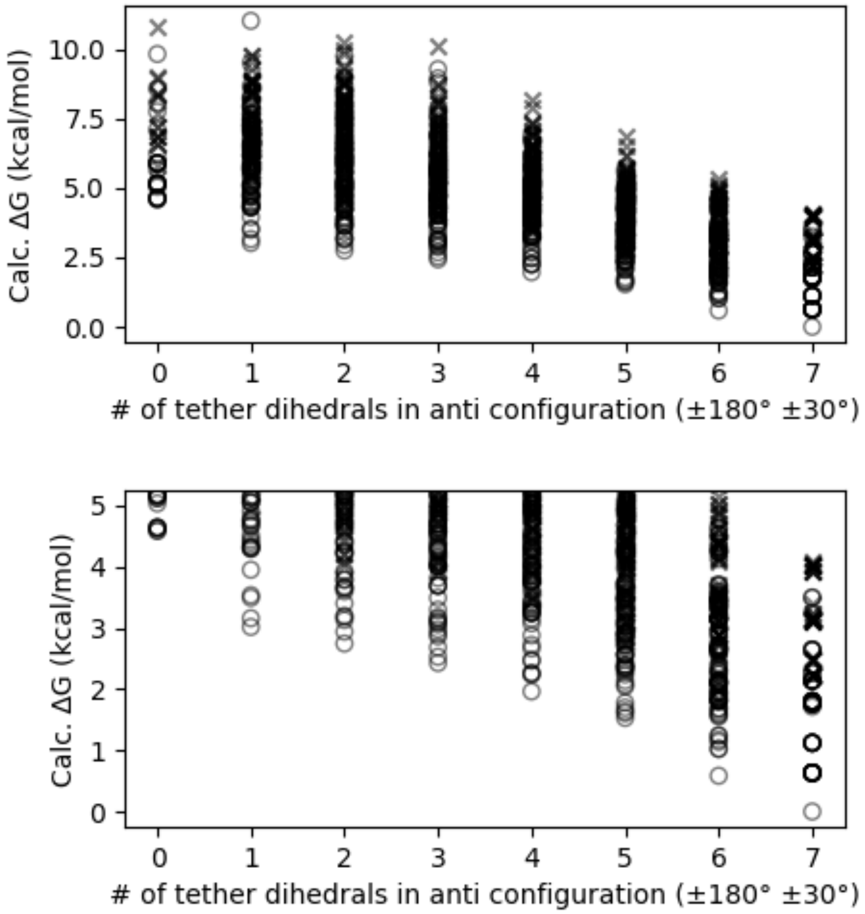

Figure S91. Number of tether dihedrals in *Anti* configuration vs. calculated ΔG for conformers of **4’** optimized at the M06-2X/def2-TZVP/(SMD=iPrOH) level.

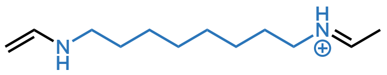

4'

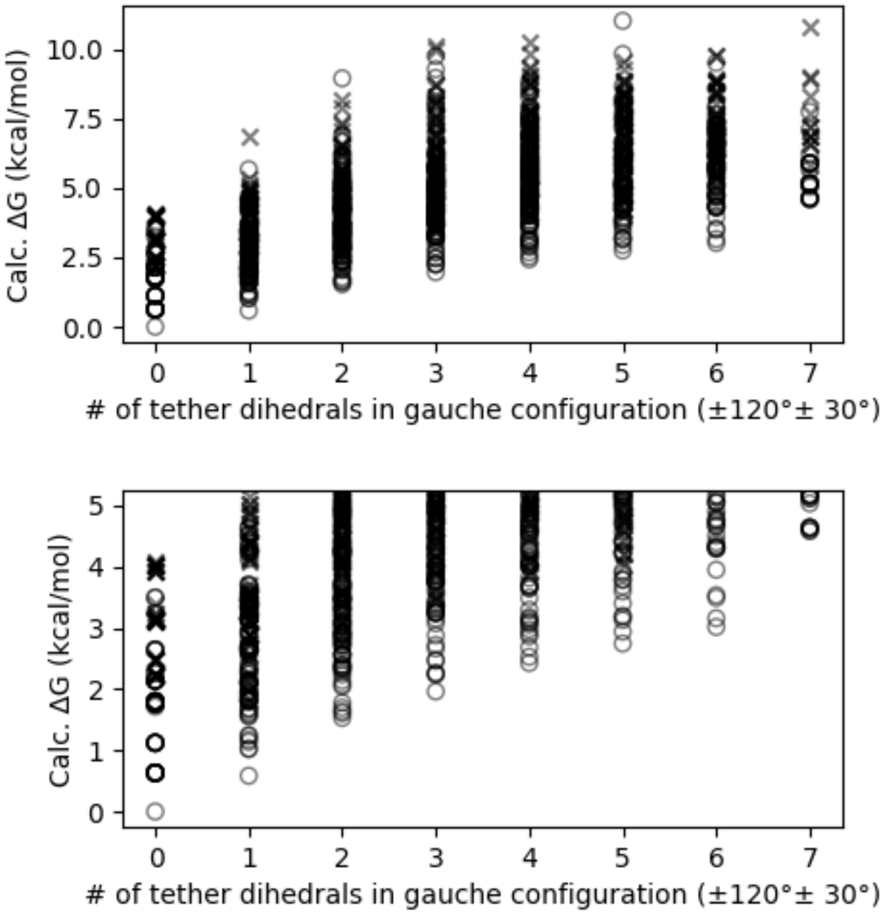

Figure S92. Number of tether dihedrals in *gauche* configuration vs. calculated ΔG for conformers of **4'** optimized at the M06-2X/def2-TZVP/(SMD=iPrOH) level. O markers = conformers with *E* iminiums. X markers = conformers with *Z* iminiums.

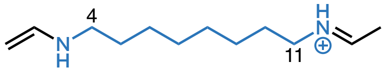

4’

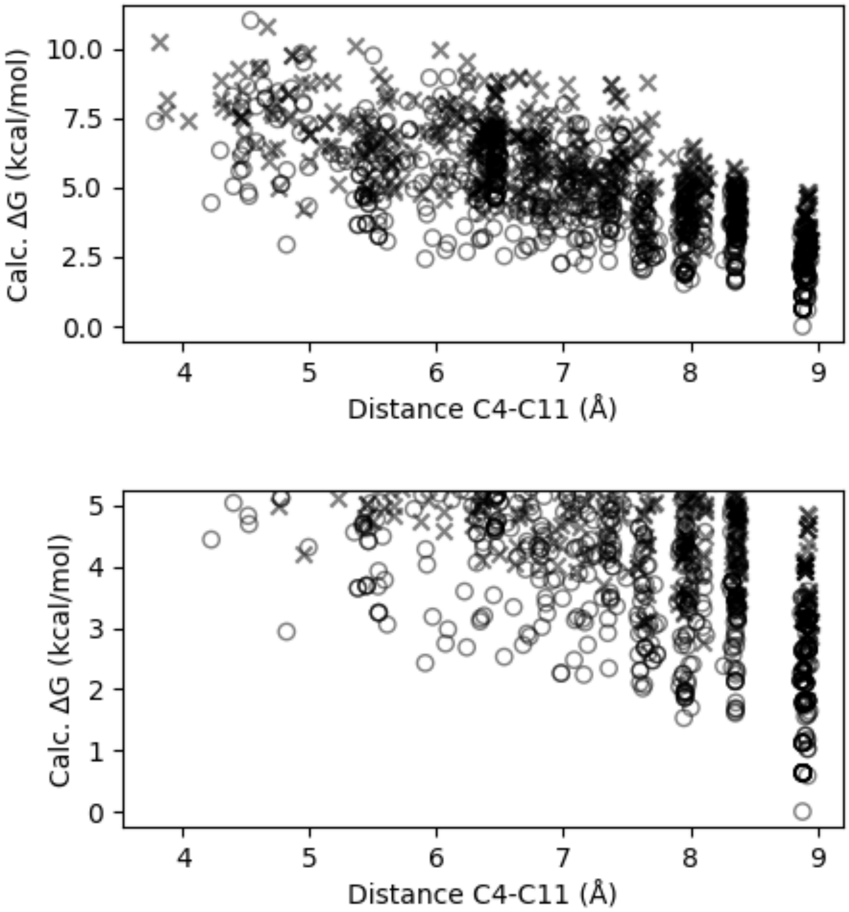

Figure S93. C4-C11 distances vs. calculated ΔG for conformers of **4’** optimized at the M06-2X/def2-TZVP/(SMD=iPrOH) level. O markers = conformers with *E* iminiums. X markers = conformers with *Z* iminiums.

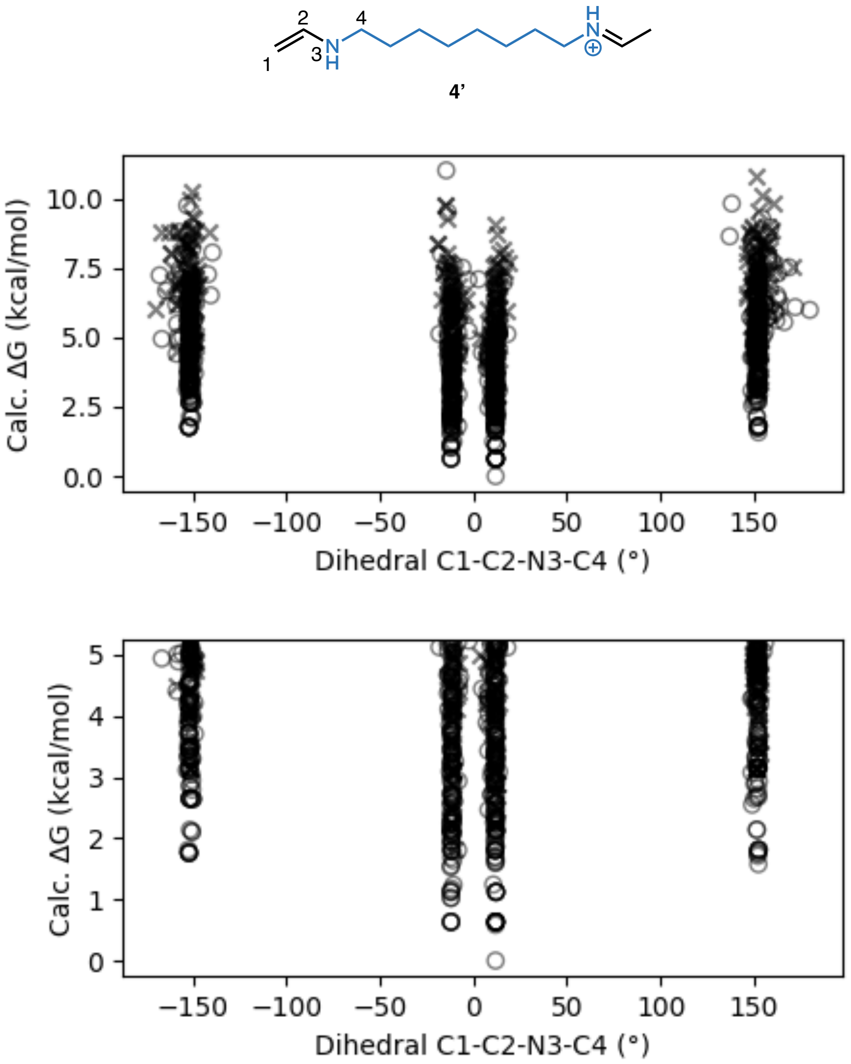

Figure S94. C1-C2-N3-C4 dihedral angles vs. calculated ΔG for conformers of **4’** optimized at the M06-2X/def2-TZVP/(SMD=iPrOH) level. O markers = conformers with *E* iminiums. X markers = conformers with *Z* iminiums.

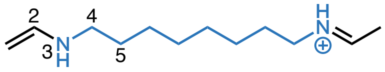

4’

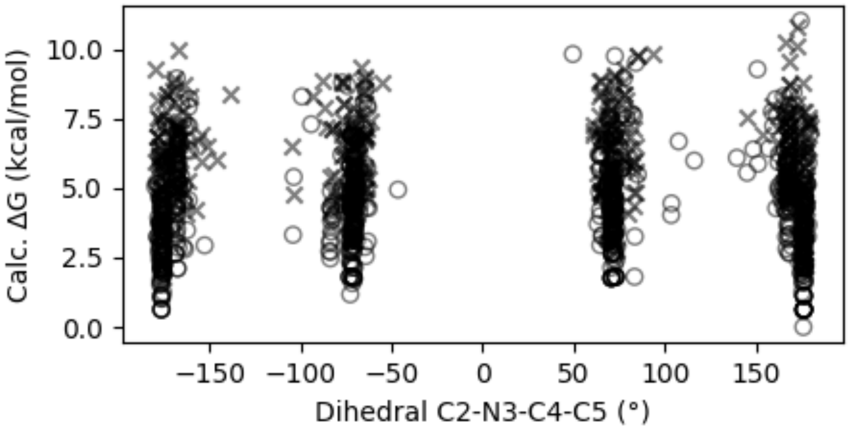

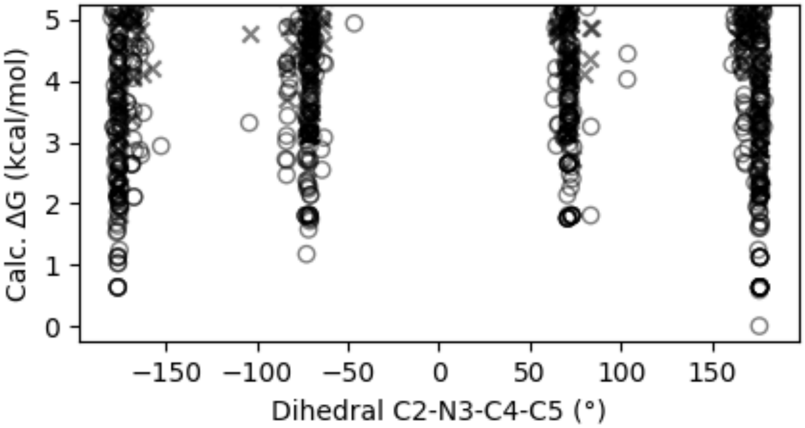

Figure S95. C2-N3-C4-C5 dihedral angles vs. calculated ΔG for conformers of **4’** optimized at the M06-2X/def2-TZVP/(SMD=iPrOH) level. O markers = conformers with *E* iminiums. X markers = conformers with *Z* iminiums.

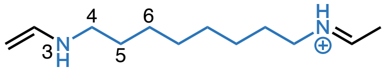

4’

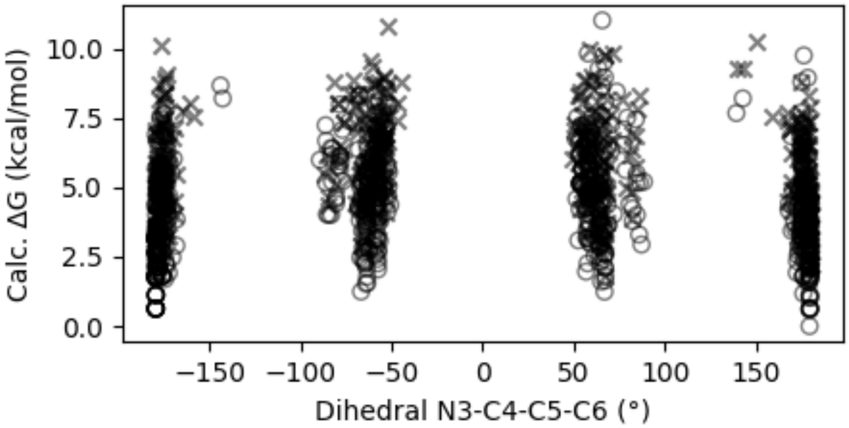

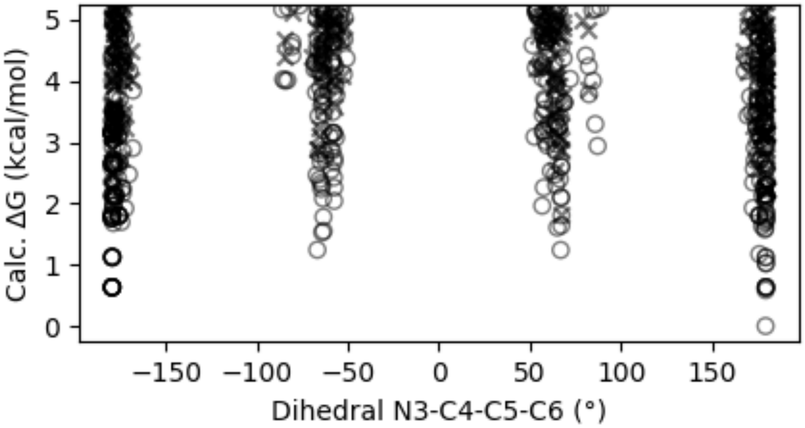

Figure S96. N3-C4-C5-C6 dihedral angles vs. calculated ΔG for conformers of **4’** optimized at the M06-2X/def2-TZVP/(SMD=iPrOH) level. O markers = conformers with *E* iminiums. X markers = conformers with *Z* iminiums.

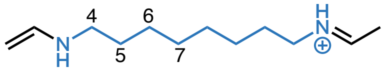

4’

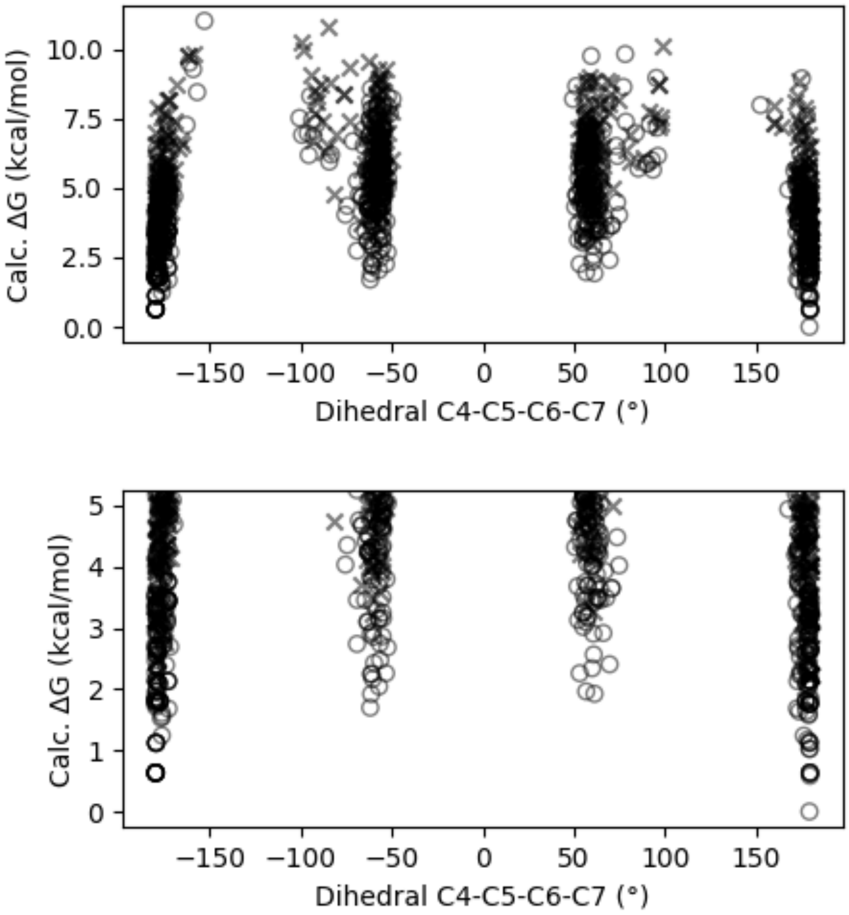

Figure S97. C4-C5-C6-C7 dihedral angles vs. calculated ΔG for conformers of **4’** optimized at the M06-2X/def2-TZVP/(SMD=iPrOH) level. O markers = conformers with *E* iminiums. X markers = conformers with *Z* iminiums.

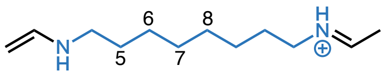

4'

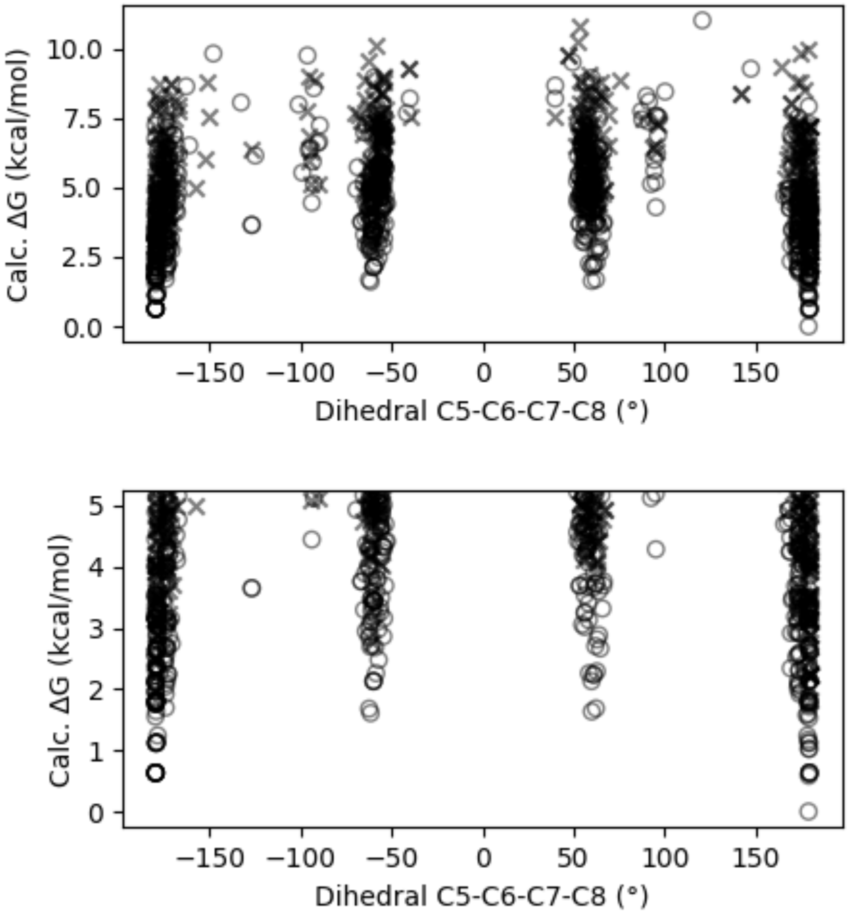

Figure S98. C5-C6-C7-C8 dihedral angles vs. calculated ΔG for conformers of **4'** optimized at the M06-2X/def2-TZVP/SMD=iPrOH) level. O markers = conformers with *E* iminiums. X markers = conformers with *Z* iminiums.

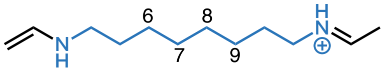

4’

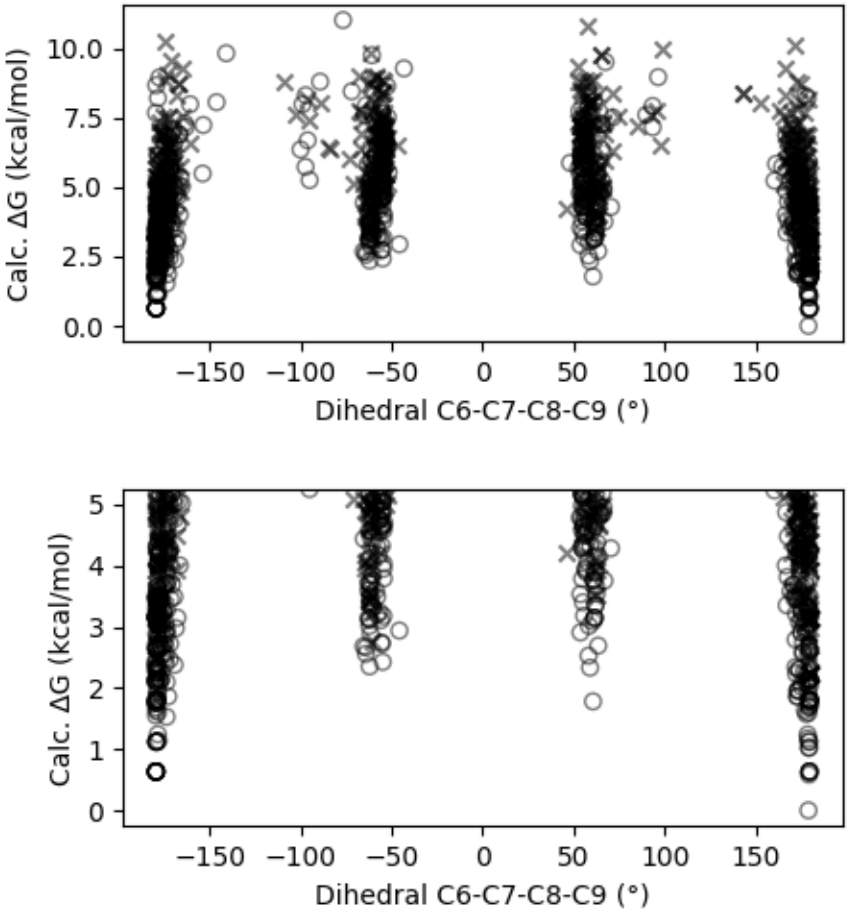

Figure S99. C6-C7-C8-C9 dihedral angles vs. calculated ΔG for conformers of **4’** optimized at the M06-2X/def2-TZVP/(SMD=iPrOH) level. O markers = conformers with *E* iminiums. X markers = conformers with *Z* iminiums.

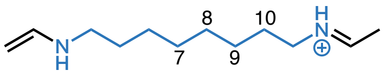

4’

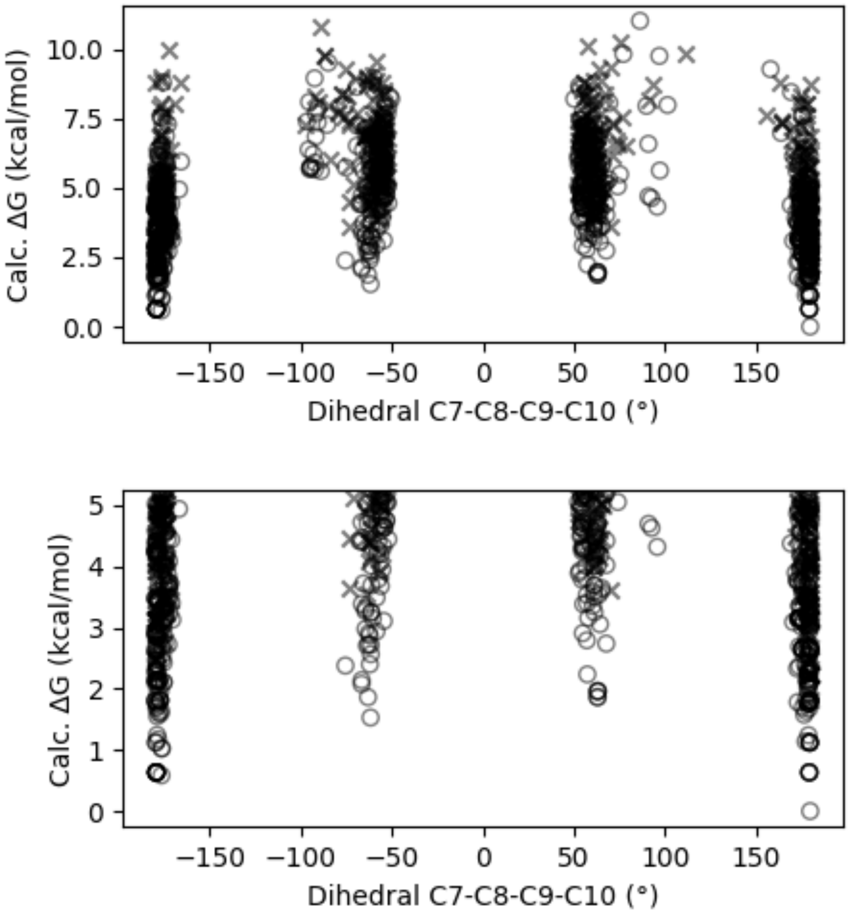

Figure S100. C7-C8-C9-C10 dihedral angles vs. calculated ΔG for conformers of **4’** optimized at the M06-2X/def2-TZVP/(SMD=iPrOH) level. O markers = conformers with *E* iminiums. X markers = conformers with *Z* iminiums.

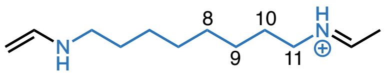

4’

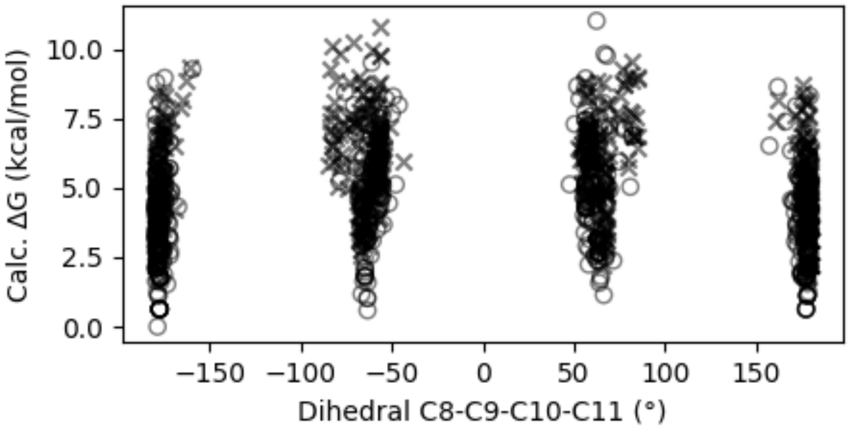

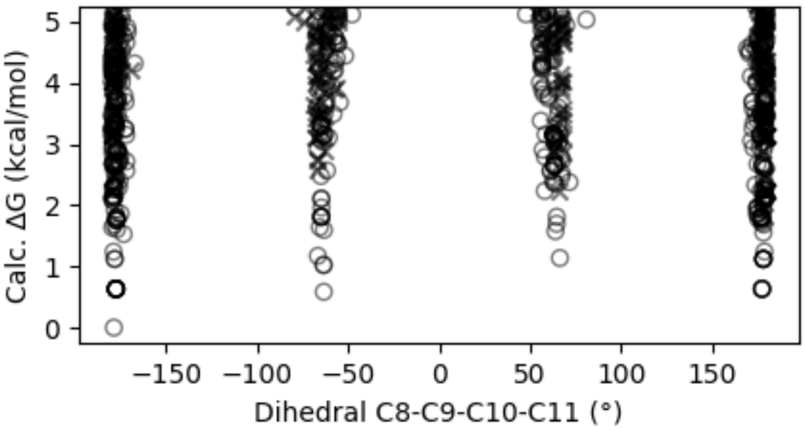

Figure S101. C8-C9-C10-C11 dihedral angles vs. calculated ΔG for conformers of **4’** optimized at the M06-2X/def2-TZVP/(SMD=iPrOH) level. O markers = conformers with *E* iminiums. X markers = conformers with *Z* iminiums.

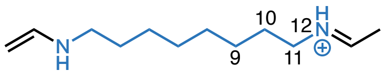

4’

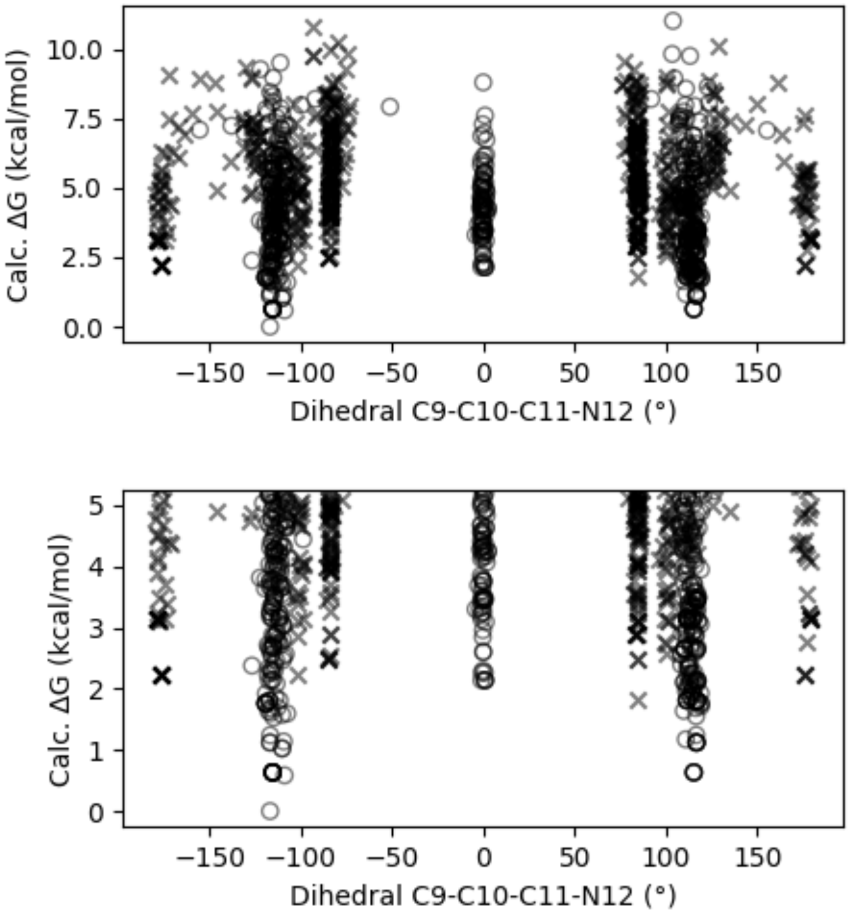

Figure S102. C9-C10-C11-N12 dihedral angles vs. calculated ΔG for conformers of **4’** optimized at the M06-2X/def2-TZVP/(SMD=iPrOH) level. O markers = conformers with *E* iminiums. X markers = conformers with *Z* iminiums.

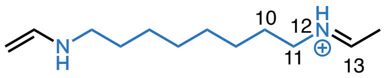

4’

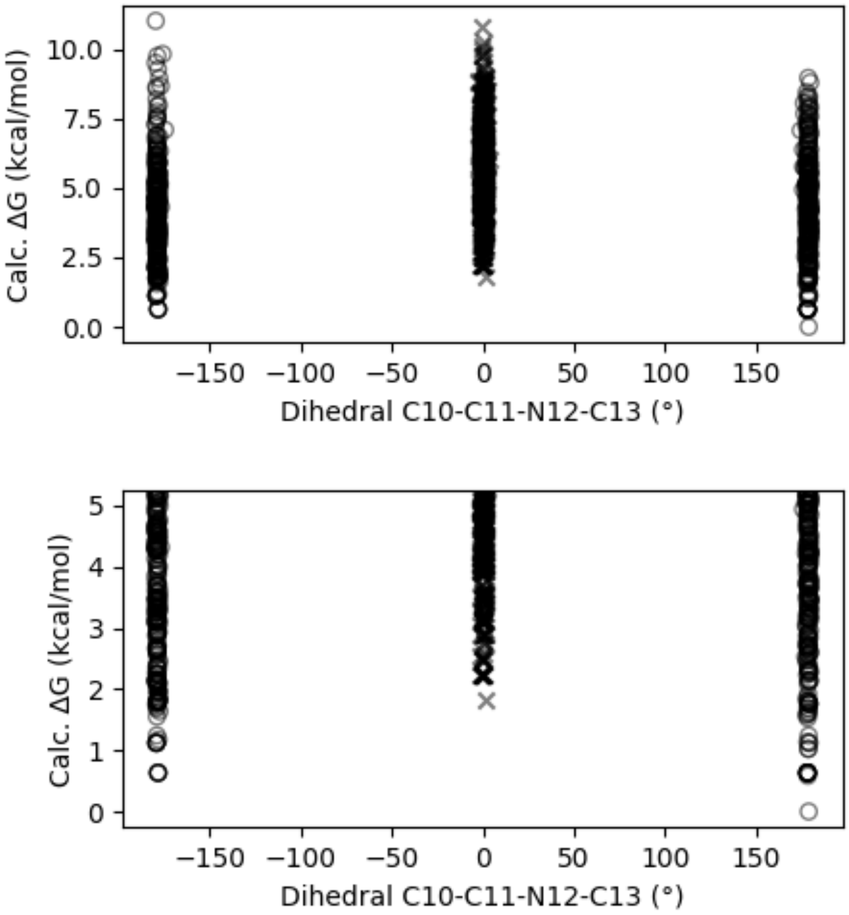

Figure S103. C10-C11-N12-C13 dihedral angles vs. calculated ΔG for conformers of **4’** optimized at the M06-2X/def2-TZVP/(SMD=iPrOH) level. O markers = conformers with *E* iminiums. X markers = conformers with *Z* iminiums.

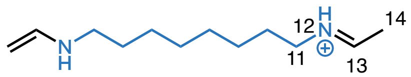

4’

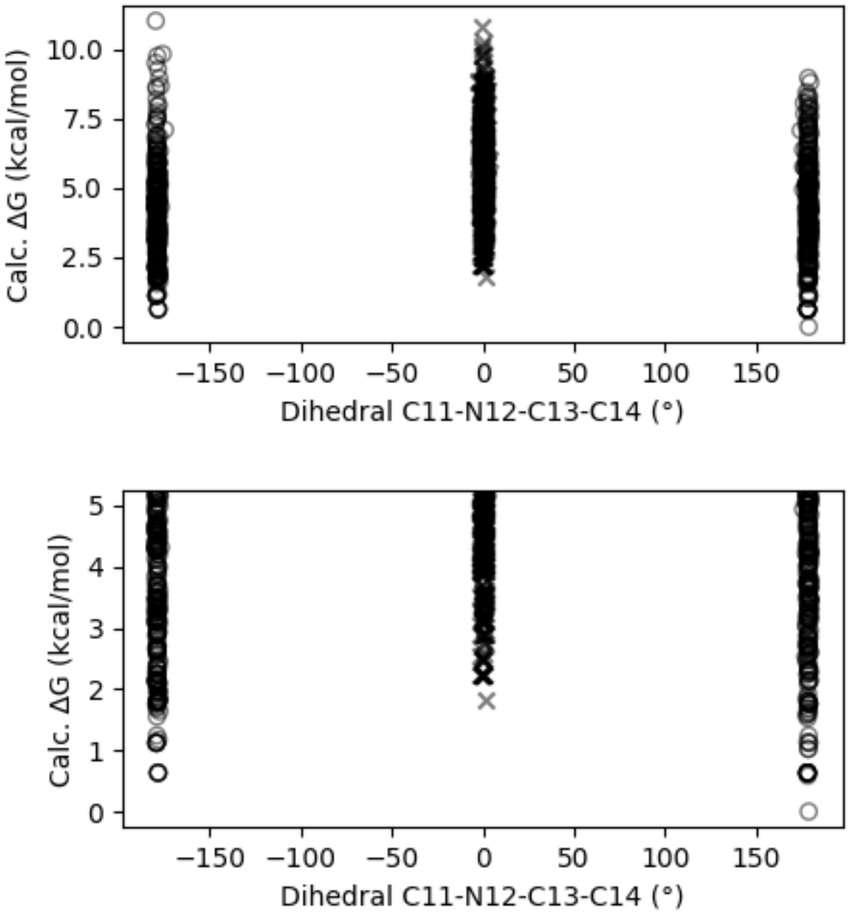

Figure S104. C11-N12-C13-C14 dihedral angles vs. calculated ΔG for conformers of **4’** optimized at the M06-2X/def2-TZVP/(SMD=iPrOH) level. O markers = conformers with *E* iminiums. X markers = conformers with *Z* iminiums.

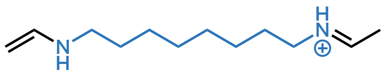

4’

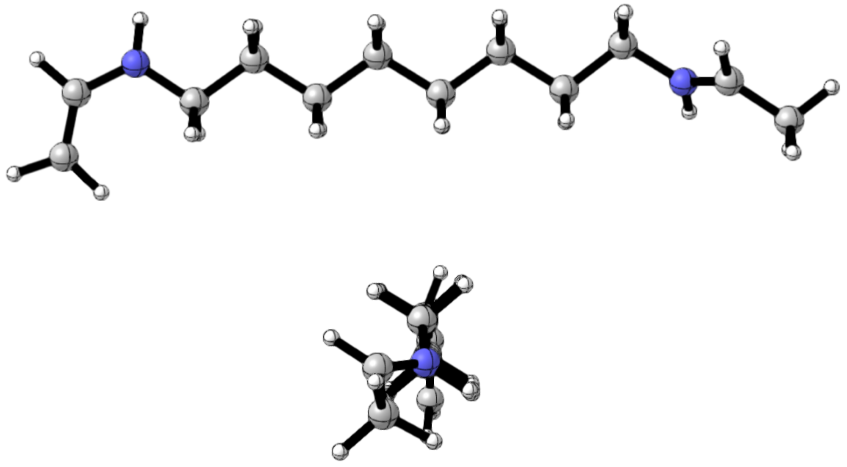

Figure S105. Front and side view of the lowest free energy conformer of **4’** at the M06-2X/def2-TZVP/(SMD=iPrOH) level.

6.6 DFT-derived Geometric Parameters for **12'** – B3LYP/6-31G(d,p)

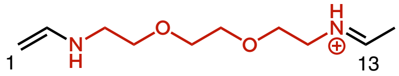

12'

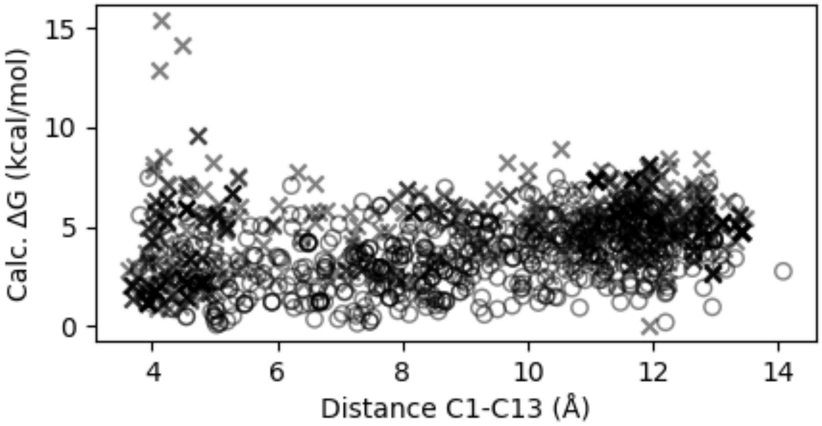

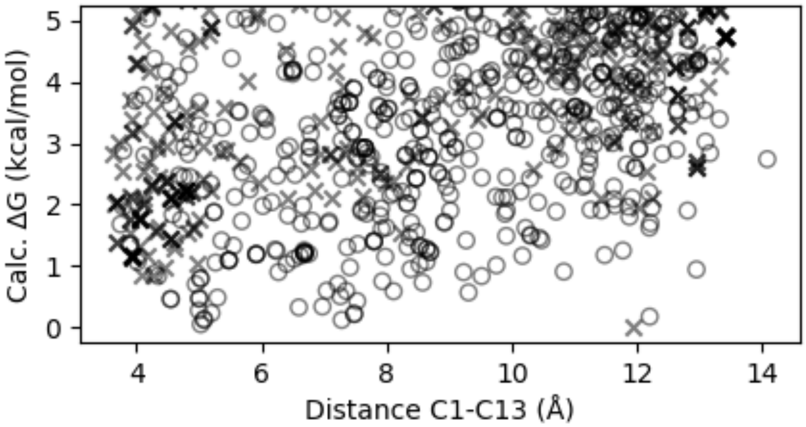

Figure S106. C1-C13 distances vs. calculated ΔG for conformers of **12'** optimized at the B3LYP/6-31G(d,p)/(SMD=iPrOH) level.

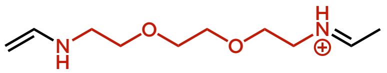

12’

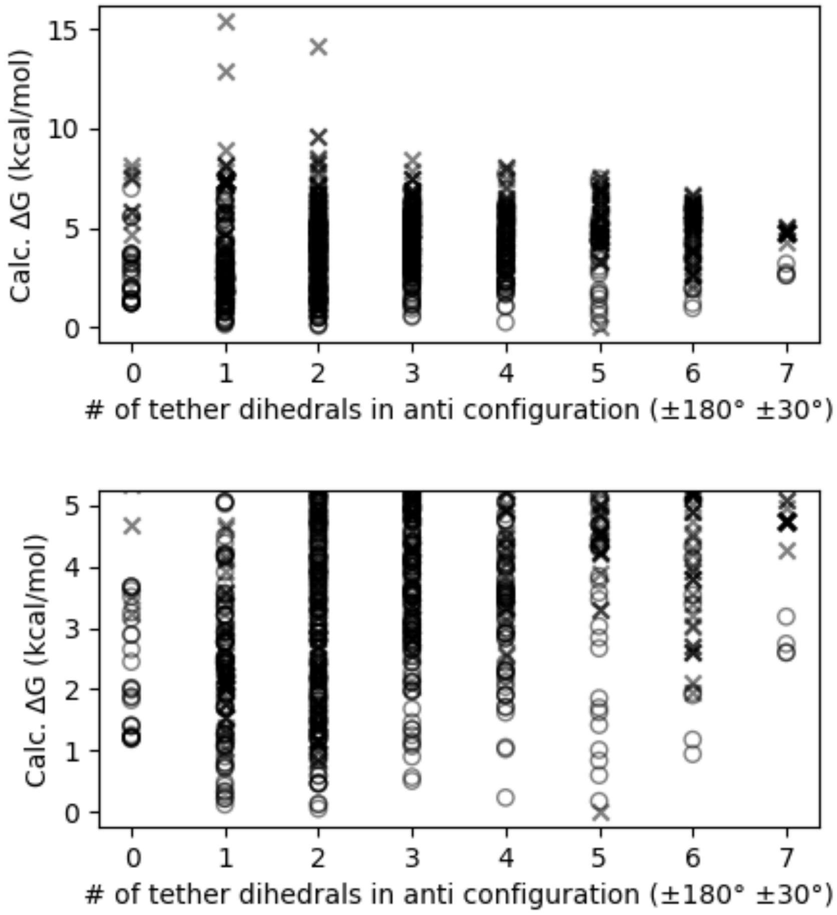

Figure S107. Number of tether dihedrals in *Anti* configuration vs. calculated ΔG for conformers of **12’** optimized at the B3LYP/6-31G(d,p)/(SMD=iPrOH) level.

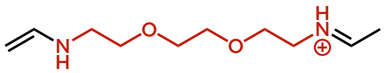

12'

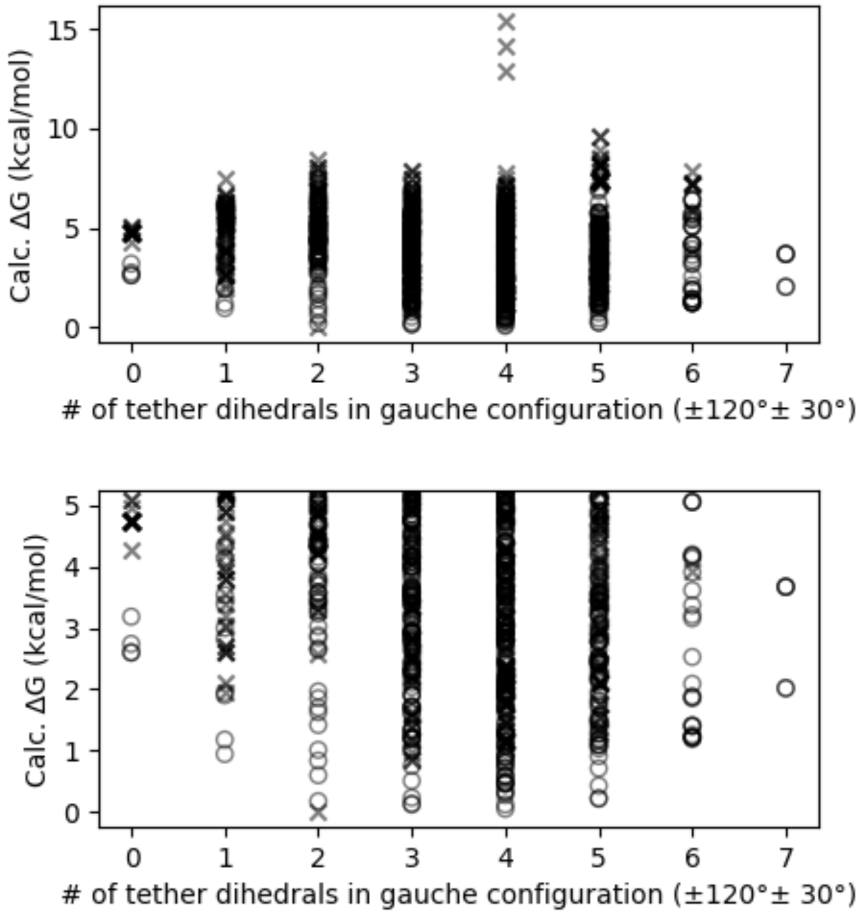

Figure S108. Number of tether dihedrals in *gauche* configuration vs. calculated ΔG for conformers of **12'** optimized at the B3LYP/6-31G(d,p)/(SMD=iPrOH) level. O markers = conformers with *E* iminiums. X markers = conformers with *Z* iminiums.

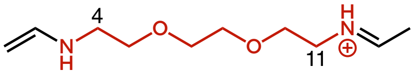

12’

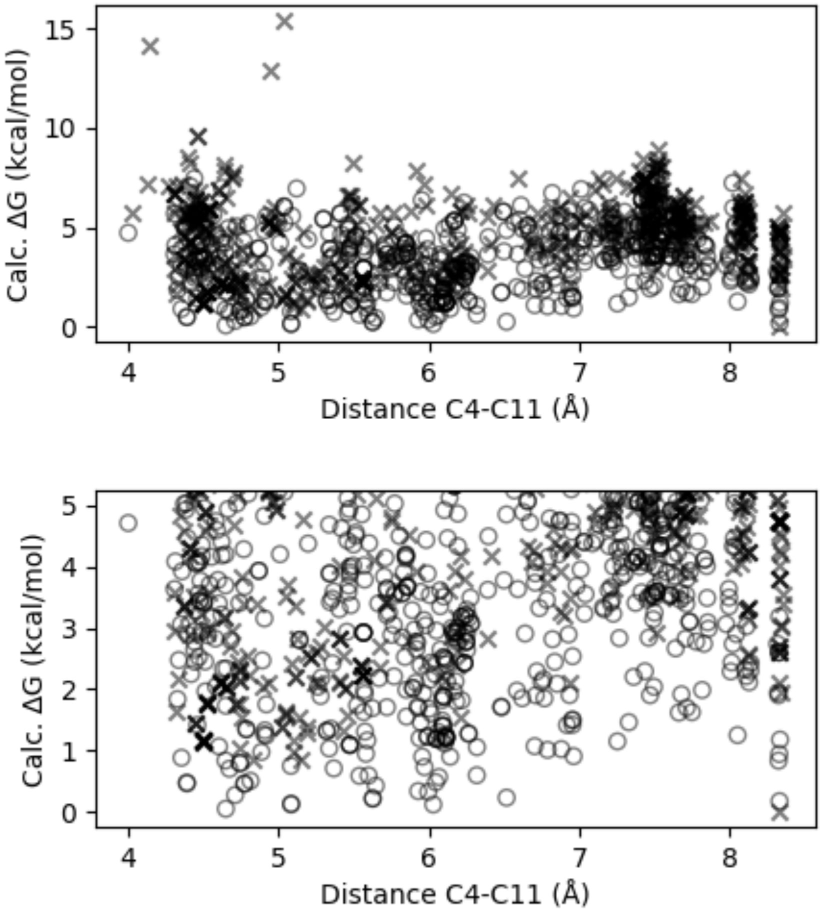

Figure S109. C4-C11 distances vs. calculated ΔG for conformers of **12’** optimized at the B3LYP/6-31G(d,p)/(SMD=iPrOH) level. O markers = conformers with *E* iminiums. X markers = conformers with *Z* iminiums.

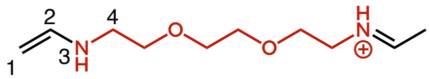

12’

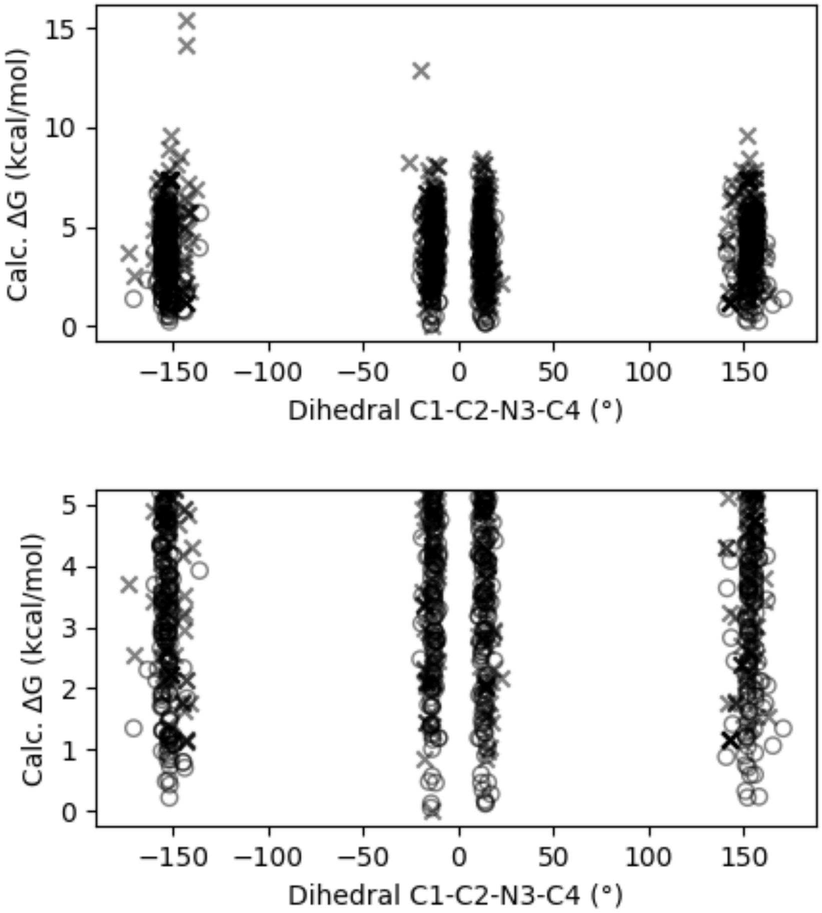

Figure S110. C1-C2-N3-C4 dihedral angles vs. calculated ΔG for conformers of **12’** optimized at the B3LYP/6-31G(d,p)/(SMD=iPrOH) level. O markers = conformers with *E* iminiums. X markers = conformers with *Z* iminiums.

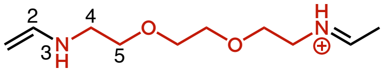

12’

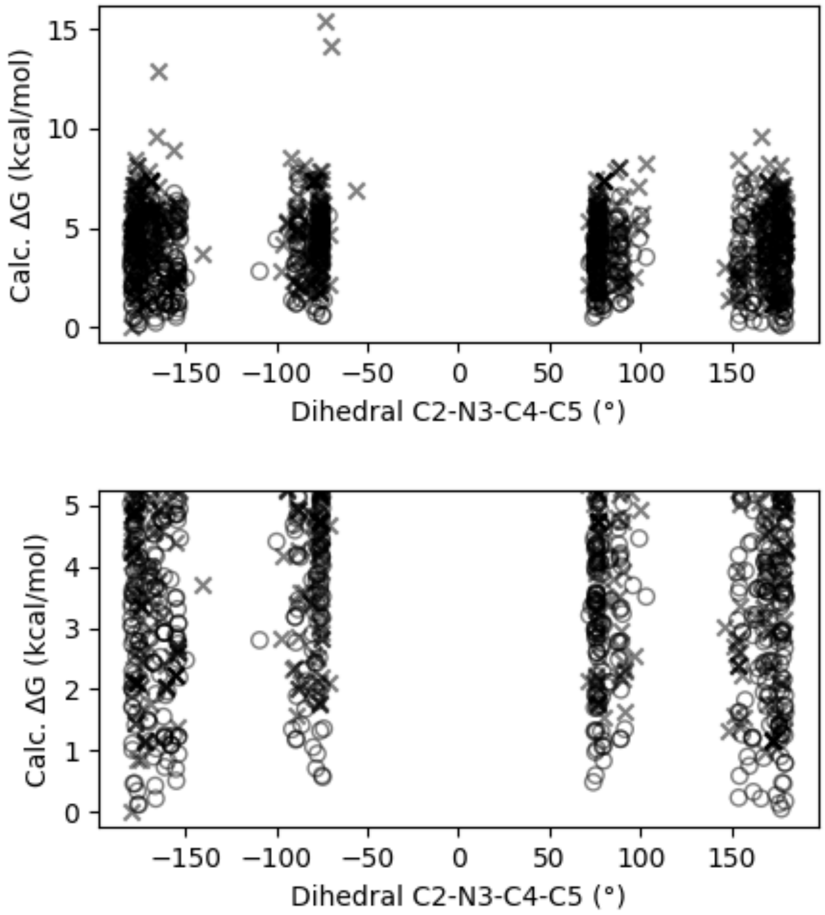

Figure S111. C2-N3-C4-C5 dihedral angles vs. calculated ΔG for conformers of **12’** optimized at the B3LYP/6-31G(d,p)/(SMD=iPrOH) level. O markers = conformers with *E* iminiums. X markers = conformers with *Z* iminiums.

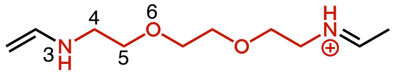

12’

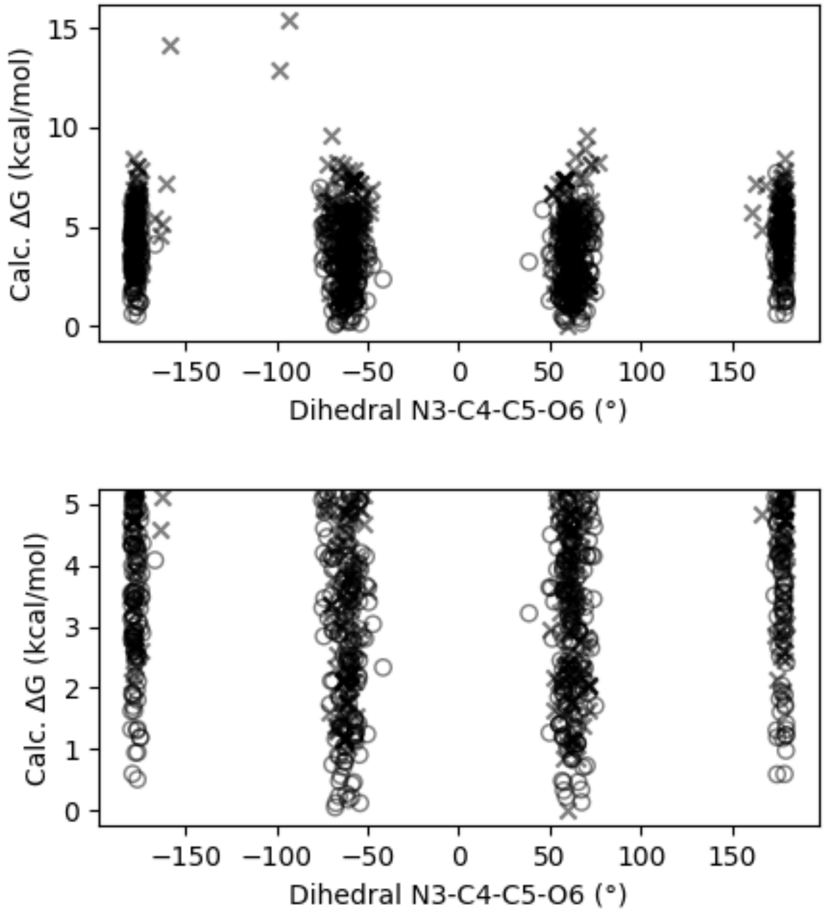

Figure S112. N3-C4-C5-O6 dihedral angles vs. calculated ΔG for conformers of **12’** optimized at the B3LYP/6-31G(d,p)/(SMD=iPrOH) level. O markers = conformers with *E* iminiums. X markers = conformers with *Z* iminiums.

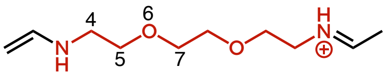

12’

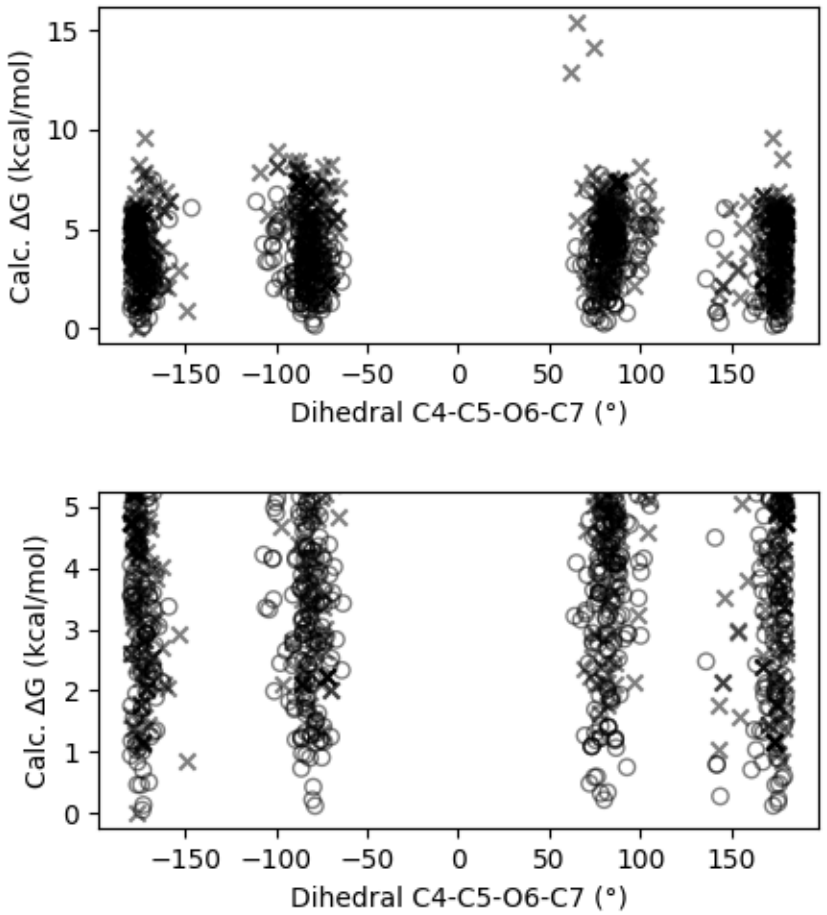

Figure S113. C4-C5-O6-C7 dihedral angles vs. calculated ΔG for conformers of **12’** optimized at the B3LYP/6-31G(d,p)/(SMD=iPrOH) level. O markers = conformers with *E* iminiums. X markers = conformers with *Z* iminiums.

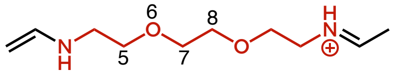

12’

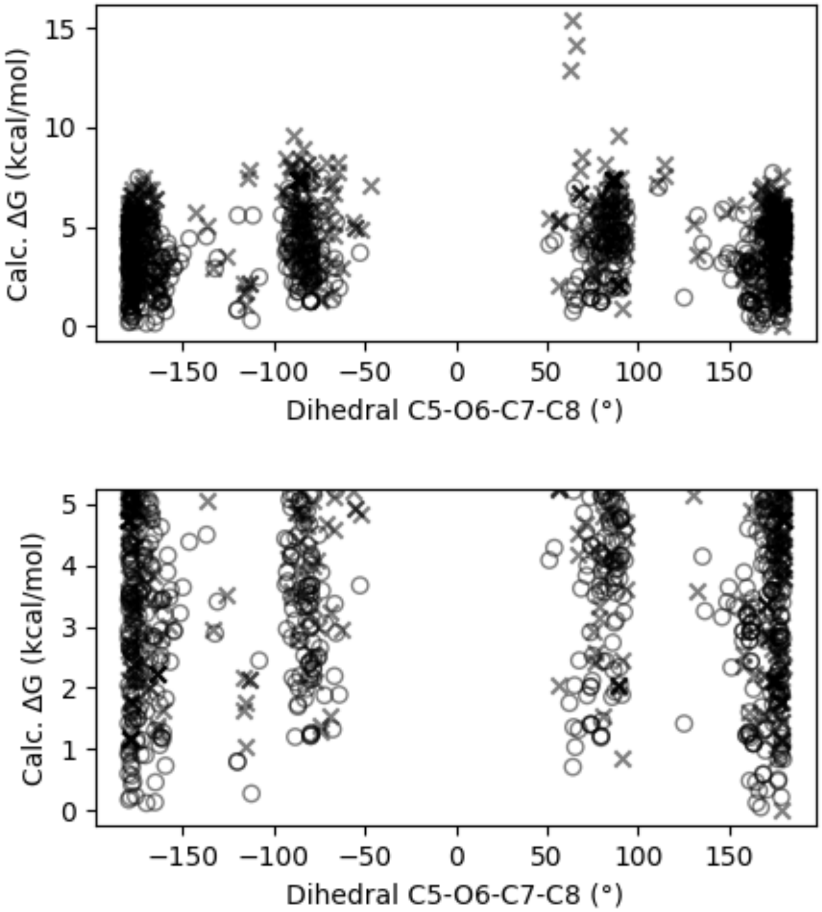

Figure S114. C5-C6-C7-C8 dihedral angles vs. calculated ΔG for conformers of **12’** optimized at the B3LYP/6-31G(d,p)/(SMD=iPrOH) level. O markers = conformers with *E* iminiums. X markers = conformers with *Z* iminiums.

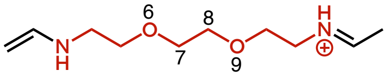

12'

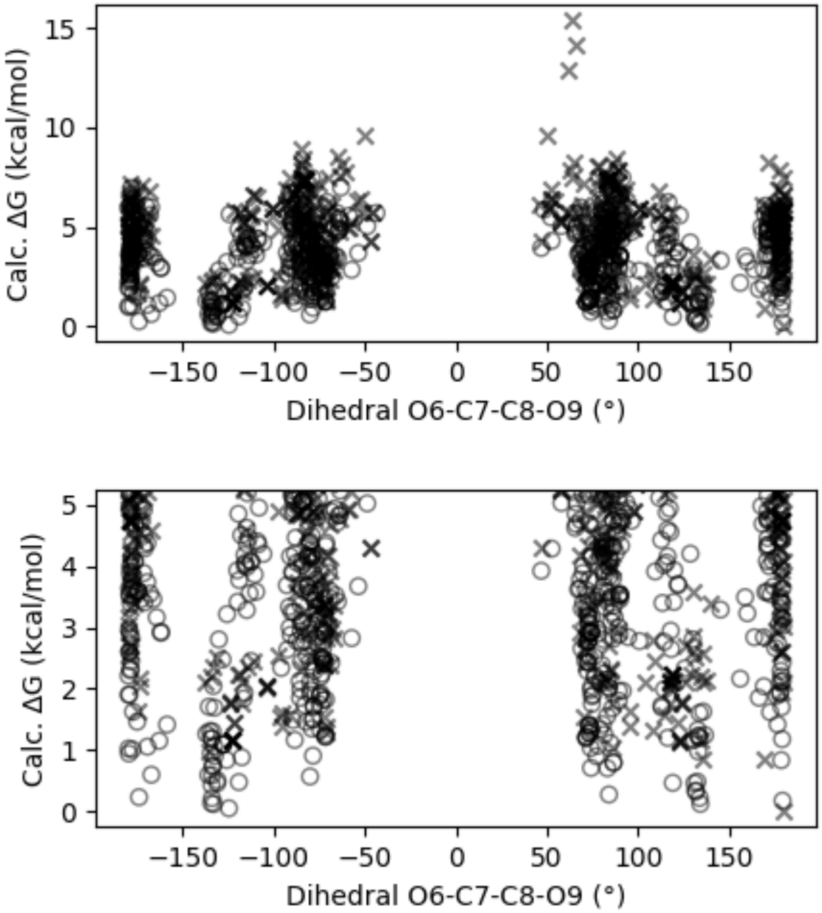

Figure S115. O6-C7-C8-O9 dihedral angles vs. calculated ΔG for conformers of **12'** optimized at the B3LYP/6-31G(d,p)/(SMD=iPrOH) level. O markers = conformers with *E* iminiums. X markers = conformers with *Z* iminiums.

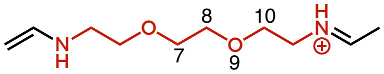

12’

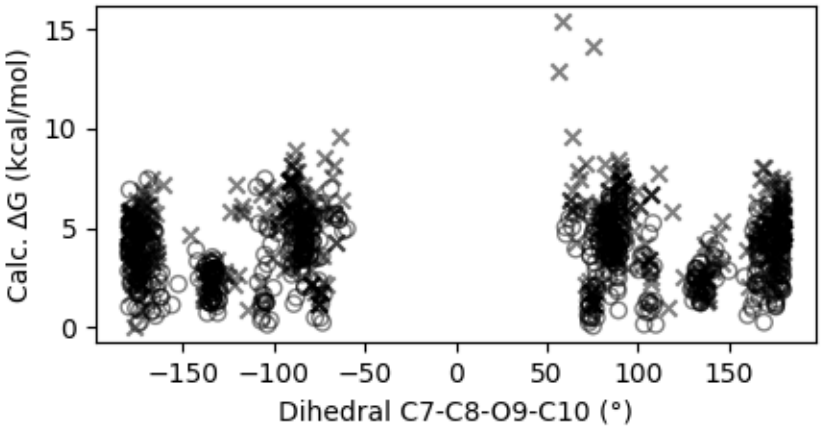

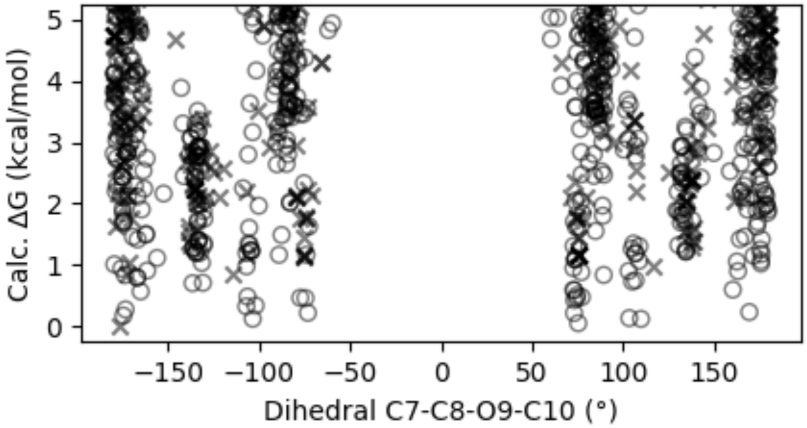

Figure S116. C7-C8-O9-C10 dihedral angles vs. calculated ΔG for conformers of **12’** optimized at the B3LYP/6-31G(d,p)/(SMD=iPrOH) level. O markers = conformers with *E* iminiums. X markers = conformers with *Z* iminiums.

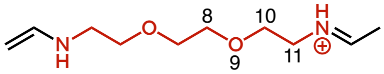

12’

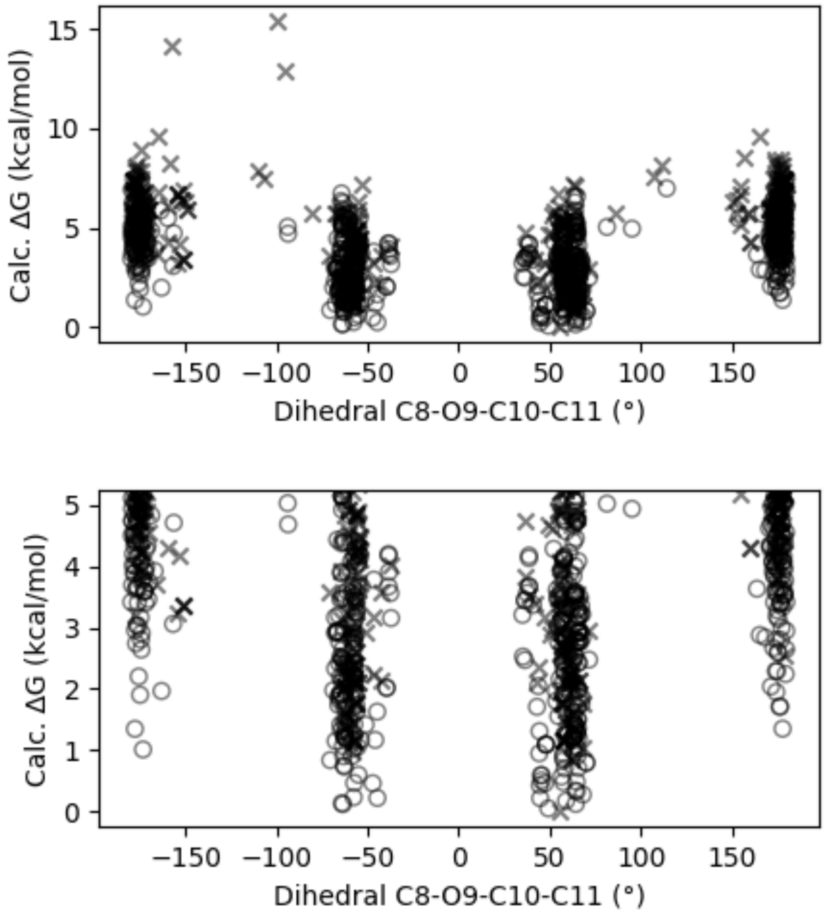

Figure S117. C8-O9-C10-C11 dihedral angles vs. calculated ΔG for conformers of **12’** optimized at the B3LYP/6-31G(d,p)/(SMD=iPrOH) level. O markers = conformers with *E* iminiums. X markers = conformers with *Z* iminiums.

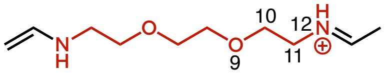

12'

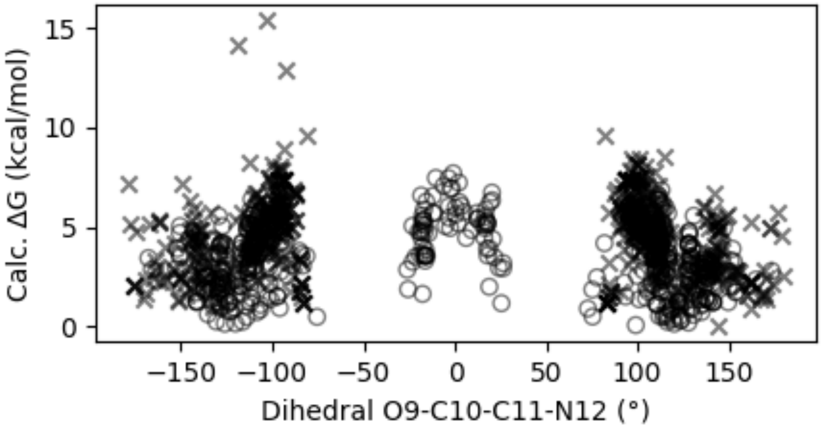

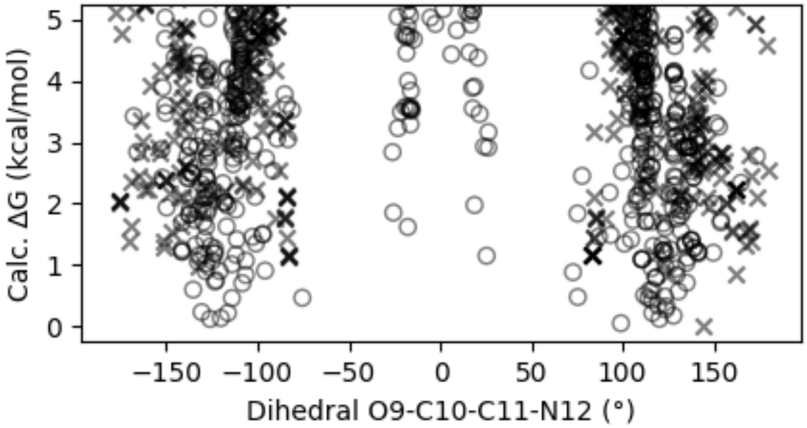

Figure S118. O9-C10-C11-N12 dihedral angles vs. calculated ΔG for conformers of **12'** optimized at the B3LYP/6-31G(d,p)/(SMD=iPrOH) level. O markers = conformers with *E* iminiums. X markers = conformers with *Z* iminiums.

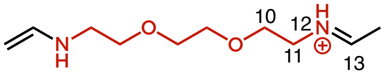

12'

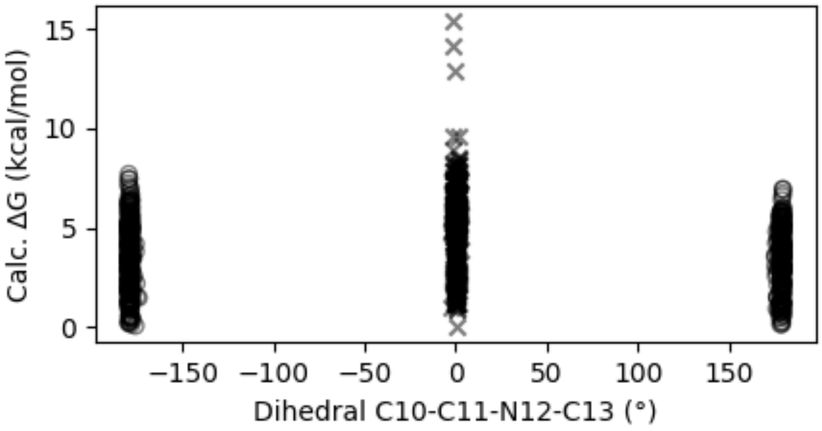

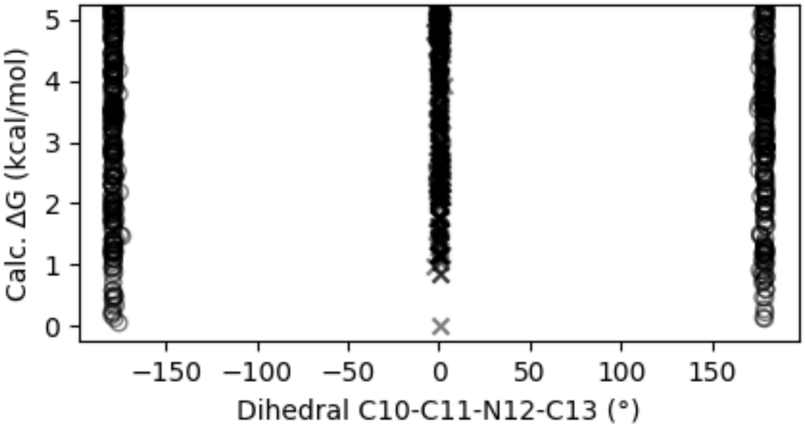

Figure S119. C10-C11-N12-C13 dihedral angles vs. calculated ΔG for conformers of **12'** optimized at the B3LYP/6-31G(d,p)/(SMD=iPrOH) level. O markers = conformers with *E* iminiums. X markers = conformers with *Z* iminiums.

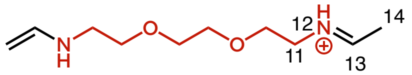

12’

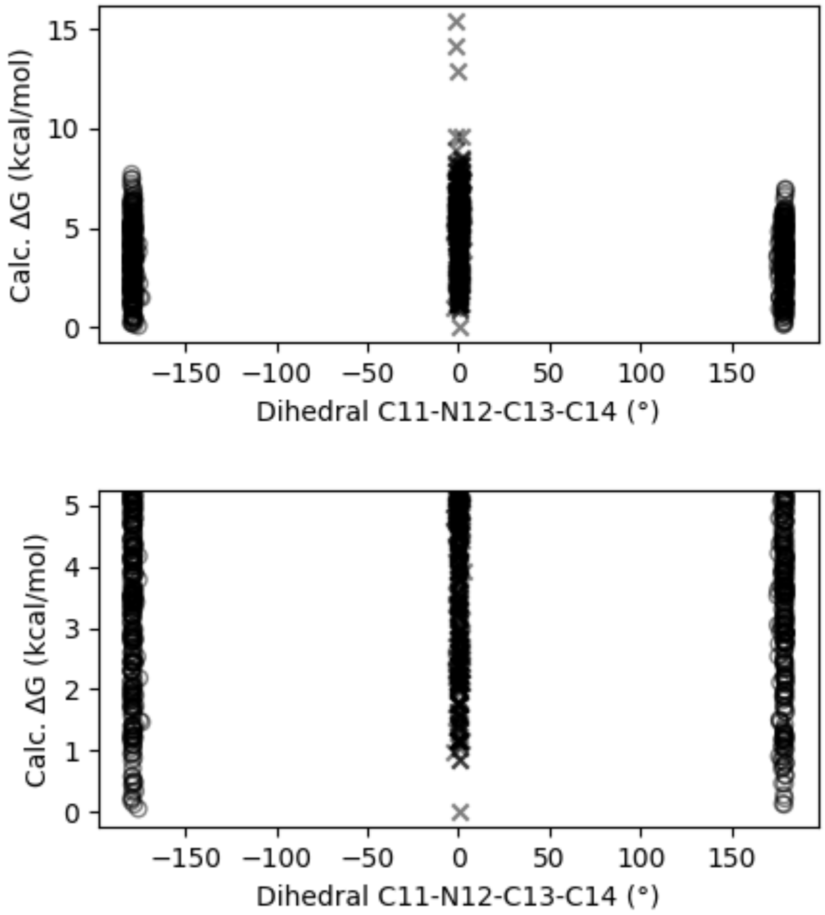

Figure S120. C11-N12-C13-C14 dihedral angles vs. calculated ΔG for conformers of **12’** optimized at the B3LYP/6-31G(d,p)/(SMD=iPrOH) level. O markers = conformers with *E* iminiums. X markers = conformers with *Z* iminiums.

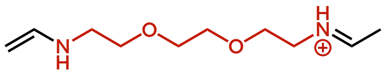

12’

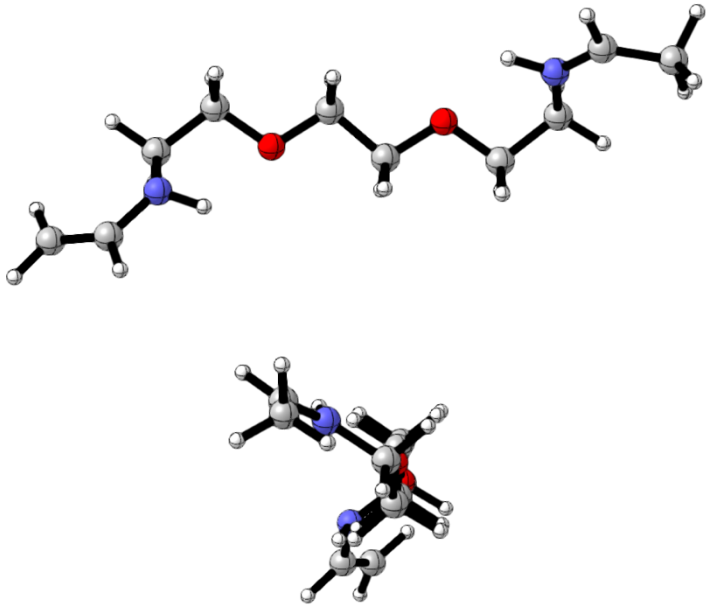

Figure S121. Front and side view of the lowest free energy conformer of **12’** at the B3LYP/6-31G(d,p)/(SMD=iPrOH) level.

6.7 DFT-derived Geometric Parameters for **12'** – M06-2X/def2-TZVP

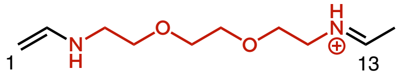

12'

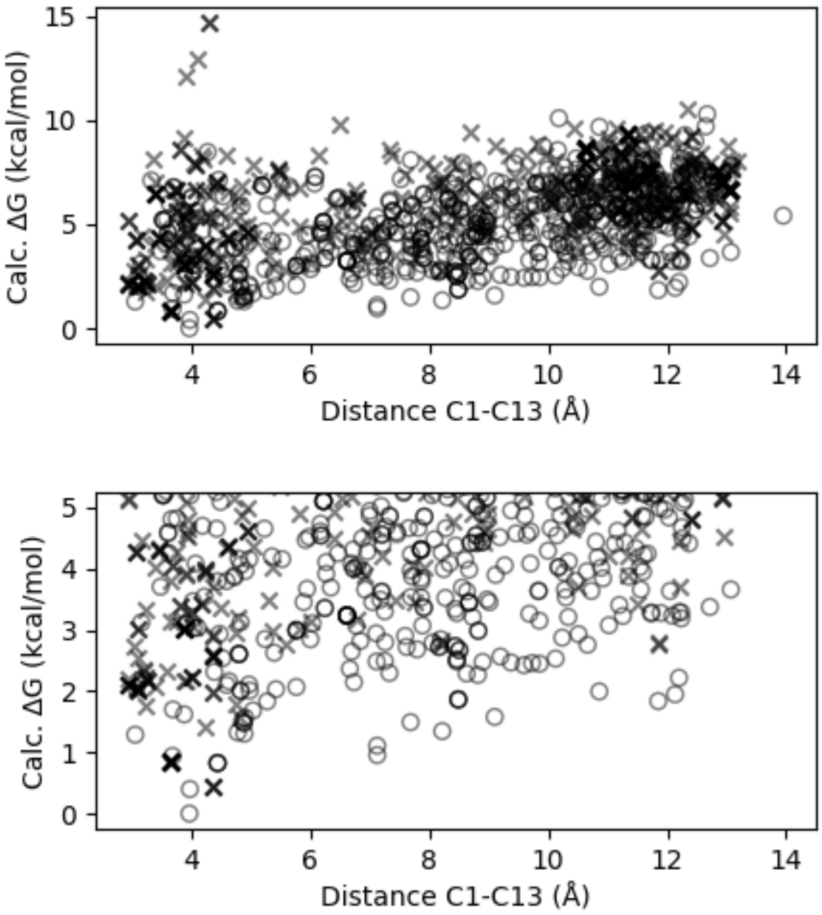

Figure S122. C1-C13 distances vs. calculated ΔG for conformers of **12'** optimized at the M06-2X/def2-TZVP/(SMD=iPrOH) level.

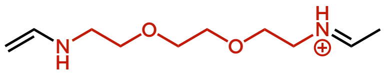

12’

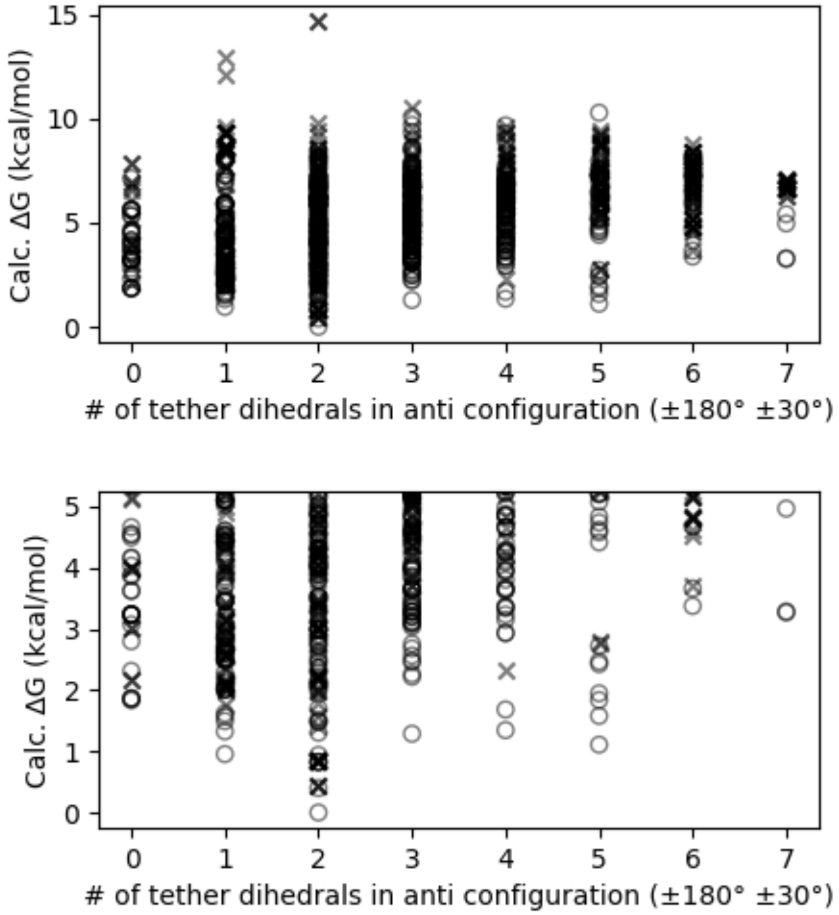

Figure S123. Number of tether dihedrals in *Anti* configuration vs. calculated ΔG for conformers of **12’** optimized at the M06-2X/def2-TZVP/(SMD=iPrOH) level.

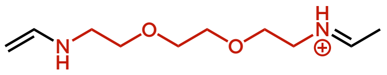

12’

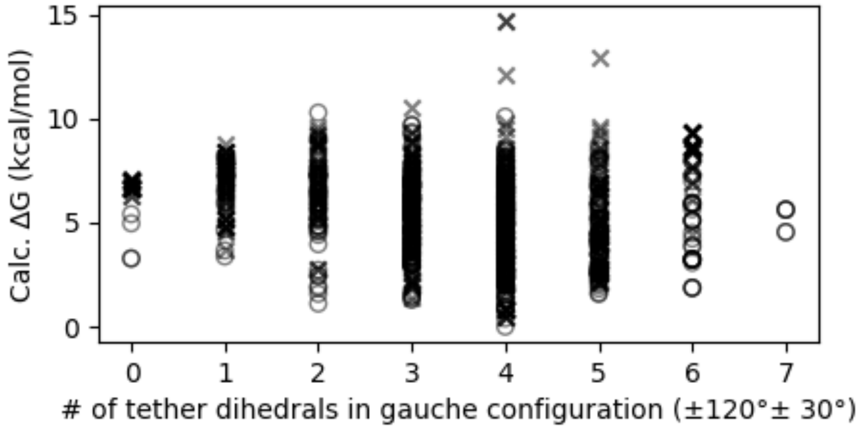

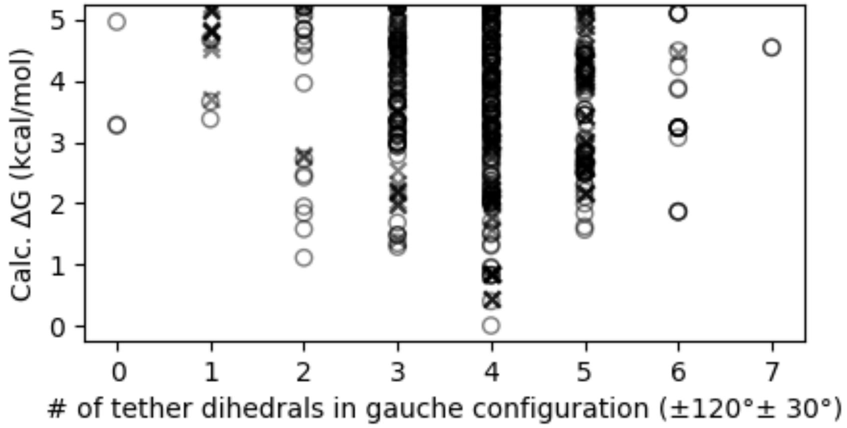

Figure S124. Number of tether dihedrals in *gauche* configuration vs. calculated ΔG for conformers of **12’** optimized at the M06-2X/def2-TZVP/(SMD=iPrOH) level. O markers = conformers with *E* iminiums. X markers = conformers with *Z* iminiums.

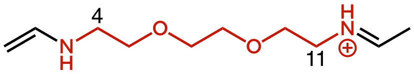

12’

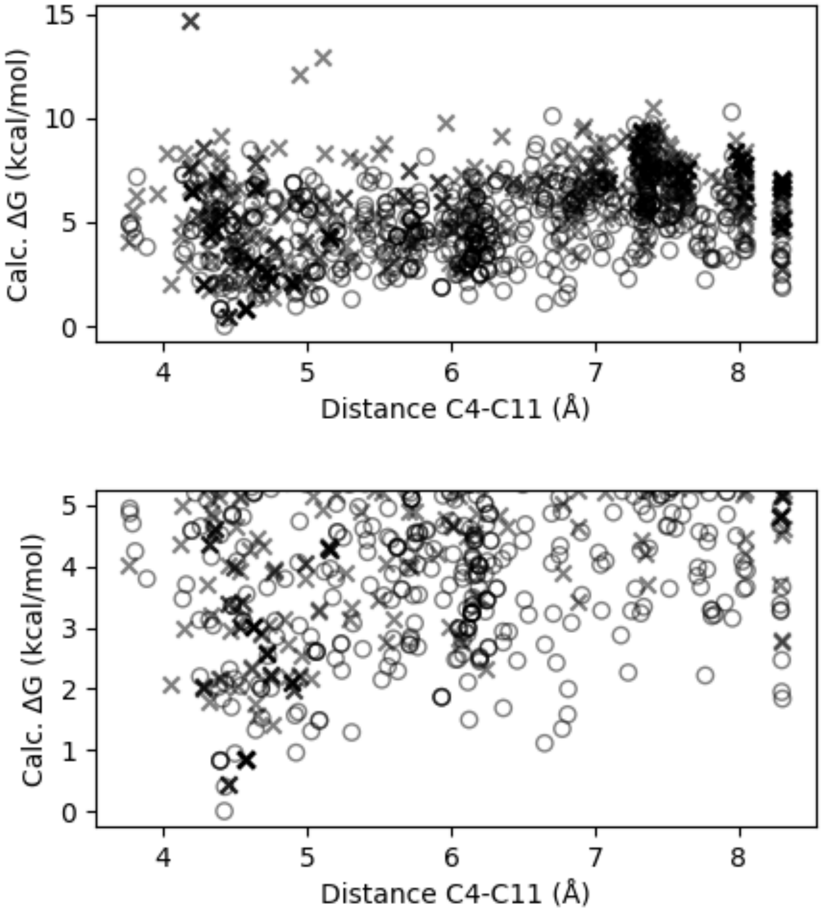

Figure S125. C4-C11 distances vs. calculated ΔG for conformers of **12’** optimized at the M06-2X/def2-TZVP/(SMD=iPrOH) level. O markers = conformers with *E* iminiums. X markers = conformers with *Z* iminiums.

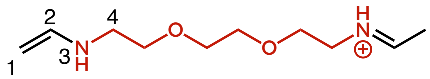

12’

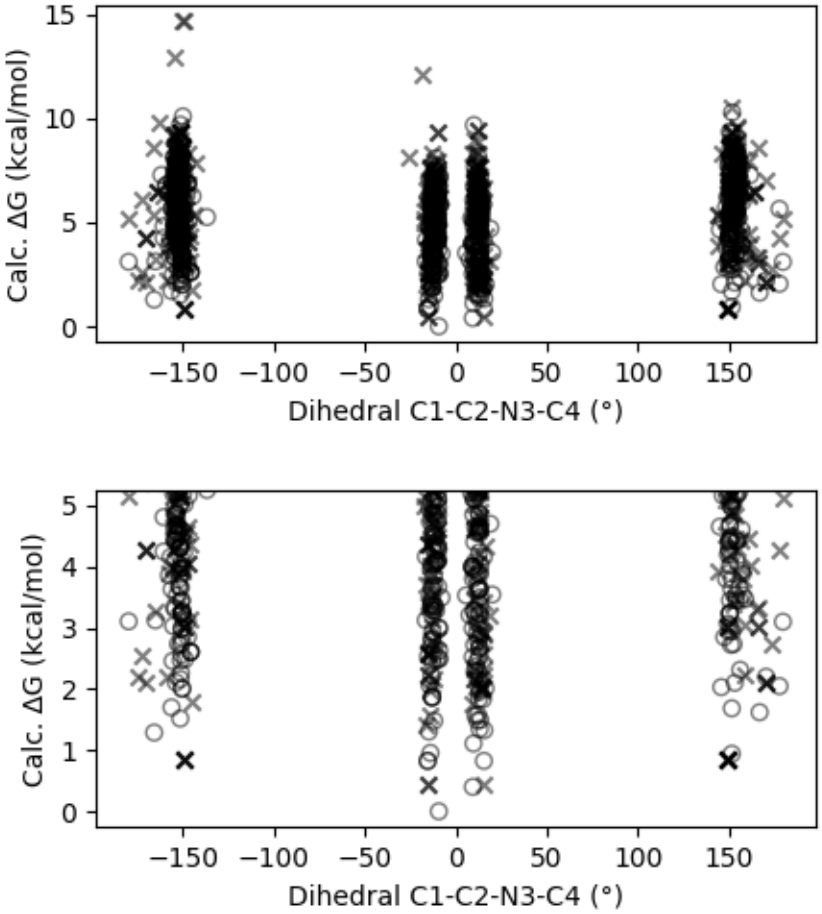

Figure S126. C1-C2-N3-C4 dihedral angles vs. calculated ΔG for conformers of **12’** optimized at the M06-2X/def2-TZVP/(SMD=iPrOH) level. O markers = conformers with *E* iminiums. X markers = conformers with *Z* iminiums.

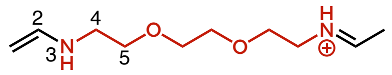

12’

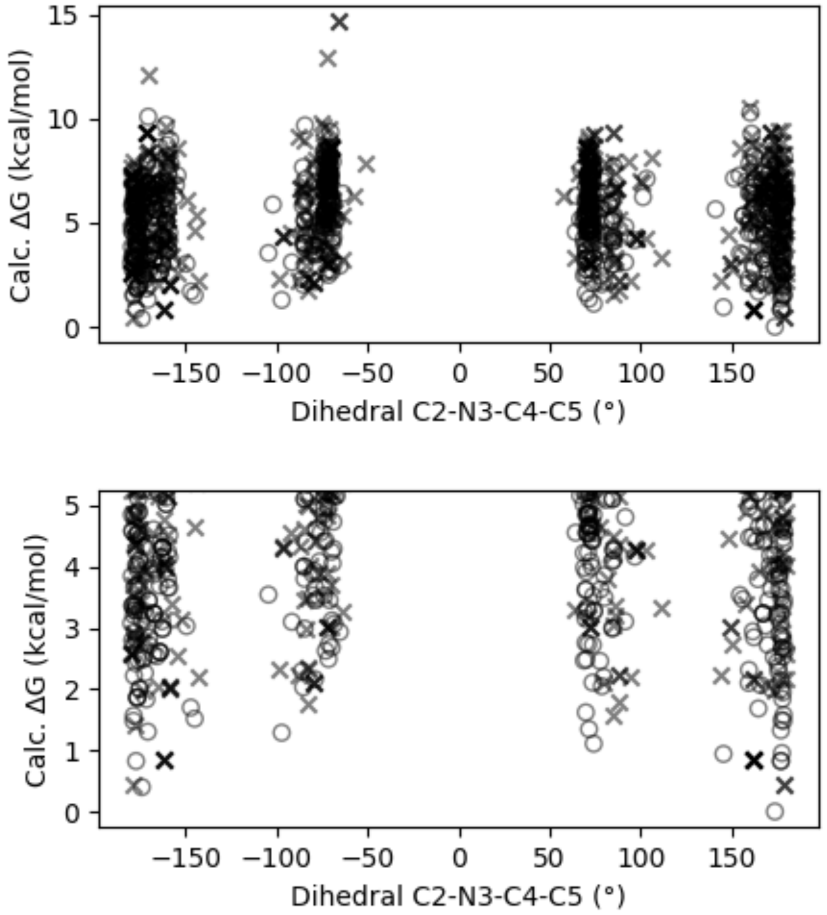

Figure S127. C2-N3-C4-C5 dihedral angles vs. calculated ΔG for conformers of **12’** optimized at the M06-2X/def2-TZVP/(SMD=iPrOH) level. O markers = conformers with *E* iminiums. X markers = conformers with *Z* iminiums.

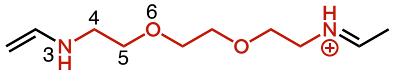

12’

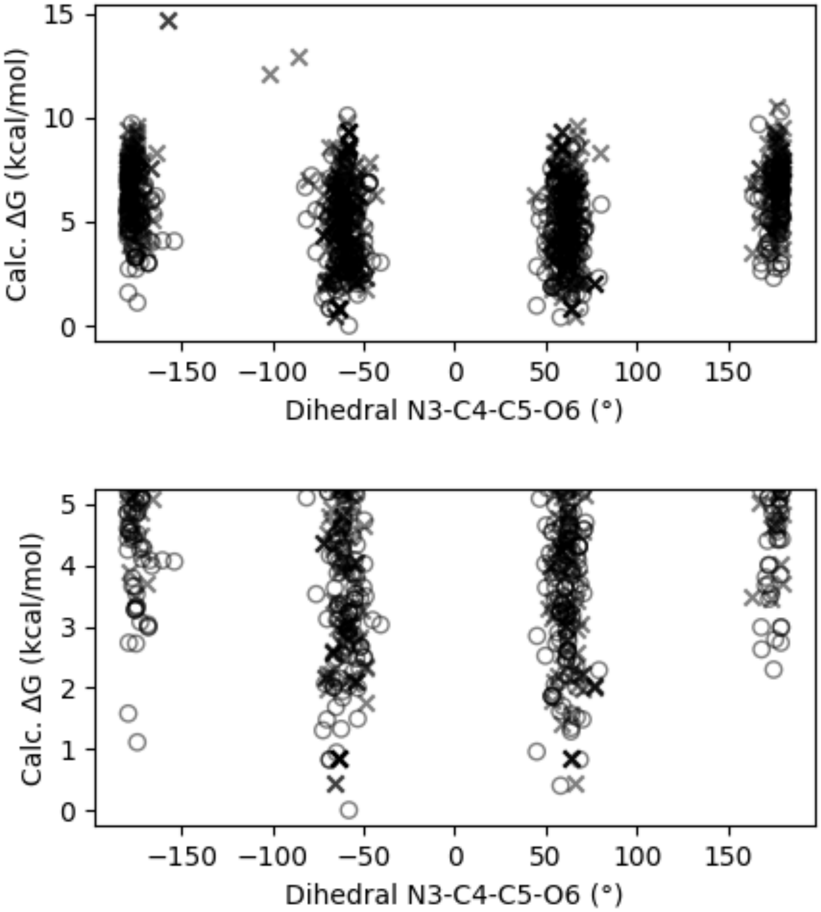

Figure S128. N3-C4-C5-O6 dihedral angles vs. calculated ΔG for conformers of **12’** optimized at the M06-2X/def2-TZVP/(SMD=iPrOH). O markers = conformers with *E* iminiums. X markers = conformers with *Z* iminiums.

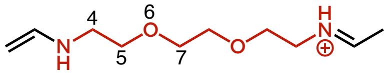

12’

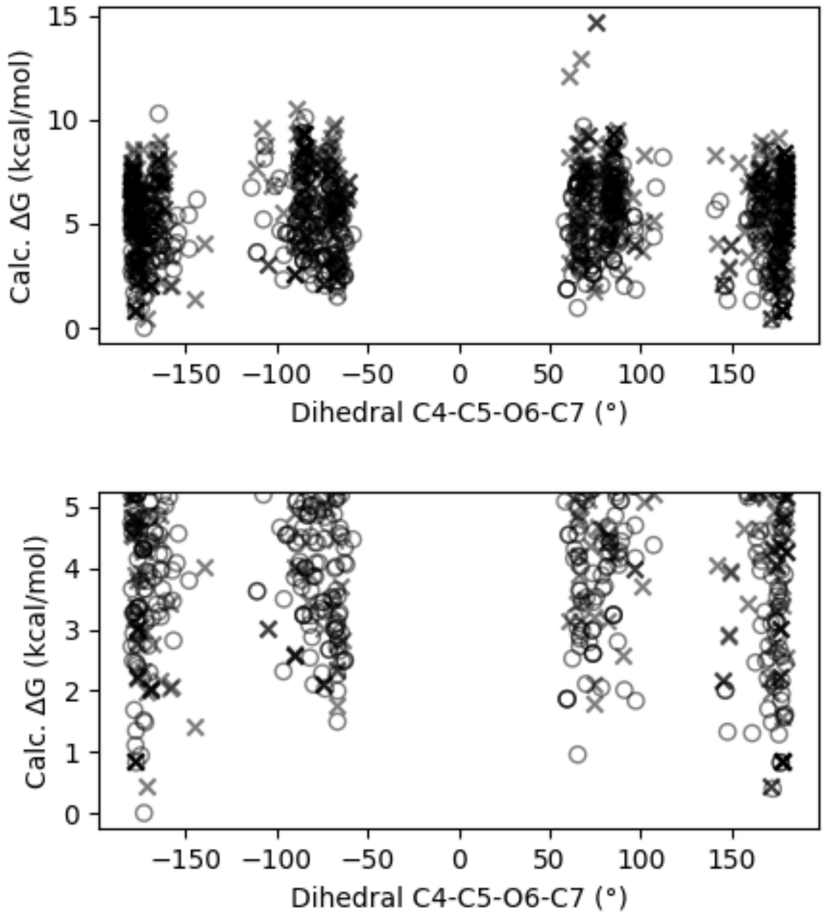

Figure S129. C4-C5-O6-C7 dihedral angles vs. calculated ΔG for conformers of **12’** optimized at the M06-2X/def2-TZVP/(SMD=iPrOH) level. O markers = conformers with *E* iminiums. X markers = conformers with *Z* iminiums.

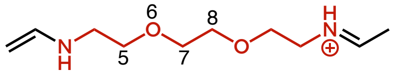

12’

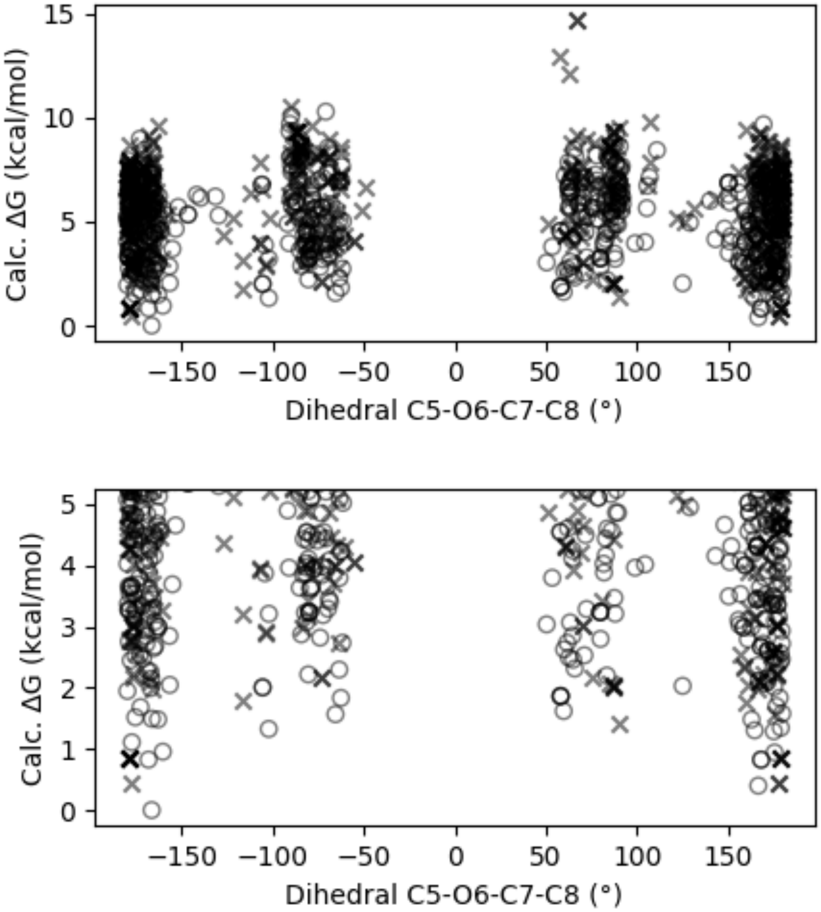

Figure S130. C5-C6-C7-C8 dihedral angles vs. calculated ΔG for conformers of **12’** optimized at the M06-2X/def2-TZVP/(SMD=iPrOH) level. O markers = conformers with *E* iminiums. X markers = conformers with *Z* iminiums.

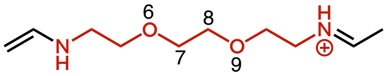

12’

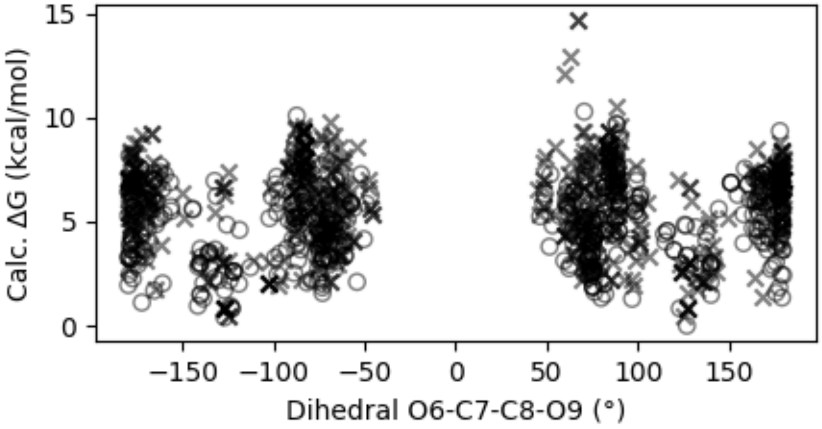

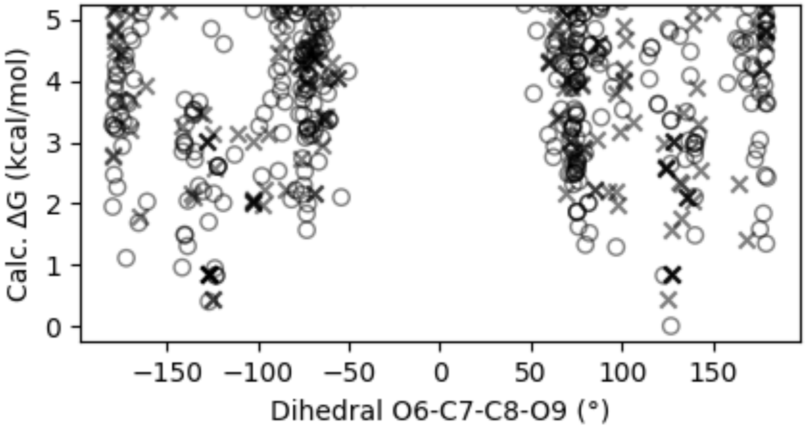

Figure S131. O6-C7-C8-O9 dihedral angles vs. calculated ΔG for conformers of **12’** optimized at the M06-2X/def2-TZVP/(SMD=iPrOH) level. O markers = conformers with *E* iminiums. X markers = conformers with *Z* iminiums.

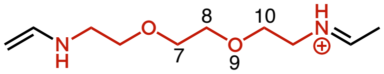

12’

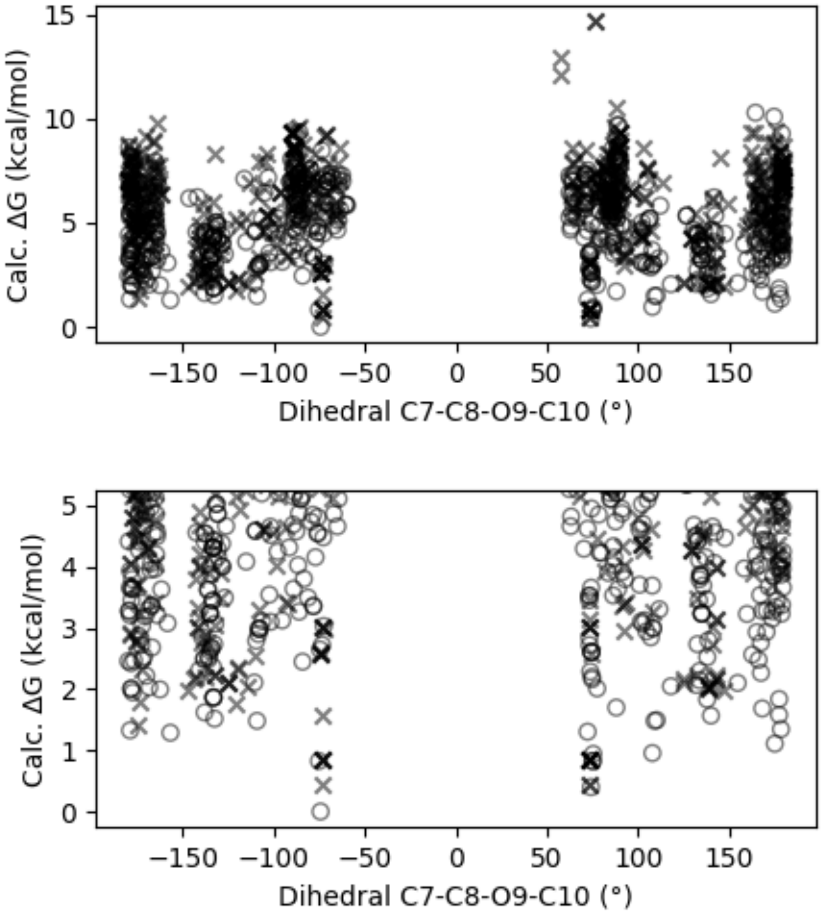

Figure S132. C7-C8-O9-C10 dihedral angles vs. calculated ΔG for conformers of **12’** optimized at the M06-2X/def2-TZVP/(SMD=iPrOH) level. O markers = conformers with *E* iminiums. X markers = conformers with *Z* iminiums.

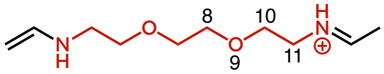

12’

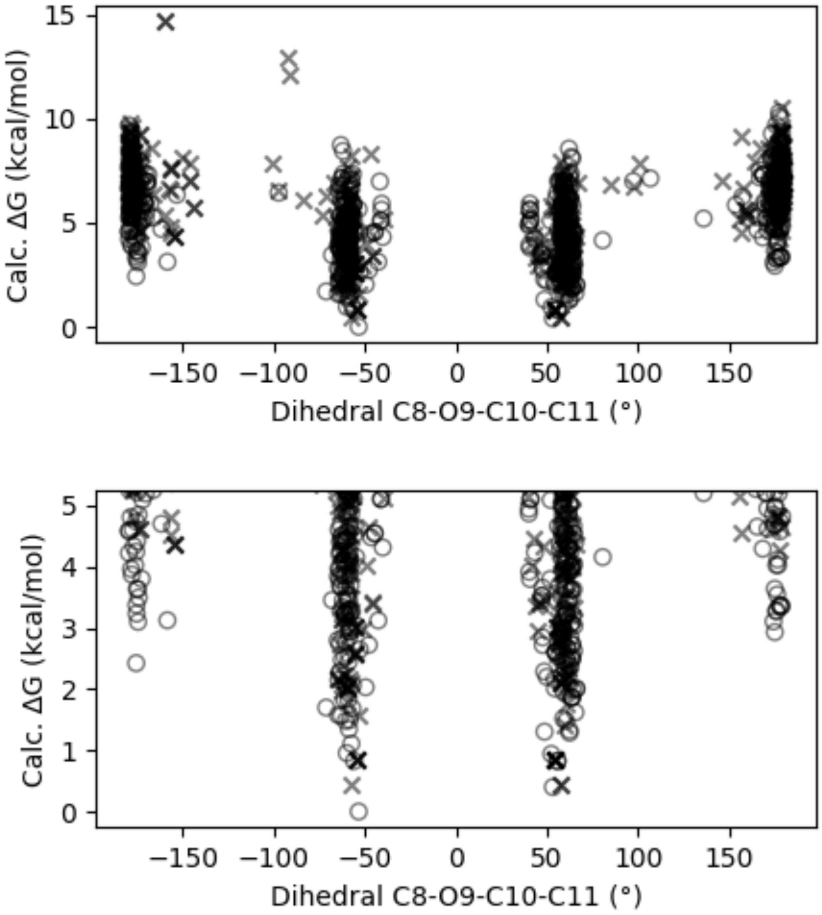

Figure S133. C8-O9-C10-C11 dihedral angles vs. calculated ΔG for conformers of **12’** optimized at the M06-2X/def2-TZVP/(SMD=iPrOH) level. O markers = conformers with *E* iminiums. X markers = conformers with *Z* iminiums.

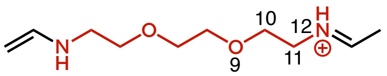

12’

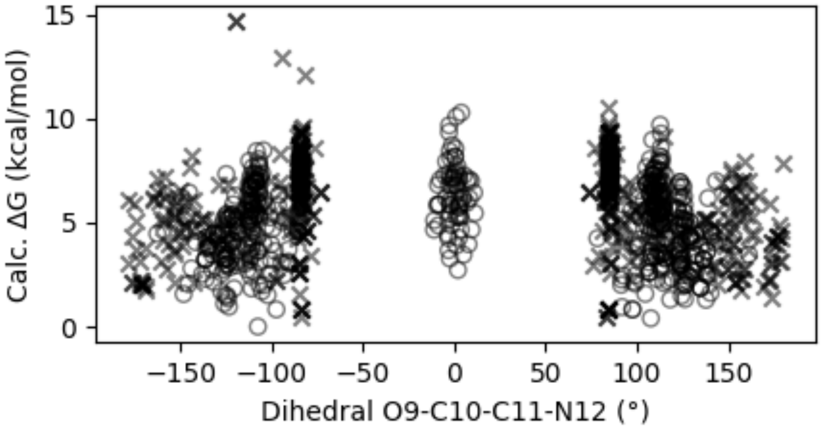

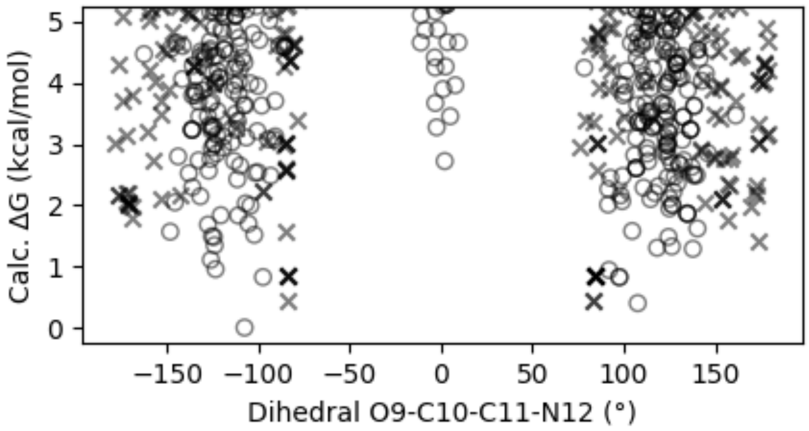

Figure S134. O9-C10-C11-N12 dihedral angles vs. calculated ΔG for conformers of **12’** optimized at the M06-2X/def2-TZVP/(SMD=iPrOH) level. O markers = conformers with *E* iminiums. X markers = conformers with *Z* iminiums.

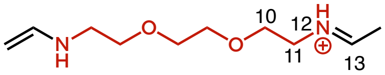

12'

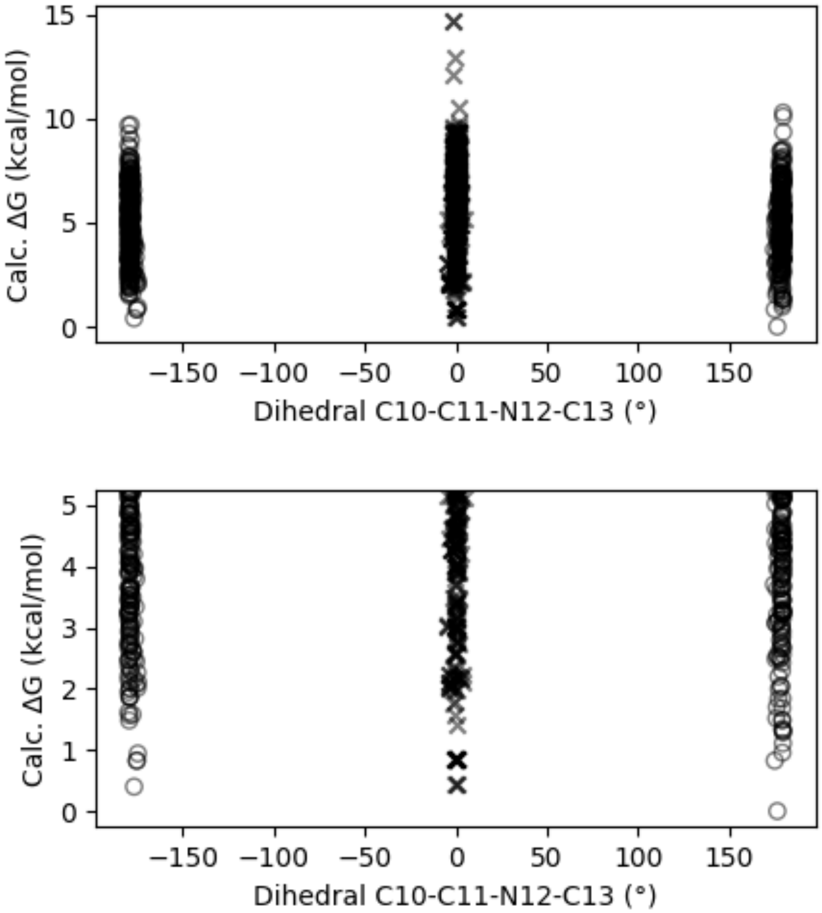

Figure S135. C10-C11-N12-C13 dihedral angles vs. calculated ΔG for conformers of **12'** optimized at the M06-2X/def2-TZVP/(SMD=iPrOH) level. O markers = conformers with *E* iminiums. X markers = conformers with *Z* iminiums.

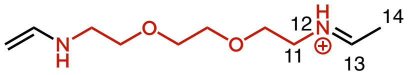

12’

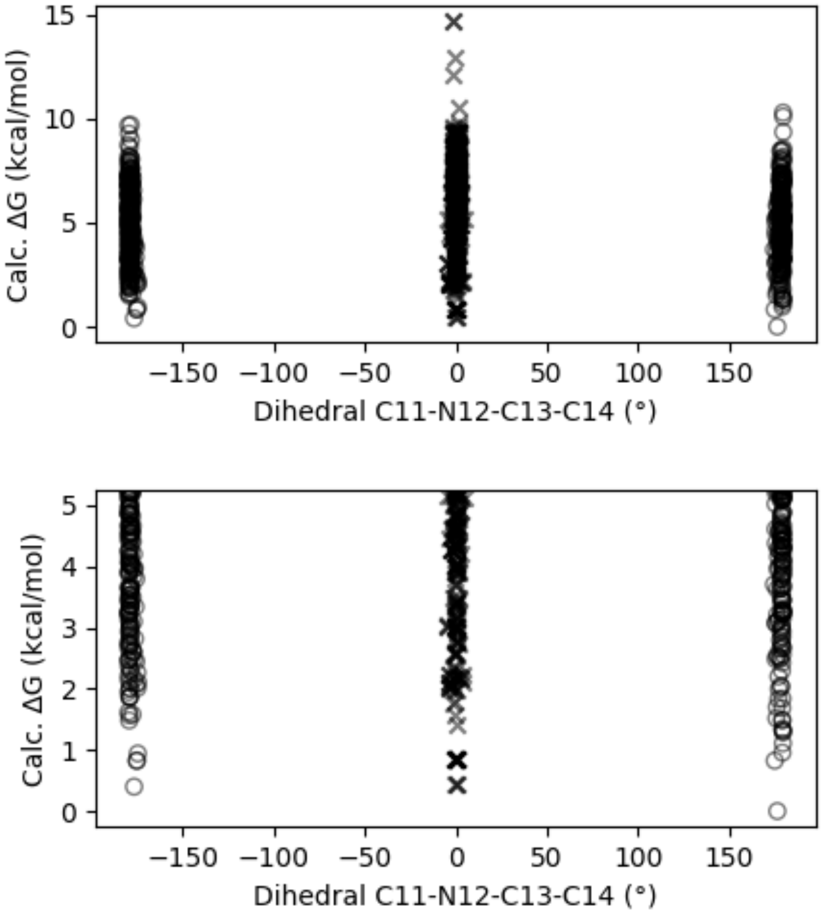

Figure S136. C11-N12-C13-C14 dihedral angles vs. calculated ΔG for conformers of **12’** optimized at the M06-2X/def2-TZVP/(SMD=iPrOH) level. O markers = conformers with *E* iminiums. X markers = conformers with *Z* iminiums.

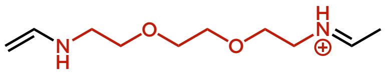

12’

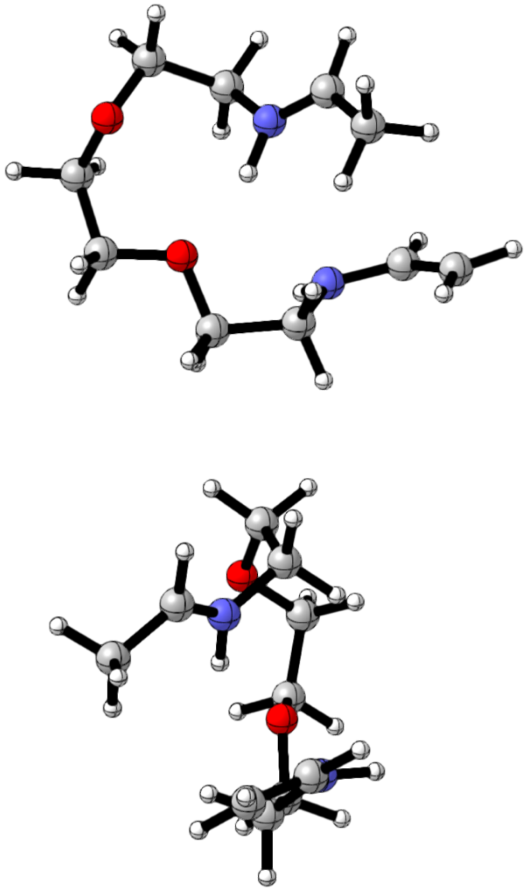

Figure S137. Front and side view of the lowest free energy conformer of **12’** at the M06-2X/def2-TZVP/(SMD=iPrOH) level.

7. NMR, MS and UPLC data

(*E*)-2-benzyl-3-(2,6-dimethoxyphenyl)acrylaldehyde ((*E*)-CA)

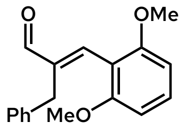

^{1}H NMR (600 MHz, $CDCl_3$):

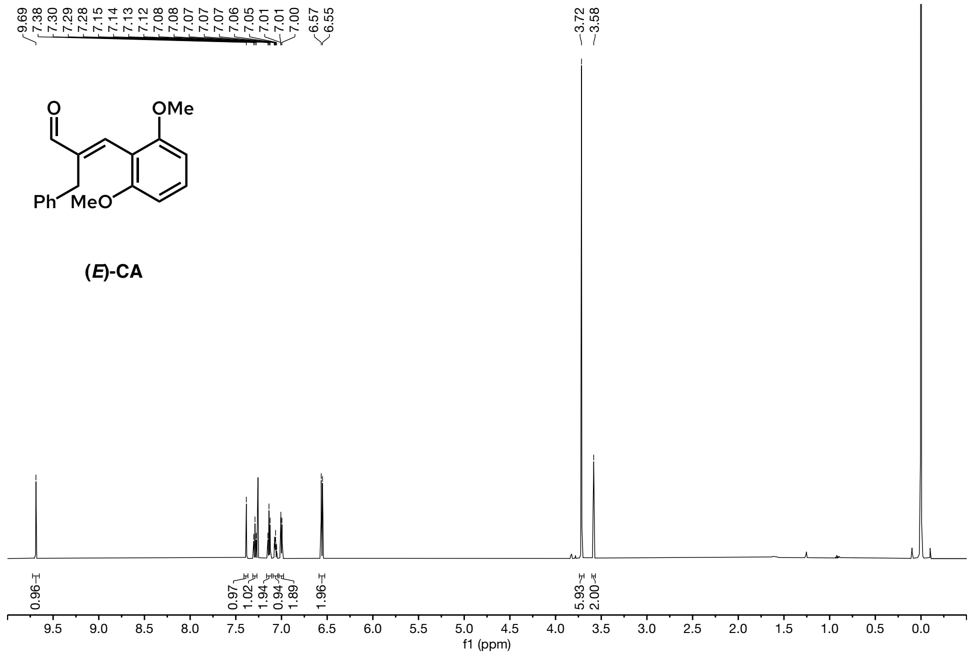

$^{13}C\{^1H\}$ NMR (151 MHz, $CDCl_3$):

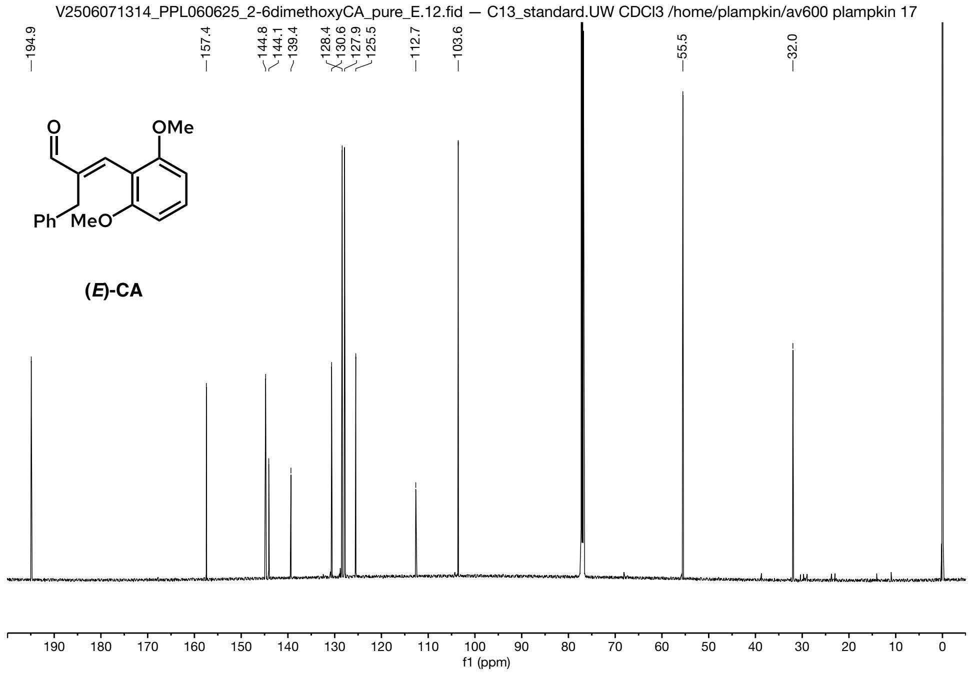

HR-ESI-MS:

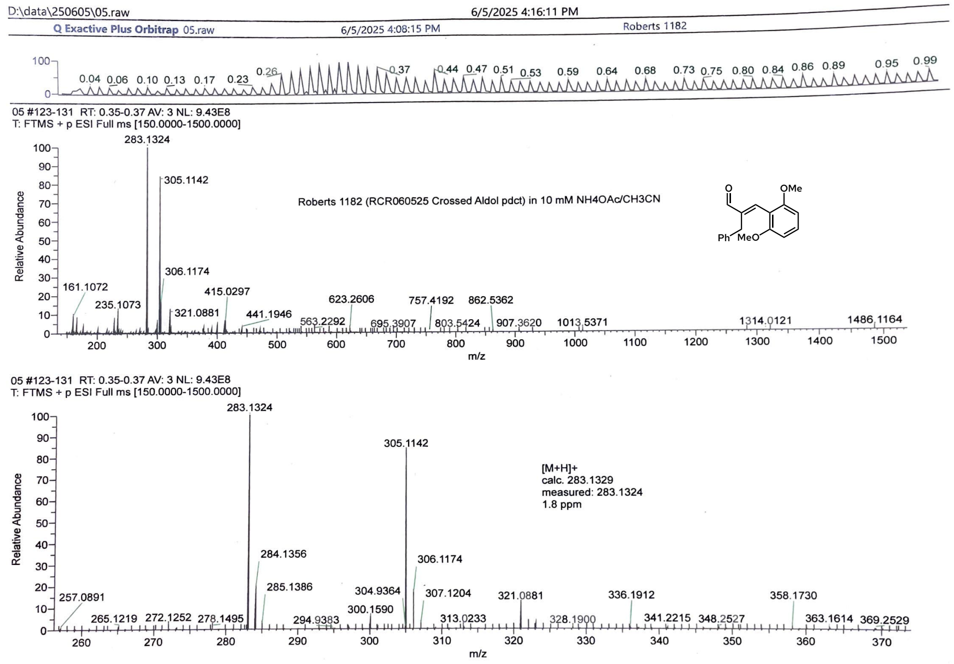

Acetyl-ACPC-ACPC-Dab-ACPC-ACPC-Dap-B[3]HTyr-C(O)NH$_2$ (9)

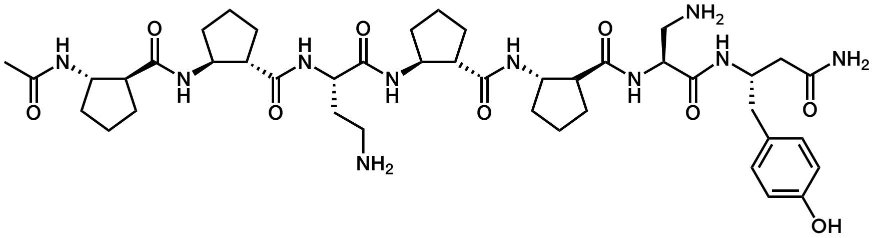

UPLC Trace (220 nm): 99% pure by integration.

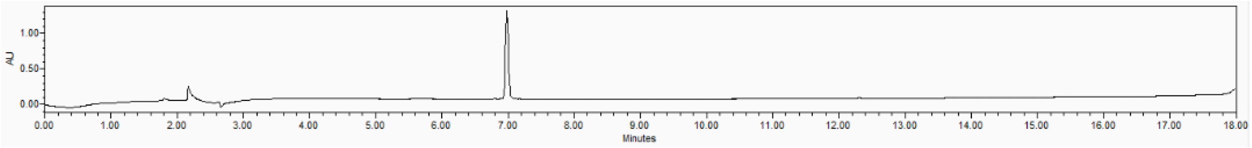

HR-ESI-MS:

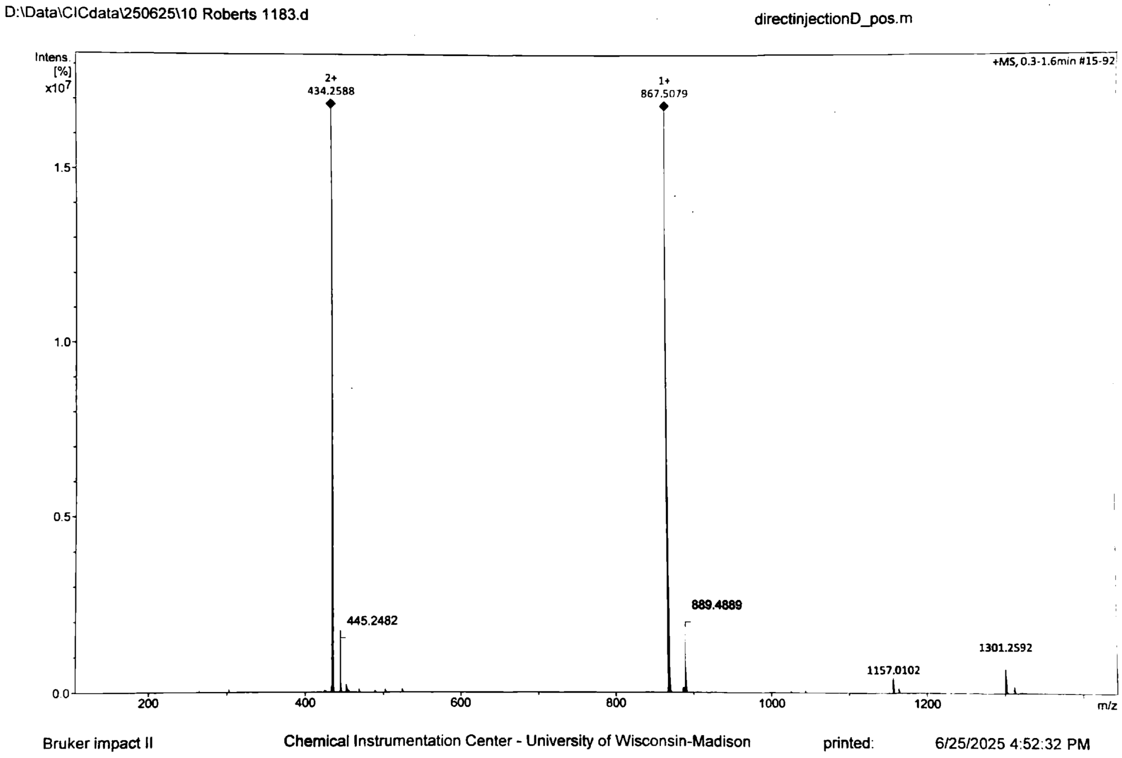

m/z observed: 867.5079 ([M+H]$^+$)

m/z calculated: $C_{42}H_{67}N_{10}O_9^+$ = 867.5087 ([M+H]$^+$)

Acetyl-ACPC-ACPC-Dab-ACPC-ACPC-Ala-B^{3}HTyr-C(O)NH$_2$ (16)

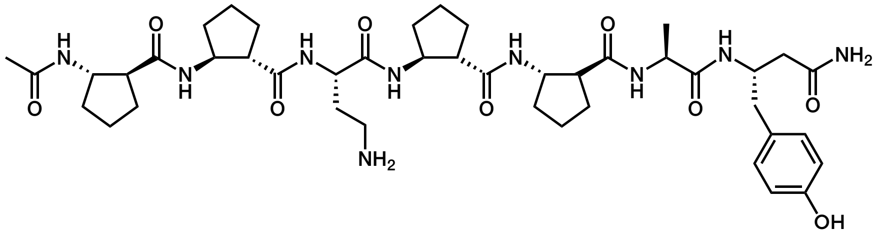

UPLC Trace (220 nm): 97% pure by integration

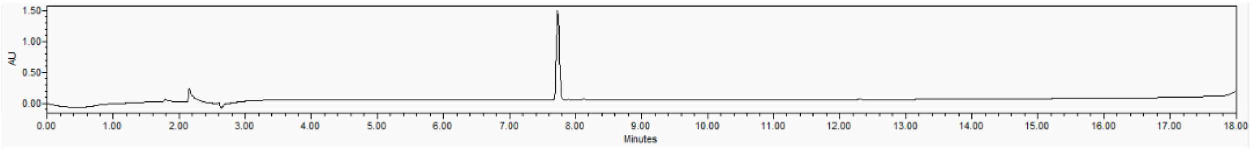

HR-ESI-MS:

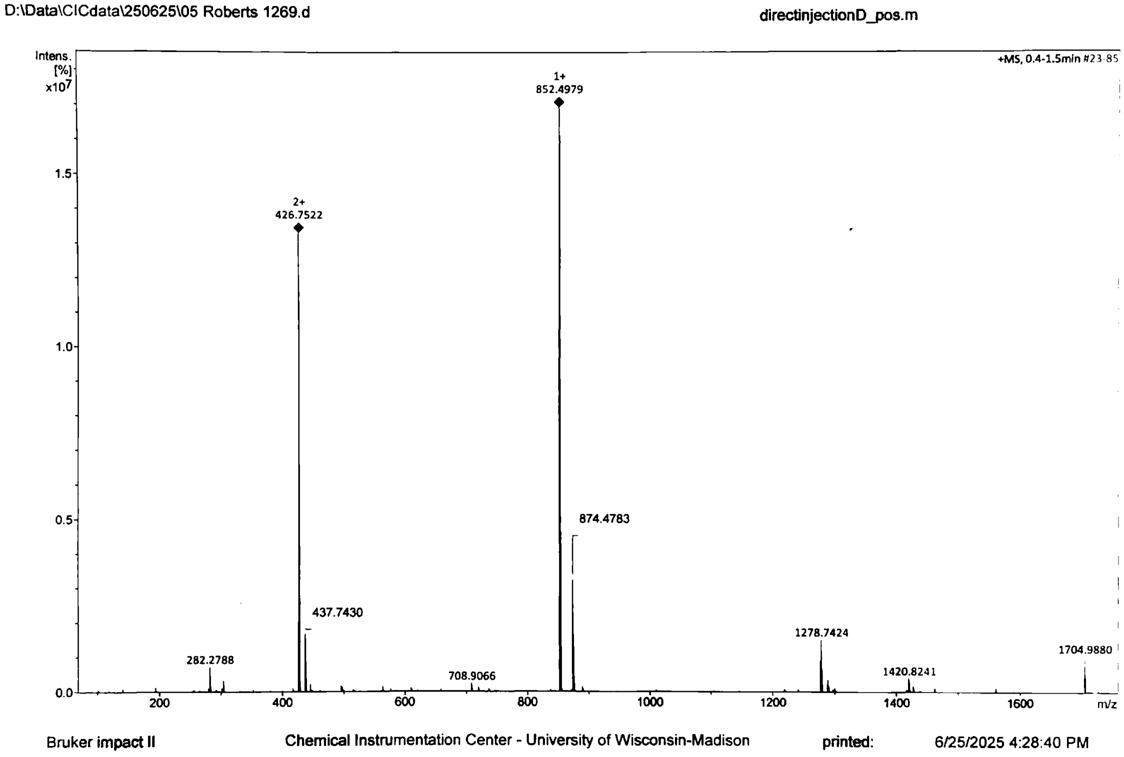

m/z observed: 852.4979 ([M+H]$^+$)

m/z calculated: $C_{43}H_{65}N_9O_9^+$ = 852.4978 ([M+H]$^+$)

Acetyl-ACPC-ACPC-Ala-ACPC-ACPC-Dap-B^{3}HTyr-C(O)NH$_2$ (17)

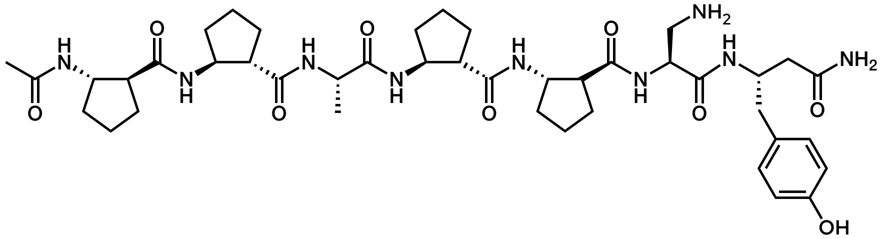

UPLC Trace (220 nm): 97% pure by integration

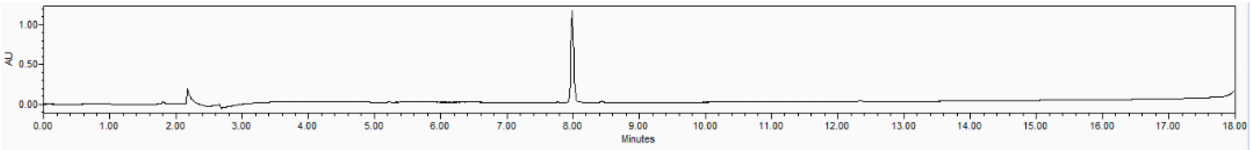

HR-ESI-MS:

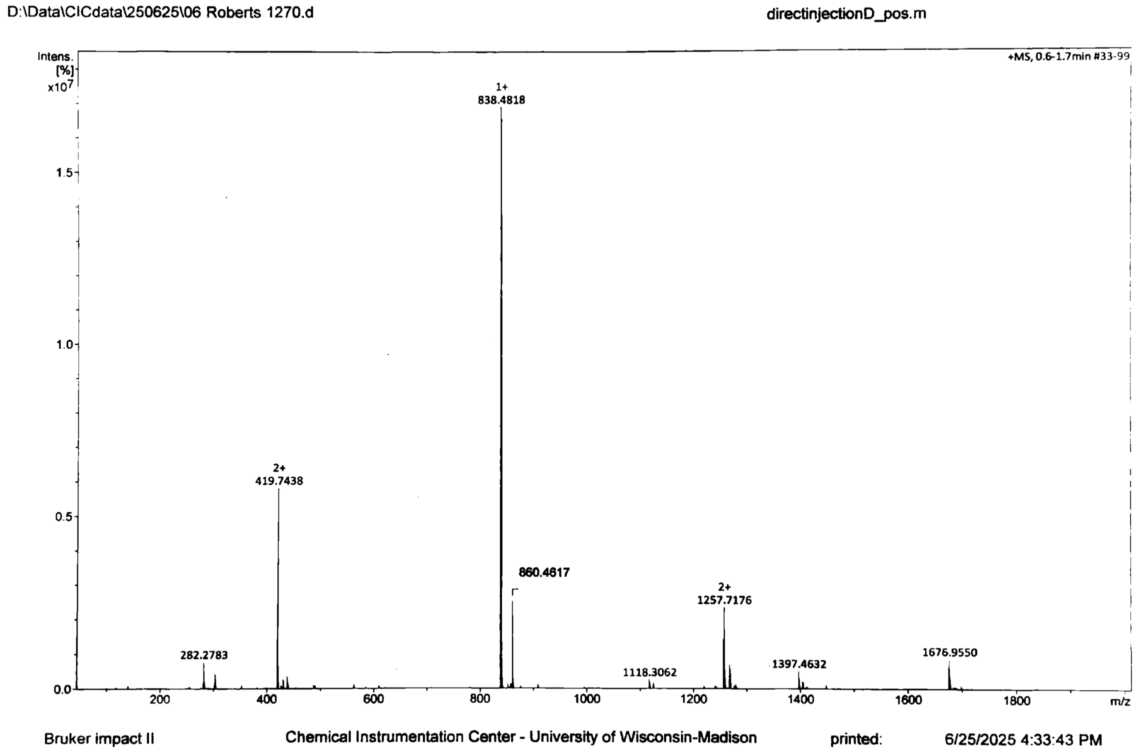

m/z observed: 838.4818 ([M+H]$^+$)

m/z calculated: $C_{42}H_{64}N_9O_9^+$ = 838.4822 ([M+H]$^+$)

8. References

(1) Andrews, M. K.; Liu, X.; Gellman, S. H. Tailoring Reaction Selectivity by Modulating a Catalytic Diad on a Foldamer Scaffold. *J. Am. Chem. Soc.* **2022**, *144*, 5, 2225–2232. https://doi.org/10.1021/jacs.1c11542.

(2) Lampkin, P. P.; Gellman, S. H. Dual Activation Modes Enable Bifunctional Catalysis of Aldol Reactions by Flexible Dihydrazides *J. Am. Chem. Soc.* **2025**, *147*, 4, 3731–3739. https://doi.org/10.1021/jacs.4c16143.

(3) Erkkilä, A.; Pihko, P. M. Rapid Organocatalytic Aldehyde-Aldehyde Condensation Reactions. *Eur. J. Org. Chem.* **2007**, *2007*, 25, 4205–4216. https://doi.org/10.1002/ejoc.200700292.

(4) Alamillo-Ferrer, C.; Hutchinson, G.; Burés, J. Mechanistic interpretation of orders in catalyst greater than one. *Nat. Chem. Rev.* **2023**, 7, 26-34. https://doi.org/10.1038/s41570-022-00447-w.

(5) *RDKit: Open-source cheminformatics – Release 2025.03.1*, Landrum, G., ETH Zurich, 2025 (https://doi.org/10.5281/zenodo.15115844).

(6) *Gaussian 16, Revision C.01* Frisch, M. J.; Trucks, G. W.; Schlegel, H. B.; Scuseria, G. E.; Robb, M. A.; Cheeseman, J. R.; Scalmani, G.; Barone, V.; Petersson, G. A.; Nakatsuji, H.; Li, X.; Caricato, M.; Marenich, A. V.; Bloino, J.; Janesko, B. G.; Gomperts, R.; Mennucci, B.; Hratchian, H. P.; Ortiz, J. V.; Izmaylov, A. F.; Sonnenberg, J. L.; Williams-Young, D.; Ding, F.; Lipparini, F.; Egidi, F.; Goings, J.; Peng, B.; Petrone, A.; Henderson, T.; Ranasinghe, D.; Zakrzewski, V. G.; Gao, J.; Rega, N.; Zheng, G.; Liang, W.; Hada, M.; Ehara, M.; Toyota, K.; Fukuda, R.; Hasegawa, J.; Ishida, M.; Nakajima, T.; Honda, Y.; Kitao, O.; Nakai, H.; Vreven, T.; Throssell, K.; Montgomery, J. A., Jr.; Peralta, J. E.; Ogliaro, F.; Bearpark, M. J.; Heyd, J. J.; Brothers, E. N.; Kudin, K. N.; Staroverov, V. N.; Keith, T. A.; Kobayashi, R.; Normand, J.; Raghavachari, K.; Rendell, A. P.; Burant, J. C.; Iyengar, S. S.; Tomasi, J.; Cossi, M.; Millam, J. M.; Klene, M.; Adamo, C.; Cammi, R.; Ochterski, J. W.; Martin, R. L.; Morokuma, K.; Farkas, O.; Foresman, J. B.; Fox, D. J. Gaussian, Inc., Wallingford CT, 2016.

(7) Dunning, T. H. Gaussian basis sets for use in correlated molecular calculations. I. The atoms boron through neon and hydrogen. *J. Chem. Phys.* **1989**, *90*, 1007–1023. https://doi.org/10.1063/1.456153.

(8) Kendall, R. A.; Dunning, T. H.; Harrison, R. J. Electron Affinities of the First-Row Atoms Revisited. Systematic Basis Sets and Wave Functions *J. Chem. Phys.* **1992**, 96, 6796-6806. http://doi.org/10.1063/1.462569.

(9) Zhao, Y.; Truhlar, D.G. The M06 suite of density functionals for main group thermochemistry, thermochemical kinetics, noncovalent interactions, excited states, and transition elements: two new functionals and systematic testing of four M06-class functionals and 12 other functionals. *Theor. Chem. Account*, **2008**, *120*, 215–241. https://doi.org/10.1007/s00214-007-0310-x.

(10) Schäfer, A.; Horn, H.; Ahlrichs, R. Fully optimized contracted Gaussian basis sets for atoms Li to Kr. *J. Chem. Phys.,* **1992**, 97, 2571-2577. https://doi.org/10.1063/1.463096.

(11) Marenich, A. V.; Cramer, C. J.; Truhlar, D. G.; Universal Solvation Model Based on Solute Electron Density and on a Continuum Model of the Solvent Defined by the Bulk Dielectric Constant and Atomic Surface Tensions. *J. Phys. Chem. B* **2009**, 113, 18, 6378–6396. https://doi.org/10.1021/jp810292n.

(12) *CYLview, 1.0b.* Legault, C. Y., Université de Sherbrooke, 2009 (http://www.cylview.org).

(13) '*torsion angle' in IUPAC Compendium of Chemical Terminology*, 5th ed. International Union of Pure and Applied Chemistry; 2025. (https://doi.org/10.1351/goldbook.T06406).

(14) Bursch, M.; Mewes, J.; Hansen, A.; Grimme, S. Best-Practice DFT Protocols for Basic Molecular Computational Chemistry** *Angewandte Chemie International Edition*, **2022**, 61, 52, 3202205735. https://doi.org/10.1002/anie.202205735

9. Instrumentation Funding Acknowledgements

Bruker D8 VENTURE Photon III Cu IμS 3.0 X-ray diffractometer – NSF grant CHE-1919350.

The Bruker NEO-500 NMR spectrometer – NSF grant CHE-2017891.

Bruker Impact II – Bender gift to the UW – Madison department of chemistry.
